# Epidemiology of gonorrhoea: systematic review, meta-analyses, and meta-regressions, World Health Organization European Region, 1949 to 2021

**DOI:** 10.2807/1560-7917.ES.2024.29.9.2300226

**Published:** 2024-02-29

**Authors:** Omar Chidiac, Sawsan AlMukdad, Manale Harfouche, Emma Harding-Esch, Laith J Abu-Raddad

**Affiliations:** 1 Infectious Disease Epidemiology Group, Weill Cornell Medicine-Qatar, Cornell University, Qatar Foundation - Education City, Doha, Qatar; 2 World Health Organization Collaborating Centre for Disease Epidemiology Analytics on HIV/AIDS, Sexually Transmitted Infections, and Viral Hepatitis, Weill Cornell Medicine–Qatar, Cornell University, Qatar Foundation – Education City, Doha, Qatar; 3 Clinical Research Department, Faculty of Infectious and Tropical Diseases, London School of Hygiene & Tropical Medicine, London, UK; 4 Department of Population Health Sciences, Weill Cornell Medicine, Cornell University, New York, New York, USA; 5 Department of Public Health, College of Health Sciences, QU Health, Qatar University, Doha, Qatar; 6 College of Health and Life Sciences, Hamad bin Khalifa University, Doha, Qatar; * These authors contributed equally to this work and share first authorship.

**Keywords:** Prevalence, Gonorrhoea, Europe, Synthesis, Region

## Abstract

**Background:**

Epidemiology of *Neisseria gonorrhoeae* (NG) infection remains inadequately understood.

**Aim:**

We aimed to characterise NG epidemiology in Europe.

**Methods:**

We used Cochrane and PRISMA guidelines to systematically review, report, synthesise and analyse NG prevalence data from 1949 to 30 September 2021. Random-effects meta-analyses estimated pooled prevalence. Meta-regression analyses investigated associations and sources of heterogeneity.

**Results:**

The 844 included publications yielded 1,573 prevalence measures. Pooled prevalence of current urogenital infection was 1.0% (95% CI: 0.7–1.2%) among general populations, 3.2% (95% CI: 1.8–4.8%) among female sex workers, 4.9% (95% CI: 4.2–5.6%) among sexually transmitted infection clinic attendees and 12.1% (95% CI: 8.8–15.8%) among symptomatic men. Among men who have sex with men, pooled prevalence was 0.9% (95% CI: 0.5–1.4%), 5.6% (95% CI: 3.6–8.1%), and 3.8% (95% CI: 2.5–5.4%), respectively, for current urogenital, anorectal or oropharyngeal infection. Current urogenital, anorectal or oropharyngeal infection was 1.45-fold (95% CI: 1.19–1.77%), 2.75-fold (95% CI: 1.89–4.02%) and 2.64-fold (95% CI: 1.77–3.93%) higher among men than women. Current urogenital infection declined 0.97-fold (95% CI: 0.96–0.98%) yearly, but anorectal and oropharyngeal infection increased (1.02-fold; 95% CI: 1.01–1.04% and 1.02-fold; 95% CI: 1.00–1.04%), respectively.

**Conclusions:**

*Neisseria gonorrhoeae* epidemiology in Europe has distinct and contrasting epidemiologies for vaginal sex transmission in heterosexual sex networks vs anal and oral sex transmission in MSM sexual networks. Increased transmission may facilitate drug-resistant strain emergence. Europe is far from achieving the World Health Organization target of 90% incidence reduction by 2030.

## Introduction

Gonorrhoea is a sexually transmitted infection (STI) caused by the bacterium *Neisseria gonorrhoeae* (NG), which infects exposed urogenital, anorectal or oropharyngeal mucosa [1,2]. NG infection is typically asymptomatic with most cases being undiagnosed and untreated, especially in women [1-3], leading to complications such as cervicitis, pelvic inflammatory disease, ectopic pregnancy and infertility [1,2,4,5]. In 2016, the global prevalence of NG among adults 15–49 years of age was estimated by the World Health Organization (WHO) at 0.9% in women and 0.7% in men [6]. Reports suggested recent increases in NG infection incidence in several countries in northern and western Europe as well as in North America [7,8].

In alignment with its third Sustainable Development Goal on good health and wellbeing, the WHO formulated its Global Health Sector Strategy on STIs to reduce the burden of STIs as a major public health concern [9,10]. This strategy aims to achieve 90% reduction in NG infection incidence globally by 2030, by increasing access to quality diagnostic, therapeutic and preventive services and by implementing evidence-based interventions. Furthermore, understanding and characterising the epidemiology of NG infection contributes to the broader effort of improving public health and reducing the impact of infectious diseases on individuals and communities.

The global health threat associated with gonorrhoea has intensified over the past two decades due to the widespread occurrence of gonococcal antimicrobial resistance (AMR) and the emergence of extensively drug-resistant NG strains [11-14]. This includes strains resistant to extended-spectrum cephalosporins, which currently serve as the last line of defence against this infection [2,11,12,15]. Consequently, the WHO declared gonococcal AMR a global high priority [16] and initiated a global action plan to control NG transmission [17]. However, recent reports of meningococcal vaccines being partially protective against acquisition of NG [18-21] have spurred optimism for the development of an NG vaccine, which may overcome the AMR setbacks in controlling NG transmission.

Against this background, this study aimed to characterise NG epidemiology in Europe by systematically reviewing and synthesising publications on NG prevalence, estimating pooled mean prevalence and assessing temporal trends of, and associations with, NG prevalence.

## Methods

The protocol of this study was not registered in PROSPERO because the methods were adapted from our existing protocol [22] for a systematic review of NG epidemiology in infertile populations [5], and more broadly, from our previously published systematic reviews of the prevalence of other STIs [23-31].

### Data sources and search strategy

The systematic literature review was informed by the Cochrane Collaboration Handbook [32], and findings were reported following the Preferred Reporting Items for Systematic Reviews and Meta-analyses (PRISMA) guidelines [33,34]. The 27-item PRISMA checklist, outlining essential elements for reporting in a systematic review, is available in Supplementary Table S1.

The systematic literature search was conducted in PubMed and Embase databases until 30 September 2021, using search strategies with exploded Mesh/Emtree terms, broad search criteria and free text terms with no language or year restrictions. The search strategies for PubMed and Embase are provided in Supplementary Box S1. Definition of Europe included 53 countries and one territory, Greenland, and was informed, along with the subregional classification of countries, by the WHO and United Nations Geoscheme (Box 1) [35,36].

Box 1 **List of the 53 countries and one territory included in the definition of Europe along with their subregional classification**

Eastern Europe: Belarus, Bulgaria, Czechia, Hungary, Poland, Republic of Moldova, Romania, Russian Federation, Slovakia and Ukraine.
Northern Europe: Denmark, Estonia, Finland, Greenland, Iceland, Ireland, Latvia, Lithuania, Norway, Sweden and United Kingdom.
Southern Europe: Albania, Andorra, Bosnia and Herzegovina, Croatia, Greece, Italy, Malta, Montenegro, Portugal, North Macedonia, San Marino, Serbia, Slovenia, and Spain.
Western Europe: Austria, Belgium, France, Germany, Luxembourg, Monaco, the Netherlands and Switzerland.
Intersection of Europe and Asia: Armenia, Azerbaijan, Cyprus, Georgia, Kazakhstan, Kyrgyzstan, Tajikistan, Turkmenistan and Uzbekistan.
Israel.
Türkiye.

### Study selection and eligibility criteria

Search results were imported into Endnote (Clarivate, Philadelphia, United States), where duplicate records were identified and removed. For the remaining records, titles and abstracts were first screened for potential relevance and then full texts of relevant and potentially relevant publications were retrieved and assessed. At least two reviewers independently screened each record with the screening split among three reviewers (OC, SM and MH). Discrepancies in screening were settled by consensus, including the three reviewers and author LJA.

Grey literature was not systematically searched. However, bibliographies of eligible articles and reviews were screened to identify additional potentially relevant publications. Among these, grey literature reports were eligible for inclusion in the study if they included relevant data.

A publication was eligible for inclusion if it reported data collected based on specimens directly obtained from humans and tested for NG infection using laboratory methods. Any publication that relied on patient self-reporting of infection as a method of diagnosis, included fewer than 10 study participants or tested tissue samples from upper genital tracts was excluded. Case reports, series, commentaries, reviews and qualitative studies were also excluded.

Although a study sample size as small as 10 participants is insufficient for a reliable individual measure of NG prevalence, these smaller studies were still included in this review. This decision was made to ensure inclusivity because the goal was to pool numerous prevalence measures through meta-analysis. While a study with only 10 participants might lack statistical precision on its own, it contributes statistical value when pooled with multiple other studies.

In this review, the term ‘publication’ refers to a document reporting one or more outcome measures (NG prevalence measures), whereas the term ‘study’ specifically pertains to an individual outcome measure. Studies that were duplicated or overlapped were included only once. A study was defined in this manner because a single publication may include several prevalence measures on different populations using different surveys and methods, such as one publication reporting the results of two surveys among two different populations. Since the outcome of this review is the prevalence of infection, it was deemed best to define a study as one prevalence measure in a specific population.

### Data extraction and synthesis

Retrieved records and articles were independently extracted and double-extracted with the work split among three authors (OC, SM, and MH). Extracted variables are listed in Supplementary Box S2. Discrepancies were discussed in consultation with LJA to reach consensus. Overall outcome measures (i.e. encompassing the entire sample) and their stratified measures were extracted provided sample size in each stratum was at least 10. Stratification hierarchy for prevalence measures in descending order of priority was: anatomical site, population type, sex, year of data collection, age group and region/city. Definitions of population-type classifications are explained in Box 2. As the study design centred on NG prevalence as the outcome, we did not extract data on resistance prevalence or the number of isolates for NG AMR from the included studies.

Box 2 **Definitions of population-type classifications**

1. General populations (populations at low risk): these include populations at lower risk of exposure to *Neisseria gonorrhoeae*, such as antenatal clinic attendees, blood donors and pregnant women, among others.
2. Intermediate-risk populations: these include populations who presumably have frequent sexual contacts with populations engaging in high sexual risk behaviour and have therefore a higher risk of exposure to *N. gonorrhoeae* than the general population. These comprise people who are incarcerated, people who inject drugs and people driving trucks, among others.
3. Female sex workers and women who have sex with women: these include reproductive-age women that are engaged in sex work, that is the exchange of sex for money (sex work as a profession) and women who engage in same-sex sexual activities.
4. Men who have sex with men and male sex workers: these include men who engage in same-sex sexual activities, specifically anal sex, and men who are engaged in providing anal-sex sexual services in return for payment.
5. Transgender people and transgender sex workers: these include populations whose gender identity is different from the sex that they were assigned at birth and populations with unspecified gender who are engaged in providing sexual services in return for payment.
6. HIV-positive individuals and individuals in HIV-discordant couples: these include populations who are HIV-positive or are in a spousal relationship with an HIV-positive individual.
7. Sexually transmitted infection clinic attendees: these include patients attending STI clinics or have clinical manifestations related to an STI.
8. Infertility clinic attendees: these were included in a separate category given the uncertainty around their risk of exposure to *N. gonorrhoeae* and the possible biological link between *N. gonorrhoeae* infection and infertility.
9. Women with miscarriage or ectopic pregnancy: these were included in a separate category given the uncertainty around their risk of exposure to *N. gonorrhoeae* and the possible biological link between *N. gonorrhoeae* infection and miscarriage or ectopic pregnancy.
10. Symptomatic women: these include women with clinical manifestations related to *N. gonorrhoeae* infection or suspected of having *N. gonorrhoeae* infection, such as those with vaginal discharge.
11. Symptomatic men: these include men with clinical manifestations related to *N. gonorrhoeae* infection or suspected of having *N. gonorrhoeae* infection, such as those with urethral discharge.
12. Symptomatic mixed sexes: these include populations without the sex being specified but with clinical manifestations related to *N. gonorrhoeae* infection or suspected of having *N. gonorrhoeae* infection, such as those with vaginal discharge or urethral discharge.
13. Sexual contacts of persons infected with *N. gonorrhoeae* or *Chlamydia trachomatis*: these include populations who are in sexual contact with persons infected with *N. gonorrhoeae* and/or *C. trachomatis*.
14. Patients with confirmed/suspected STIs and related infections: these include populations who are diagnosed with an STI or suspected to have concomitant^a ^STIs or other related infections.
15. Other populations: these include populations not satisfying above definitions, or populations with an undetermined risk of acquiring *N. gonorrhoeae* such as cervical cancer patients, victims of sexual assault, specimens from virology/bacteriology laboratory and requesting home-based *N. gonorrhoeae* or *C. trachomatis *testing.

STI: sexually transmitted infection.^a^ Includes, for example, patients with suspected NG, patients with suspected STI and patients with suspected genital tract infection.

Since the aim was to understand the natural heterogeneity that exists in NG epidemiology, such as the variation in prevalence by population type and anatomical site, both overall measures and stratified measures were extracted from relevant studies. Meta-regression analyses were conducted to estimate effects of epidemiological factors on prevalence of infection (note below). This analytical approach allows the generation of concrete inferences about the epidemiology of this infection based on understanding the sources of variation that exist in available measures.

Studies that used different assays on the same biological specimens were extracted and pooled separately in the meta-analysis to estimate NG prevalence by diagnostic method. These data were also incorporated into the meta-regression analyses to investigate the effects of diagnostic methods on observed NG prevalence. This approach was adopted to examine the assay impact on the heterogeneity of NG prevalence and to generate STI-estimation adjustment factors based on assay type. These factors can inform future mathematical modelling studies forecasting NG infection and disease burden metrics [37-39].

Studies reporting the same diagnostic test on different biological specimens in a defined population were included only once based on a pre-set stratification hierarchy for women (endocervical swabs, followed by vaginal swabs and urine samples) and for men (urethral swabs, followed by urine and semen samples) [23].

### Precision and risk of bias assessments

The precision and risk of bias (ROB) assessments of the included studies were guided by the Cochrane approach [32], pertinent quality components in prevalence studies [40] and a methodology honed through a series of systematic reviews focusing on STI prevalence [5,22-31]. This methodology, tailored and refined for the research questions in the present study, comprised one component for study precision and two components for ROB.

Other components were excluded, either because they were inherently met by our study design and inclusion/exclusion criteria, or because they were investigated under a different but more relevant research question within our study, as explained in Supplementary Table S2. For instance, the assessment of the validity and reliability of the study instrument measuring the parameter of interest [40] was implicitly implemented through the meta-regression analyses (note below), where we explored the impact of assay type on observed prevalence.

A study’s precision was classified as low vs high, based on the study sample size (< 200 vs ≥ 200). For an expected NG prevalence of ca 1% in the general population and a sample size of 200, the 95% confidence interval (CI) is 0–3.6% [41], which provides an acceptable level of precision for a prevalence measure [23]. Studies were classified as having low vs high ROB based on the following two domains: sampling methodology (probability vs non-probability-based sampling) and response rate (≥ 80% vs < 80%). Data on precision and ROB were used to provide summary statistics of the precision and ROB of studies. These data were also included in the meta-regression analyses to investigate their effects on observed prevalence.

### Meta-analyses

Dersimonian–Laird random-effects models were used to conduct meta-analyses [42] with the Freeman–Tukey double arcsine transformation to stabilise the variance [43] after ensuring the applicability of this transformation [44]. Pooled estimates for NG prevalence were calculated if each analysis stratum had at least three measures. Given the application of random-effects meta-analysis, a minimum count of three studies was set to conduct a meta-analysis, for the stability of the pooled estimate. Considering heterogeneity in prevalence measures, these pooled means are meant to provide an average summary measure of prevalence for each population and anatomical site. The sources of heterogeneity were investigated through meta-regression analyses as indicated below.

Cochran’s Q statistic was used to examine the presence of heterogeneity across studies. The I^2^ statistic was calculated to assess the magnitude of between-study variation due to true differences in prevalence rather than sampling variation. The prediction interval was estimated to describe the distribution of true prevalence around the pooled mean [42,45].

Cumulative meta-analyses, using the year of publication as the ordering variable, were also conducted to confirm the trend in NG prevalence generated by the meta-regression analyses. All meta-analyses were conducted in R version 4.1.2 [46] using the meta package [47].

### Meta-regressions

Univariable and multivariable random-effects meta-regression analyses of log-transformed proportions were conducted to investigate the sources of between-study heterogeneity and possible predictors of higher NG prevalence. These predictors were set a priori based on epidemiological relevance and knowledge of HIV or STI epidemiology [5,23,24,30,31]. Predictors are listed in Supplementary Box S3.

Sensitivity analyses were performed (i) to validate findings from the main analyses, where, for each anatomical site, the year of publication was incorporated into the models instead of the year of data collection; and (ii) to examine whether the results differed based on different diagnostic methods. Here, for each of the urogenital, anorectal and oropharyngeal datasets, the meta-regression analyses were re-run separately for the NAAT/PCR, culture and Gram staining datasets, totalling nine additional meta-regression analyses. 

Variables with a p value ≤ 0.10 in the univariable analysis were included in the multivariable analysis. Associations in the multivariable analysis with a p value ≤ 0.05 were considered to provide evidence of statistically significant associations. Meta-regressions were conducted in Stata/SE version 16.1 using the metareg package [48].

## Results

### Search results

The study selection process is illustrated in the Figure. A total of 18,987 records were identified, 7,984 from PubMed and 11,003 from Embase. After de-duplication and title and abstract screening, 5,009 unique citations were identified as relevant or potentially relevant for further screening. Full text screening of these citations identified 824 relevant publications. Bibliographic screening of eligible articles and reviews yielded 20 additional publications. In total, 844 publications met the inclusion criteria (these publications are listed in Supplementary Box S4). Extracted NG prevalence measures included 1,573 overall measures and 2,199 stratified measures.

Apart from 28 prevalence measures, 1,545 measures pertained to current NG infection, assessing current urogenital, anorectal or oropharyngeal NG prevalence. The remaining 28 studies assessed ever infection prevalence through serological testing.

**Figure:**
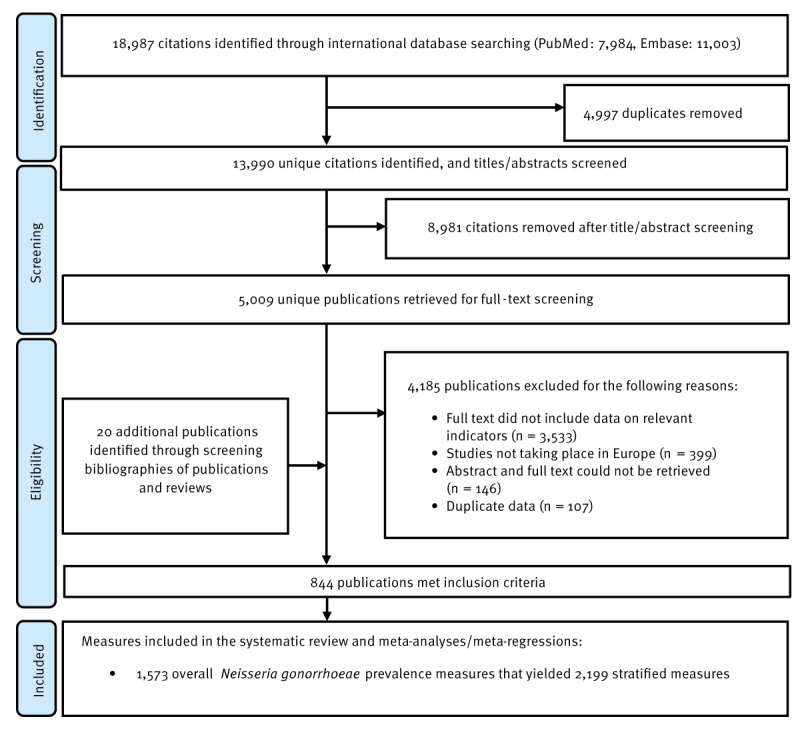
Flowchart of article selection for the systematic review of *Neisseria gonorrhoeae* infection in World Health Organization European Region countries, 1949–2021

Selection was carried out as per PRISMA guidelines [34].

### Scope of evidence for the prevalence measures

The earliest extracted study was published in 1949, and 507 studies (32.2%) were published before 2000, 226 studies (14.4%) between 2000 and 2009, and 840 studies (53.4%) starting from 2010. Most studies were based on convenience sampling (n = 1,519, 96.6%). The included studies encompassed various study designs, including cross-sectional (n = 1,470), case–control (n = 53), cohort (n = 29) and randomised controlled trials (n = 21). In the case of the latter two study designs, the included prevalence measures refer to the baseline measurements conducted at the beginning of the respective studies.

The number of NG prevalence measures categorised by European subregion and country are listed in Supplementary Table S3. The stratified NG prevalence measures are summarised by population type, anatomical site and assay type in Table 1, Table 2 and Table 3, including ranges and medians.

**Table 1:** Pooled estimates for *Neisseria gonorrhoeae* prevalence in general populations, intermediate-risk populations, infertility clinic attendees and other populations, World Health Organization European Region countries, 1949–2021

Population type		Outcome measures	Sample size	NG prevalence (%)		Pooled NG prevalence		Heterogeneity measures				
		Total n	Total n	Range	Median	Mean (%)	95% CI	Cochran’s Q statistic^a^		I^2b^		Prediction interval^c^ (%)
								Q	p-value	I^2^ (%)	95% CI	
**General populations**												
Current urogenital infection	NAAT/PCR	133	246,316	0.0–15.9	0.7	1.3	0.9–1.7	4,665.4	p < 0.001	97.2	96.9–97.4	0.0–9.0
	Culture	110	387,709	0.0–13.0	0.5	0.7	0.5–1.0	6,669.6	p < 0.001	98.4	98.2–98.5	0.0–5.0
	Gram staining	6	3,573	0.0–4.5	1.7	1.4	0.1–3.5	57.9	p < 0.001	91.4	84.0–95.3	0.0–11.6
	Other^d^	13	7,315	0.0–0.8	0.2	0.4	0.2–0.6	8.5	p = 0.745	0.0	0.0–56.6	0.1–1.0
	**Overall**	**262**	**644,913**	**0.0–15.9**	**0.6**	**1.0 **	**0.7–1.2**	**12,242.7 **	**p < 0.001**	**97.9 **	**97.7–98.0**	**0.0–6.9**
Current anorectal infection	NAAT/PCR	2	499	2.9–5.4	4.2	4.0	1.9–6.8	NA		NA		NA
	Culture	1^e^	759	NA	NA	0.4	0.1–1.0	NA		NA		NA
	**Overall**	**3**	**1,258**	**0.4–5.4**	**2.9**	**2.3 **	**0.2–6.3**	**24.3 **	**p < 0.001**	**91.8 **	**79.0–96.8**	**0.0–97.1**
Current oropharyngeal infection	NAAT/PCR	3	475	0.0–4.4	0.0	0.9	0.0–4.6	12.8	p = 0.002	84.4	53.3–94.8	0.0–99.4
	**Overall**	3	475	0.0–4.4	0.0	0.9	0.0–4.6	12.8	p = 0.002	84.4	53.3–94.8	0.0–99.4
Unspecified/mixed anatomical site	NAAT/PCR	28	297,953	0.0–4.9	1.0	0.9	0.6–1.3	901.4	p < 0.001	97.0	96.4–97.5	0.0–3.4
	Culture	33	1,763,032	0.0–2.7	0.6	0.5	0.3–0.8	14,871.0	p < 0.001	99.8	99.8–99.8	0.0–2.4
	Gram staining	1^e^	100	NA	NA	0.0	0.0–1.7	NA		NA		NA
	Other^d^	62	5,521,391	0.0–8.0	0.6	0.6	0.3–0.9	6,897.5	p < 0.001	99.1	99.0–99.2	0.0–4.7
	**Overall**	**125**	**7,582,476**	**0.0–8.0**	**0.7**	**0.6 **	**0.4–0.8**	**36,040.4 **	**p < 0.001**	**99.7 **	**99.6–99.7**	**0.0–3.6**
Sera	Blood tested for antibodies	13	1,780	0.5–51.4	11.1	11.4	5.3–19.2	246.9	p < 0.001	95.1	93.2–96.5	0.0–48.9
**Intermediate-risk populations**												
Current urogenital infection	NAAT/PCR	16	5,169	0.0–30.4	0.5	3.1	0.5–7.6	607.6	p < 0.001	97.5	96.8–98.1	0.0–33.8
	Culture	7	916	0.0–15.4	0.0	1.3	0.0–5.0	44.1	p < 0.001	86.4	74.1–92.8	0.0–20.7
	Other^d^	1^e^	474	NA	NA	0.2	0.0–0.9	NA		NA		NA
	**Overall**	**24**	**6,559**	**0.0–30.4**	**0.2**	**2.4 **	**0.5–5.2**	**680.5 **	**p < 0.001**	**96.6 **	**95.8–97.3**	**0.0–25.7**
Current anorectal infection	NAAT/PCR	2^e^	141	0.0–21.7	10.9	5.7	0.0–41.9	NA		NA		NA
	**Overall**	**2^e^**	**141**	**0.0–21.7**	**10.9**	**5.7 **	**0.0–41.9**	**NA**		**NA**		**NA**
Current oropharyngeal infection	NAAT/PCR	1^e^	23	NA	NA	17.4	4.2–36.0	NA		NA		NA
	**Overall**	**1^e^**	**23**	**NA**	**NA**	**17.4 **	**4.2–36.0**	**NA**		**NA**		**NA**
Unspecified/mixed anatomical site	NAAT/PCR	2^e^	2,816	0.2–1.0	0.6	0.5	0.0–1.6	NA		NA		NA
	Culture	3	443	1.3–4.6	2.2	2.4	0.8–4.8	3.3	p = 0.195	38.7	0.0–80.9	0.0–48.7
	Other^d^	4	647	0.0–3.1	0.0	0.3	0.0–1.7	9.4	p = 0.050	67.9	6.8–89.0	0.0–13.2
	**Overall**	**9**	**3,906**	**0.0–4.6**	**1.0**	**0.8 **	**0.1–1.9**	**33.2 **	**p < 0.001**	**75.9 **	**53.8–87.5**	**0.0–5.8**
Sera	Blood tested for antibodies	1^e^	10	NA	NA	0.0	0.0–16.5	NA		NA		NA
**Infertility clinic attendees**												
Current urogenital infection	NAAT/PCR	10	1,023	0.0–0.7	0.0	0.0	0.0–0.2	3.1	p = 0.962	0.0	0.0–62.4	0.0–0.3
	Culture	34	5,125	0.0–33.3	0.0	1.2	0.2–2.9	571.1	p < 0.001	94.2	92.8–95.3	0.0–17.7
	Other^d^	7	1,002	0.0–23.8	0.0	3.5	0.0–10.6	65.2	p < 0.001	90.8	83.6–94.8	0.0–39.9
	**Overall**	**51**	**7,150**	**0.0–33.3**	**0.0**	**1.1 **	**0.3–2.4**	**704.6 **	**p < 0.001**	**92.9 **	**91.4–94.1**	**0.0–15.7**
Unspecified/mixed anatomical site	Other^d^	5	1,199	3.4–62.2	7.0	18.5	3.0–42.7	361.0	p < 0.001	98.9	98.4–99.2	0.0–99.7
	**Overall**	**5**	**1,199**	**3.4–62.2**	**7.0**	**18.5 **	**3.0–42.7**	**361.0 **	**p < 0.001**	**98.9 **	**98.4–99.2**	**0.0–99.7**
Sera	Blood tested for antibodies	9	759	0.0–60.6	14.5	16.0	5.7–29.8	107.9	p < 0.001	92.6	88.1–95.4	0.0–72.7
**Other populations^f^**												
Current urogenital infection	NAAT/PCR	17	21,639	0.0–12.0	2.7	3.7	2.2–5.5	526.5	p < 0.001	97.0	96.1–97.6	0.0–14.2
	Culture	8	2,076	0.0–44.1	7.5	8.3	1.1–20.8	200.4	p < 0.001	96.5	94.8–97.7	0.0–66.6
	**Overall**	**25**	**23,715**	**0.0–44.1**	**2.7**	**4.7 **	**2.4–7.7**	**822.3 **	**p < 0.001**	**97.1 **	**96.4–97.6**	**0.0–26.8**
Current anorectal infection	NAAT/PCR	6	1,295	1.0–30.0	8.2	5.6	1.3–11.9	45.8	p < 0.001	89.1	78.9–94.4	0.0–33.4
	Culture	1^e^	53	NA	NA	0.0	0.0–3.2	NA		NA		NA
	**Overall**	**7**	**1,348**	**0.0–30.0**	**7.0**	**4.4 **	**0.8–10.0**	**47.5 **	**p < 0.001**	**87.4 **	**76.2–93.3**	**0.0–29.3**
Current oropharyngeal infection	NAAT/PCR	6	1,376	0.7–50.0	5.6	6.9	0.6–18.0	60.7	p < 0.001	91.8	84.8–95.5	0.0–59.4
	Culture	1^e^	61	NA	NA	0.0	0.0–2.8	NA		NA		NA
	**Overall**	**7**	**1,437**	**0.0–50.0**	**4.5**	**5.3 **	**0.3–14.4**	**63.1 **	**p < 0.001**	**90.5 **	**83.0–94.7**	**0.0–49.4**
Unspecified/mixed anatomical site	NAAT/PCR	4	5,182	0.0–16.0	1.8	3.4	0.0–11.5	141.9	p < 0.001	97.9	96.5–98.7	0.0–65.7
	Culture	4	209	2.2–16.7	4.4	5.3	1.0–11.8	7.4	p = 0.061	59.2	0.0–86.4	0.0–41.3
	Other^d^	5	90,865	0.1–22.4	2.1	4.1	0.0–14.0	724.6	p < 0.001	99.4	99.3–99.6	0.0–61.8
	**Overall**	**13**	**96,256**	**0.0–22.4**	**2.3**	**4.1 **	**1.2–8.4**	**1,088.3 **	**p < 0.001**	**98.9 **	**98.6–99.1**	**0.0–28.1**
Sera	Blood tested for antibodies	3	504	12.9–34.0	16.5	18.8	9.4–30.3	7.8	p = 0.020	74.3	14.5–92.3	0.0–100.0

CI: confidence interval; NA: not applicable; NAAT: nucleic acid amplification test; NG: *Neisseria gonorrhoeae*.

The main results have been bolded to emphasise them and to align them with the corresponding discussions in the results section.

^a ^Q: the Cochran’s Q statistic is a measure assessing the existence of heterogeneity in pooled outcome measures, here NG prevalence.

^b ^I^2^: a measure that assesses the magnitude of between-study variation that is due to actual differences in NG prevalence across studies rather than chance.

^c ^Prediction interval: a measure that estimates the distribution (95% interval) of true NG prevalence around the estimated mean.

^d ^Other assays include unclear testing technique, enzyme immunoassay, complement fixation or mixed testing techniques.

^e ^No meta-analysis was done due to the small number of studies (n < 3).

^f ^Other populations include populations with an undetermined risk of acquiring *Neisseria gonorrhoeae* infection such as patients with cervical cancer, victims of sexual assault, specimens from virology/bacteriology laboratory and requesting home-based *N. gonorrhoeae or Chlamydia trachomatis* testing.

**Table 2:** Pooled estimates for *Neisseria gonorrhoeae* prevalence in populations at high-risk of infection, HIV-positive individuals and sexually transmitted infection clinic attendees in World Health Organization European Region countries, 1949–2021

Population type		Outcome measures	Sample size	NG prevalence (%)		Pooled NG prevalence		Heterogeneity measures				
		Total n	Total n	Range	Median	Mean (%)	95% CI	Cochran’s Q statistic^a^		I^2b^		Prediction interval^c^ (%)
								Q	p-value	I^2^ (%)	95% CI	
**FSWs**												
Current urogenital infection	NAAT/PCR	17	4,329	0.0–66.7	2.9	1.9	0.7–3.5	79.6	p < 0.001	79.9	68.6–87.1	0.0–10.1
	Culture	7	2,735	0.0–14.0	5.8	4.3	1.4–8.4	27.9	p < 0.001	78.5	55.7–89.6	0.0–21.7
	Gram staining	2^e^	253	0.0–10.2	5.1	3.0	0.0–20.4	NA		NA		NA
	Other^d^	5	2,704	1.0–16.2	6.2	6.0	1.6–12.4	90.3	p < 0.001	95.6	92.2–97.5	0.0–38.2
	**Overall**	**31**	**10,021**	**0.0–66.7**	**3.6**	**3.2 **	**1.8–4.8**	**318.5 **	**p < 0.001**	**90.6 **	**87.7–92.8**	**0.0–14.8**
Current anorectal infection	NAAT/PCR	3	2,091	1.4–1.8	1.4	1.5	1.0–2.0	0.5	p = 0.798	0.0	0.0–89.6	0.0–6.7
	Culture	1^e^	299	NA	NA	0.0	0.0–0.6	NA		NA		NA
	Other^d^	1^e^	50	NA	NA	12.0	4.2–22.7	NA		NA		NA
	**Overall**	**5**	**2,440**	**0.0–12.0**	**1.4**	**1.7 **	**0.1–5.0**	**22.6 **	**p < 0.001**	**82.3 **	**59.4–92.3**	**0.0–21.2**
Current oropharyngeal infection	NAAT/PCR	5	2,600	0.5–9.0	1.5	2.4	0.5–5.5	41.5	p < 0.001	90.4	80.4–95.3	0.0–19.2
	Culture	1^e^	299	NA	NA	0.0	0.0–0.6	NA		NA		NA
	Other^d^	1^e^	50	NA	NA	14.0	5.6–25.2	NA		NA		NA
	**Overall**	7	2,949	0.0–14.0	1.5	2.6	0.4–6.3	69.7	p < 0.001	91.4	84.8–95.1	0.0–21.7
Unspecified/mixed anatomical site	Other^d^	15	19,629	1.1–36.3	4.0	6.5	2.9–11.2	1,022.6	p < 0.001	98.6	98.3–98.9	0.0–34.3
	**Overall**	**15**	**19,629**	**1.1–36.3**	**4.0**	**6.5 **	**2.9–11.2**	**1,022.6 **	**p < 0.001**	**98.6 **	**98.3–98.9**	**0.0–34.3**
**MSM and MSWs**												
Current urogenital infection	NAAT/PCR	14	4,564	0.0–4.0	1.2	0.9	0.4–1.4	21.7	p = 0.061	40.0	0.0–68.1	0.0–2.5
	Other^d^	1^e^	1,832	NA	NA	1.5	1.0–2.2	NA		NA		NA
	**Overall**	**15**	**6,396**	**0.0–4.0**	**1.3**	**0.9 **	**0.5–1.4**	**22.5 **	**p = 0.070**	**37.7 **	**0.0–66.3**	**0.1–2.3**
Current anorectal infection	NAAT/PCR	11	6,095	3.4–14.0	4.6	5.8	4.1–7.7	53.5	p < 0.001	81.3	67.6–89.2	1.0–13.8
	Other^d^	3	3,758	0.4–15.4	4.5	5.0	0.1–16.5	168.7	p < 0.001	98.8	98.0–99.3	0.0–100.0
	**Overall**	**14**	**9,853**	**0.4–15.4**	**4.5**	**5.6 **	**3.6–8.1**	**257.0 **	**p < 0.001**	**94.9 **	**93.0–96.4**	**0.1–18.0**
Current oropharyngeal infection	NAAT/PCR	12	6,548	0.0–14.2	5.4	5.2	3.6–7.1	52.9	p < 0.001	79.2	64.4–87.9	0.7–12.9
	Culture	1^e^	239	NA	NA	2.5	0.8–5.0	NA		NA		NA
	Other^d^	5	4,568	0.5–4.9	1.3	1.9	0.7–3.7	82.4	p < 0.001	95.1	91.3–97.3	0.0–11.6
	**Overall**	**18**	**11,355**	**0.0–14.2**	**4.0**	**3.8 **	**2.5–5.4**	**231.3 **	**p < 0.001**	**92.6 **	**89.8–94.7**	**0.0–12.3**
Unspecified/mixed anatomical site	NAAT/PCR	14	15,840	0.0–69.6	10.7	11.7	5.7–19.4	345.5	p < 0.001	96.2	94.9–97.2	0.0–50.8
	Other^d^	1^e^	1,235	10.0–42.9	26.5	24.4	1.6–61.7	NA		NA		NA
	**Overall**	**16**	**17,075**	**0.0–69.6**	**10.7**	**13.2 **	**7.0–20.8**	**484.0 **	**p < 0.001**	**96.9 **	**96.0–97.6**	**0.0–53.7**
**Transgender people and transgender sex workers**												
Current urogenital infection	NAAT/PCR	1^e^	40	NA	NA	0.0	0.0–4.3	NA		NA		NA
	**Overall**	**1^e^**	**40**	NA	NA	**0.0 **	**0.0–4.3**	NA		NA		NA
Current anorectal infection	NAAT/PCR	1^e^	40	NA	NA	0.0	0.0–4.3	NA		NA		NA
	**Overall**	**1^e^**	**40**	NA	NA	**0.0 **	**0.0–4.3**	NA		NA		NA
Current oropharyngeal infection	NAAT/PCR	1^e^	40	NA	NA	2.5	0.0–10.4	NA		NA		NA
	**Overall**	**1^e^**	**40**	NA	NA	**2.5 **	**0.0–10.4**	NA		NA		NA
Unspecified/mixed anatomical site	NAAT/PCR	1^e^	14	NA	NA	0.0	0.0–11.9	NA		NA		NA
	**Overall**	**1^e^**	**14**	NA	NA	**0.0 **	**0.0–11.9**	NA		NA		NA
**HIV-positive individuals and individuals in HIV-discordant couples**												
Current urogenital infection	NAAT/PCR	15	3,753	0.0–2.0	0.0	0.3	0.0–0.7	28.4	p = 0.013	50.7	10.9–72.8	0.0–2.0
	Culture	1^e^	85	NA	NA	0.0	0.0–2.0	NA		NA		NA
	Other^d^	3	1,231	0.0–4.2	3.2	1.8	0.0–6.0	29.6	p < 0.001	93.2	83.6–97.2	0.0–99.9
	**Overall**	**19**	**5,069**	**0.0–4.2**	**0.0**	**0.5 **	**0.1–1.0**	**74.3 **	**p < 0.001**	**75.8 **	**62.3–84.4**	**0.0–3.6**
Current anorectal infection	NAAT/PCR	16	3,761	0.0–21.4	2.7	3.4	2.1–4.9	61.7	p < 0.001	75.7	60.5–85.0	0.0–10.3
	Other^d^	5	911	0.0–26.6	22.0	10.5	0.8–27.4	158.1	p < 0.001	97.5	95.9–98.4	0.0–85.1
	**Overall**	**21**	**4,672**	**0.0–26.6**	**3.0**	**4.5 **	**2.2–7.4**	**221.4 **	**p < 0.001**	**91.0 **	**87.6–93.4**	**0.0–23.0**
Current oropharyngeal infection	NAAT/PCR	12	2,907	0.0–8.0	2.3	2.4	1.7–3.1	16.6	p = 0.120	33.7	0.0–66.6	1.2–4.0
	Culture	1^e^	264	NA	NA	9.5	6.2–13.3	NA		NA		NA
	Other^d^	1^e^	339	NA	NA	0.0	0.0–0.5	NA		NA		NA
	**Overall**	**14**	**3,510**	**0.0–9.5**	**2.3**	**2.3 **	**1.1–3.8**	**66.5 **	**p < 0.001**	**80.5 **	**68.1–88.0**	**0.0–9.4**
Unspecified/mixed anatomical site	NAAT/PCR	1^e^	174	NA	NA	11.5	7.1–16.7	NA		NA		NA
	Culture	4	1,353	1.4–3.6	2.6	2.1	1.0–3.4	4.6	p = 0.201	35.1	0.0–77.4	0.0–8.2
	Other^d^	23	19,819	0.0–65.0	11.3	11.2	5.1–19.1	3,225.1	p < 0.001	99.3	99.2–99.4	0.0–62.3
	**Overall**	**28**	**21,346**	**0.0–65.0**	**7.3**	**9.5 **	**4.7–15.8**	**3,256.9 **	**p < 0.001**	**99.2 **	**99.1–99.3**	**0.0–55.0**
Sera	Blood tested for antibodies	1^e^	24	NA	NA	4.2	0.0–17.0	NA		NA		NA
**STI clinic attendees**												
Current urogenital infection	NAAT/PCR	154	1,125,284	0.0–36.4	2.1	2.7	2.2–3.3	11,248.8	p < 0.001	98.6	98.6–98.7	0.0–13.5
	Culture	154	545,153	0.0–63.6	5.1	6.8	5.5–8.2	17,349.6	p < 0.001	99.1	99.1–99.2	0.0–30.8
	Gram staining	16	241,508	0.1–32.5	12.5	10.9	5.5–17.9	9,370.6	p < 0.001	99.8	99.8–99.9	0.0–49.7
	Other^d^	50	50,461	0.0–32.0	4.6	5.4	3.7–7.4	1,826.3	p < 0.001	97.3	96.9–97.7	0.0–24.5
	**Overall**	**374**	**1,962,406**	**0.0–63.6**	**3.7**	**4.9 **	**4.2–5.6**	**53,523.5 **	**p < 0.001**	**99.3 **	**99.3–99.3**	**0.0–24.4**
Current anorectal infection	NAAT/PCR	59	576,033	0.0–36.9	4.2	4.2	2.9–5.6	14,357.1	p < 0.001	99.6	99.6–99.6	0.0–19.5
	Culture	48	120,628	0.0–29.2	4.2	4.4	3.1–5.8	4,137.9	p < 0.001	98.9	98.7–99.0	0.0–17.2
	Gram staining	3	9,462	5.2–10.1	6.6	6.4	5.9–7.0	3.5	p = 0.178	42.0	0.0–82.4	3.1–10.8
	Other^d^	27	33,419	1.4–22.7	5.2	6.2	4.6–8.1	628.8	p < 0.001	95.9	94.8–96.7	0.3–18.2
	**Overall**	**137**	**739,542**	**0.0–36.9**	**4.4**	**4.7 **	**3.9–5.5**	**22,200.9 **	**(p < 0.001)**	**99.4 **	**99.4–99.4**	**0.0–18.1**
Current oropharyngeal infection	NAAT/PCR	54	345,007	0.0–90.6	4.3	6.0	3.6–8.9	30,245.8	p < 0.001	99.8	99.8–99.8	0.0–37.4
	Culture	35	28,685	0.0–13.2	1.9	2.2	1.3–3.3	547.6	p < 0.001	93.8	92.3–95.0	0.0–10.4
	Gram staining	3	2,544	1.6–3.4	1.7	1.6	1.1–2.2	1.9	p = 0.390	0.0	0.0–89.6	0.0–6.7
	Other^d^	15	18,135	0.7–20.1	5.4	6.8	4.3–9.7	244.5	p < 0.001	94.3	92.0–95.9	0.1–21.6
	**Overall**	**107**	**394,371**	**0.0–90.6**	**3.6**	**4.7 **	**3.4–6.1**	**32,161.7 **	**p < 0.001**	**99.7 **	**99.7–99.7**	**0.0–26.7**
Unspecified/mixed anatomical site	NAAT/PCR	67	551,158	0.0–27.2	2.1	3.2	2.4–4.1	8,803.5	p < 0.001	99.3	99.2–99.3	0.0–13.8
	Culture	182	3,062,427	0.0–56.8	7.0	8.5	7.1–10.0	67,565.5	p < 0.001	99.7	99.7–99.7	0.0–36.0
	Gram staining	1^e^	3,179	NA	NA	7.0	6.1–7.9	NA		NA		NA
	Other^d^	163	4,859,245	0.0–81.3	4.0	7.7	6.2–9.4	84,540.2	p < 0.001	99.8	99.8–99.8	0.0–36.9
	**Overall**	**413**	**8,476,009**	**0.0–81.3**	**4.8**	**7.2 **	**6.3–8.1**	**230,664.4 **	**p < 0.001**	**99.8 **	**99.8–99.8**	**0.0–33.2**
Sera	Blood tested for antibodies	4	989	25.8–79.2	28.6	40.3	16.7–66.6	136.0	p < 0.001	97.8	96.3–98.7	0.0–100.0

CI: confidence interval; FSWs: female sex workers; MSM: men who have sex with men; MSWs: male sex workers; NA: not applicable; NAAT: nucleic acid amplification test; NG: *Neisseria gonorrhoeae*; STI: sexually transmitted infection.

The main results have been bolded to emphasize them and to align them with the corresponding discussions in the results section.

^a ^Q: the Cochran’s Q statistic is a measure assessing the existence of heterogeneity in pooled outcome measures, here NG prevalence.

^b ^I^2^: a measure that assesses the magnitude of between-study variation that is due to actual differences in NG prevalence across studies rather than chance.

^c ^Prediction interval: a measure that estimates the distribution (95% interval) of true NG prevalence around the estimated mean.

^d ^Other assays include unclear testing technique, enzyme immunoassay, complement fixation or mixed testing techniques.

^e ^No meta-analysis was done due to the small number of studies (n < 3).

**Table 3:** Pooled estimates for *Neisseria gonorrhoeae* prevalence in symptomatic populations, sexual contacts of persons infected with *Neisseria gonorrhoeae* or *Chlamydia trachomatis* and patients with confirmed or suspected sexually transmitted infections and related infections in World Health Organization European Region countries, 1949–2021

Population type		Outcome measures	Sample size	NG prevalence (%)		Pooled NG prevalence			Heterogeneity measures				
		Total n	Total n	Range	Median	Mean (%)		95% CI	Cochran’s Q statistic^a^		I^2b^		Prediction interval^c^ (%)
									Q	p-value	I^2^ (%)	95% CI	
**Symptomatic women**													
Current urogenital infection	NAAT/PCR	17	10,128	0.0–16.5	1.1	1.1	0.3–2.2		115.4	p < 0.001	86.1	79.3–90.7	0.0–7.0
	Culture	74	16,617	0.0–44.4	5.6	7.1	4.9–9.6		1,935.8	p < 0.001	96.2	95.7–96.7	0.0–37.0
	Gram staining	4	865	0.0–10.1	3.1	2.9	0.0–9.3		65.9	p < 0.001	95.4	91.2–97.6	0.0–52.3
	Other^d^	8	875	0.0–52.9	6.3	7.4	0.6–19.1		101.6	p < 0.001	93.1	88.7–95.8	0.0–60.2
	**Overall**	**103**	**28,485**	**0.0–52.9**	**2.5**	**5.6 **	**4.0–7.4**		**2,566.4 **	**p < 0.001**	**96.0 **	**95.6–96.4**	**0.0–31.9**
Current anorectal infection	NAAT/PCR	1^e^	50	NA	NA	14.0	5.6–25.2		NA		NA		NA
	Culture	3	3,368	0.2–1.5	1.3	0.9	0.2–2.0		16.4	p = 0.003	87.8	65.8–95.7	0.0–40.9
	Gram staining	1^e^	395	NA	NA	0.3	0.0–1.1		NA		NA		NA
	**Overall**	**5**	**3,813**	**1.2–14.0**	**1.3**	**1.6 **	**0.0–5.5**		**36.6 **	**p < 0.001**	**89.1 **	**77.2–94.8**	**0.0–25.9**
Current oropharyngeal infection	NAAT/PCR	1^e^	50	NA	NA	8.0	1.8–17.4		NA		NA		NA
	**Overall**	**1^e^**	**50**	NA	NA	**8.0 **	**1.8–17.4**		NA		NA		NA
Unspecified/mixed anatomical site	NAAT/PCR	1^e^	1,457	NA	NA	1.0	0.6–1.6		NA		NA		NA
	Culture	41	33,686	0.0–44.6	15.9	14.2	10.7–18.1		1,341.5	p < 0.001	97.0	96.5–97.5	0.0–44.2
	Gram staining	1^e^	438	NA	NA	0.0	0.0–0.4		NA		NA		NA
	Other^d^	13	4,015	1.0–52.2	22.7	19.6	10.7–30.5		475.6	p < 0.001	97.5	96.7–98.1	0.0–68.7
	**Overall**	**56**	**39,596**	**0.0–52.2**	**15.9**	**14.6 **	**11.1–18.4**		**2,519.0 **	**p < 0.001**	**97.8 **	**97.5–98.1**	**0.0–49.4**
Sera	Blood tested for antibodies	6	726	17.3–32.8	21.4	22.5	17.9–27.4		12.1	p = 0.033	58.7	0.0–83.3	10.2–37.8
**Symptomatic men**													
Current urogenital infection	NAAT/PCR	18	7,288	0.0–49.0	8.9	11.9	5.9–19.5		326.8	p < 0.001	94.8	93.0–96.1	0.0–53.0
	Culture	31	12,784	1.3–51.8	8.9	12.8	8.5–17.8		950.8	p < 0.001	96.8	96.2–97.4	0.0–47.9
	Gram staining	4	908	0.6–61.7	24.9	23.5	2.9–55.2		188.9	p < 0.001	98.4	97.5–99.0	0.0–100.0
	Other^d^	8	3,904	1.0–30.0	4.0	5.7	1.6–12.0		104.6	p < 0.001	93.3	89.1–95.9	0.0–35.4
	**Overall**	**61**	**24,884**	**0.0–61.7**	**8.9**	**12.1 **	**8.8–15.8**		**1,906.6 **	**p < 0.001**	**96.9 **	**96.4–97.2**	**0.0–48.6**
Current anorectal infection	NAAT/PCR	4	616	14.5–31.3	25.1	23.3	18.8–28.1		4.3	p = 0.234	29.7	0.0–74.4	10.6–39.0
	Culture	8	4,716	0.5–22.0	8.8	7.0	2.0–14.3		323.2	p < 0.001	97.8	96.9–98.5	0.0–41.0
	Other^d^	1^e^	3,066	NA	NA	0.3	0.1–0.5		NA		NA		NA
	**Overall**	**13**	**8,398**	**0.3–31.3**	**12.0**	**9.8 **	**4.3–17.1**		**771.3 **	**p < 0.001**	**98.4 **	**98.0–98.8**	**0.0–46.5**
Unspecified/mixed anatomical site	NAAT/PCR	16	1,127	3.6–45.6	27.6	25.8	19.7–32.3		83.5	p < 0.001	82.0	71.9–88.5	5.3–54.0
	Culture	3	330	2.4–12.0	7.2	6.9	2.5–13.0		6.6	p = 0.037	69.7	0.0–91.2	0.0–98.8
	Other^d^	7	1,205	0.0–50.0	16.6	14.9	3.2–32.3		240.3	p < 0.001	97.5	96.3–98.3	0.0–83.1
	**Overall**	**26**	**2,662**	**0.0–50.0**	**23.3**	**20.1 **	**14.2–26.7**		**411.3 **	**p < 0.001**	**93.9 **	**92.2–95.3**	**0.0–58.4**
**Symptomatic mixed sexes**													
Unspecified/mixed anatomical site	NAAT/PCR	1^e^	1,168	NA	NA	5.2	4.0–6.6		NA		NA		NA
	Other^d^	1^e^	1,055	NA	NA	9.2	7.5–11.0		NA		NA		NA
	**Overall**	**2^e^**	**2,223**	**0.0–87.0**	**4.2**	**7.1 **	**3.7–11.5**		NA		NA		NA
**Sexual contacts of persons infected with NG/CT**													
Current urogenital infection	NAAT/PCR	5	5,586	1.0–46.7	4.9	9.8	0.6–27.7		233.8	p < 0.001	98.3	97.4–98.9	0.0–89.7
	Culture	5	789	11.1–51.3	17.2	22.8	10.5–38.0		90.4	p < 0.001	95.6	92.2–97.5	0.0–82.6
	**Overall**	**10**	**6,375**	**1.0–51.3**	**14.7**	**15.7 **	**6.5–27.8**		**648.4 **	**p < 0.001**	**98.6 **	**98.2–98.9**	**0.0–68.7**
Current anorectal infection	NAAT/PCR	3	433	0.7–25.7	23.4	13.5	0.8–37.0		68.9	p < 0.001	97.1	94.2–98.5	0.0–100.0
	Culture	2^e^	34	17.7–23.5	20.6	20.5	8.0–36.4		NA		NA		NA
	**Overall**	**5**	**467**	**0.7–25.7**	**23.4**	**15.4 **	**4.5–30.7**		**70.5 **	**p < 0.001**	**94.3 **	**89.6–96.9**	**0.0–77.8**
Current oropharyngeal infection	NAAT/PCR	3	433	4.2–36.5	21.3	18.5	3.6–41.0		49.4	p < 0.001	96.0	91.3–98.1	0.0–100.0
	**Overall**	**3**	**433**	**4.2–36.5**	**21.3**	**18.5 **	**3.6–41.0**		**49.4 **	**p < 0.001**	**96.0 **	**91.3–98.1**	**0.0–100.0**
Unspecified/mixed anatomical site	Culture	10	1,360	10.8–87.0	42.9	43.7	26.0–62.3		418.8	p < 0.001	97.9	97.1–98.4	0.0–98.7
	Other^d^	14	25,331	1.4–84.5	70.6	52.5	33.9–70.7		3,246.9	p < 0.001	99.6	99.5–99.7	0.0–100.0
	**Overall**	**24**	**26,691**	**1.4–87.0**	**59.5**	**48.9 **	**35.7–62.1**		**3,680.3 **	**p < 0.001**	**99.4 **	**99.3–99.4**	**0.3–99.3**
**Patients with confirmed/suspected STIs and related infections**													
Current urogenital infection	NAAT/PCR	10	13,001	0.5–22.5	2.7	3.6	1.3–7.0		156.3	p < 0.001	94.2	91.3–96.2	0.0–20.7
	Culture	22	11,493	1.0–28.7	8.6	9.2	6.5–12.2		608.5	p < 0.001	96.5	95.6–97.3	0.3–27.3
	Other^d^	4	6,222	0.0–32.7	10.1	9.9	0.7–27.7		384.7	p < 0.001	99.2	98.9–99.5	0.0–99.0
	**Overall**	**36**	**30,716**	**0.0–32.7**	**7.0**	**7.5 **	**5.2–10.2**		**2,555.2**	**p < 0.001**	**98.6 **	**98.4–98.8**	**0.0–28.4**
Current anorectal infection	NAAT/PCR	13	2,458	0.0–50.0	15.9	16.8	9.3–25.7		73.1	p < 0.001	83.6	73.3–89.9	0.0–54.4
	Culture	1^e^	32	NA	NA	3.1	0.0–12.9		NA		NA		NA
	Other^d^	2^e^	115	4.2–15.4	9.8	10.7	2.6–22.6		NA		NA		NA
	**Overall**	**16**	**2,605**	**0.0–50.0**	**14.8**	**14.7 **	**8.6–21.9**		**78.9 **	**p < 0.001**	**81.0 **	**70.1–87.9**	**0.0–47.8**
Current oropharyngeal infection	NAAT/PCR	4	387	0.0–10.1	5.0	4.9	1.2–10.5		8.9	p = 0.031	66.3	1.3–88.5	0.0–38.2
	Culture	1^e^	32	NA	NA	3.1	0.0–12.9		NA		NA		NA
	**Overall**	**5**	**419**	**0.0–10.1**	**3.0**	**4.7 **	**1.5–9.2**		**9.3 **	**p = 0.054**	**57.0 **	**0.0–84.1**	**0.0–22.9**
Unspecified/mixed anatomical site	NAAT/PCR	6	1,461	2.0–29.0	13.3	10.6	3.9–19.8		78.2	p < 0.001	93.6	88.7–96.4	0.0–50.1
	Culture	4	2,506	11.5–52.4	37.6	33.5	16.9–52.4		192.4	p < 0.001	98.4	97.5–99.0	0.0–100.0
	Gram staining	3	692	7.8–33.2	24.6	21.4	8.7–37.7		20.7	p < 0.001	90.3	74.4–96.3	0.0–100.0
	Other^d^	7	2,744	7.1–50.0	19.5	19.9	10.4–31.6		277.2	p < 0.001	97.8	96.9–98.5	0.0–66.2
	**Overall**	**20**	**7,403**	**2.0–52.4**	**19.2**	**19.7 **	**13.5–26.8**		**806.5 **	**p < 0.001**	**97.6 **	**97.1–98.1**	**0.1–57.5**
Sera	Blood tested for antibodies	1^e^	43	NA	NA	23.3	11.7–37.2		NA		NA		NA

CI: confidence interval; CT: *Chlamydia trachomatis*; NA: not applicable; NAAT: nucleic acid amplification test; NG: *Neisseria gonorrhoeae*; STIs: sexually transmitted infections.

The main results have been bolded to emphasise them and to align them with the corresponding discussions in the results section.

^a ^Q: the Cochran’s Q statistic is a measure assessing the existence of heterogeneity in pooled outcome measures, here NG prevalence.

^b ^I^2^: a measure that assesses the magnitude of between-study variation that is due to actual differences in NG prevalence across studies rather than chance.

^c ^Prediction interval: a measure that estimates the distribution (95% interval) of true NG prevalence around the estimated mean.

^d ^Other assays include unclear testing technique, enzyme immunoassay, complement fixation or mixed testing techniques.

^e ^No meta-analysis was done due to the small number of studies (n < 3).

### Precision and risk of bias assessments

Results of the precision and ROB assessments are summarised in Supplementary Table S4. Among all studies (n = 1,573), 1,232 (78.3%) had high precision, 50 (3.2%) had low ROB in the sampling method domain and 93 (5.9%) had low ROB in the response rate domain. In contrast, 341 (21.7%) studies had low precision, 1,523 (96.8%) had high ROB in the sampling method domain and 92 (5.9%) had high ROB in the response rate domain. For 1,388 (88.2%) studies, the ROB assessment for the response rate domain was ‘unclear’. Only 6 (0.4%) studies had low ROB in both quality domains, whereas 75 (4.8%) studies had high ROB in both quality domains.

Notably, in the meta-regression analyses for NG prevalence (note below), no evidence was found for variation in prevalence by sampling method or response rate. However, there was evidence for a small-study effect with larger (high precision) studies reporting lower prevalence than smaller (low precision) studies.

### Pooled estimates for *Neisseria gonorrhoeae* prevalence

Pooled NG prevalence among the different population types stratified by anatomical site and assay type is listed in Tables 1–3. Among general populations for all assay types, pooled prevalence was 1.0% (95% CI: 0.7–1.2%) for urogenital infection, 2.3% (95% CI: 0.2–6.3%) for anorectal infection and 0.9% (95% CI: 0.0–4.6%) for oropharyngeal infection (Table 1).

Among female sex workers (FSWs), pooled prevalence was 3.2% (95% CI: 1.8–4.8%) for urogenital infection, 1.7% (95% CI: 0.1–5.0%) for anorectal infection and 2.6% (95% CI: 0.4–6.3%) for oropharyngeal infection (Table 2). Among men who have sex with men (MSM) and male sex workers (MSWs), pooled prevalence was 0.9% (95% CI: 0.5–1.4%) for urogenital infection, 5.6% (95% CI: 3.6–8.1%) for anorectal infection and 3.8% (95% CI: 2.5–5.4%) for oropharyngeal infection (Table 2). Among STI clinic attendees, pooled prevalence was 4.9% (95% CI: 4.2–5.6%) for urogenital infection, 4.7% (95% CI: 3.9–5.5%) for anorectal infection and 4.7% (95% CI: 3.4–6.1%) for oropharyngeal infection (Table 2).

Among symptomatic women, pooled prevalence was 5.6% (95% CI: 4.0–7.4%) for urogenital infection and 1.6% (95% CI: 0.0–5.5%) for anorectal infection (Table 3). Among symptomatic men, pooled prevalence was 12.1% (95% CI: 8.8–15.8%) for urogenital infection and 9.8% (95% CI: 4.3–17.1%) for anorectal infection (Table 3). Among sexual contacts of persons infected with NG or *Chlamydia trachomatis* (CT), pooled prevalence was 15.7% (95% CI: 6.5–27.8%) for urogenital infection, 15.4% (95% CI: 4.5–30.7%) for anorectal infection and 18.5% (95% CI: 3.6–41.0%) for oropharyngeal infection (Table 3).

Most meta-analyses showed strong evidence for heterogeneity (p value < 0.001) with most of the heterogeneity being attributed to true variation in prevalence across studies rather than sampling variation (I^2^ > 50%) (Tables 1–3). Heterogeneity was confirmed by the wide prediction intervals of the distribution of prevalence around the pooled means (Tables 1–3). Forest plots of prevalence of current urogenital infection across all populations are found in Supplementary Figure S1.

### Associations with *Neisseria gonorrhoeae* prevalence

Results of the univariable and multivariable meta-regression analyses of NG prevalence by anatomical site are shown in Table 4, Table 5 and Table 6. Two multivariable models were implemented for each anatomical site to account for the collinearity between the year of data collection as a categorical variable and the year of data collection as a linear term. In these multivariable analyses, the models considered explained more than 30% of the variation in prevalence (Tables 4–6).

**Table 4:** Univariable and multivariable meta-regression analyses for *Neisseria gonorrhoeae* prevalence in urogenital specimens in World Health Organization European Region countries, 1949–2021

Urogenital specimens		Outcome measures	Sample size	Univariable analysis					Multivariable analyses					
		Total n	Total n	RR	95% CI	p value	LT test p-value	Adjusted R^2^	Model 1^a^			Model 2^b^		
									ARR	95% CI	p value	ARR	95% CI	p value
**Population characteristics**														
**Population type**	General populations	262	644,913	1.00		NA	< 0.001	18.84	**1.00**		NA	**1.00**		NA
	Intermediate-risk populations	24	6,559	2.54	1.28–5.04	0.008		NA	**3.00 **	**1.59–5.68**	**0.001**	**2.47 **	**1.32–4.61**	**0.005**
	FSWs	31	10,021	3.28	1.96–5.47	< 0.001		NA	**4.04 **	**2.52–6.48**	** < 0.001**	**3.73 **	**2.35–5.92**	** < 0.001**
	MSM, MSWs, and transgender people	16	6,436	0.95	0.45–2.01	0.887		NA	**1.31 **	**0.64–2.65**	**0.459**	**1.36 **	**0.68–2.73**	**0.383**
	Infertility clinic attendees	51	7,150	2.58	1.38–4.80	0.003		NA	**1.79 **	**1.00–3.22**	**0.051**	**1.46 **	**0.81–2.61**	**0.204**
	Symptomatic women	103	28,485	4.11	2.97–5.69	< 0.001		NA	**3.27 **	**2.39–4.46**	** < 0.001**	**2.98 **	**2.20–4.05**	** < 0.001**
	Symptomatic men	61	24,884	7.19	4.99–10.30	< 0.001		NA	**5.53 **	**3.74–8.18**	** < 0.001**	**5.22 **	**3.57–7.64**	** < 0.001**
	STI clinic attendees	374	1,962,406	2.91	2.33–3.65	< 0.001		NA	**3.14 **	**2.51–3.93**	** < 0.001**	**2.98 **	**2.40–3.71**	** < 0.001**
	HIV-positive individuals and individuals in HIV discordant couples	19	5,069	0.85	0.35–2.04	0.714		NA	**1.02 **	**0.45–2.30**	**0.970**	**0.94 **	**0.42–2.09**	**0.873**
	Sexual contacts of persons infected with NG/CT	10	6,375	7.83	3.67–16.60	< 0.001		NA	**8.08 **	**4.08–16.00**	** < 0.001**	**7.03 **	**3.59–13.70**	** < 0.001**
	Patients with confirmed/suspected STIs and related infections	36	30,716	4.53	2.93–7.01	< 0.001		NA	**4.85 **	**3.25–7.25**	** < 0.001**	**4.59 **	**3.10–6.81**	** < 0.001**
	Other populations^c^	25	23,715	2.83	1.67–4.81	< 0.001		NA	**4.05 **	**2.50–6.55**	** < 0.001**	**3.75 **	**2.34–6.02**	** < 0.001**
**Age group**	< 20 years	50	11,635	1.00		NA	0.007	1.53	**1.00**		NA	**1.00**		NA
	20–29 years	34	16,002	0.74	0.39–1.41	0.359		NA	**0.83 **	**0.49–1.41**	**0.489**	**0.88 **	**0.52–1.47**	**0.617**
	30–39 years	20	5,386	0.99	0.43–2.26	0.979		NA	**1.26 **	**0.65–2.45**	**0.499**	**1.37 **	**0.71–2.63**	**0.351**
	≥ 40 years	18	4,012	0.82	0.37–1.85	0.639		NA	**1.69 **	**0.85–3.34**	**0.132**	**2.06 **	**1.05–4.04**	**0.035**
	Mixed ages	890	2,719,694	0.53	0.35–0.80	0.003		NA	**0.37 **	**0.26–0.52**	** < 0.001**	**0.42 **	**0.30–0.60**	** < 0.001**
**Sex**	Women	558	1,154,985	1.00		NA	< 0.001	2.40	**1.00**		NA	**1.00**		NA
	Men	371	933,280	1.62	1.34–1.98	< 0.001	NA	NA	**1.41 **	**1.16–1.73**	**0.001**	**1.45 **	**1.19–1.77**	** < 0.001**
	Mixed sexes	83	668,464	1.21	0.88–1.65	0.239	NA	NA	**1.28 **	**0.97–1.68**	**0.076**	**1.17 **	**0.90–1.53**	**0.248**
**European subregions**	Eastern Europe	77	281,396	1.00		NA	0.922	0.00	NA		NA	NA		NA
	Southern Europe	121	72,030	1.08	0.70–1.67	0.724	NA	NA	NA		NA	NA		NA
	Western Europe	330	1,242,348	0.96	0.66–1.38	0.816	NA	NA	NA		NA	NA		NA
	Northern Europe	424	1,136,092	1.04	0.73–1.50	0.815	NA	NA	NA		NA	NA		NA
	Israel, Türkiye and mixed regions	60	24,863	1.06	0.62–1.81	0.838	NA	NA	NA		NA	NA		NA
**Country’s income level**	LMIC	7	1,353	1.00		NA	0.021	1.01	**1.00**		NA	**1.00**		NA
	UMIC	76	279,505	2.16	0.68–6.89	0.193	NA	NA	**2.12 **	**0.78–5.79**	**0.143**	**1.49 **	**0.56–3.96**	**0.427**
	HIC	927	2,467,907	1.93	0.63–5.88	0.246	NA	NA	**1.46 **	**0.56–3.83**	**0.441**	**1.05 **	**0.41–2.71**	**0.918**
	Mixed income	2	7,964	0.14	0.02–1.17	0.069	NA	NA	**0.39 **	**0.07–2.33**	**0.303**	**0.26 **	**0.04–1.47**	**0.127**
Study methodology characteristics														
**Assay type**	NAAT/PCR	427	1,448,120	1.00		NA	< 0.001	6.31	**1.00**		NA	**1.00**		NA
	Culture	453	985,482	1.91	1.57–2.32	< 0.001	NA	NA	**0.98 **	**0.76–1.26**	**0.850**	**0.81 **	**0.64–1.03**	**0.087**
	Gram Staining	32	247,107	2.52	1.53–4.14	< 0.001	NA	NA	**1.31 **	**0.83–2.06**	**0.246**	**0.98 **	**0.62–1.53**	**0.919**
	Other/unclear	100	76,020	1.79	1.30–2.46	< 0.001	NA	NA	**0.94 **	**0.70–1.27**	**0.686**	**0.84 **	**0.63–1.13**	**0.250**
**Sample size** ^d^	< 200	189	14,814	1.00		NA	< 0.001	7.82	**1.00**		NA	**1.00**		NA
	≥ 200	823	2,741,915	0.38	0.29–0.49	< 0.001	NA	NA	**0.44 **	**0.34–0.56**	** < 0.001**	**0.43 **	**0.34–0.55**	** < 0.001**
**Sampling method**	Probability based	39	15,610	1.00		NA	0.106	0.00	NA		NA	NA		NA
	Non-probability based	973	2,741,119	1.73	0.89–3.34	0.106	NA	NA	NA		NA	NA		NA
**Response rate**	≥ 80%	62	44,933	1.00		NA	< 0.001	1.44	**1.00**		NA	**1.00**		NA
	< 80%	68	30,509	1.05	0.58–1.89	0.874	NA	NA	**0.83 **	**0.50–1.37**	**0.460**	**0.78 **	**0.48–1.28**	**0.334**
	Unclear	882	2,681,287	1.99	1.33–2.98	0.001	NA	NA	**1.35 **	**0.96–1.90**	**0.080**	**1.31 **	**0.94–1.84**	**0.110**
Temporal trend														
**Year of data collection category**	< 2000	455	939,407	1.00		NA	< 0.001	6.90	**1.00**		NA	NA		NA
	2000–2010	258	881,492	0.53	0.43–0.66	< 0.001	NA	NA	**0.46 **	**0.36–0.59**	** < 0.001**	NA		NA
	> 2010	299	935,830	0.49	0.39–0.61	< 0.001	NA	NA	**0.54 **	**0.41–0.71**	** < 0.001**	NA		NA
**Year of data collection**		1,012	2,756,729	0.97	0.97–0.98	< 0.001	< 0.001	11.08	NA		NA	**0.97 **	**0.96–0.98**	** < 0.001**

ARR*:* adjusted risk ratio; CI: confidence interval; CT: *Chlamydia trachomatis*; FSWs: female sex workers; HIC: high-income country; MSM: men who have sex with men, MSWs: male sex workers; NA: not applicable; NAAT: nucleic acid amplification test; NG: *Neisseria gonorrhoeae*; LMIC: low-middle income country; LT test: likelihood ratio test; RR*:* risk ratio; STI: sexually transmitted infection; UMIC: upper-middle income country.

The main results have been bolded to emphasize them and to align them with the corresponding discussions in the results section.

^a ^Adjusted R^2^ in the final multivariable model 1 = 37.43%.

^b ^Adjusted R^2^ in the final multivariable model 2 = 39.81%.

^c ^Other populations include populations with an undetermined risk of acquiring *Neisseria gonorrhoeae* infection such as patients with cervical cancer, victims of sexual assault, specimens from virology/bacteriology laboratory and requesting home-based *N. gonorrhoeae* or *Chlamydia trachomatis* testing.

^d ^Sample size denotes the sample size of each study population at the baseline found in the original publication.

**Table 5:** Univariable and multivariable meta-regression analyses for *Neisseria gonorrhoeae* prevalence in anorectal specimens in World Health Organization European Region countries, 1949–2021

Anorectal specimens		Outcome measures	Sample size	Univariable analysis					Multivariable analyses					
		Total n	Total n	RR	95% CI	p value	LT test p value	Adjusted R^2^	Model 1^a^			Model 2^b^		
									ARR	95% CI	p value	ARR	95% CI	p value
**Population characteristics**														
**Population type**	MSM, MSWs, and transgender people^c^	15	9,893	1.00		NA	< 0.001	12.87	**1.00**		NA	**1.00**		NA
	General populations	3	1,258	0.41	0.11–1.55	0.187	NA	NA	**1.12 **	**0.33–3.85**	**0.854**	**1.23 **	**0.35–4.27**	**0.744**
	Intermediate-risk populations	2	141	4.01	0.46–34.70	0.207	NA	NA	**10.10 **	**1.43–72.00**	**0.021**	**9.35 **	**1.31–66.60**	**0.026**
	FSWs	5	2,440	0.50	0.16–1.61	0.244	NA	NA	**1.18 **	**0.39–3.57**	**0.766**	**1.09 **	**0.36–3.31**	**0.877**
	Symptomatic women	5	3,813	0.27	0.09–0.81	0.020	NA	NA	**1.33 **	**0.39–4.46**	**0.647**	**1.21 **	**0.36–4.06**	**0.759**
	Symptomatic men	13	8,398	1.30	0.59–2.85	0.512	NA	NA	**1.13 **	**0.56–2.30**	**0.730**	**1.11 **	**0.54–2.25**	**0.782**
	STI clinic attendees	137	739,542	0.86	0.49–1.52	0.597	NA	NA	**1.27 **	**0.75–2.16**	**0.366**	**1.24 **	**0.73–2.09**	**0.427**
	HIV-positive individuals and individuals in HIV discordant couples	21	4,672	1.05	0.51–2.19	0.887	NA	NA	**0.90 **	**0.47–1.74**	**0.765**	**0.89 **	**0.46–1.72**	**0.731**
	Sexual contacts of persons infected with NG/CT	5	467	2.97	0.98–9.00	0.054	NA	NA	**3.45 **	**1.25–9.53**	**0.017**	**3.59 **	**1.29–9.95**	**0.014**
	Patients with confirmed/suspected STIs and related infections	16	2,605	3.13	1.44–6.78	0.004	NA	NA	**3.23 **	**1.60–6.50**	**0.001**	**3.20 **	**1.59–6.47**	**0.001**
	Other populations^d^	7	1,348	1.17	0.42–3.25	0.762	NA	NA	**2.21 **	**0.86–5.68**	**0.100**	**2.29 **	**0.89–5.91**	**0.087**
**Age group**	≤ 30 years	8	1,165	1.00		NA	0.559	0.00	NA		NA	NA		NA
	> 30 years	6	498	1.41	0.34–5.85	0.631	NA	NA	NA		NA	NA		NA
	Mixed ages	215	772,914	0.80	0.34–1.89	0.615	NA	NA	NA		NA	NA		NA
**Sex**	Women	52	176,849	1.00		NA	< 0.001	11.86	**1.00**		NA	**1.00**		NA
	Men	167	579,396	2.62	1.83–3.75	< 0.001	NA	NA	**2.86 **	**1.96–4.19**	** < 0.001**	**2.75**	**1.89–4.02**	** < 0.001**
	Mixed sexes	10	18,332	2.05	0.99–4.24	0.052	NA	NA	**2.05 **	**1.06–3.98**	**0.033**	**2.05 **	**1.06–3.97**	**0.034**
**European subregions**	Eastern Europe	6	2,132	1.00		NA	0.004	5.83	**1.00**		NA	**1.00**		NA
	Southern Europe	24	11,557	3.39	1.19–9.64	0.023	NA	NA	**0.71 **	**0.24–2.12**	**0.537**	**0.63 **	**0.21–1.89**	**0.408**
	Western Europe	120	633,385	1.58	0.61–4.14	0.347	NA	NA	**0.80 **	**0.30–2.14**	**0.650**	**0.70 **	**0.26–1.88**	**0.473**
	Northern Europe	78	127,487	2.25	0.85–5.97	0.101	NA	NA	**0.94 **	**0.34–2.62**	**0.910**	**0.85 **	**0.31–2.37**	**0.759**
	Israel, Türkiye and mixed regions	1	16	12.30	1.17–129.80	0.037	NA	NA	**2.10 **	**0.24–18.50**	**0.504**	**1.96 **	**0.22–17.50**	**0.545**
**Country’s income level**	UMIC	4	1,342	1.00		NA	0.784	0.00	NA		NA	NA		NA
	HIC	225	773,235	1.18	0.37–3.76	0.784	NA	NA	NA		NA	NA		NA
**Study methodology characteristics**														
**Assay type**	NAAT/PCR	121	593,512	1.00		NA	0.288	0.41	NA		NA	NA		NA
	Culture	65	129,889	0.74	0.52–1.06	0.098	NA	NA	NA		NA	NA		NA
	Gram staining	4	9,857	0.80	0.25–2.53	0.707	NA	NA	NA		NA	NA		NA
	Other/unclear	39	41,319	1.10	0.73–1.65	0.661	NA	NA	NA		NA	NA		NA
**Sample size** ^e^	< 200	38	2,715	1.00		NA	< 0.001	11.42	**1.00**		NA	**1.00**		NA
	≥ 200	191	771,862	0.38	0.26–0.56	< 0.001	NA	NA	**0.46 **	**0.31–0.67**	** < 0.001**	**0.45 **	**0.31–0.66**	** < 0.001**
**Sampling method**	Probability based	4	1,154	1.00		NA	0.927	0.00	NA		NA	NA		NA
	Non-probability based	225	773,423	0.95	0.32–2.85	0.927	NA	NA	NA		NA	NA		NA
**Response rate**	≥ 80%	7	22,936	1.00		NA	0.509	0.00	NA		NA	NA		NA
	< 80%	16	5,859	0.95	0.32–2.78	0.925	NA	NA	NA		NA	NA		NA
	Unclear	206	745,782	1.32	0.54–3.21	0.536	NA	NA	NA		NA	NA		NA
**Temporal trend**														
**Year of data collection category**	< 2000	49	130,000	1.00		NA	0.001	6.73	**1.00**		NA	NA		NA
	2000–2010	60	139,618	1.41	0.93–2.16	0.109	NA	NA	**1.60 **	**1.10–2.33**	**0.014**	NA		NA
	> 2010	120	504,959	2.02	1.40–2.91	< 0.001	NA	NA	**2.07 **	**1.45–2.96**	** < 0.001**	NA		NA
**Year of data collection**		229	774,577	1.02	1.01–1.03	0.001	0.001	5.60	NA		NA	**1.02 **	**1.01–1.04**	** < 0.001**

ARR: adjusted risk ratio; CI: confidence interval; CT: *Chlamydia trachomatis*; FSWs: female sex workers; HIC: high-income country; MSM: men who have sex with men; MSWs: male sex workers; NA: not applicable; NAAT: nucleic acid amplification test, NG: *Neisseria gonorrhoeae*; LT test: likelihood ratio test, *RR*: risk ratio; STI: sexually transmitted infection; UMIC: upper-middle income country.

The main results have been bolded to emphasise them and to align them with the corresponding discussions in the results section.

^a ^Adjusted R^2^ in the final multivariable model 1 = 35.94%.

^b ^Adjusted R^2^ in the final multivariable model 2 = 35.20%.

^c ^MSM, MSWs, and transgender people group was used as a reference because of epidemiological relevance and because the general populations group had small number of measures.

^d ^Other populations include populations with an undetermined risk of acquiring *Neisseria gonorrhoeae* infection such as patients with cervical cancer, victims of sexual assault, specimens from virology/bacteriology laboratory and requesting home-based *N. gonorrhoeae* or *Chlamydia trachomatis* testing.

^e ^Sample size denotes the sample size of each study population at the baseline found in the original publication.

**Table 6:** Univariable and multivariable meta-regression analyses for *Neisseria gonorrhoeae* prevalence in oropharyngeal specimens in World Health Organization European Region countries, 1949–2021

Oropharyngeal specimens		Outcome measures	Sample size	Univariable analysis					Multivariable analyses					
		Total n	Total n	*RR*	95% CI	p value	LT test p value	Adjusted R^2^	Model 1^a^			Model 2^b^		
									ARR	95% CI	p value	ARR	95% CI	p value
**Population characteristics**														
**Population type^c^**	MSM, MSWs, and transgender people^d^	19	11,395	1.00		NA	0.266	1.56	**1.00**		**NA**	**1.00**		**NA**
	General populations	3	475	1.08	0.14–8.34	0.936	NA	NA	**1.83 **	**0.29–11.60**	**0.518**	**1.63 **	**0.25–10.60**	**0.609**
	FSWs	7	2,949	0.84	0.31–2.28	0.738	NA	NA	**1.89 **	**0.74–4.78**	**0.180**	**1.67 **	**0.65–4.26**	**0.284**
	STI clinic attendees	107	394,371	1.08	0.63–1.82	0.772	NA	NA	**1.81 **	**1.12–2.92**	**0.016**	**1.60 **	**0.99–2.59**	**0.053**
	HIV-positive individuals and individuals in HIV discordant couples	14	3,510	0.87	0.39–1.95	0.752	NA	NA	**0.90 **	**0.45–1.82**	**0.776**	**0.79 **	**0.39–1.60**	**0.514**
	Sexual contacts of persons infected with NG/CT	3	433	4.37	1.26–15.10	0.020	NA	NA	**5.20 **	**1.82–14.90**	**0.002**	**5.08 **	**1.74–14.80**	**0.003**
	Patients with confirmed/suspected STIs and related infections	6	469	1.73	0.57–5.28	0.329	NA	NA	**3.00 **	**1.13–7.99**	**0.028**	**2.86 **	**1.06–7.71**	**0.038**
	Other populations^e^	8	1,460	1.76	0.70–4.45	0.224	NA	NA	**2.58 **	**1.12–5.93**	**0.026**	**2.69 **	**1.16–6.27**	**0.022**
**Age group**	≤ 30 years	6	664	1.00		NA	0.253	0.45	**NA**		**NA**	**NA**		**NA**
	> 30 years	4	462	0.83	0.16–4.37	0.832	NA	NA	**NA**		**NA**	**NA**		**NA**
	Mixed ages	157	413,936	0.50	0.20–1.22	0.129	NA	NA	NA		NA	NA		NA
**Sex**	Women	43	151,890	1.00		NA	0.020	5.66	**1.00**		NA	**1.00**		NA
	Men	116	241,147	1.70	1.15–2.50	0.007	NA	NA	**2.55 **	**1.72–3.78**	** < 0.001**	**2.64 **	**1.77–3.93**	** < 0.001**
	Mixed sexes	8	22,025	1.12	0.51–2.45	0.767	NA	NA	**1.30 **	**0.62–2.75**	**0.488**	**1.49 **	**0.70–3.16**	**0.297**
**European subregions**	Eastern Europe	3	1,198	1.00		- NA	0.979	0.00	NA		NA	NA		NA
	Southern Europe	19	8,217	1.36	0.35–5.30	0.648	NA	NA	NA		NA	NA		NA
	Western Europe	85	379,721	1.31	0.36–4.72	0.668	NA	NA	NA		NA	NA		NA
	Northern Europe	57	23,580	1.43	0.39–5.21	0.579	NA	NA	NA		NA	NA		NA
	Israel, Türkiye and mixed regions	3	2,346	1.25	0.22–7.05	0.793	NA	NA	NA		NA	NA		NA
**Country’s income level**	UMIC	3	1,198	1.00		NA	0.629	0.00	NA		NA	NA		NA
	HIC	164	413,864	1.35	0.38–4.76	0.629	NA	NA	NA		NA	NA		NA
**Study methodology characteristics**														
**Assay type**	NAAT/PCR	102	359,846	1.00		NA	0.029	4.14	**1.00**		NA	**1.00**		NA
	Culture	40	29,580	0.56	0.37–0.85	0.008	NA	NA	**0.60 **	**0.36–1.01**	**0.056**	**0.59 **	**0.34–1.02**	**0.060**
	Gram staining	3	2,544	0.44	0.13–1.46	0.181	NA	NA	**0.57 **	**0.19–1.66**	**0.299**	**0.49 **	**0.16–1.46**	**0.198**
	Other/unclear	22	23,092	1.05	0.65–1.71	0.822	NA	NA	**0.71 **	**0.45–1.11**	**0.134**	**0.71 **	**0.45–1.13**	**0.145**
**Sample size** ^f^	< 200	18	1,557	1.00		NA	0.011	5.10	**1.00**		NA	**1.00**		NA
	≥ 200	149	413,505	0.48	0.27–0.84	0.011	NA	NA	**0.42 **	**0.25–0.71**	**0.001**	**0.40 **	**0.24–0.68**	**0.001**
**Sampling method**	Probability based	4	1,118	1.00		NA	0.505	0.00	NA		NA	NA		NA
	Non-probability based	163	413,944	1.43	0.49–4.20	0.505	NA	NA	NA		NA	NA		NA
**Response rate**	≥ 80%	9	3,826	1.00		NA	0.156	1.10	NA		NA	NA		NA
	< 80%	11	3,909	0.36	0.12–1.01	0.054	NA	NA	NA		NA	NA		NA
	Unclear	147	407,327	0.59	0.27–1.29	0.192	NA	NA	NA		NA	NA		NA
**Temporal trend**														
**Year of data collection category**	< 2000	26	9,190	1.00		NA	< 0.001	11.74	**1.00**		NA	NA		NA
	2000–2010	49	161,036	1.23	0.74–2.07	0.423	NA	NA	**1.15 **	**0.65–2.06**	**0.626**	NA		NA
	> 2010	92	244,836	2.33	1.45–3.73	< 0.001	NA	NA	**1.92 **	**1.03–3.57**	**0.040**	NA		NA
**Year of data collection**		167	415,062	1.02	1.01–1.04	0.001	0.001	7.85	NA		NA	**1.02 **	**1.00–1.04**	**0.097**

ARR: adjusted risk ratio; CI: confidence interval; CT: *Chlamydia trachomatis*; FSWs: female sex workers; HIC: high-income country; MSM: men who have sex with men; MSWs: male sex workers; NA: not applicable; NAAT: nucleic acid amplification test; NG: *Neisseria gonorrhoeae*; LT test: likelihood ratio test; RR: risk ratio; STI: sexually transmitted infection; UMIC: upper-middle income country.

The main results have been bolded to emphasise them and to align them with the corresponding discussions in the results section.

^a ^Adjusted R^2^ in the final multivariable model 1 = 33.15%.

^b ^Adjusted R^2^ in the final multivariable model 2 = 30.43%.

^c ^Population classification was included in the multivariable analyses for epidemiological relevance.

^d ^MSM, MSWs, and transgender people group was used as a reference because of epidemiological relevance and because the general populations group had small number of measures.

^e ^Other populations include populations with an undetermined risk of acquiring *Neisseria gonorrhoeae* infection such as patients with cervical cancer, victims of sexual assault, specimens from virology/bacteriology laboratory and requesting home-based *N. gonorrhoeae* or *Chlamydia trachomatis* testing.

^f ^Sample size denotes the sample size of each study population at the baseline found in the original publication.

#### Urogenital *Neisseria gonorrhoeae* infection

Compared with general populations, prevalence was highest among sexual contacts of persons infected with NG or CT, followed by symptomatic men, patients with confirmed or suspected STIs, FSWs, symptomatic women, STI clinical attendees and intermediate risk populations (Table 4). Compared with women, men had 1.45-fold (95% CI: 1.19–1.77) higher prevalence (Table 4). Prevalence declined by 0.97-fold (95% CI: 0.96–0.98) per year, that is a 3% decline per year (Table 4).

#### Anorectal *Neisseria gonorrhoeae* infection

Compared with MSM, MSWs, and transgender people, prevalence was also highest among sexual contacts of persons infected with NG or CT, but otherwise differences in prevalence were not statistically significant, or significant but with relatively wide 95% CIs (Table 5). Compared with women, men had 2.75-fold (95% CI: 1.89–4.02) higher prevalence (Table 5). Prevalence increased by 1.02-fold (95% CI: 1.01–1.04) per year, that is a 2% increase per year (Table 5). 

#### Oropharyngeal *Neisseria gonorrhoeae* infection

Compared with MSM, MSWs and transgender people, prevalence was also highest among sexual contacts of persons infected with NG or CT, but otherwise differences in prevalence were not statistically significant, or significant but with relatively wide 95% CIs (Table 6). Compared with women, men had 2.64-fold (95% CI: 1.77–3.93) higher prevalence (Table 6). Prevalence increased by 1.02-fold (95% CI: 1.00–1.04) per year, that is a 2% increase per year, with this increase being of borderline statistical significance (Table 6).

#### Other results for all anatomical sites

There was no evidence for differences in prevalence by age group, European subregion, or country income level for all analyses across the anatomical sites (Tables 4–6). Regarding the effects of study methods on prevalence, no statistically significant differences in prevalence were found based on assay type, sampling method or response rate in all analyses across the anatomical sites (Tables 4–6). However, there was evidence for a small-study effect with studies including a sample size ≥ 200 reporting > 50% lower prevalence in all analyses across the anatomical sites.

### Sensitivity analyses to confirm the findings

The sensitivity analyses performed to validate the findings from the main analysis showed similar results when using the year of publication vs year of data collection in the models, as shown in Supplementary Tables S5-S7.

The sensitivity analyses performed to examine whether the results differed based on different diagnostic methods yielded results consistent with those observed in the main analysis (not shown). However, due to the smaller number of included studies in each subanalysis, some effect sizes had wider 95% CIs, leading to non-significant effects for some outcomes.

The cumulative meta-analyses, using the year of publication as the ordering variable, supported the observed trends in NG prevalence generated by the meta-regression analyses, as shown in Supplementary Figure S2.

## Discussion

By providing an assessment of NG epidemiology in Europe from 1949 to 2021, this study identified two distinct and contrasting epidemiologies arising from infection transmission in two different sexual transmission networks. The first epidemiology is that of NG transmission in heterosexual sexual networks. Here, prevalence of urogenital infection averaged at 1% among the general population over the last few decades, a level comparable to the global prevalence level [6]. Prevalence of infection showed strong hierarchy with higher prevalence in populations at higher risk of infection (such as FSWs), as has been observed for other STIs [23,24,49,50]. Prevalence was particularly high, as expected, among symptomatic populations and populations suspected of exposure to STIs.

*Neisseria gonorrhoeae* urogenital prevalence was found to decline at a relative rate of 3% per year (Table 4), but this rate of decline is substantially slower than that needed to attain the WHO target of 90% incidence reduction by 2030. The decline may be attributed to safer sex practices following recognition of the HIV epidemic [51,52], improved awareness of STIs [53], enhanced access to HIV and STI services [9,10,54] and/or changes to structure of sexual networks following changes in socioeconomic conditions [26].

The second epidemiology is that of NG transmission in sexual networks of MSM, MSWs and transgender people where infection is being transmitted through both anal and oral sex. Higher prevalence of infection is found in these networks. *Neisseria gonorrhoeae* prevalence among MSM, MSWs and transgender people was estimated at 6% for anorectal infection and at 4% for oropharyngeal infection, much higher than the prevalence of urogenital infection in this population at only 1%. Prevalence was also found to be increasing at a relative rate of 2% per year for both anorectal and oropharyngeal infections (Tables 5–6).

These findings are concerning given these estimated high levels of infection, the widespread AMR observed in gonococcal strains and the critical role played by the oropharynx in the development of gonococcal AMR [2,55-59]. The oropharynx can be inhabited by diverse *Neisseria* species, capable of harbouring a range of genetic elements associated with antibiotic resistance, acquired through various past exposures to antibiotics [55,56].

The increase in infection transmission in sexual networks of MSM and MSWs may reflect higher number of sexual partners facilitated by availability of social media apps [2,60,61], increased use of chemsex [62-66] and the introduction of HIV pre-exposure prophylaxis leading to increases in unprotected and risky sexual behaviour [67,68].

The results indicate several other notable findings. Highest NG prevalence was observed among sexual contacts of persons infected with NG or CT, regardless of the anatomical site of infection. This finding highlights the criticality of partner notification and expedited partner therapy services and affirms the role of these services as part of the WHO Global Health Sector Strategy on STIs [9,10]. In all analyses and regardless of anatomical site, men had a higher prevalence of infection than women. This finding further supports the proportionally higher role of sexual networks of high-risk groups among MSM, MSWs and transgender people in NG transmission in Europe. Prevalence of infection among infertility clinic attendees was similar to that in the general population, unlike in other regions such as the Middle East and North Africa where it was considerably higher [5], perhaps reflecting better access to reproductive and screening services. No differences in prevalence were observed by age group, European subregion or country income level, regardless of anatomical site, suggesting that exposure to infection may not be restricted to younger persons but also occurs among older cohorts, and that infection transmission may not vary substantially within Europe.

This study has limitations. Data availability and quality varied across European countries, anatomical sites and population groups. Studies were missing for 16 of the 53 European countries. Western and northern Europe had a higher number of conducted studies compared with other subregions, as shown in Supplementary Table S3. Research on urogenital infections was more common, whereas studies on oropharyngeal infections were scarce. Prevalence studies were common in general populations where NG prevalence is typically lower. However, in key populations like MSM and FSWs, where prevalence levels were higher, the number of conducted studies was comparatively lower.

The included studies demonstrated diversity in assay types, sample sizes, sampling methods and response rates. Over time, there were shifts in the usage of diagnostic assays, and most studies used convenience sampling instead of probability-based methods. A consistent trend was noted, with smaller studies reporting higher prevalence levels, indicating a strong small-study effect across all analyses. Formal assessment of publication bias was not possible due to methodological challenges associated with evaluating it for proportion measures [69]. Given the extensive scope of this review encompassing multiple diverse population types, certain population categories, such as MSM, transgender people and MSWs were combined for analysis as they are epidemiologically related and exhibit somewhat related levels of infection risk.

However, data were available for 37 countries, constituting 90% of Europe’s total population [70]. The countries without data primarily comprised small populations and were not representative of the overall European population. Despite variations in assay type, sampling method and response rate among studies, these factors did not appear to influence prevalence, as indicated by the meta-regression analyses across anatomical sites. Although there was heterogeneity in prevalence, a large portion of this heterogeneity was explained by epidemiological factors and study methods in subsequent meta-regression analyses.

The study incorporated an extensive volume of NG prevalence data, comprising 1,573 prevalence measures and including 2,199 stratified measures. This dataset surpasses those published for other regions [6], enabling diverse analyses across anatomical sites and facilitating the identification of infection patterns in different population types. Despite variations in the number of studies across categories and strata, there was a meaningful number of studies even in categories and strata with sparse data points. Most studies were published after 2010, a period marked by significant improvement in study design, diagnostic assays and laboratory methods compared with earlier years. While the review was based on data up to 2021, it is improbable that very recent data would appreciably impact the findings, as changes in prevalence typically take several years to materialise and be observed. Therefore, the limitations of this study are not likely to have affected the findings, and the results should be representative, applicable and generalisable for the European Region.

## Conclusions

*Neisseria gonorrhoeae* epidemiology in Europe up to 2021, presents with two distinct and contrasting epidemiologies. Infection transmission through vaginal sex appears to be decreasing leading to lower prevalence of urogenital infection in populations exposed to NG through heterosexual sexual networks. Meanwhile, infection transmission through anal and oral sex is increasing leading to higher prevalence of anorectal and oropharyngeal infection among populations such as MSM. This increased transmission could foster opportunities for new drug-resistant strains to emerge. Europe is far from achieving the WHO target of 90% incidence reduction by 2030. Controlling infection transmission requires a major expansion of STI services combined with the introduction of novel interventions such as vaccines. The current vaccines under development may provide a much-needed tool to fundamentally tackle NG infection and its drug resistance in Europe and elsewhere.

## Supplementary Data

10393000 Supplement
