# Supplementary material for: Epidemiology of gonorrhoea: systematic review, meta-analyses, and meta-regressions, World Health Organization European Region, 1949 to 2021
Source: Euro Surveill. 2024 Feb 29;29(9):2300226. doi: 10.2807/1560-7917.ES.2024.29.9.2300226 (PMC10986664; doi:10.2807/1560-7917.ES.2024.29.9.2300226)
Supplement: Supplement [file 23-00226_ABU-RADDAD_Supplement.pdf]

# Supplementary Material: Additional inserts of methods and results

This supplementary material is hosted by *Eurosurveillance* as supporting information alongside the article 'Epidemiology of gonorrhoea: systematic review, meta-analyses, and meta-regressions, World Health Organization European Region, 1949 to 2021', on behalf of the authors, who remain responsible for the accuracy and appropriateness of the content. The same standards for ethics, copyright, attributions, and permissions as for the article apply. Supplements are not edited by *Eurosurveillance* and the journal is not responsible for the maintenance of any links or email addresses provided therein.

**Table of Contents**

Supplementary Table S1. Preferred Reporting Items for Systematic Reviews and Meta-analyses (PRISMA) checklist [1]. ....... 3

Supplementary Box S1. Search strategies used to identify studies reporting *Neisseria gonorrhoeae* infection prevalence in Europe. ....... 6

Supplementary Box S2. Variables extracted from relevant reports meeting the inclusion criteria. ....... 7

Supplementary Table S2. Range of quality components applicable to prevalence studies [2]. ....... 8

Supplementary Box S3. Predictors selected a priori and included in univariable and multivariable meta-regression analyses ....... 9

Supplementary Box S4. List of articles from which *Neisseria gonorrhoeae* prevalence measures in Europe were extracted ....... 10

Supplementary Table S3. Number of *Neisseria gonorrhoeae* prevalence measures by European subregion and country/territory. ....... 33

Supplementary Table S4. Summary of precision assessment and risk of bias (ROB) assessment for studies reporting *Neisseria gonorrhoeae* prevalence in Europe. ....... 34

Supplementary Figure S1. Forest plots presenting outcomes of the pooled urogenital *Neisseria gonorrhoeae* prevalence among the different populations in Europe. ....... 35

Supplementary Table S5. Univariable and multivariable meta-regression analyses for *Neisseria gonorrhoeae* prevalence in urogenital specimens in Europe using the year of publication instead of the year of data collection as the time variable. ....... 46

Supplementary Table S6. Univariable and multivariable meta-regression analyses for *Neisseria gonorrhoeae* prevalence in anorectal specimens in Europe using the year of publication instead of the year of data collection as the time variable. ....... 48

Supplementary Table S7. Univariable and multivariable meta-regression analyses for *Neisseria gonorrhoeae* prevalence in oropharyngeal specimens in Europe using the year of publication instead of the year of data collection as the time variable. ....... 50

Supplementary Figure S2. Forest plots displaying the results of cumulative meta-analyses, utilizing the year of publication as the ordering variable, to confirm the trend in *Neisseria gonorrhoeae* prevalence identified by the meta-regression analyses. ....... 52

References ....... 62

**Supplementary Table S1. Preferred Reporting Items for Systematic Reviews and Meta-analyses (PRISMA) checklist [1].**

| Section and topic | Item # | Checklist item | Location where item is reported |
|---|---|---|---|
| **Title** | | | |
| Title | 1 | Identify the report as a systematic review. | Title |
| **Abstract** | | | |
| Abstract | 2 | See the PRISMA 2020 for Abstracts checklist (table 2). | Abstract section |
| **Introduction** | | | |
| Rationale | 3 | Describe the rationale for the review in the context of existing knowledge. | Introduction section |
| Objectives | 4 | Provide an explicit statement of the objective(s) or question(s) the review addresses. | Introduction section |
| **Methods** | | | |
| Eligibility criteria | 5 | Specify the inclusion and exclusion criteria for the review and how studies were grouped for the syntheses. | Methods: Study selection eligibility criteria and section |
| Information sources | 6 | Specify all databases, registers, websites, organisations, reference lists and other sources searched or consulted to identify studies. Specify the date when each source was last searched or consulted. | Methods: Data sources and search strategy section; Study selection and eligibility criteria section |
| Search strategy | 7 | Present the full search strategies for all databases, registers and websites, including any filters and limits used. | Methods: Data sources and search strategy section; Supplementary Box S1 |
| Selection process | 8 | Specify the methods used to decide whether a study met the inclusion criteria of the review, including how many reviewers screened each record and each report retrieved, whether they worked independently, and if applicable, details of automation tools used in the process. | Methods: Study selection and eligibility criteria section |
| Data collection process | 9 | Specify the methods used to collect data from reports, including how many reviewers collected data from each report, whether they worked independently, any processes for obtaining or confirming data from study investigators, and if applicable, details of automation tools used in the process. | Methods: Data extraction and synthesis section |
| Data items | 10a | List and define all outcomes for which data were sought. Specify whether all results that were compatible with each outcome domain in each study were sought (e.g., for all measures, time points, analyses), and if not, the methods used to decide which results to collect. | Methods: Study selection and eligibility criteria section; Data extraction and synthesis section; Supplementary Box S2 |
| | 10b | List and define all other variables for which data were sought (e.g., participant and intervention characteristics, funding sources). Describe any assumptions made about any missing or unclear information. | Methods: Study selection and eligibility criteria section; Data extraction and synthesis section; Box 2; Supplementary Box S2 |
| Study risk of bias assessment | 11 | Specify the methods used to assess risk of bias in the included studies, including details of the tool(s) used, how many reviewers assessed each study and whether they worked independently, and if applicable, details of automation tools used in the process. | Methods: Precision and risk of bias assessments section |
| Effect measures | 12 | Specify for each outcome the effect measure(s) (e.g. risk ratio, mean difference) used in the synthesis or presentation of results. | Methods: Meta-analyses section; Meta-regressions section; Supplementary Box S3 |
| Synthesis methods | 13a | Describe the processes used to decide which studies were eligible for each synthesis (e.g. tabulating the study intervention characteristics and comparing against the planned groups for each synthesis (item #5)). | Methods: Meta-analyses section; Meta-regressions section; Supplementary Box S3 |
| | 13b | Describe any methods required to prepare the data for presentation or synthesis, such as handling of missing summary statistics, or data conversions. | Methods: Meta-analyses section; Meta-regressions section |
| | 13c | Describe any methods used to tabulate or visually display results of individual studies and syntheses. | Methods: Meta-analyses section; Meta-regressions section |
| | 13d | Describe any methods used to synthesise results and provide a rationale for the choice(s). If meta-analysis was performed, describe the model(s), method(s) to identify the presence and extent of statistical heterogeneity, and software package(s) used. | Methods: Meta-analyses section |
| | 13e | Describe any methods used to explore possible causes of heterogeneity among study results (e.g. subgroup analysis, metaregression). | Methods: Meta-regressions section; Supplementary Box S3 |

| | | | |
|---|---|---|---|
| | 13f | Describe any sensitivity analyses conducted to assess robustness of the synthesised results. | Methods: Meta-analyses section; Meta-regressions section |
| Reporting bias assessment | 14 | Describe any methods used to assess risk of bias due to missing results in a synthesis (arising from reporting biases). | N/A |
| Certainty assessment | 15 | Describe any methods used to assess certainty (or confidence) in the body of evidence for an outcome. | N/A |
| **Results** | | | |
| Study selection | 16a | Describe the results of the search and selection process, from the number of records identified in the search to the number of studies included in the review, ideally using a flow diagram (see fig 1). | Results: Search results section; Figure 1 |
| | 16b | Cite studies that might appear to meet the inclusion criteria, but which were excluded, and explain why they were excluded. | Results: Search results section; Figure 1 |
| Study characteristics | 17 | Cite each included study and present its characteristics. | Results: Scope of evidence for the prevalence measures section; Tables 1-3; Supplementary Box S4 |
| Risk of bias in studies | 18 | Present assessments of risk of bias for each included study. | Results: Precision and risk of bias assessments section; Supplementary Table S3 |
| Results of individual studies | 19 | For all outcomes, present, for each study: (a) summary statistics for each group (where appropriate) and (b) an effect estimate and its precision (e.g. confidence/credible interval), ideally using structured tables or plots. | Tables 1-6; Supplementary Figure S1 |
| Results of syntheses | 20a | For each synthesis, briefly summarise the characteristics and risk of bias among contributing studies. | Results: Precision and risk of bias assessments section; Supplementary Table S3 |
| | 20b | Present results of all statistical syntheses conducted. If meta-analysis was done, present for each the summary estimate and its precision (e.g. confidence/credible interval) and measures of statistical heterogeneity. If comparing groups, describe the direction of the effect. | Results: Pooled estimates for NG prevalence section; Tables 1-3; Supplementary Figure S1 |
| | 20c | Present results of all investigations of possible causes of heterogeneity among study results. | Results: Associations with NG prevalence section; Tables 4-6 |
| | 20d | Present results of all sensitivity analyses conducted to assess the robustness of the synthesised results. | Results: Sensitivity analyses to confirm the findings section; Supplementary Tables S4-S6; Supplementary Figure S2 |
| Reporting biases | 21 | Present assessments of risk of bias due to missing results (arising from reporting biases) for each synthesis assessed. | N/A |
| Certainty of evidence | 22 | Present assessments of certainty (or confidence) in the body of evidence for each outcome assessed. | N/A |
| **Discussion** | | | |
| Discussion | 23a | Provide a general interpretation of the results in the context of other evidence. | Discussion section |
| | 23b | Discuss any limitations of the evidence included in the review. | Discussion section |
| | 23c | Discuss any limitations of the review processes used. | Discussion section |
| | 23d | Discuss implications of the results for practice, policy, and future research. | Conclusions section |
| **Other information** | | | |
| Registration and protocol | 24a | Provide registration information for the review, including register name and registration number, or state that the review was not registered. | N/A |
| | 24b | Indicate where the review protocol can be accessed, or state that a protocol was not prepared. | N/A |
| | 24c | Describe and explain any amendments to information provided at registration or in the protocol. | N/A |
| Support | 25 | Describe sources of financial or non-financial support for the review, and the role of the funders or sponsors in the review. | Funding statement |
| Competing interests | 26 | Declare any competing interests of review authors. | Conflict of interest statement |

| | | | |
|---|---|---|---|
| Availability of data, code, and other materials | 27 | Report which of the following are publicly available and where they can be found: template data collection forms; data extracted from included studies; data used for all analyses; analytic code; any other materials used in the review. | Data availability statement |

Abbreviations: NA: Not applicable, NG: *Neisseria gonorrhoeae*.

**Supplementary Box S1. Search strategies used to identify studies reporting *Neisseria gonorrhoeae* infection prevalence in Europe.**

| **PubMed** |
|---|
| "Neisseria gonorrhoeae"[Mesh] OR "Gonorrhea"[Mesh] OR "Pelvic Inflammatory Disease"[Mesh] OR "Epididymitis"[Mesh] OR "Orchitis"[Mesh] OR "Seminal vesicles"[Mesh] OR "Neisseria gonorrhoeae"[Text] OR "Gonorrhoeae"[Text] OR "Gonorrhea"[Text] OR "Gonococcus"[Text] OR "Gonococci"[Text] OR "Gonococcal"[Text] OR "Gonococcal infection"[Text] OR "Pelvic inflammatory disease"[Text] OR "Gonococcal epididymitis"[Text] OR "Orchi-epididymitis"[Text] OR "Orchiepididymitis"[Text] OR "seminal vesicle disease"[Text] OR "Seminal vesiculitis"[Text] OR "Infertility"[Mesh] OR "Fertility"[Mesh] OR "Reproductive Techniques, Assisted"[Mesh] OR "Infertility"[Text] OR "Infertile"[Text] OR "Fertility"[Text] OR "Reproductive"[Text] OR "Subfertility"[Text] OR "Subfertile"[Text] OR "Sub-fertility"[Text] OR "Sub-fertile"[Text] AND ("Europe"[MeSH] OR "Israel"[MeSH] OR "Turkey"[MeSH] OR "USSR"[MeSH] OR "Cyprus"[MeSH] OR Albania*[Text] OR Andorra*[Text] OR Armenia*[Text] OR Austria*[Text] OR Azerbaijan*[Text] OR Belarus*[Text] OR Belgi*[Text] OR Bosnia*[Text] OR Bosnia and Herzegovina[Text] OR Bulgaria*[Text] OR Croatia*[Text] OR Cypr*[Text] OR Czech Republic[Text] OR Czech*[Text] OR Denmark*[Text] OR Danish[Text] OR Estonia*[Text] OR Finland*[Text] OR Finnish[Text] OR France*[Text] OR French[Text] OR Georgia*[Text] OR German*[Text] OR Gree*[Text] OR Hungar*[Text] OR Iceland*[Text] OR Ireland*[Text] OR Irish[Text] OR Israel*[Text] OR Ital*[Text] OR Kazakh*[Text] OR Kyrgyz*[Text] OR Latvia*[Text] OR Lithuania*[Text] OR Luxembourg*[Text] OR Malta*[Text] OR Monac*[Text] OR Montenegr*[Text] OR Netherlands*[Text] OR Dutch[Text] OR Norway*[Text] OR Norweg*[Text] OR Poland*[Text] OR Polish[Text] OR Portug*[Text] OR Republic of Moldova*[Text] OR Moldov*[Text] OR Romania*[Text] OR Russia*[Text] OR Russian Federation*[Text] OR San Marino*[Text] OR Serbia*[Text] OR Slovakia*[Text] OR Slovenia*[Text] OR Spain*[Text] OR Spanish[Text] OR Swed*[Text] OR Switzerland*[Text] OR Swiss[Text] OR Tajik*[Text] OR Yugoslav*[Text] OR Republic of Macedonia*[Text] OR Macedonia*[Text] OR Turkey*[Text] OR Turkmen*[Text] OR Ukrain*[Text] OR United Kingdom*[Text] OR Great Britain*[Text] OR UK[Text] OR Uzbek*[Text]) |
| **Embase** |
| exp gonorrhea / or exp 6eisseria gonorrhoeae / or exp epididymitis / or exp orchitis / or exp pelvic inflammatory disease/ or gonorrhea.mp. or 6eisseria gonorrhoeae.mp. or gonorrhoeae.mp. or gonococcus.mp. or gonococci.mp. or gonococcal.mp. or gonococcal infection.mp. or pelvic inflammatory disease.mp. or gonococcal epididymitis.mp. or orchi-epididymitis.mp. or orchiepididymitis.mp. or seminal vesicle disease.mp. or seminal disease.mp. or seminal vasculitis.mp. or exp infertility/ or exp fertility/ or exp infertility therapy/ or exp reproductive procedure/ or reproductive.mp. or infertility.mp. or infertile.mp. or fertility.mp. or subfertility.mp. or subfertile.mp. or sub-fertility.mp. or sub-fertile.mp. and (exp Europe/ or exp Cyprus/ or exp Israel/ or exp USSR or exp Turkey republic/) or (Albania* or Andorra* or Armenia* or Austria* or Azerbaijan* or Belarus* or Belgi* or Bosnia* or Bosnia and Herzegovina or Bulgaria* or Croatia* or Cypr* or Czech Republic* or Czech* or Denmark* or Danish* or Estonia* or Finland* or Finnish* or France* or French* or Georgia* or German* or Gree* or Hungar* or Iceland* or Ireland* or Irish* or Israel* or Ital* or Kazakh* or Kyrgyz* or Latvia* or Lithuania* or Luxembourg* or Malta* or Monac* or Montenegr* or Netherlands* or Dutch* or Norway* or Norweg* or Poland* or Polish* or Portug* or Republic of Moldova* or Moldov* or Romania* or Russia* or Russian Federation* or San Marino* or Serbia* or Slovakia* or Slovenia* or Spain* or Spanish* or Swed* or Switzerland* or Swiss* or Tajik* or Yugoslav Republic of Macedonia* or Macedonia* or Turk* or Turkmen* or Ukrain* or United Kingdom* or Great Britain* or UK or Uzbek*).mp. |

Abbreviations: NG: Neisseria gonorrhoeae; USSR: Union of Soviet Socialist Republics.

**Supplementary Box S2. Variables extracted from relevant reports meeting the inclusion criteria.**

- Author(s)
- Year of publication
- Full citation
- Country
- City
- Study design
- Sampling methodology
- Year(s) of data collection
- Study site
- Study population
- Population characteristics (e.g., sex and age)
- Response rate
- Sample size of tested population
- Number of participants positive for *Neisseria gonorrhoeae* infection
- Reported *N. gonorrhoeae* prevalence
- Anatomical site urogenital (urethral, vaginal, endocervical, urine, and semen), anorectal, oropharyngeal, serum, and unclear)
- Type of assay used for gonorrhea infection ascertainment (nucleic acid amplification test/polymerase chain reaction, culture, gram staining, direct fluorescent antibody assay, blood tested for antibodies, unclear)

**Supplementary Table S2. Range of quality components applicable to prevalence studies [2].**

| | |
|---|---|
| 1. Was the study's target population a close representation of the national population in relation to relevant variables? | Met in the study design. The study investigated prevalence in all population groups. The meta-regression analyses also explored the impact of sampling method on observed prevalence. |
| 2. Was the sampling frame a true or close representation of the target population? | Met in the study design. Included as the probability-based vs. non-probability-based sampling domain. The meta-regression analyses also explored the impact of sampling method on observed prevalence. |
| 3. Was some form of random selection used to select the sample, OR was a census undertaken? | Met in the study design. Included as the probability-based vs. non-probability-based sampling domain. The meta-regression analyses also explored the impact of sampling method on observed prevalence. |
| 4. Was the likelihood of nonresponse bias minimal? | Met in the study design. Included as the response rate domain. |
| **Internal validity** | **Relevance to our study** |
| 5. Were data collected directly from the subjects (as opposed to a proxy)? | Met in the study design. The inclusion criteria specified that only studies based on biomarkers collected directly from individuals are included. |
| 6. Was an acceptable case definition used in the study? | Met in the study design. A standardized and consistent case definition was used, that of *Neisseria gonorrhoeae* infection. |
| 7. Was the study instrument that measured the parameter of interest shown to have validity and reliability? | Met in the study design. Only studies that used diagnostic assays measuring specific biomarkers were included. The reported assays have acceptable specificity and sensitivity and are commonly used in research and clinical settings. The meta-regression analyses also explored the impact of assay type on observed prevalence. |
| 8. Was the same mode of data collection used for all subjects? | Met in the study design. It is standard for STI studies involving biomarkers, by design, to use a consistent mode of data collection from all subjects recruited for a study, including specimen type and assay type. |
| 9. Was the length of the shortest prevalence period for the parameter of interest appropriate? | Met in the study design. Included studies reported point prevalence measures, that is prevalence based on a cross-section survey at a specific and defined time. |
| 10. Were the numerator(s) and denominator(s) for the parameter of interest appropriate? | Met in the study design. The numerator and denominator were defined with no ambiguity: number of positive *N. gonorrhoeae* cases over total number of tested subjects. |

STI: Sexually transmitted infection.

**Supplementary Box S3. Predictors selected a priori and included in univariable and multivariable meta-regression analyses.**

1- Population type as defined in Box 2
2- Sex
3- Age groups classified to best fit reported data as:
  - <20 years old
  - 20-29 years old
  - 30-39 years old
  - ≥40 years old
  - Mixed ages

4- Europe subregion as defined in Box 1
5- National income as classified by the World Bank [3]
6- Assay type:
  - NAAT/PCR
  - Culture
  - Gram staining
  - Other[a]

7- Sample size:
  - <200
  - ≥200

8- Sampling method:
  - Probability-based sampling
  - Non-probability-based sampling

9- Response rate:
  - ≥80%
  - <80%
  - Unclear

10- Year of data collection category
  - <2000
  - 2000-2010
  - >2010

11- Year of data collection as a linear term
12- Year of publication category
  - <2005
  - 2005-2014
  - ≥2015

13- Year of publication as a linear term

[a]Other assays include unclear testing technique, enzyme immunoassay, complement fixation, or mixed testing techniques.
Abbreviations: NAAT: Nucleic acid amplification test; PCR: Polymerase chain reaction.

**Supplementary Box S4. List of articles from which *Neisseria gonorrhoeae* prevalence measures in Europe were extracted.**


1. Achterbergh R, van der Helm JJ, van den Boom W, et al. Is rectal douching and sharing douching equipment associated with anorectal chlamydia and gonorrhoea? A cross-sectional study among men who have sex with men. Sex Transm Infect 2017; 93(6): 431-7.
2. Achterbergh RCA, Drückler S, van Rooijen MS, et al. Sex, drugs, and sexually transmitted infections: A latent class analysis among men who have sex with men in Amsterdam and surrounding urban regions, the Netherlands. Drug Alcohol Depend 2020; 206: 107526.
3. Acik Y, Gungor L, Asci Toraman Z. Prevalance and clinical importance of some pathogen microorganisms found in the cervix of women who apply for voluntary abortion. [Turkish]. Erciyes Tip Dergisi 2004; 26(2): 55-61.
4. Adelantado Lacasa M, Beristain X. Prevalence of Mycoplasma genitalium infection and antibiotic resistance in Navarra (North Spain). Sex Transm Infect 2019; 95(7): 549.
5. Adler MW. Trends for gonorrhea and pelvic inflammatory disease in England and Wales and for gonorrhea in a defined population. Am J Obstet Gynecol 1980; 138(7 Pt 2): 901-4.
6. Adler MW, Belsey EM, Rogers JS. Sexually transmitted diseases in a defined population of women. Br Med J (Clin Res Ed) 1981; 283(6283): 29-32.
7. Agacfidan A, Moncada J, Aydin D, et al. Prevalence of Chlamydia trachomatis and Neisseria gonorrhoeae in Turkey among men With urethritis. Sex Transm Dis 2001; 28(11): 630-2.
8. Aguirrebengoa O, Vera Garcia M, Arias Ramirez D, et al. Low use of condom and high STI incidence among men who have sex with men in PrEP programs. PLoS One 2021; 16(2): e0245925.
9. Agusti C, Montoliu A, Mascort J, et al. Missed opportunities for HIV testing of patients diagnosed with an indicator condition in primary care in Catalonia, Spain. Sex Transm Infect 2016; 92(5): 387-92.
10. Ahmed-Jushuf IH, Arya OP, Hobson D, et al. Ciprofloxacin treatment of chlamydial infections of urogenital tracts of women. Genitourinary Medicine 1988; 64(1): 14-7.
11. Akgul A, Kadioglu A, Koksal MO, Ozmez A, Agacfidan A. Sexually transmitted agents and their association with leucocytospermia in infertility clinic patients. Andrologia 2018; 50(10): e13127.
12. Akhvlediani T, Mdivani K, Ispireli M, et al. Sexually transmitted infections in military personnel in the country of Georgia. International Journal of STD and AIDS 2015; 1): 112.
13. Al-Jilaihawi S, Borg K, Jamieson K, Maguire S, Hodes D. Clinical characteristics of children presenting with a suspicion or allegation of historic sexual abuse. Archives of Disease in Childhood 2018; 103(6): 533-9.
14. Albus C, Köhler G. [Is there is seasonal incidence of acute adnexitis?]. Zentralbl Gynakol 1988; 110(13): 824-6.
15. Alexandre C. [Study of 534 cases of lowered male fertility]. Gynecol Obstet (Paris) 1971; 70(4): 377-400.
16. Almeida N, Melo M, Soares I, Carvalho H. [Screening of Human Immunodeficiency Virus and Other Sexually Transmitted Infections in a Group of Sex Workers in Indoor Settings in the Porto Metropolitan Area]. Acta Med Port 2020; 33(3): 166-73.
17. Amirkhanian YA, Kelly JA, Kabakchieva E, et al. High-risk sexual behavior, HIV/STD prevalence, and risk predictors in the social networks of young Roma (Gypsy) men in Bulgaria. J Immigr Minor Health 2013; 15(1): 172-81.
18. Andersen BL, Nielsen FH. [Gonorrhoea in young women admitted to a gynaecology department]. Ugeskr Laeger 1974; 136(36): 2027-9.
19. Andersson N, Allard A, Lidgren Y, Boman J, Nylander E. Are Urogenital Symptoms Caused by Sexually Transmitted Infections and Colonizing Bacteria? J Low Genit Tract Dis 2021b; 25(3): 232-5.
20. Andersson N, Ejnestrand J, Lidgren Y, Allard A, Boman J, Nylander E. Are Swedish swingers a risk group for sexually transmitted infections? Int J STD AIDS 2021a; 32(5): 427-34.
21. Anestad G, Bruu AL, Thorvaldsen J, et al. [Urogenital Chlamydia trachomatis infection in a student population]. Tidsskr Nor Laegeforen 1984; 104(7): 499-502.
22. Anisimova N, Ivanova T, Guschin A, et al. Undiscovered burden of stis in Russia: Current system shortcomings. Sexually Transmitted Infections 2011; 1): A100-A1.
23. Annan NT, Sullivan AK, Nori A, et al. Rectal chlamydia--a reservoir of undiagnosed infection in men who have sex with men. Sex Transm Infect 2009; 85(3): 176-9.
24. Ansart S, Hochedez P, Perez L, Bricaire F, Caumes E. Sexually transmitted diseases diagnosed among travelers returning from the tropics. J Travel Med 2009; 16(2): 79-83.
25. Apers L, Florence E, Crucitti T, Anwar N. Lymphogranuloma venereum among patients presenting at the HIV/STI clinic in Antwerp, Belgium: A case series. Acta Gastro-Enterologica Belgica 2017; 80(3): 385-7.
26. Apers L, Koole O, Bottieau E, et al. Incidence of HCV and sexually transmitted diseases among hiv positive msm in antwerp, belgium, 2001-2011. Acta Clin Belg 2013; 68(6): 421-6.
27. Arando M, Fernandez-Naval C, Mota-Foix M, et al. Early syphilis: risk factors and clinical manifestations focusing on HIV-positive patients. BMC Infect Dis 2019; 19(1): 727.
28. Arumainayagam JT, de Silva Y, Shahmanesh M. Anaerobic vaginosis: study of male sexual partners. Int J STD AIDS 1991; 2(2): 102-4.
29. Arvidson M, Hellberg D, Mårdh PA. Sexually transmitted diseases in Swedish women with experience of casual sex with men of foreign nationalities within Sweden. Acta Obstet Gynecol Scand 1995; 74(10): 794-8.
30. Arya OP, Hobson D, Hart CA, Bartzokas C, Pratt BC. Evaluation of ciprofloxacin 500 mg twice daily for one week in treating uncomplicated gonococcal, chlamydial, and non-specific urethritis in men. Genitourinary Medicine 1986; 62(3): 170-4.
31. Arya OP, Mallinson H, Goddard AD. Epidemiological and clinical correlates of chlamydial infection of the cervix. Br J Vener Dis 1981; 57(2): 118-24.
32. Asicioglu O, Gungorduk K, Ozdemir A, et al. Single daily dose of moxifloxacin versus ofloxacin plus metronidazole as a new treatment approach to uncomplicated pelvic inflammatory disease: a multicentre prospective randomized trial. Eur J Obstet Gynecol Reprod Biol 2013; 171(1): 116-21.
33. Asmaryan A, Grigoryan S, Hovhannisyan E. Unprotected sex with non-commercial partners as the main risk factor to get sti for female sex workers in armenia. Sexually Transmitted Infections 2017; 93(Supplement 2): A98-A9.
34. Asmaryan A, Papoyan A, Hakobyan A, et al. Prevalence of STI/HIV and assessment of risky behaviours among sex workers. Sexually Transmitted Infections Conference: STI and AIDS World Congress 2013; 89(SUPPL. 1).
35. Avonts D, Piot P. Genital infections in women undergoing therapeutic abortion. Eur J Obstet Gynecol Reprod Biol 1985; 20(1): 53-9.
36. Avonts D, Sercu M, Heyerick P, Vandermeeren I, Piot P. Sexually transmitted diseases and Chlamydia trachomatis in women consulting for contraception. J R Coll Gen Pract 1989; 39(327): 418-20.
37. Ayerdi Aguirrebengoa O, Vera Garcia M, Rueda Sanchez M, et al. Risk factors associated with sexually transmitted infections and HIV among adolescents in a reference clinic in Madrid. PLoS One 2020; 15(3): e0228998.
38. Bacon PM. Social distribution of sexually transmitted diseases. A survey of female clinic registrations. Br J Vener Dis 1979; 55(4): 295-9.
39. Bailey JV, Farquhar C, Owen C, Mangtani P. Sexually transmitted infections in women who have sex with women. Sex Transm Infect 2004; 80(3): 244-6.

40. Balbi G, Piscitelli V, Di Grazia F, Martini S, Balbi C, Cardone A. [Acute pelvic inflammatory disease: comparison of therapeutic protocols]. Minerva Ginecol 1996; 48(1-2): 19-23.
41. Banani S, Schlaeffer F, Leibenson L, et al. [Prevalence of sexually transmitted diseases (STD) in HIV positive women in southern Israel]. Harefuah 2013; 152(4): 204-6, 48.
42. Banerjee P, Thorley N, Radcliffe K. A service evaluation comparing home-based testing to clinic-based testing for Chlamydia and gonorrhoea in Birmingham and Solihull. Int J STD AIDS 2018; 29(10): 974-9.
43. Bardov PV, Lebediuk MM, Stepanenko VI. [Complex diagnosis of chronic prostatitis complicated by disorders in sexual and reproductive functions]. Lik Sprava 2001; (3): 77-81.
44. Barlow D, Nayyar K, Phillips I, Barrow J. Diagnosis of gonorrhoea in women. Br J Vener Dis 1976; 52(5): 326-8.
45. Barthell E, Woelber L, Hellner K, et al. Baseline characteristics and prevalence of HPV 6, 11, 16, 18 in young German women participating in phase III clinical trials of a quadrivalent HPV (6/11/16/18) vaccine. Arch Gynecol Obstet 2009; 279(6): 803-7.
46. Bartholomew RD, Kerry-Barnard S, Beckley-Hoelscher ND, et al. Alcohol use, cigarette smoking, vaping and number of sexual partners: A cross-sectional study of sexually active, ethnically diverse, inner city adolescents. Health Expect 2021; 24(3): 1009-14.
47. Bartunek J, Scholtes G. [Significance of modern gonorrhea diagnostics in the management of inflammatory gynecologic diseases]. Geburtshilfe Frauenheilkd 1972; 32(11): 928-31.
48. Bassiri M, Mårdh PA, Domeika M. Multiplex AMPLICOR PCR screening for Chlamydia trachomatis and Neisseria gonorrhoeae in women attenting non-sexually transmitted disease clinics. The European Chlamydia Epidemiology Group. J Clin Microbiol 1997; 35(10): 2556-60.
49. Bayette J, Jreige R, Marchandin H, et al. [Prevalence of Chlamydia trachomatis, Neisseria gonorrhoeae and Mycoplasma genitalium infections in the emergency department]. Pathol Biol (Paris) 2013; 61(6): 245-9.
50. Bebear C, Le Roy C, Le Hen I, et al. Performance of the Bio-Rad Dx CT/NG/MG assay for simultaneous detection of chlamydia trachomatis, Neisseria gonorrhoeae and mycoplasma genitalium in urogenital samples. Sexually Transmitted Infections 2011; 1): A74.
51. Beckett H, Nulty K, Barber T. Assessing self-taken vaginal swabs compared to clinician sampling in women presenting with vaginal discharge only in our central London location. HIV Medicine 2018; 19(Supplement 2): S128.
52. Bell G, Ward H, Day S, et al. Partner notification for gonorrhoea: a comparative study with a provincial and a metropolitan UK clinic. Sex Transm Infect 1998; 74(6): 409-14.
53. Beltrami C, Manfredi R, D'Antuono A, Chiodo F, Varotti C. Sexually-transmitted infections in adolescents and young adults in a large city of Northern Italy: a nine-year prospective survey. New Microbiol 2003; 26(3): 233-41.
54. Belzunegui J, Rodríguez-Arrondo F, González C, et al. Musculoskeletal infections in intravenous drug addicts: report of 34 cases with analysis of microbiological aspects and pathogenic mechanisms. Clin Exp Rheumatol 2000; 18(3): 383-6.
55. Benjamin L, Araujo MA, Martin-Bouyer G. [Asymptomatic cases of gonorrhea and syphilis in 2 family planning clinics in the Paris Region]. Gynecologie 1978; 29(3): 303-7.
56. Benn PD, Rooney G, Carder C, et al. Chlamydia trachomatis and Neisseria gonorrhoeae infection and the sexual behaviour of men who have sex with men. Sex Transm Infect 2007; 83(2): 106-12.
57. Bennett A, Jeffery K, O'Neill E, Sherrard J. Outbreak or illusion: Consequences of "improved" diagnostics for gonorrhoea. Sexually Transmitted Infections 2015; 1): A23-A4.
58. Bercot B, Amarsy R, Goubard A, et al. Assessment of coinfection of sexually transmitted pathogen microbes by use of the anyplex II STI-7 molecular kit. J Clin Microbiol 2015; 53(3): 991-3.
59. Bernier A, Rumyantseva T, Reques L, et al. HIV and other sexually transmitted infections among female sex workers in Moscow (Russia): prevalence and associated risk factors. Sex Transm Infect 2020; 96(8): 601-7.
60. Bevan CD, Johal BJ, Mumtaz G, Ridgway GL. Clinical, laparoscopic and microbiological findings in acute salpingitis: Report on a United Kingdom cohort. British Journal of Obstetrics and Gynaecology 1995; 102(5): 407-14.
61. Bhaduri S, Howells V. Evaluation of the Roche Cobas Amplicor polymerase chain reaction test for gonorrhoea in a low prevalence area. Int J STD AIDS 2007; 18(11): 796-7.
62. Bhagey A, Foster K, Ralph S, et al. High prevalence of anti-hepatitis A IgG in a cohort of UK HIV-negative men who have sex with men: implications for local hepatitis A vaccine policy. Int J STD AIDS 2018; 29(10): 1007-10.
63. Bhatt K, Goodall L. An audit of gonorrhoea management in a UK sexual health clinic. Sexually Transmitted Infections 2015; 1): A54.
64. Bialo-Wojcicka E, Mlynarczyk-Bonikowska B, Winiarska A, et al. Syphilis in patients of the Department of Dermatology and Venereology at Medical University of Warsaw in 2015 - Epidemiological and clinical characteristics, and coexistence of other sexually transmitted diseases. Przeglad Dermatologiczny 2017; 104(6): 597-605.
65. Bilagi A, Mahavarkar S. Teenage pregnancy: An experience in a university hospital. BJOG: An International Journal of Obstetrics and Gynaecology 2018; 125(Supplement 2): 111.
66. Bird E, Micanovic C, McCrann C, Hegazi A. Keeping on top of BV-an audit of bacterial vaginosis and STI screening in gum clinic attendees requesting a termination of pregnancy. Sexually Transmitted Infections 2016; 92(Supplement 1): A97.
67. Bjartling C, Osser S, Persson K. The association between Mycoplasma genitalium and pelvic inflammatory disease after termination of pregnancy. BJOG 2010; 117(3): 361-4.
68. Bjornelius E, Lidbrink P, Jensen JS. Mycoplasma genitalium in non-gonococcal urethritis--a study in Swedish male STD patients. Int J STD AIDS 2000; 11(5): 292-6.
69. Blackwell AL, Thomas PD, Wareham K, Emery SJ. Health gains from screening for infection of the lower genital tract in women attending for termination of pregnancy. Lancet 1993; 342(8865): 206-10.
70. Body G, Quentin R, Lyon J, Pinon G, Lansac J. [Acute gonococcal salpingitis]. J Gynecol Obstet Biol Reprod (Paris) 1985; 14(4): 477-85.
71. Boeke AJ, Dekker JH, Peerbooms PG. A comparison of yield from cervix versus vagina for culturing Candida albicans and Trichomonas vaginalis. Genitourin Med 1993; 69(1): 41-3.
72. Boesecke C, Kimhofer T, Tocha C, et al. Are we reaching the at-risk patient populations for hiv and other stis? Topics in Antiviral Medicine 2021; 29(1): 267.
73. Boiko I, Golparian D, Krynytska I, Unemo M. High prevalence of Chlamydia trachomatis, Neisseria gonorrhoeae and particularly Trichomonas vaginalis diagnosed using US FDA-approved Aptima molecular tests and evaluation of conventional routine diagnostic tests in Ternopil, Ukraine. Apmis 2019; 127(9): 627-34.
74. Bolumburu C, Zamora V, Munoz-Algarra M, Portero-Azorin F, Escario JA, Ibanez-Escribano A. Trichomoniasis in a tertiary hospital of Madrid, Spain (2013-2017): prevalence and pregnancy rate, coinfections, metronidazole resistance, and endosymbiosis. Parasitol Res 2020; 119(6): 1915-23.
75. Bond E, Lu D, Herweijer E, et al. Sexually transmitted infections after bereavement - a population-based cohort study. BMC Infect Dis 2016; 16(1): 419.
76. Borges da Costa J, Azevedo J, Santo I. Sexually transmitted infections and related sociodemographic factors in Lisbon's major Venereology Clinic: a descriptive study of the first 4 months of 2007. J Eur Acad Dermatol Venereol 2010; 24(7): 811-4.

77. Borges-Costa J, Matos C, Pereira F. Sexually transmitted infections in pregnant adolescents: prevalence and association with maternal and foetal morbidity. J Eur Acad Dermatol Venereol 2012; 26(8): 972-5.
78. Bos JM, Fennema JSA, Postma MJ. Cost-effectiveness of HIV screening of patients attending clinics for sexually transmitted diseases in Amsterdam. Aids 2001; 15(15): 2031-6.
79. Boucher A, Meybeck A, Alidjinou K, et al. Clinical and virological features of acute hepatitis A during an ongoing outbreak among men who have sex with men in the North of France. Sex Transm Infect 2019; 95(1): 75-7.
80. Bourgeois-Nicolaos N, Jaureguy F, Pozzi-Gaudin S, et al. Benefits of Rapid Molecular Diagnosis of Chlamydia Trachomatis and Neisseria Gonorrhoeae Infections in Women Attending Family Planning Clinics. Sex Transm Dis 2015; 42(11): 652-3.
81. Bovet R, Calanchini E, Sakellarides E, Jéquier N. [Pharyngeal and anorectal gonorrhea. Incidence in Lausanne in 1981]. Schweiz Med Wochenschr 1982; 112(43): 1494-7.
82. Bozděch V, Drazdáková M, Kvícera J, Zítková A. [Trichomonal infections in promiscuous women]. Angew Parasitol 1982; 23(2): 65-9.
83. Bozicevic I, Lepej SZ, Rode OD, et al. Prevalence of HIV and sexually transmitted infections and patterns of recent HIV testing among men who have sex with men in Zagreb, Croatia. Sexually Transmitted Infections 2012; 88(7): 539-44.
84. Bozicevic I, Rode OD, Lepej SZ, et al. Prevalence of sexually transmitted infections among men who have sex with men in Zagreb, Croatia. AIDS and Behavior 2009; 13(2): 303-9.
85. Braun DL, Kouyos R, Ghenzi R, Grube C, Weber R, Gunthard H. Increasing rates of STI are linked to reduced condom use in patients with primary HIV-1 infection. Topics in Antiviral Medicine 2014; 22(E-1): 540-1.
86. Braun DL, Marzel A, Steffens D, et al. High Rates of Subsequent Asymptomatic Sexually Transmitted Infections and Risky Sexual Behavior in Patients Initially Presenting With Primary Human Immunodeficiency Virus-1 Infection. Clin Infect Dis 2018; 66(5): 735-42.
87. Braunholtz-Speight J, Turner E, Wallace H, Schoeman S, Evans A. Working the streets-targetting migrant sexworkers through dedicated outreach. Sexually Transmitted Infections 2017; 93(Supplement 1): A94.
88. Bremer V, Haar K, Gassowski M, Hamouda O, Nielsen S. STI tests and proportion of positive tests in female sex workers attending local public health departments in Germany in 2010/11. BMC Public Health 2016; 16(1): 1175.
89. Bremer V, Jansen K, Steffen G, et al. Prevalence of genital infections with chlamydia trachomatis (CT), neisseria gonorrhoea (NG) and Trichomonas Vaginalis (TV) in clients of public hiv counselling institutions in north rhine-westphalia, Germany: The STI-HIT Study. Sexually Transmitted Infections Conference: STI and AIDS World Congress 2013; 89(SUPPL. 1).
90. Breustedt W. [The incidence of genital chlamydial infections in patients of a venereologic consultation clinic]. Dermatol Monatsschr 1989; 175(7): 443-9.
91. Brihmer C, Kallings I, Nord CE, Brundin J. Salpingitis; aspects of diagnosis and etiology: a 4-year study from a Swedish capital hospital. Eur J Obstet Gynecol Reprod Biol 1987; 24(3): 211-20.
92. Bro F. Vaginal microbial flora in women with and without vaginal discharge registered in general practice. Dan Med Bull 1989; 36(5): 483-5.
93. Broad CE, Furegato M, Harrison MA, et al. High prevalence of coinfection of azithromycin-resistant Mycoplasma genitalium with other STIs: a prospective observational study of London-based symptomatic and STI-contact clinic attendees. Sex Transm Infect 2021; 97(1): 63-8.
94. Brorson JE, Holmberg I, Nygren B, Seeberg S. Vancomycin-sensitive strains of Neisseria gonorrhoeae. A problem for the diagnostic laboratory. Br J Vener Dis 1973; 49(5): 452-3.
95. Brosh-Nissimov T, Kedem R, Ophir N, Shental O, Keller N, Amit S. Management of sexually transmissible infections in the era of multiplexed molecular diagnostics: a primary care survey. Sex Health 2018; 15(4): 298-303.
96. Brunner A, Nemes-Nikodem E, Mihalik N, Marschalko M, Karpati S, Ostorhazi E. Incidence and antimicrobial susceptibility of Neisseria gonorrhoeae isolates from patients attending the national Neisseria gonorrhoeae reference laboratory of Hungary. BMC Infect Dis 2014; 14: 433.
97. Bryman I, Granberg S, Norstrom A. Reduced incidence of postoperative endometritis by the use of Laminaria tents in connection with first trimester abortion. Acta Obstet Gynecol Scand 1988; 67(4): 323-5.
98. Buekens P, Hubrechts JM, Romero M, Vekemans M. Chlamydia trachomatis and induced abortion. European Journal of Sexually Transmitted Diseases 1984; 1(3-4): 133-6.
99. Burns F, Curran B, Tobin R, Kell P. An urban epidemic--an audit of an inner city young persons' clinic. Int J STD AIDS 2002; 13(1): 52-4.
100. Burns FM, Mercer CH, Evans AR, Gerry CJ, Mole R, Hart GJ. Increased attendances of people of eastern European origin at sexual health services in London. Sex Transm Infect 2009; 85(1): 75-8.
101. Burton R, Duncan S, Wardle E, Patel C, Clarke E. Unintended consequences: the potential impacts of the British Association for Sexual Health and HIV (BASHH) 2019 gonorrhoea guidelines. Int J STD AIDS 2021; 32(4): 344-51.
102. Cabello Ubeda A, Fernandez Roblas R, Garcia Delgado R, et al. Anorectal Lymphogranuloma Venereum in Madrid: A Persistent Emerging Problem in Men Who Have Sex With Men. Sex Transm Dis 2016; 43(7): 414-9.
103. Cameron RL, Cuschieri K, Pollock KGJ. Baseline HPV prevalence in rectal swabs from men attending a sexual health clinic in Scotland: assessing the potential impact of a selective HPV vaccination programme for men who have sex with men. Sex Transm Infect 2020; 96(1): 55-7.
104. Camporiondo MP, Farchi F, Ciccozzi M, et al. Detection of HPV and co-infecting pathogens in healthy Italian women by multiplex real-time PCR. Infez Med 2016; 24(1): 12-7.
105. Carlsson I, Breding K, Larsson PG. Complications related to induced abortion: a combined retrospective and longitudinal follow-up study. BMC Womens Health 2018; 18(1): 158.
106. Carne CA, Gibbs J, Delaney A, et al. Prevalence, clinical features and quantification of genital non-viral infections. Int J STD AIDS 2013; 24(4): 273-7.
107. Casin I, Vexiau-Robert D, De La Salmoniere P, Eche A, Grandry B, Janier M. High prevalence of Mycoplasma genitalium in the lower genitourinary tract of women attending a sexually transmitted disease clinic in Paris, France. Sex Transm Dis 2002; 29(6): 353-9.
108. Cassidy S, Artykov R, White J. Equivocal Aptima Combo 2 results: What do they mean in clinical practice? Sexually Transmitted Infections Conference: 4th Joint BASHH ASTDA Meeting Brighton United Kingdom Conference Publication: 2012; 88(SUPPL. 1).
109. Castriciano S, Montrucchio G, Moglia R, et al. Comparison of copan uriswab with BD probetec urine preservative transport kit for preservation and detection of CT and NG in the probetec assay. Sexually Transmitted Infections 2011; 1): A277-A8.
110. Catarino R, Cherkaoui A, Trellu LT, Yaron M. Who should be screened for Chlamydia trachomatis infection? Three years' experience at a University Hospital in Switzerland. Journal of Infection in Developing Countries 2018; 12(3): 208-10.
111. Cavilla S, Churchill D, Dean G, Smith S. Greater focus on incident HIV infections is required to end the HIV epidemic. HIV Medicine 2020; 21(S4): 16-74.
112. Ceccarani C, Marangoni A, Severgnini M, et al. Rectal Microbiota Associated With Chlamydia trachomatis and Neisseria gonorrhoeae Infections in Men Having Sex With Other Men. Front Cell Infect Microbiol 2019; 9: 358.
113. Cevenini R, Possati G, La Placa M. Chlamydia trachomatis infection in infertile women. Fernstrom Foundation Series 1982; Vol. 2: 189-92.
114. Chalker VJ, Jordan K, Ali T, Ison C. Real-time PCR detection of the mg219 gene of unknown function of Mycoplasma genitalium in men with and without non-gonococcal urethritis and their female partners in England. J Med Microbiol 2009; 58(Pt 7): 895-9.

115. Champion JK, Ross JDC. Referral patterns between primary care and genitourinary medicine. International Journal of STD and AIDS 1999; 10(2): 121-3.
116. Chan SY, Hegazi A, Beardall A, Hay P. An audit on the management of pelvic inflammatory disease (PID). Sexually Transmitted Infections Conference: 4th Joint BASHH ASTDA Meeting Brighton United Kingdom Conference Publication: 2012; 88(SUPPL. 1).
117. Cherkaoui A, Renzi G, Mombelli M, et al. Comparison of analytical performances of the Roche Cobas 6800 CT/NG assay with the Abbott m2000 Real Time CT/NG assay for detecting Chlamydia trachomatis and Neisseria gonorrhoeae. J Med Microbiol 2019; 68(2): 197-200.
118. Childs T, Simms I, Alexander S, Eastick K, Hughes G, Field N. Rapid increase in lymphogranuloma venereum in men who have sex with men, United Kingdom, 2003 to September 2015. Euro Surveill 2015; 20(48): 30076.
119. Chima-Okereke C, Burton A, Blackwell A. A comparison of demographic details, source of referral, and spectrum of infection in patients under 16 years attending Singleton Hospital Genitourinary Medicine Department in 1997 and 2000. Int J STD AIDS 2002; 13(12): 859-60.
120. Chiodo E, Latino MA, Chiodo F, Gordini G. Genital infections in adult men observed at Ospedale S. Anna department of pathologic anatomy, Turin, from july 1, 1986 to june 30, 1990. Giornale di Malattie Infettive e Parassitarie 1991; 43(2): 160-1.
121. Christiansen MAD, Lowhagen GB. Sexually transmitted diseases and sexual behaviour in men attending an outpatients' clinic for gay men in Gotheburg, Sweden. Acta Dermato-Venereologica 2000; 80(2): 136-9.
122. Christophersen J, Menne T, Friis-Moller A, Nielsen JO, Hansted B, Ohlenschloeger J. Sexually transmitted diseases in hetero-, homo- and bisexual males in Copenhagen. Dan Med Bull 1988; 35(3): 285-8.
123. Chung E, Waters L, Mercey D, Edwards S. High rates of recreational drug use (RDU) in HIV+ men who have sex with men (MSM) with sexually transmitted infections (STI). HIV Medicine 2014; 3): 50.
124. Cicinelli E, De Ziegler D, Nicoletti R, et al. Chronic endometritis: correlation among hysteroscopic, histologic, and bacteriologic findings in a prospective trial with 2190 consecutive office hysteroscopies. Fertil Steril 2008; 89(3): 677-84.
125. Cielniak I, Siwak E, Pazdan I, Szymanska-Kotwica B, Parczewski M. Clinical outcomes of pre-exposure prophylaxis from clinical center in Warsaw, Poland. HIV Medicine 2019; 20(S9): 317-37.
126. Claeys P, Ismailov R, Rathe S, et al. Sexually transmitted infections and reproductive health in Azerbaijan. Sex Transm Dis 2001; 28(7): 372-8.
127. Clarivet B, Picot E, Marchandin H, et al. Prevalence of Chlamydia trachomatis, Neisseria gonorrhoeae and Mycoplasma genitalium in asymptomatic patients under 30 years of age screened in a French sexually transmitted infections clinic. Eur J Dermatol 2014; 24(5): 611-6.
128. Coffey AJ, Dwyer J. An insight into stis commonly encountered ina university health department - Prevalence and predictive factors. Irish Journal of Medical Science 2018; 187(3 Supplement 1): S27-S8.
129. Cohen L. The "pill", promiscuity, and venereal disease. Br J Vener Dis 1970; 46(2): 108-10.
130. Colon RL, Vives N, Vall M, et al. Sexually transmitted infections surveillance through a primary care sentinel network, Catalonia, 2011–2014. International Journal of STD & AIDS 2015; 26(11_suppl): 1-121.
131. Conway RJ, Cook S, Malone C, Bone S, Hassan-Ibrahim MO, Soni S. Resistance-guided treatment of Mycoplasma genitalium infection at a UK sexual health centre. Int J STD AIDS 2021; 32(8): 758-65.
132. Coorevits L, Traen A, Bingé L, et al. Identifying a consensus sample type to test for Chlamydia trachomatis, Neisseria gonorrhoeae, Mycoplasma genitalium, Trichomonas vaginalis and human papillomavirus. Clin Microbiol Infect 2018; 24(12): 1328-32.
133. Corbeto EL, Lugo R, Martró E, et al. [Prevalence and determining factors of acquiring C. trachomatis infection among adolescents and young adults in Catalonia]. Enferm Infecc Microbiol Clin 2011; 29(2): 96-101.
134. Cordtz T. A three months survey of gonorrhea in the VD-clinic of Nuuk, Greenland. Arctic Med Res 1988; 47 Suppl 1: 683-5.
135. Cornier N, Petrova E, Cavailler P, et al. Optimising the management of vaginal discharge syndrome in Bulgaria: cost effectiveness of four clinical algorithms with risk assessment. Sex Transm Infect 2010; 86(4): 303-9.
136. Cousins D, Cutforth R, Ghanem M, Riddell L. Limited clinical benefits of endocervical microscopy when the use of nucleic acid testing for N. gonorrhoea is standard practice in symptomatic women. International Journal of STD and AIDS 2015; 1): 101.
137. Cox C, Watt AP, McKenna JP, Coyle PV. Gardnerella vaginalis and Mollicute detection in rectal swabs from men who have sex with men. Int J STD AIDS 2017; 28(7): 708-14.
138. Creighton S, Edwards S, Welch J, Miller R. News from the frontline: sexually transmitted infections in teenagers attending a genitourinary clinic in south east London. Sex Transm Infect 2002; 78(5): 349-51.
139. Cresswell F, Ross S, Booth T, et al. An outbreak of shigella flexneri infection amongst MSM in Brighton: A descriptive study. HIV Medicine 2014; 3): 136.
140. Criado MD, Garrido CF, San Martin EM, et al. Is vertical transmission of chlamydia trachomatis a health problem in Spain? European Journal of Pediatrics 2019; 178(11): 1621-2.
141. Cribier B, Asch PH, Tardieu JC. Declining rates of gonorrhoea and syphilis in Strasbourg, France: a 20-year study. Genitourin Med 1994; 70(4): 273-7.
142. Currie S, Nicol E, Thompson T, Tomassi S, McQuillan O. It's what's inside that counts: Review of sexual health care provision in a large high-security men's prison. HIV Medicine 2018; 19(Supplement 2): S136.
143. Cuypers WJ, Niekamp AM, Keesmekers R, et al. High prevalence of HIV, other sexually transmitted infections and risk profile in male commercial sex workers who have sex with men in the Netherlands. Sexually Transmitted Infections 2011; 1): A127.
144. Cwikel JG, Lazer T, Press F, Lazer S. Sexually transmissible infections among illegal female sex workers in Israel. Sexual Health 2006; 3(4): 301-3.
145. D'Antuono A, Andalo F, Carla EM, De Tommaso S. Prevalence of STDs and HIV infection among immigrant sex workers attending an STD centre in Bologna, Italy. Sex Transm Infect 2001; 77(3): 220.
146. D'Antuono A, De Tommaso S, Calderoni O, Negosanti M. Sexually transmitted diseases among foreigners in Bologna. [Italian]. Giornale Italiano di Dermatologia e Venereologia 1999; 134(1): 11-5.
147. Dahl E. [Venereal diseases among seamen. Venereal diseases among Norwegian and foreign staff on a Norwegian cruise ship in the Carribbean during 2 and one-half years of sailing]. Tidsskr Nor Laegeforen 1974; 94(34-36): 2372-4.
148. Dakshina S, Cowdy C, Dave J, Sampson M, Sarner L, Tong W. Confirmatory tests for oropharyngeal gonorrhoea in GUM clinics. HIV Medicine 2014; 3): 104-5.
149. Dale AW, Wilson JD, Forster GE, Daniels D, Brook MG. Management of epididymo-orchitis in Genitourinary Medicine clinics in the United Kingdom's North Thames region 2000. Int J STD AIDS 2001; 12(5): 342-5.
150. Dan M, Kaneti N, Levin D, Poch F, Samra Z. Vaginitis in a gynecologic practice in Israel: causes and risk factors. Isr Med Assoc J 2003; 5(9): 629-32.
151. Dao TL, Canard N, Hoang VT, et al. Risk factors for symptoms of infection and microbial carriage among French medical students abroad. Int J Infect Dis 2020; 100: 104-11.
152. Dautigny M, Ebel A, Merlin S, Souchet A, Ronsin C. Prevalence of Trichomonas Vaginalis in france and comparison with chlamydia trachomatis and Neisseria Gonorrhoeae as determined by the APTIMA Trichomonas Vaginalis nucleic acid amplification assay. Sexually Transmitted Infections Conference: STI and AIDS World Congress 2013; 89(SUPPL. 1).

153. Dave S, Kerry SR, Oakeshott P, McGregor F, Cannon E, Stephenson JM. Women's health study of Mycoplasma genitalium: A feasibility study. Sexually Transmitted Infections Conference: 4th Joint BASHH ASTDA Meeting Brighton United Kingdom Conference Publication: 2012; 88(SUPPL. 1).
154. David N. Sexually transmitted infections in elderly people. Sexually Transmitted Infections 2000; 76(3): 222-.
155. David Parker R, Regier MD, Widmeyer J, Honaker J, Ruutel K. Reported contraceptive use, risk behaviours and STIs among military conscripts in Estonian defence forces. Int J STD AIDS 2015; 26(11): 815-20.
156. Davies B, Daley S, Brown J, Talbot A, Fifer H, Wilson J. An outbreak of high level azithromycin resistant gonorrhoea in a UK City-actions taken by the clinical team and lessons learnt. Sexually Transmitted Infections 2016; 92(Supplement 1): A13.
157. Davies O, Costelloe S, Cross G, et al. Impact of rectal gonorrhoea and chlamydia on HIV viral load in the rectum: potential significance for onward transmission. Int J STD AIDS 2017; 28(10): 1034-7.
158. Day S, Smith J, Perera S, Jones S, Kinsella R. Beyond the binary: sexual health outcomes of transgender and non-binary service users of an online sexual health service. Int J STD AIDS 2021; 32(10): 896-902.
159. De Baetselier I, Apers L, Platteau T, et al. The impact of physical restriction measures imposed during the two waves of COVID-19 on chlamydia and gonorrhea diagnoses in Belgium. Results of an sexually transmitted infection clinic. Int J STD AIDS 2021a; 32(11): 998-1003.
160. De Baetselier I, Osbak KK, Smet H, Kenyon CR, Crucitti T. Take three, test one: a cross-sectional study to evaluate the molecular detection of Chlamydia trachomatis and Neisseria gonorrhoeae in pooled pharyngeal, anorectal and urine samples versus single-site testing among men who have sex with men in Belgium. Acta Clin Belg 2020; 75(2): 91-5.
161. De Baetselier I, Reyniers T, Platteau T, et al. Recurrent Sexually Transmitted Infections Among a Cohort of Men Who Have Sex With Men Using Preexposure Prophylaxis in Belgium Are Highly Associated With Sexualized Drug Use. Sex Transm Dis 2021b; 48(10): 726-32.
162. De Baetselier I, Smet H, Abdellati S, et al. Evaluation of the 'Colli-Pee', a first-void urine collection device for self-sampling at home for the detection of sexually transmitted infections, versus a routine clinic-based urine collection in a one-to-one comparison study design: efficacy and acceptability among MSM in Belgium. BMJ Open 2019; 9(4): e028145.
163. De Francesco D, Verboeket SO, Underwood J, et al. Patterns of co-occurring comorbidities in people living with HIV. Open Forum Infectious Diseases 2018; 5(11).
164. de Jong AS, Rahamat-Langendoen JC, van Alphen P, et al. Large two-centre study into the prevalence of Mycoplasma genitalium and Trichomonas vaginalis in the Netherlands. Int J STD AIDS 2016; 27(10): 856-60.
165. de Salazar A, Espadafor B, Fuentes-Lopez A, et al. Comparison between Aptima Assays (Hologic) and the Allplex STI Essential Assay (Seegene) for the diagnosis of Sexually transmitted infections. PLoS One 2019; 14(9): e0222439.
166. de Sanjose S, Munoz N, Bosch FX, et al. Sexually transmitted agents and cervical neoplasia in Colombia and Spain. Int J Cancer 1994; 56(3): 358-63.
167. De Silva D, Peters J, Cole K, et al. Whole-genome sequencing to determine transmission of Neisseria gonorrhoeae: an observational study. Lancet Infect Dis 2016; 16(11): 1295-303.
168. de Vries HJ, van der Helm JJ, Schim van der Loeff MF, van Dam AP. Multidrug-resistant Neisseria gonorrhoeae with reduced cefotaxime susceptibility is increasingly common in men who have sex with men, Amsterdam, the Netherlands. Euro Surveill 2009; 14(37).
169. De Vrieze NHN, Van Rooijen M, Van Der Loeff MFS, De Vries HJC. Anorectal and inguinal lymphogranuloma venereum among men who have sex with men in Amsterdam, the Netherlands: Trends over time, symptomatology and concurrent infections. Sexually Transmitted Infections 2013; 89(7): 548-52.
170. De Vrieze NHN, Van Rooijen MS, Van De Loeff MS, De Vries HJC. Additional gonorrhea and chlamydia infections found with rapid follow-up screening in men who have sex with men with an indication for hiv postexposure prophylaxis. Sexually Transmitted Diseases 2014; 41(8): 515-7.
171. de Wit JB, van den Hoek JA, Sandfort TG, van Griensven GJ. Increase in unprotected anogenital intercourse among homosexual men. Am J Public Health 1993; 83(10): 1451-3.
172. Defraye A, Van Beckhoven D, Sasse A. Surveillance of sexually transmitted infections among persons living with HIV. Int J Public Health 2011; 56(2): 169-74.
173. Del Prete R, Ronga L, Lestingi M, et al. Simultaneous detection and identification of STI pathogens by multiplex Real-Time PCR in genital tract specimens in a selected area of Apulia, a region of Southern Italy. Infection 2017; 45(4): 469-77.
174. den Heijer CDJ, Hoebe C, van Liere G, et al. A comprehensive overview of urogenital, anorectal and oropharyngeal Neisseria gonorrhoeae testing and diagnoses among different STI care providers: a cross-sectional study. BMC Infect Dis 2017; 17(1): 290.
175. Desclaux A, Touati A, Neau D, et al. Extra-rectal lymphogranuloma venereum in France: A clinical and molecular study. Sexually Transmitted Infections 2018; 94(1): 3-8.
176. Detels R, Green AM, Klausner JD, et al. The incidence and correlates of symptomatic and asymptomatic Chlamydia trachomatis and Neisseria gonorrhoeae infections in selected populations in five countries. Sex Transm Dis 2011; 38(6): 503-9.
177. Devadass V, Leverett K, Macauley S, Singh S. Evaluation of chlamydia trachomatis (CT) and neisseria gonorrhoeae (GC) infections in female sex workers (FSW) attending a dedicated sex worker clinic. Sexually Transmitted Infections 2016; 92(Supplement 1): A86-A7.
178. Dewilde A, Torpier A, Dewulf J, Wattre P. Evaluation of procedures for laboratory diagnosis of genital infections by Chlamydia Trachomatis in 2310 patients. [French]. Medecine et Maladies Infectieuses 1986; 16(1): 6-12.
179. Dhairyawan R, Creighton S, Sivyour L, Anderson J. Testing the fathers: carrying out HIV and STI tests on partners of pregnant women. Sex Transm Infect 2012; 88(3): 184-6.
180. Diquelou JY, Pia P, Tesquier L, Henry-Suchet J, Gicquel JM, Boyer S. [The role of Chlamydia trachomatis in the infectious etiology of extra-uterine pregnancy]. J Gynecol Obstet Biol Reprod (Paris) 1988; 17(3): 325-32.
181. Dixon J, Chauhan M, Sankar N. Comparative study of STI prevalence as an indicator of increasing risk-taking sexual behaviour in new HIV diagnoses in MSM over a 10-year period. HIV Medicine 2014; 3): 85.
182. Dolapci I, Tekeli A, Ozsan M, Yaman O, Ergin S, Elhan A. Detecting of Mycoplasma genitalium in male patients with urethritis symptoms in Turkey by polymerase chain reaction. Saudi Med J 2005; 26(1): 64-8.
183. Dolenec N. [Bacteriological findings in the cervical mucus of sterile women]. Jugosl Ginekol Opstet 1980; 20(3-4): 203-7.
184. Dolling D, Desai M, Saunders J, et al. Who accesses prep? An analysis of baseline data in the PROUD pilot. HIV Medicine 2014; 3): 15.
185. Domeika M, Savicheva A, Shipitsyna E, et al. International validation of amplisens naat systems manufactured in Russia for diagnosis of Neisseria Gonorrhoeae, chlamydia trachomatis, mycoplasma genitalium and trichomonas vaginalis. Sexually Transmitted Infections 2011; 1): A353-A4.
186. Dona V, Smid JH, Kasraian S, et al. Mismatch Amplification Mutation Assay-Based Real-Time PCR for Rapid Detection of Neisseria gonorrhoeae and Antimicrobial Resistance Determinants in Clinical Specimens. J Clin Microbiol 2018; 56(9): e00365.
187. Donati M, Di Francesco A, D'Antuono A, et al. Chlamydia trachomatis serovar distribution and other concurrent sexually transmitted infections in heterosexual men with urethritis in Italy. Eur J Clin Microbiol Infect Dis 2009; 28(5): 523-6.
188. Downing J, Cook PA, Madden HC, Phillips-Howard PA, Higgins SP, Bellis MA. Management of cases testing positive for gonococcal infection in a community-based chlamydia screening programme. Sex Transm Infect 2010; 86(6): 474-7.
189. Drasnar J, Uher J, Klanica J. [Results of microbiological diagnosis in complicated female gonorrhea with tubal sterility using laparoscopy (proceedings)]. Cesk Gynekol 1978; 43(7): 538-9.

190. Drew RJ, Cormican M, Machale E, Fleming C. Low rate of co-infection in patients presenting with Chlamydia trachomatis to a sexually transmitted infection clinic in Galway, Ireland. Int J STD AIDS 2007; 18(11): 796.
191. Druckler S, Daans C, Hoornenborg E, et al. HIV and STI positivity rates among transgender people attending two large STI clinics in the Netherlands. Sex Transm Infect 2021.
192. Druckler S, van Rooijen MS, de Vries HJC. Chemsex Among Men Who Have Sex With Men: a Sexualized Drug Use Survey Among Clients of the Sexually Transmitted Infection Outpatient Clinic and Users of a Gay Dating App in Amsterdam, the Netherlands. Sex Transm Dis 2018; 45(5): 325-31.
193. Druckler S, van Rooijen MS, de Vries HJC. Substance Use and Sexual Risk Behavior Among Male and Transgender Women Sex Workers at the Prostitution Outreach Center in Amsterdam, the Netherlands. Sex Transm Dis 2020; 47(2): 114-21.
194. Dudareva-Vizule S, Haar K, Sailer A, Wisplinghoff H, Wisplinghoff F, Marcus U. Prevalence of pharyngeal and rectal Chlamydia trachomatis and Neisseria gonorrhoeae infections among men who have sex with men in Germany. Sex Transm Infect 2014; 90(1): 46-51.
195. Dukers NHTM, Spaargaren J, Geskus RB, Beijnen J, Coutinho RA, Fennema HSA. HIV incidence on the increase among homosexual men attending an Amsterdam sexually transmitted disease clinic: Using a novel approach for detecting recent infections. Aids 2002; 16(10): F19-F24.
196. Dukers-Muijrers N, Leenen J, Hoebe C. Implementing home-sampling strategies to engage young heterosexuals in chlamydia trachomatis re-testing and peer-testing. Sexually Transmitted Infections 2019; 95(Supplement 1): A89.
197. Dukers-Muijrers NH, Niekamp AM, Brouwers EE, Hoebe CJ. Older and swinging; need to identify hidden and emerging risk groups at STI clinics. Sex Transm Infect 2010; 86(4): 315-7.
198. Durglishvili G, Galdava G. Influence of epididymitis on reproductive function. Georgian Med News 2013; (215): 32-5.
199. Duron S, Panjo H, Bohet A, et al. Prevalence and risk factors of sexually transmitted infections among French service members. PLoS One 2018; 13(4): e0195158.
200. Duthie SJ, Hobson D, Tait IA, et al. Morbidity after termination of pregnancy in first trimester. Genitourin Med 1987; 63(3): 182-7.
201. Ebel A, Venard C, Brunengo D, Merlin S. Prevalence of Mycoplasma genitalium in France and comparison with Chlamydia trachomatis and Neisseria gonorrhoeae using transcriptionmediated amplification assays. International Journal of STD and AIDS 2015; 1): 68.
202. Eccleston K, Richards M. Sexual health in men who have sex with men in a rural setting: Is this a population at high risk of acquiring human immunodeficiency virus? HIV Medicine 2014; 3): 98-9.
203. Edouard S, Tamalet C, Tissot-Dupont H, et al. Evaluation of self-collected rectal swabs for the detection of bacteria responsible for sexually transmitted infections in a cohort of HIV-1-infected patients. Journal of Medical Microbiology 2017; 66(6): 693-7.
204. Edouard S, Tissot-Dupont H, Dubourg G, et al. Mycoplasma genitalium, an agent of reemerging sexually transmitted infections. Apmis 2017b; 125(10): 916-20.
205. Eggert J, Sundquist K, van Vuuren C, Fianu-Jonasson A. The clinical diagnosis of pelvic inflammatory disease - Reuse of electronic medical record data from 189 patients visiting a Swedish university hospital emergency department. BMC Women's Health 2006; 6 (no pagination).
206. Eggert-Kruse W, Batschulat K, Demirakca T, Strowitzki T. Male immunity to the chlamydial 60 kDa heat shock protein (HSP 60) - associated with semen quality? Andrologia 2015; 47(1): 66-76.
207. Eggert-Kruse W, Gerhard I, Hofmann H, Runnebaum B, Petzoldt D. Influence of microbial colonization on sperm-mucus interaction in vivo and in vitro. Hum Reprod 1987; 2(4): 301-8.
208. Eggert-Kruse W, Hofmann H, Gerhard I, Bilke A, Runnebaum B, Petzoldt D. Effects of antimicrobial therapy on sperm-mucus interaction. Hum Reprod 1988; 3(7): 861-9.
209. Eggert-Kruse W, Reimann-Andersen J, Rohr G, Pohl S, Tilgen W, Runnebaum B. Clinical relevance of sperm morphology assessment using strict criteria and relationship with sperm-mucus interaction in vivo and in vitro. Fertil Steril 1995; 63(3): 612-24.
210. Eksi F, Dikensoy E, Gayyurhan ED, et al. The prevalence of Chlamydia trachomatis and Neisseria gonorrhoeae in the endocervical swab specimens of symptomatic, asymptomatic and infertile women in Turkey. Arch Gynecol Obstet 2011; 283(6): 1349-55.
211. Eldivan O, Evliyaoglu O, Ersoy E, Aksu G, Dilbaz S, Goktolga U. Does screening for vaginal infection have an impact on pregnancy rates in intracytoplasmic sperm injection cycles? Turkish Journal of Obstetrics and Gynecology 2016; 13(1): 11-5.
212. Elsner P, Hartmann AA. Gardnerella vaginalis in a female West German STD clinic population. European Journal of Sexually Transmitted Diseases 1985; 3(1): 43-6.
213. Elsner P, Hartmann AA, Wecker I. Sexually transmittable organisms in the urethra of males with and without urethritis. Zentralbl Bakteriol Mikrobiol Hyg A 1987; 265(1-2): 268-75.
214. Eriksson G, Wanger L. Frequency of N. gonorrhoeae, T. vaginalis, and C. albicans in female venereological patients. A one-year study. Br J Vener Dis 1975; 51(3): 192-7.
215. Esser S, Krotzek J, Dirks H, Scherbaum N, Schadendorf D. Sexual risk behavior, sexually transmitted infections, and HIV transmission risks in HIV-positive men who have sex with men (MSM) - approaches for medical prevention. J Dtsch Dermatol Ges 2017; 15(4): 421-8.
216. Evaldson GR, Fianu S, Jonasson A. Does the hygroscopic property of the laminaria tent imply a risk for ascending infection in legal abortions? Acta Obstetricia et Gynecologica Scandinavica 1986; 65(3): 257-61.
217. Evans BA, Bond RA, MacRae KD. Heterosexual behaviour, risk factors and sexually transmitted infections among self-classified homosexual and bisexual men. International Journal of STD and AIDS 1998; 9(3): 129-33.
218. Evans BA, Bond RA, MacRae KD. Racial origin, sexual behaviour, and genital infection among heterosexual men attending a genitourinary medicine clinic in London (1993-4). Sex Transm Infect 1998a; 74(1): 40-4.
219. Evans BA, Kell PD, Bond RA, MacRae KD. Heterosexual relationships and condom-use in the spread of sexually transmitted diseases to women. Genitourin Med 1995; 71(5): 291-4.
220. Evans DT, Jaleel H, Kinsella MT, Aggarwal V. A retrospective audit of the management and complications of pelvic inflammatory disease. Int J STD AIDS 2008; 19(2): 123-4.
221. Evjen OC, Haram K, Ulstein M. [Gonorrhea, candidiasis and vaginal trichomoniasis in patients requesting legal abortion]. Tidsskr Nor Laegeforen 1979; 99(9-10): 470-1.
222. Fadrhoncova A. Study on gonorrhea. V. Screening of persons endangered by gonococcal infection. [Czech]. Ceskoslovenska dermatologie 1975; 50(4): 268-79.
223. Falk ES, Vandbakk O. Declining trends in some sexually transmitted diseases in Norway between 1975 and 1991 with special reference to a Lapp population. Acta Derm Venereol Suppl (Stockh) 1993; 182: 15-20.
224. Falk L, Fredlund H, Jensen JS. Symptomatic urethritis is more prevalent in men infected with Mycoplasma genitalium than with Chlamydia trachomatis. Sex Transm Infect 2004; 80(4): 289-93.
225. Fanta D, Gebhart W, Gross W, Kokoschka EM, Soltz-Szots J, Thurner J. Results of routine screening for sexually transmitted diseases in Austria over a six-year period. Br J Vener Dis 1979; 55(2): 147-8.
226. Farfour E, Dimi S, Chassany O, et al. Trends in asymptomatic STI among HIV-positive MSM and lessons for systematic screening. PLoS One 2021; 16(6): e0250557.
227. Fata LL, Cotte L, Godinot M, et al. High Rate of Asymptomatic Bacterial Sexually Transmitted Infections (STIs) in Men who Have Sex with Men on Pre Exposure Prophylaxis (PrEP). Open Forum Infectious Diseases 2017; 4(suppl_1): S669-S.

228. Feeney JG, Badri AE. Screening for gonococcal salpingitis. Lancet 1976; 2(7998): 1309.
229. Fennema JS, van Ameijden EJ, Coutinho RA, Van den Hoek A. Clinical sexually transmitted diseases among human immunodeficiency virus-infected and noninfected drug-using prostitutes. Associated factors and interpretation of trends, 1986 to 1994. Sex Transm Dis 1997; 24(6): 363-71.
230. Fenoll A, Berron S, Vazquez JA. Analysis of penicillinase-producing Neisseria gonorrhoeae isolates in Madrid (Spain) from 1983-85. Epidemiol Infect 1987; 99(2): 455-62.
231. Fernandez-Huerta M, Zarzuela F, Barbera MJ, et al. Sexual Transmission of Intestinal Parasites and Other Enteric Pathogens among Men Who Have Sex with Men Presenting Gastrointestinal Symptoms in an STI Unit in Barcelona, Spain: A Cross-Sectional Study. Am J Trop Med Hyg 2019; 101(6): 1388-91.
232. Ferrero DV, Biglino A. Successful use of non-invasive self obtained glans/meatal dry floqswabs (COPAN) for CT/NG detection with the BD probetec ET assay in Northern Italy. Sexually Transmitted Infections 2011; 1): A277.
233. FitzGerald MR, Thirlby D, Bedford CA. The outcome of contact tracing for gonorrhoea in the United Kingdom. Int J STD AIDS 1998; 9(11): 657-60.
234. Folch C, Casabona J, Brugal MT, et al. Sexually transmitted infections and sexual practices among injecting drug users in harm reduction centers in Catalonia. Eur Addict Res 2011; 17(5): 271-8.
235. Folch C, Esteve A, Sanclemente C, et al. Prevalence of human immunodeficiency virus, Chlamydia trachomatis, and Neisseria gonorrhoeae and risk factors for sexually transmitted infections among immigrant female sex workers in Catalonia, Spain. Sex Transm Dis 2008; 35(2): 178-83.
236. Folch C, Sanclemente C, Esteve A, Martró E, Molinos S, Casabona J. [Social characteristics, risk behaviours and differences in the prevalence of HIV/sexually transmitted infections between Spanish and immigrant female sex workers in Catalonia, Spain]. Med Clin (Barc) 2009; 132(10): 385-8.
237. Forbes K, Byrne R, Gupta D. What women want: Opportunities to integrate screening for sexual infections alongside fitting of an intrauterine device. HIV Medicine 2014; 15: 17-159.
238. Foronda-Garcia-Hidalgo C, Liebana-Martos C, Gutierrez-Soto B, Exposito-Ruiz M, Navarro-Mari JM, Gutierrez-Fernandez J. [Prevalence among males from the general population of agents responsible of not ulcerative genital tract infections, assisted in specialized care]. Rev Esp Quimioter 2019; 32(6): 545-50.
239. Forslin L, Falk V, Danielsson D. Changes in the incidence of acute gonococcal and nongonococcal salpingitis. A five-year study from an urban area of central Sweden. Br J Vener Dis 1978; 54(4): 247-50.
240. Forster GE, Pritchard J, Munday PE, Goldmeier D. Incidence of sexually transmitted diseases in rape victims during 1984. Genitourin Med 1986; 62(4): 267-9.
241. Foschi C, Banzola N, Gaspari V, D'Antuono A, Cevenini R, Marangoni A. Evaluation of the aptima assays for the detection of bacterial sexually transmitted infections in a selected population of women. Sexually Transmitted Infections 2017; 93(Supplement 2): A49.
242. Foschi C, Gaspari V, Sgubbi P, Salvo M, D'Antuono A, Marangoni A. Sexually transmitted rectal infections in a cohort of 'men having sex with men'. J Med Microbiol 2018; 67(8): 1050-7.
243. Foschi C, Nardini P, Banzola N, et al. Chlamydia trachomatis infection prevalence and serovar distribution in a high-density urban area in the north of Italy. J Med Microbiol 2016; 65(6): 510-20.
244. Foschi C, Zagarrigo M, Belletti M, Marangoni A, Re MC, Gaspari V. Genital and extra-genital Chlamydia trachomatis and Neisseria gonorrhoeae infections in young women attending a Sexually Transmitted Infections (STI) clinic. New Microbiol 2020; 43(3): 115-20.
245. Fougas J, Hiquet J, Christin E, et al. Sexually transmitted infections among victims attending a French sexual offence centre. Sex Transm Infect 2017; 93(6): 451.
246. Fournet N, Koedijk FD, van Leeuwen AP, van Rooijen MS, van der Sande MA, van Veen MG. Young male sex workers are at high risk for sexually transmitted infections, a cross-sectional study from Dutch STI clinics, the Netherlands, 2006-2012. BMC Infect Dis 2016; 16: 63.
247. Fowler T, Edeghere O, Inglis N, Bradshaw S. Estimating the positive predictive value of opportunistic population testing for gonorrhoea as part of the English Chlamydia Screening Programme. Int J STD AIDS 2013; 24(3): 185-91.
248. Fox H. Non-specific genital infection in a general practice. Br J Vener Dis 1974; 50(2): 125-31.
249. Fragoso J, Guerra D, Cipriano A, et al. Sexually transmitted diseases clinic in a Portuguese Infectious Diseases unit. HIV Medicine 2019; 20(S9): 317-37.
250. Franceschi S, Smith JS, van den Brule A, et al. Cervical infection with Chlamydia trachomatis and Neisseria gonorrhoeae in women from ten areas in four continents. A cross-sectional study. Sex Transm Dis 2007; 34(8): 563-9.
251. Fraser G, McAdams R, Heng J, Macpherson A. Psychosocial and sexual healthcare needs in men selling sex in Glasgow: a retrospective case note review. Sex Transm Infect 2014; 90(6): 504.
252. Frati ER, Fasoli E, Martinelli M, et al. Sexually Transmitted Infections: A Novel Screening Strategy for Improving Women's Health in Vulnerable Populations. Int J Mol Sci 2017; 18(6).
253. Frolova NI, Belokrinitskaya TE, Shipunova EA, Spesivtcev AS, Yakimova YV. Preconception risk factors in young healthy women: cross-sectional study. BJOG: An International Journal of Obstetrics & Gynaecology 2013; 120: 191-225.
254. Fuchs W, Kreuter A, Hellmich M, et al. Asymptomatic anal sexually transmitted infections in HIV-positive men attending anal cancer screening. Br J Dermatol 2016; 174(4): 831-8.
255. Furegato M, Broad C, Phillips L, et al. Importation of ciprofloxacin resistant Neisseria gonorrhoeae into the UK: A public health challenge. Sexually Transmitted Infections 2019; 95(Supplement 1): A298.
256. García-Lechuz JM, Rivera M, Catalán P, Sánchez-Carrillo C, Rodríguez-Créixems M, Bouza E. Differences in curable STDs between HIV and non-HIV populations in Spain. AIDS Patient Care STDS 1999; 13(3): 175-7.
257. Gardner D, French P. An audit of the management and aetiology of proctitis in men who have sex with men (MSM). Sexually Transmitted Infections 2016; 92(Supplement 1): A47.
258. Garner A, Gibbons N, Williams N, Olufunso O, Gupta N, Dewsnap C. Sexual health outreach clinic in a deprived GP setting : A service evaluation. HIV Medicine 2014; 3): 43.
259. Garner AL, Schembri G, Cullen T, Lee V. Should we screen heterosexuals for extra-genital chlamydial and gonococcal infections? Int J STD AIDS 2015; 26(7): 462-6.
260. Gasbarrini N, Dubravic D, Combs L, et al. Increasing integrated testing in community settings through interventions for change, including the Spring European Testing Week. BMC Infect Dis 2021; 21(Suppl 2): 874.
261. Geelen TH, Rossen JW, Beerens AM, et al. Performance of cobas 4800 and m2000 real-timeTM assays for detection of Chlamydia trachomatis and Neisseria gonorrhoeae in rectal and self-collected vaginal specimen. Diagnostic Microbiology and Infectious Disease 2013; 77(2): 101-5.
262. Gellan MC, Ison CA. Declining incidence of gonorrhoea in London: a response to fear of AIDS? Lancet 1986; 2(8512): 920.
263. Genc M, Agacfidan A, Yegenoglu Y, Turan O, Kuru U, Mardh PA. Screening for Chlamydia trachomatis and Neisseria gonorrhoeae in pregnant Turkish women. Eur J Clin Microbiol Infect Dis 1993; 12(5): 395-6.
264. Georgala S, Schulpis K, Georgala C, Karikas G. Gonorrhoea control programme in Athens, 1974-98. Sex Transm Infect 2001; 77(6): 458-9.
265. Gesink DC, Mulvad G, Montgomery-Andersen R, et al. Mycoplasma genitalium presence, resistance and epidemiology in Greenland. Int J Circumpolar Health 2012; 71: 1-8.

266. Geuenich HH, Muller HE. Comparative clinical, microbiologic and serologic studies of the incidence of genital and para-genital infections in prostitutes and women with mainly monogamous relations. Gesundheitswesen (Bundesverband der Arzte des Offentlichen Gesundheitsdienstes (Germany)) 1993; 55(11): 567-76.
267. Ghanem M, Radcliffe K, Allan P. The role of urethral samples in the diagnosis of gonorrhoea in women. Int J STD AIDS 2004; 15(1): 45-7.
268. Ghani AC, Ison CA, Ward H, et al. Sexual partner networks in the transmission of sexually transmitted diseases. An analysis of gonorrhea cases in Sheffield, UK. Sex Transm Dis 1996; 23(6): 498-503.
269. Ghinsberg RC, Nitzan Y. Chlamydia trachomatis direct isolation, antibody prevalence and clinical symptoms in women attending outpatient clinics. New Microbiol 1994; 17(3): 231-42.
270. Giertz G, Kallings I, Nordenvall M, Fuchs T. A prospective study of Chlamydia trachomatis infection following legal abortion. Acta Obstet Gynecol Scand 1987; 66(2): 107-9.
271. Gilbart VL, Simms I, Gobin M, Jenkins C, Oliver I, Hughes G. High-risk drug practises associated with shigella flexneri serotype 3A infections amongst men who have sex with men (MSM) in England. Sexually Transmitted Infections Conference: STI and AIDS World Congress 2013; 89(SUPPL. 1).
272. Gill H, Portman M, Schoeman S. 2013 RITA data in a UK city centre GUM clinic – a valuable tool in guiding local services. HIV Medicine 2014; 15(SUPPL. 3): 17-159.
273. Gillespie T, McKay I. Survey of the use of selective culture for Neisseria gonorrhoeae in specimens from the female genital tract sent by general practitioners to a microbiology laboratory. Br J Gen Pract 2000; 50(451): 129-30.
274. Giomi B, Silvestri C, Bravi S, et al. Epidemiological and clinical characteristics of patients attending STI clinics in Tuscany, Italy: a multicenter report on new infections in 2011. G Ital Dermatol Venereol 2015; 150(2): 135-41.
275. Gnarpe H, Hillstrom L, Pettersson L. [Culture problems in the diagnosis of gonorrhea--study of a gonorrhea epidemic in Gavleborg county]. Lakartidningen 1976; 73(48): 4209-10.
276. Gomez-Ayerbe C, Palacios R, Perez C, et al. Treatment of acute HCV infection with direct acting antivirals (DAA) in HIV patients. HIV Medicine 2019; 20(S9): 317-37.
277. Gonzalez V, Alvarez-Dardet C, Rodriguez Pichardo A, Camacho F, Perea EJ. [Gonococcal infection in the male]. Med Clin (Barc) 1983; 81(20): 883-6.
278. Gottesman T, Yossepowitch O, Samra Z, Rosenberg S, Dan M. Prevalence of Mycoplasma genitalium in men with urethritis and in high risk asymptomatic males in Tel Aviv: a prospective study. Int J STD AIDS 2017; 28(2): 127-32.
279. Gotz HM, Luijendijk A, Nuradini B, Van Westreenen M. Women friendly gonorrhoea culture by self taken vaginal swabs for the purpose of surveillance of gonorrhoea resistance. Sexually Transmitted Infections 2011; 1): A282.
280. Götz HM, van Oeffelen LA, Hoebe C, van Benthem BH. Regional differences in chlamydia and gonorrhoeae positivity rate among heterosexual STI clinic visitors in the Netherlands: contribution of client and regional characteristics as assessed by cross-sectional surveillance data. BMJ Open 2019; 9(1): e022793.
281. Gotz HM, Veldhuijzen IK, Ossewaarde JM, de Zwart O, Richardus JH. Chlamydia trachomatis infections in multi-ethnic urban youth: a pilot combining STI health education and outreach testing in Rotterdam, Netherlands. Sex Transm Infect 2006; 82(2): 148-52; discussion 52-3.
282. Grad AI, Vica ML, Ungureanu L, Siserman CV, Tataru AD, Matei HV. Assessment of STI screening in Romania using a multiplex PCR technique. J Infect Dev Ctries 2020; 14(4): 341-8.
283. Granitzka S, Nickel C, Schramm K, Kremer P. [Symptomatology and changes in frequency of gonorrhea in gynecologic-obstetrical patients (10-years-study 1968-1977)]. Arch Gynecol 1979; 228(1-4): 614-5.
284. Graspeuntner S, Bohlmann MK, Gillmann K, et al. Microbiota-based analysis reveals specific bacterial traits and a novel strategy for the diagnosis of infectious infertility. PLoS ONE 2018; 13(1) (no pagination).
285. Grech P, Marchant R, Samuel M. Health care needs of women aged 40 and over attending an inner city integrated sexual health clinic. Sexually Transmitted Infections 2016; 92(Supplement 1): A34-A5.
286. Gregersen E. [Incidence of gonorrhea among women admitted to hospitals]. Ugeskr Laeger 1972; 134(43): 2268-70.
287. Gronhoj C, Jakobsen KK, Wingstrand VL, et al. Association between head and neck cancer and sexually transmitted diseases: a Danish nationwide, case-control study. Acta Otolaryngol 2020; 140(7): 615-9.
288. Groom TM, Nandwani R. Characteristics of men who pay for sex: a UK sexual health clinic survey. Sex Transm Infect 2006; 82(5): 364-7.
289. Guillet-Rosso F, Fari A, Taylor S, et al. Systematic semen culture and its influence on IVF management. Br J Obstet Gynaecol 1987; 94(6): 543-7.
290. Guralp O, Bostanci A, Basaran EO, Schild-Suhren M, Kaya B. Evaluation of the prevalence of sexually transmitted bacterial pathogens in northern cyprus by nucleic acid amplification tests, and investigation of the relationship between these pathogens and cervicitis. Turkish Journal of Obstetrics and Gynecology 2019; 16(4): 242-8.
291. Guschin A, Ryzhikh P, Rumyantseva T, Gomberg M, Unemo M. Treatment efficacy, treatment failures and selection of macrolide resistance in patients with high load of Mycoplasma genitalium during treatment of male urethritis with josamycin. BMC Infect Dis 2015; 15(1): 40.
292. Gyarmathy VA, Neaigus A, Ujhelyi E. Vulnerability to drug-related infections and co-infections among injecting drug users in Budapest, Hungary. Eur J Public Health 2009; 19(3): 260-5.
293. Hagemann CT, Nordbo SA, Myhre AK, Ormstad K, Schei B. Sexually transmitted infections among women attending a Norwegian Sexual Assault Centre. Sex Transm Infect 2014; 90(4): 283-9.
294. Haidari G, Perry MEO, White JA. P3.121 Are We Seeing a True Rise inNeisseria GonorrhoeaeandChlamydia Trachomatisin England in Men Who Have Sex with Men?: Abstract P3.121 Table 1. Sexually Transmitted Infections 2013; 89(Suppl 1): A185.2-A.
295. Hakama M, Lehtinen M, Knekt P, et al. Serum antibodies and subsequent cervical neoplasms: a prospective study with 12 years of follow-up. Am J Epidemiol 1993; 137(2): 166-70.
296. Halvorsen LE, Skjeldestad FE, Nordbo SA, Loge I. Which women should be tested for Chlamydia trachomatis in general practice? Tidsskrift for den Norske laegeforening 1992; 112(16): 2080-1.
297. Handy P, Pattman RS, Richards J. 'I'm OK?' Evaluation of a new walk-in quick-check clinic. Int J STD AIDS 2006; 17(10): 677-80.
298. Hanna NF, Taylor-Robinson D, Csonka GW, Harris JR, Al-Sowaygh IA. One-step staining of Neisseria gonorrhoeae in urethral discharge by methyl green-pyronin. Br J Vener Dis 1980; 56(4): 227-9.
299. Hansen JG, Schmidt H, Bitsch N. [Occurrence of Chlamydia trachomatis in vaginal discharges. A study from general practice]. Ugeskr Laeger 1983; 145(50): 3896-9.
300. Hansen MK, Lange K. [Incidence of gonorrhoea in women referred to a gynecological department. Routine study with reference to asymptomatic cases]. Ugeskr Laeger 1973; 135(20): 914-6.
301. Harb AK, Mohammed H, Furegato M, Wayal S, Mercer CH, Hughes G. The association between region of birth and sexually transmitted infections among people of black Caribbean ethnicity attending sexual health services in England, 2015. PLoS ONE 2020; 15(2) (no pagination).
302. Harding-Esch EM, Fuller SS, Chow SLC, et al. Diagnostic accuracy of a prototype rapid chlamydia and gonorrhoea recombinase polymerase amplification assay: a multicentre cross-sectional preclinical evaluation. Clinical Microbiology and Infection 2019; 25(3): 380.e1-.e7.
303. Harding-Esch EM, Nori AV, Hegazi A, et al. Impact of deploying multiple point-of-care tests with a 'sample first' approach on a sexual health clinical care pathway. A service evaluation. Sex Transm Infect 2017; 93(6): 424-9.

304. Harindra V, Tobin JM, Underhill G. Opportunistic chlamydia screening; should positive patients be screened for co-infections? Int J STD AIDS 2002; 13(12): 821-5.
305. Hassan SJ, Dunphy E, Navin E, et al. Screening for Chlamydia is acceptable and feasible during Cervical Screening in General Practice. Ir Med J 2016; 109(1): 326-7.
306. Hatton S, Snell W, Sacks R, Nugent D. Performance of microscopy to diagnose rectal gonorrhoea. HIV Medicine 2021; 22(S2): 24-119.
307. Hay PE, Kerry SR, Normansell R, et al. Which sexually active young female students are most at risk of pelvic inflammatory disease? A prospective study. Sex Transm Infect 2016; 92(1): 63-6.
308. Heijman RLJ, Stolte IG, Thiesbrummel HFJ, et al. Opting out increases HIV testing in a large sexually transmitted infections outpatient clinic. Sexually Transmitted Infections 2009; 85(4): 249-55.
309. Heijman TL, Van der Bij AK, De Vries HJ, Van Leent EJ, Thiesbrummel HF, Fennema HS. Effectiveness of a risk-based visitor-prioritizing system at a sexually transmitted infection outpatient clinic. Sex Transm Dis 2007; 34(7): 508-12.
310. Heijne JCM, van den Broek IVF, Bruisten SM, van Bergen JEA, de Graaf H, van Benthem BHB. National prevalence estimates of chlamydia and gonorrhoea in the Netherlands. Sex Transm Infect 2019; 95(1): 53-9.
311. Heiligenberg M, Rijnders B, Schim van der Loeff MF, et al. High prevalence of sexually transmitted infections in HIV-infected men during routine outpatient visits in the Netherlands. Sex Transm Dis 2012; 39(1): 8-15.
312. Heiligenberg M, Wermeling P, van Rooijen M, et al. P1-S5.13 Recreational drug use during sex is associated with STI among clients of a large STI outpatient clinic in Amsterdam, The Netherlands. Sexually Transmitted Infections 2011; 87(Suppl 1): A179-A.
313. Heinonen PK, Leinonen M. Fecundity and morbidity following acute pelvic inflammatory disease treated with doxycycline and metronidazole. Arch Gynecol Obstet 2003; 268(4): 284-8.
314. Hellberg D, Mogilevkina I, Mardh PA. Sexually transmitted diseases and gynecologic symptoms and signs in women with a history of induced abortion. Sex Transm Dis 1999; 26(4): 197-200.
315. Hellgren L, Johannisson G, Lofmarck PW. A comparison of diagnostic reliability between smear and culture tests for female gonorrhoea. Microbios Letters 1982; 20(79-80): 125-8.
316. Henriques CU, Wilken-Jensen C, Thorsen P, Moller BR. A randomised controlled trial of prophylaxis of post-abortal infection: ceftriaxone versus placebo. Br J Obstet Gynaecol 1994; 101(7): 610-4.
317. Henry-Suchet J, Catalan F, Loffredo V, et al. Microbiology of specimens obtained by laparoscopy from controls and from patients with pelvic inflammatory disease or infertility with tubal obstruction: Chlamydia trachomatis and Ureaplasma urealyticum. Am J Obstet Gynecol 1980; 138(7 Pt 2): 1022-5.
318. Henry-Suchet J, Solal P, Loffredo V, Pez JP. [Subclinical perihepatitis (Fitz-Hugh-Curtis syndrome) in salpingitis or tubal sterility. Cultures positive for Chlamydia trachomatis in perihepatic adhesions]. Presse Med 1983; 12(27): 1725.
319. Heras E, Llibre JM, Martro E, Casabona J, Martin-Iguacel R, Sirera G. [Lymphogranuloma venereum proctocolitis in men with HIV-1 infection]. Enferm Infecc Microbiol Clin 2011; 29(2): 124-6.
320. Hermabessiere J, Sirot J, Boucher D, Cluzel R. [Clinical and bacteriological criteria of genitaltract infections in male sterility (author's transl)]. Rev Fr Gynecol Obstet 1977; 72(10): 653-6, 7-8.
321. Herman A, Roash Z, Samra Z. Prevalence of low genital tract infectious agents in young Israeli women. Israel Journal of Medical Sciences 1985; 21(4): 346-50.
322. Hetem DJ, Kuizenga Wessel S, Bruisten SM, et al. High prevalence and resistance rates of Mycoplasma genitalium among patients visiting two sexually transmitted infection clinics in the Netherlands. Int J STD AIDS 2021; 32(9): 837-44.
323. Heymans R, A AM, Bruisten SM, et al. Distinct Neisseria gonorrhoeae transmission networks among men who have sex with men in Amsterdam, The Netherlands. J Infect Dis 2012; 206(4): 596-605.
324. Higgins SP, Klapper PE, Struthers JK, et al. Detection of male genital infection with Chlamydia trachom atis and Neisseria gonorrhoeae using an automated multiplex PCR system (Cobas AmplicorTM). International Journal of STD & AIDS 2016; 9(1): 21-4.
325. Hill-Tout R, Harding-Esch EM, Pacho A, Furegato M, Fuller SS, Sadiq ST. Health-related quality of life and psychosocial impacts of a diagnosis of non-specific genital infection in symptomatic heterosexual men attending UK sexual health clinics: a feasibility study. BMJ Open 2018; 8(6): e018213.
326. Hilmarsdottir I, Arnardottir EM, Johannesdottir ER, Golparian D, Unemo M. Chlamydia trachomatis in Iceland: Prevalence, Clinico-epidemiological Features and Comparison of Cobas 480 CT/NG and Aptima Combo 2 (CT/NG) for Diagnosis. Acta Derm Venereol 2021; 101(2): adv00393.
327. Hilton AL, Richmond SJ, Milne JD, Hindley F, Clarke SK. Chlamydia A in the female genital tract. Br J Vener Dis 1974; 50(1): 1-10.
328. Hiltunen-Back E, Kautiainen H. Increasing sexually transmitted infection rates among men who have sex with men in Finland 2004-2014. International Journal of STD & AIDS 2015; 26(11_suppl): 1-121.
329. Hiltunen-Back E, Rostila T, Kautiainen H, Paavonen J, Reunala T. Rapid decrease of endemic gonorrhea in Finland. Sex Transm Dis 1998; 25(4): 181-6.
330. Hinton RA, Egdell LM, Andrews BE, Clarke SK, Richmond SJ. A double-blind cross-over study of the effect of doxycycline on mycoplasma infection and infertility. Br J Obstet Gynaecol 1979; 86(5): 379-83.
331. Hirst G, Richardson D, Soni S. Non-specific urethritis: Can we be a little more specific? Sexually Transmitted Infections 2016; 92(Supplement 1): A19.
332. Hjelmevoll SO, Olsen ME, Sollid JU, et al. Appropriate time for test-of-cure when diagnosing gonorrhoea with a nucleic acid amplification test. Acta Derm Venereol 2012; 92(3): 316-9.
333. Hodoglugil NN, Aslan D, Bertan M. Intrauterine device use and some issues related to sexually transmitted disease screening and occurrence. Contraception 2000; 61(6): 359-64.
334. Hodoglugil NNS, Ozek B, Bertan M. Prevalence of reproductive tract infections in family planning clients in Trabzon, Turkey. Sexually Transmitted Infections 1999; 75(5): 360.
335. Hoebe CJ, Rademaker CW, Brouwers EE, ter Waarbeek HL, van Bergen JE. Acceptability of self-taken vaginal swabs and first-catch urine samples for the diagnosis of urogenital Chlamydia trachomatis and Neisseria gonorrhoeae with an amplified DNA assay in young women attending a public health sexually transmitted disease clinic. Sex Transm Dis 2006; 33(8): 491-5.
336. Hoenderboom BM, van Benthem BHB, van Bergen J, et al. Relation between Chlamydia trachomatis infection and pelvic inflammatory disease, ectopic pregnancy and tubal factor infertility in a Dutch cohort of women previously tested for chlamydia in a chlamydia screening trial. Sex Transm Infect 2019; 95(4): 300-6.
337. Hofmann H, Petzoldt D. [Detection of the gonococcal antigen with an enzyme immunoassay (Gonozyme). Results of the original and modified test procedure]. Hautarzt 1985; 36(12): 675-81.
338. Hofmann H, Petzoldt D, Kunze-Janssenharms F, et al. [Improvement of the diagnosis of gonorrhea in the advisory board of the Public Health Department]. Offentl Gesundheitswes 1984; 46(1): 8-10.
339. Homøe AS, Berntsen S, Pedersen ML. Gonorrhoea in Greenland: geographic differences in diagnostic activity and incidence of gonorrhoea in 2015. Int J Circumpolar Health 2018; 77(1): 1445938.

340. Hoornenborg E, Coyer L, Achterbergh RCA, et al. Sexual behaviour and incidence of HIV and sexually transmitted infections among men who have sex with men using daily and event-driven pre-exposure prophylaxis in AMPrEP: 2 year results from a demonstration study. The Lancet HIV 2019; 6(7): e447-e55.
341. Hopkins MJ, Smith G, Hart IJ, Alloba F. Screening tests for Chlamydia trachomatis or Neisseria gonorrhoeae using the cobas 4800 PCR system do not require a second test to confirm: an audit of patients issued with equivocal results at a sexual health clinic in the Northwest of England, U.K. Sex Transm Infect 2012; 88(7): 495-7.
342. Hovding G, Bjerke JR. [The gonorrhea problem. A 1-year statistics from the Bergen Health Council Ambulatory Clinic for Venereal Diseases]. Tidsskr Nor Laegeforen 1977; 97(12): 543-51.
343. Hoverak A, Jagars GA, Casingo PA. [Occurrence of Chlamydia trachomatis and Neisseria gonorrhoeae among asymptomatic soldiers]. Tidsskr Nor Laegeforen 1989; 109(13): 1429.
344. Hovhannisyan G, von Schoen-Angerer T, Babayan K, Fenichiu O, Gaboulaud V. Antimicrobial susceptibility of Neisseria gonorrheae strains in three regions of Armenia. Sex Transm Dis 2007; 34(9): 686-8.
345. Hoyos-Mallecot Y, Garcia JN, Sulleiro E, et al. Drassanes Expres: a public and confidential testing service for asymptomatic STIs with same-day result notification. Sex Transm Infect 2021.
346. Hughes G, Catchpole M, Rogers PA, et al. Comparison of risk factors for four sexually transmitted infections: results from a study of attenders at three genitourinary medicine clinics in England. Sex Transm Infect 2000; 76(4): 262-7.
347. Hunter JM, Neilson M. Sexually transmitted diseases in Edinburgh: patients under 18 years of age. Health Bull (Edinb) 1980; 38(1): 23-8.
348. Hunter JM, Smith IW, Peutherer JF, MacAulay A, Tuach S, Young H. Chlamydia trachomatis and Ureaplasma urealyticum in men attending a sexually transmitted diseases clinic. Br J Vener Dis 1981; 57(2): 130-3.
349. Hunter JM, Smith IW, Peutherer JF, MacAulay AJ. Chlamydia trachomatis infection of the cervix: the need for a diagnostic service. Scott Med J 1982; 27(2): 147-51.
350. Hussey J, Edirisinghe DN, Pattman RS, et al. Does bacterial vaginosis alter the sensitivity of screening tests for Chlamydia trachomatis? An analysis of patient characteristics. Int J STD AIDS 2003; 14(7): 448-50.
351. Igoe D, Kelleher M, Cooney F, et al. There has been a true rise in Neisseria gonorrhoeae but not in Chlamydia trachomatis in men who have sex with men in Dublin, Ireland. Sex Transm Infect 2014; 90(7): 523.
352. Ivens D, Macdonald K, Bansi L, Nori A. Screening for rectal chlamydia infection in a genitourinary medicine clinic. Int J STD AIDS 2007; 18(6): 404-6.
353. Jackson L, Thorley N, Munetsi L, Ross J. Evaluating online and clinic-based STI screening services: A case study of umbrella sexual health services, UK. Sexually Transmitted Infections 2019; 95(Supplement 1): A112-A3.
354. Jacob L, Bohler F, Kalder M, Kostev K. Prevalence and treatment of sexually transmitted diseases in gynecological practices in Germany: A retrospective study with more than 1,000,000 patients. Int J Clin Pharmacol Ther 2018b; 56(5): 212-6.
355. Jacob L, Duse DA, Kostev K. Prevalence and treatment of sexually transmitted infections in men followed by urologists in Germany - a cross sectional study with 347,090 men. Ger Med Sci 2018a; 16: Doc03.
356. Jacobson L, Westrom L. Objectivized diagnosis of acute pelvic inflammatory disease. Diagnostic and prognostic value of routine laparoscopy. Am J Obstet Gynecol 1969; 105(7): 1088-98.
357. Jamalova M. Tajikistan: STD survey results. Entre Nous Cph Den 1999; (42): 12.
358. Janier M, Lassau F, Casin I, et al. Male urethritis with and without discharge: a clinical and microbiological study. Sex Transm Dis 1995; 22(4): 244-52.
359. Janier M, Ramel F, Lajoie C, et al. Asymptomatic urethral carriage of Chlamydia trachomatis in male patients with genital ulcerations in Paris, France [1]. Sexually Transmitted Diseases 1990; 17(3): 156-7.
360. Jansen K, Bremer V, Steffen G, et al. Prevalence of genital infections with chlamydia trachomatis (CT), neisseria gonorrhoea (NG) and Trichomonas Vaginalis (TV) in Hard-To-Reach Female Sex Workers in North Rhine-Westphalia, Germany: The STI-Outreach Study. Sexually Transmitted Infections Conference: STI and AIDS World Congress 2013; 89(SUPPL. 1).
361. Jansen K, Steffen G, Potthoff A, et al. STI in times of PrEP: high prevalence of chlamydia, gonorrhea, and mycoplasma at different anatomic sites in men who have sex with men in Germany. BMC Infect Dis 2020; 20(1): 110.
362. Jauréguy F, Chariot P, Vessières A, Picard B. Prevalence of Chlamydia trachomatis and Neisseria gonorrhoeae infections detected by real-time PCR among individuals reporting sexual assaults in the Paris, France area. Forensic Sci Int 2016; 266: 130-3.
363. Jaureguy F, Masson C, Lavisse F, Larmignat P, Picard B. Interest of the cepheid xpert CT/NG assay to rapid detection and differentiation of chlamydia trachomatis (CT) and Neisseria Gonorrhoeae (NG) uro-genital infections. Sexually Transmitted Infections Conference: STI and AIDS World Congress 2013; 89(SUPPL. 1).
364. Jeffery A, Nielson A. Online innovation to reach men who have sex with men. HIV Medicine 2018; 19(Supplement 2): S105-S6.
365. Jenniskens ML, Veerbeek JH, Deurloo KL, van Hannen EJ, Thijsen SF. Routine testing of Mycoplasma genitalium and Trichomonas vaginalis. Infect Dis (Lond) 2017; 49(6): 461-5.
366. Jensen NE, Andersen BL. The prevalence of group B streptococci in human urogenital secretions. Scand J Infect Dis 1979; 11(3): 199-202.
367. Jeverica S, Unemo M, Mlakar B, et al. Prevalence of Neisseria Gonorrhoeae infection in two distinct men-who-have-sex-with-men (MSM) populations in Slovenia in 2012. Sexually Transmitted Infections Conference: STI and AIDS World Congress 2013; 89(SUPPL. 1).
368. Jiménez E, Pedrazuela MG, Pérez MM, de Mosteyrín SF, Arrieta JJ, Guerrero ML. Prevalence of pharyngeal infection by Neisseria gonorrhoeae among human immunodeficiency virus-positive men who have sex with men in downtown Madrid, 2011. Int J STD AIDS 2013; 24(11): 875-8.
369. Joffe H, Bamberger E, Nurkin S, et al. Sexually transmitted diseases among patients with human immunodeficiency virus in northern Israel. Isr Med Assoc J 2006; 8(5): 333-6.
370. Johansson E, Forslin L, Moi H, Danielsson D. [Occurrence of gonorrhea, Chlamydia infection and urethritis in permanent partners of women with acute salpingitis]. Lakartidningen 1988; 85(37): 2974-6.
371. Joly-Guillou ML, Judlin P, Lefevre JC, et al. [Bacteria isolated in 1994-1995 in female upper genitalia infections and in male urethritis. Distribution and sensitivity to antibiotics]. Presse Med 1996; 25(8): 342-8.
372. Joore IK, Twisk DE, Vanrolleghem AM, et al. The need to scale up HIV indicator condition-guided testing for early case-finding: a case-control study in primary care. BMC Fam Pract 2016; 17(1): 161.
373. Jungmann E, Johnson AM, Ridgway G, Durrant K, Robinson AJ. How useful are high vaginal swabs in general practice? Results of a multicentre study. Int J STD AIDS 2004; 15(4): 238-9.
374. Kabakchieva E, Vassileva S, Kelly JA, et al. HIV risk behavior patterns, predictors, and sexually transmitted disease prevalence in the social networks of young Roma (Gypsy) men in Sofia, Bulgaria. Sex Transm Dis 2006; 33(8): 485-90.
375. Kacena KA, Dohnal K, Benesova V, et al. Chlamydia, gonorrhea, and HIV-1 prevalence among five populations of women in the Czech and Slovak Republics. Sex Transm Dis 2001; 28(6): 356-62.
376. Kahn JA, Larssen KE, Svarva PL. [Gonorrhea in pregnant women. Hospital case material]. Tidsskr Nor Laegeforen 1984; 104(22): 1396-7.

377. Kamarashev J, Riess CE, Mosimann J, Lauchlf S. Lymphogranuloma venereum in Zurich, Switzerland: Chlamydia trachomatis serovar L2 proctitis among men who have sex with men. Swiss Medical Weekly 2010; 140(13-14): 209-12.
378. Kamwendo F, Forslin L, Bodin L, Danielsson D. Decreasing incidences of gonorrhea- and chlamydia-associated acute pelvic inflammatory disease. A 25-year study from an urban area of central Sweden. Sex Transm Dis 1996; 23(5): 384-91.
379. Kamwendo F, Forslin L, Danielsson D. Epidemiology and aetiology of acute non-tuberculous salpingitis. A comparison between the early 1970s and the early 1980s with special reference to gonorrhoea and use of intrauterine contraceptive device. Genitourin Med 1990; 66(5): 324-9.
380. Kamwendo F, Johansson E, Moi H, Forslin L, Danielsson D. Gonorrhea, genital chlamydial infection, and nonspecific urethritis in male partners of women hospitalized and treated for acute pelvic inflammatory disease. Sex Transm Dis 1993; 20(3): 143-6.
381. Kanbur S. Comparison of screening and treatment protocols of sexually transmitted diseases. [Turkish]. Zeynep Kamil Tip Bulteni 2018; 49(1): 15-20.
382. Karp-Damdin M. [Incidence of trichomonal and trichomonal-gonococcal infections in women examined at the Provincial Clinic for Dermatologic and Venereal Diseases in Rzeszow]. Przegl Dermatol 1975; 62(2): 215-7.
383. Karp-Damdin M, Wisniewski R. [Extensiveness of infection with Trichomonas vaginalis, yeast-like fungi and Neisseria gonorrhoeae in women in the city of Rzeszow and its vicinity]. Wiad Parazytol 1987; 33(1): 47-51.
384. Kavli G, Saetrom KM, Gundersen T, Volden G. Higher incidence of asymptomatic gonorrhoea in men with initial infection than with reinfection. Br J Vener Dis 1984; 60(4): 274.
385. Keaveney S, Sadlier C, O'Dea S, Delamere S, Bergin C. High prevalence of asymptomatic sexually transmitted infections in HIV-infected men who have sex with men: a stimulus to improve screening. Int J STD AIDS 2014; 25(10): 758-61.
386. Kelly C, Johnston J, Carey F. Evaluation of a partnership between primary and secondary care providing an accessible Level 1 sexual health service in the community. Int J STD AIDS 2014; 25(10): 751-7.
387. Kenyon C, Baetselier I, Wouters K. Screening for STIs in PrEP cohorts results in high levels of antimicrobial consumption. Int J STD AIDS 2020; 31(12): 1215-8.
388. Kerry-Barnard S, Beddows S, Reid F, et al. Human papillomavirus (HPV) vaccination and oropharyngeal HPV in ethnically diverse, sexually active adolescents: community-based cross-sectional study. Sexually transmitted infections 2020; 03.
389. Khatib N, Bradbury C, Chalker V, et al. Prevalence of Trichomonas vaginalis, Mycoplasma genitalium and Ureaplasma urealyticum in men with urethritis attending an urban sexual health clinic. Int J STD AIDS 2015; 26(6): 388-92.
390. Killick P, Goold P. Audit of sexually transmitted infection (STI) screening in HIV-positive pregnant women. HIV Medicine 2019; 20(S5): 16-75.
391. Killick V, Kell P, Plumb H, Hurd N, Turner P. Screening for Neisseria gonorrhoeae using the BD ProbeTec nucleic acid amplification test in a low prevalence genitourinary medicine clinic. Int J STD AIDS 2012; 23(6): e1-3.
392. Kimmitt PT, Tabrizi SN, Crosatti M, et al. Pilot study of the utility and acceptability of tampon sampling for the diagnosis of Neisseria gonorrhoeae and Chlamydia trachomatis infections by duplex realtime polymerase chain reaction in United Kingdom sex workers. Int J STD AIDS 2010; 21(4): 279-82.
393. King GP, Evans DT. Behind the camera: Sexual health testing patterns and outcomes amongst UK adult film performers. Int J STD AIDS 2020; 31(1): 62-5.
394. Kinghorn GR. Survey of human immunodeficiency virus infection and sexually transmitted diseases in homosexual and bisexual men attending genitourinary medicine clinics in the UK during 1986-88. Genitourinary Medicine 1990; 66(5): 387-92.
395. Kinghorn GR. Gonorrhoea in homosexual and bisexual men. Int J STD AIDS 1994; 5(5): 379-80.
396. Kinghorn GR, Waugh MA. Oral contraceptive use and prevalence of infection with Chlamydia trachomatis in women. Br J Vener Dis 1981; 57(3): 187-90.
397. Kjaer SK. Risk factors for cervical neoplasia in Denmark. APMIS Suppl 1998; 80(80): 1-41.
398. Kjaergaard N, Kristensen B, Hansen ES, et al. Microbiology of semen specimens from males attending a fertility clinic. APMIS : acta pathologica, microbiologica, et immunologica Scandinavica 1997; 105(7): 566-70.
399. Klanica J, Drasnar J, Stejskalova M. [The incidence of chronic gonorrhoea in sterile women (author's transl)]. Cesk Gynekol 1978; 43(1): 21-6.
400. Klanica J, Stejskalová M. Direct immunofluorescent test for the detection of gonorrhoea. Br J Vener Dis 1976; 52(1): 33-5.
401. Klavs I, Milavec M, Kustec T, et al. Prevalence of chlamydia, Gonorrhoea, M. Genitalium and T. Vaginalis in the general population of Slovenia, 2016-2017. Sexually Transmitted Infections 2019; 95(Supplement 1): A187.
402. Klingenberg RE, Mannherz S, Brockmeyer NH, et al. [Local health study : Outreach medical services for female sex workers in Bochum]. Hautarzt 2016; 67(12): 989-95.
403. Knapper CM, Temple M, Roderick J, Smith J, Birley HDL. Audit of a network of sexually transmitted infections centred in South Wales. International Journal of STD and AIDS 2010; 21(1): 63-5.
404. Knudsen A, Kjaergaard B, Ejlertsen T. [Chlamydia trachomatis in at-risk groups in legal abortion in the first trimester]. Ugeskr Laeger 1989; 151(47): 3157-9.
405. Knudsen O, Larsen LH, Worm AM. [Simultaneous occurrence of Trichomonas vaginalis and gonorrhea in women]. Ugeskr Laeger 1985; 147(21): 1691-2.
406. Koch A, Bilina A, Teodorowicz L, Stary A. Mycoplasma hominis and Ureaplasma urealyticum in patients with sexually transmitted diseases. Wien Klin Wochenschr 1997; 109(14-15): 584-9.
407. Koedijk FD, van Benthem BH, Vrolings EM, Zuilhof W, van der Sande MA. Increasing sexually transmitted infection rates in young men having sex with men in the Netherlands, 2006-2012. Emerg Themes Epidemiol 2014; 11(1): 12.
408. Koedijk FD, van Bergen JE, Dukers-Muijrers NH, van Leeuwen AP, Hoebe CJ, van der Sande MA. The value of testing multiple anatomic sites for gonorrhoea and chlamydia in sexually transmitted infection centres in the Netherlands, 2006-2010. Int J STD AIDS 2012; 23(9): 626-31.
409. Koedijk FDH, Van Benthem BH, Vrolings EMDC, et al. Increasing STI rates in Young MSM in the Netherlands, 2006-2011. Sexually Transmitted Infections Conference: STI and AIDS World Congress 2013; 89(SUPPL. 1).
410. Kogoj R, Pervic S, Bernjak SI, Murnik Rauh A, Maticic M, Kee D. Epidemiology of Chlamydia trachomatis, Neisseria gonorrhoeae, and urogenital mycoplasma infections in central Slovenia. Clinical Chemistry and Laboratory Medicine 2018; 56(6): eA114.
411. Kolader ME, Dukers NH, van der Bij AK, et al. Molecular epidemiology of Neisseria gonorrhoeae in Amsterdam, The Netherlands, shows distinct heterosexual and homosexual networks. J Clin Microbiol 2006; 44(8): 2689-97.
412. Kolmorgen K, Seidenschnur G, Arab MT. [Second-look laparoscopy--results and pregnancy rate in patients with a state of laparoscopically assessed acute adnexitis]. Geburtshilfe Frauenheilkd 1987; 47(10): 724-8.
413. KOSKIMIES AI, PAAVONEN J, MEYER B, KAJANOJA P. Cervicitis and Infertility. American Journal of Reproductive Immunology 1981; 1(6): 299-302.
414. Kostyakova E, Korolev S, Andreeva I, et al. DIAGNOSTIC AND TREATMENT PATTERNS FOR ACUTE MALE URETHRITIS IN 4 RUSSIAN CITIES: P269. Basic & Clinical Pharmacology & Toxicology 2011; 109(SUPPL. 1): 56-164.
415. Kourbatova EV, Akovbyan VA, Chesson HW, et al. Assessment of the routine, occupation-based gonorrhea and syphilis screening program in Moscow, Russia: an analysis of sexually transmitted infection prevalence and cost-effectiveness. Sex Transm Dis 2008; 35(5): 453-60.

416. Krolov K, Frolova J, Tudoran O, et al. Sensitive and rapid detection of Chlamydia trachomatis by recombinase polymerase amplification directly from urine samples. J Mol Diagn 2014; 16(1): 127-35.
417. Kuklova I, Resl V, Cerna L, et al. International healthcare projects of sexually transmitted infections prevention in prostitutes in the Czech Republic. Prakticky Lekar 2005; 85(9): 490-2.
418. Kurova T, Shoubnikova M, Malceva A, Mårdh PA. Prostitution in Riga, Latvia--a socio-medical matter of concern. Acta Obstet Gynecol Scand 1998; 77(1): 83-6.
419. Lacey HB. Sexually transmitted diseases and rape: the experience of a sexual assault centre. Int J STD AIDS 1990; 1(6): 405-9.
420. Lamb AM. New methods of contact tracing in infectious venereal diseases. Br J Vener Dis 1966; 42(4): 276-9.
421. Lampejo T, Abdulcadir M. P093 Audit on the management of epididymo-orchitis in a London-based level 3 sexual health clinic: Abstract P093 Table 1. Sexually Transmitted Infections 2016; 92(Suppl 1): A51.1-A.
422. Lampejo T, Turner R, Roberts C, et al. Novel outreach settings to enhance sexually transmitted infection/HIV awareness, diagnosis and treatment in hard-to-reach populations. Int J STD AIDS 2018; 29(3): 266-72.
423. Landolt A, Kunz J, Schreiner WE. [Routine gonorrhoeae cultures: a cost--benefit analysis on obstetric--gynaecological patients (author's transl)]. Geburtshilfe Frauenheilkd 1978; 38(4): 255-9.
424. Larsson PG, Platz-Christensen JJ, Thejls H, Forsum U, Pahlson C. Incidence of pelvic inflammatory disease after first-trimester legal abortion in women with bacterial vaginosis after treatment with metronidazole: a double-blind, randomized study. Am J Obstet Gynecol 1992; 166(1 Pt 1): 100-3.
425. Latini A, Dona MG, Alei L, et al. Recreational drugs and STI diagnoses among patients attending an STI/HIV reference clinic in Rome, Italy. Sexually Transmitted Infections 2019; 95(8): 588-93.
426. Latino MA, Lanza A, Bello L, et al. [Cervico-vaginal infections. Study of a population in the Turin area]. Minerva Ginecol 2002; 54(4): 309-16.
427. Lavelle SJ, Jones KE, Mallinson H, Webb AM. Finding, confirming, and managing gonorrhoea in a population screened for chlamydia using the Gen-Probe Aptima Combo2 assay. Sex Transm Infect 2006; 82(3): 221-4.
428. Lawrence D, Vera J, Richardson D, Gilbert D, Marchant R, Morgan C. Anal intraepithelial neoplasia (AIN) in a cohort of HIVpositive individuals: A retrospective data analysis. HIV Medicine 2017; 18(Supplement 1): 33.
429. Lawton M, Schembri G, Kingston M. Testing for Trichomonas vaginalis (TV) by transcription mediated amplification (TMA). An evaluation in a large city clinic. Sexually Transmitted Infections Conference: 4th Joint BASHH ASTDA Meeting Brighton United Kingdom Conference Publication: 2012; 88(SUPPL. 1).
430. Le Faou AE, Bottlaender M, Tardieu JC. Incidence of Neisseria gonorrhoeae and Chlamydia trachomatis infections in Strasbourg over 8 years. Genitourin Med 1991; 67(5): 428-30.
431. Le Roy C, Le Hen I, Clerc M, et al. The first performance report for the Bio-Rad Dx CT/NG/MG assay for simultaneous detection of Chlamydia trachomatis, Neisseria gonorrhoeae and Mycoplasma genitalium in urogenital samples. J Microbiol Methods 2012; 89(3): 193-7.
432. Le Roy C, Peuchant O, Le Hen I, Bertrand-Salles I, Bebear C, De Barbeyrac B. Chlamydia trachomatis/Neisseria Gonorrhoeae screening in duplex: Should we verify N. gonorrhoeae positive results? Sexually Transmitted Infections Conference: STI and AIDS World Congress 2013; 89(SUPPL. 1).
433. Lee M, Hegazi A, Barbour A, et al. Chemsex and the city: Sexualised substance use in gay bisexual and other men who have sex with men. Sexually Transmitted Infections 2015; 1): A4.
434. Lefèvre JC, Averous S, Bauriaud R, Blanc C, Bertrand MA, Lareng MB. Lower genital tract infections in women: comparison of clinical and epidemiologic findings with microbiology. Sex Transm Dis 1988; 15(2): 110-3.
435. Lefevre JC, Lepargneur JP, Bauriaud R, Bertrand MA, Blanc C. Clinical and microbiologic features of urethritis in men in Toulouse, France. Sex Transm Dis 1991; 18(2): 76-9.
436. Lelijveld JL, Leentvaar-Kuijpers A, Hekker AC, Polak-Vogelzang AA, de Wit RF. [Sexually transmitted diseases in female clients of an outpatient clinic for venereal diseases in Utrecht]. Ned Tijdschr Geneeskd 1981; 125(12): 463-6.
437. Leung A, Eastick K, Haddon LE, Horn CK, Ahuja D, Horner PJ. Mycoplasma genitalium is associated with symptomatic urethritis. Int J STD AIDS 2006; 17(5): 285-8.
438. Leuridan E, Wouters K, Stalpaert M, Van Damme P. Male sex workers in Antwerp, Belgium: A descriptive study. International Journal of STD and AIDS 2005; 16(11): 744-8.
439. Levy I, Gefen-Halevi S, Nissan I, et al. Delayed diagnosis of colorectal sexually transmitted diseases due to their resemblance to inflammatory bowel diseases. Int J Infect Dis 2018; 75: 34-8.
440. Lewis DA, McDonald A, Thompson G, Bingham JS. The 374 clinic: an outreach sexual health clinic for young men. Sex Transm Infect 2004; 80(6): 480-3.
441. Libois A, Hallin M, Crucitti T, Delforge M, De Wit S. Prevalence of Mycoplasma genitalium in men with urethritis in a large hospital in Brussels, Belgium. International Journal of STD and AIDS 2015; 1): 22.
442. Lind I, Hoffmann S. Recorded gonorrhoea rates in Denmark, 1900-2010: the impact of clinical testing activity and laboratory diagnostic procedures. BMJ Open 2015; 5(11): e008013.
443. Linhart Y, Shohat T, Amitai Z, et al. Sexually transmitted infections among brothel-based sex workers in Tel-Aviv area, Israel: high prevalence of pharyngeal gonorrhoea. Int J STD AIDS 2008; 19(10): 656-9.
444. Lister P, Robinson A. STD in travellers presenting to a GUM clinic. Int J STD AIDS 1993; 4(1): 59-60.
445. Lomas DA, Natin D, Stockley RA, Shahmanesh M. Chemotactic activity of urethral secretions in men with urethritis and the effect of treatment. J Infect Dis 1993; 167(1): 233-6.
446. Lomholt G, Berg O. Gonorrhoea situation in South Greenland in the summer of 1964. Br J Vener Dis 1966; 42(1): 1-7.
447. López-Corbeto E, González V, Lugo R, Rivaya B, Casabona J, Matas L. Pooling of urine samples for molecular detection of Chlamydia trachomatis, Neisseria gonorrhoeae and Mycoplasma genitalium as a screening strategy among young adults in Catalonia. Enferm Infecc Microbiol Clin (Engl Ed) 2020; 38(2): 65-71.
448. Loubinoux J, Reglier-Poupet H, Collobert G, Billoet A, Tavares N, Poyart C. Detection of Chlamydia trachomatis, Neisseria gonorrhoeae, and Mycoplasma genitalium in uro-genital samples by the real-time Dx CT/NG/MGTM PCR assay. Clinical Microbiology and Infection 2012; 3): 509.
449. Lourtet Hascoet J, Dahoun M, Cohen M, et al. Clinical diagnostic and therapeutic aspects of 221 consecutive anorectal Chlamydia trachomatis and Neisseria gonorrhoeae sexually transmitted infections among men who have sex with men. Int J Infect Dis 2018; 71: 9-13.
450. Loveday C, Mercey D. The prevalence of human retroviral infections in female patients attending a central London sexually transmitted disease clinic: 1985-1990. Genitourin Med 1993; 69(1): 31-4.
451. Low N, Bertisch B, Hauser C, et al. Factors associated with antimicrobial resistant gonorrhoea infections in men who have sex with men: case-control study. Sexually Transmitted Infections 2017; 93(Supplement 2): A28.
452. Low N, Connell P, McKevitt C, et al. 'You can't tell by looking': pilot study of a community-based intervention to detect asymptomatic sexually transmitted infections. Int J STD AIDS 2003; 14(12): 830-4.
453. Lucisano A, Morandotti G, Marana R, et al. Chlamydial genital infections and laparoscopic findings in infertile women. Eur J Epidemiol 1992; 8(5): 645-9.

454. Lycke E, Löwhagen GB, Hallhagen G, Johannisson G, Ramstedt K. The risk of transmission of genital Chlamydia trachomatis infection is less than that of genital Neisseria gonorrhoeae infection. Sex Transm Dis 1980; 7(1): 6-10.
455. Macauley S, Creighton S. Testing commercial sex workers for chlamydia and gonorrhoea on outreach. Sex Transm Infect 2009; 85(3): 231-2.
456. MacDonald R. BASHH Virtual Annual Conference, 19th to 21st October 2020. International Journal of STD & AIDS 2020; 31(12_suppl): 1-114.
457. Madjar S, Nativ O, Barbara J, Tal J, Potasman I, Srugo I. [Diagnostic and therapeutic approach to sexually transmitted diseases]. Harefuah 1996; 130(12): 811-4, 80, 79.
458. Magnusson SS, Oskarsson T, Geirsson RT. Lower genital tract infection with Chlamydia trachomatis and Neisseria gonorrhoeae in Icelandic women with salpingitis. American Journal of Obstetrics and Gynecology 1986; 155(3): 602-7.
459. Mahto M, Evans-Jones J, Mallinson H. Response to 'Trichomonas vaginalis among multiethnic female UK students'. Sex Transm Infect 2011; 87(6): 537.
460. Mahto M, Zia S. Measuring the gap: from Home Office to the National Health Service in the provision of a one-stop shop sexual health service in a female prison in the UK. Int J STD AIDS 2008; 19(9): 586-9.
461. Mahto M, Zia S, Ritchie D, Mallinson H. Diagnosis, management and prevalence estimation of gonorrhoea: influences of Aptima Combo 2 assay with alternative target confirmation. Int J STD AIDS 2009; 20(5): 315-9.
462. Maini M, French P, Prince M, Bingham JS. Urethritis due to Neisseria meningitidis in a London genitourinary medicine clinic population. International Journal of STD and AIDS 1992; 3(6): 423-5.
463. Maleckiene L, Nadisauskiene RJ, Barcaite E. Laparoscopy improves the diagnostic accuracy in pelvic inflammatory disease. Salud(i)Ciencia 2012; 19(4): 326-9.
464. Malosso P, Badia L, Guardigni V, et al. Absence of hepatitis C infection despite high-risk sexual behavior in a cohort of men who have sex with men screened in the community: preliminary results of SEXCHECK study. Journal of Hepatology 2019; 70(1): 500-1.
465. Manavi K, Luo PL, McMillan A. The three-year positivity rate of sexually transmitted infections among a group of HIV-infected men attending the Department of Genitourinary Medicine, Edinburgh, UK. Int J STD AIDS 2005; 16(11): 730-2.
466. Manavi K, McMillan A, Young H. The prevalence of rectal chlamydial infection amongst men who have sex with men attending the genitourinary medicine clinic in Edinburgh. Int J STD AIDS 2004; 15(3): 162-4.
467. Marangoni A, Ceccarani C, Camboni T, et al. Pharyngeal microbiome alterations during Neisseria gonorrhoeae infection. PLoS One 2020; 15(1): e0227985.
468. Marangoni A, Foschi C, Nardini P, et al. Evaluation of Versant CT/GC DNA 1.0 assay for the detection of chlamydia trachomatis and neisseria gonorrhoeae in urine specimens in selected STD patients. Clinical Microbiology and Infection 2011; 4): S489.
469. Marangoni A, Foschi C, Tartari F, Gaspari V, Re MC. Lymphogranuloma venereum genovariants in men having sex with men in Italy. Sex Transm Infect 2021; 97(6): 441-5.
470. Marchant R, Morgan C, Gilbert D, Vera J, Richardson D, Lawrence D. Anal squamous cell carcinoma (SCC) in a large urban cohort of HIV-positive individuals living in the UK: A retrospective data analysis. HIV Medicine 2017; 18(Supplement 1): 33.
471. Marcone V, Recine N, Gallinelli C, et al. Epidemiology of Chlamydia trachomatis endocervical infection in a previously unscreened population in Rome, Italy, 2000 to 2009. Euro Surveill 2012; 17(25).
472. Marconi M, Pilatz A, Wagenlehner F, Diemer T, Weidner W. Are antisperm antibodies really associated with proven chronic inflammatory and infectious diseases of the male reproductive tract? Eur Urol 2009; 56(4): 708-15.
473. Marcos C, Bergamaschi L, Garcia P, Ubeda A, Fabra G. Prevalence of chlamydia trachomatis, gonorrhea and high-risk human papilloma virus in women older than 50 years old with abnormal cervical cytology. Maturitas 2019; 124: 174.
474. Marcus U, Ort J, Grenz M, Eckstein K, Wirtz K, Wille A. Risk factors for HIV and STI diagnosis in a community-based HIV/STI testing and counselling site for men having sex with men (MSM) in a large German city in 2011-2012. BMC Infect Dis 2015; 15: 14.
475. Mårdh PA, Lind I, From E, Andersen AL. Prevalence of Chlamydia trachomatis and Neisseria gonorrhoeae infections in Greenland. A seroepidemiological study. Br J Vener Dis 1980; 56(5): 327-31.
476. Märdh PA, Lind I, Svensson L, Weström L, Møller BR. Antibodies to Chlamydia trachomatis, Mycoplasma hominis, and Neisseria gonorrhoeae in sera from patients with acute salpingitis. Br J Vener Dis 1981; 57(2): 125-9.
477. Mardh PA, Stormby N, Westrom L. [Gonorrhea in a female out-patient-material]. Lakartidningen 1971; 68(38): 4261-2.
478. Mareş M, Socolov D, Doroftei B, Botezatu A, Goia CD. The prevalence of some bacterial markers in female patients undergoing an initial infertility evaluation in north-east Romania. Roum Arch Microbiol Immunol 2009; 68(3): 171-4.
479. Markowska-Bernaczyk D, Chodynicka B, Perkowska Z, Budkiewicz-Juchnowicz B, Zdrodowska-Stefanow B. [Relation between alcohol abuse and the prevalence of sexually-transmitted diseases]. Przegl Dermatol 1983; 70(2): 185-91.
480. Marschall B. Chlamydia trachomatis infections in a Greenlandic district. Arctic Med Res 1992; 51(1): 23-8.
481. Marsh K, Chan S, Wheatley N, Duffell S, Lau R, Hughes G. Missed STI and HIV testing opportunities among male prisoners in England. Sexually Transmitted Infections Conference: STI and AIDS World Congress 2013; 89(SUPPL. 1).
482. Masterton RG, Strike PW. Sexually transmitted diseases in a British military force in peacetime Europe, 1970-83. Genitourin Med 1988; 64(1): 54-8.
483. Matinato C, Lunghi G, Orlandi A, et al. Evaluation of a multiplex real-time PCR system (DX CT/ NG/MG assay, Bio-Rad) for the detection of Chlamydia trachomatis, Neisseria gonorrhoeae and Mycoplasma genitalium in urogenital specimens. Clinical Microbiology and Infection 2012; 18: 114-715.
484. Matser A, Schim van der Loeff M, Geskus R. Determining the Most Likely Source of Infection: An Application to Neisseria Gonorrhoeae Among Men Who Have Sex with Men. Epidemiology 2018; 29(3): 421-30.
485. Matteelli A, Capelli M, Sulis G, et al. Prevalence of Chlamydia trachomatis and Neisseria gonorrhoeae infection in adolescents in Northern Italy: an observational school-based study. BMC public health 2016; 16: 200.
486. Mc Grath-Lone L, Marsh K, Hughes G, Ward H. The sexual health of female sex workers compared with other women in England: analysis of cross-sectional data from genitourinary medicine clinics. Sex Transm Infect 2014; 90(4): 344-50.
487. Mc Grath-Lone L, Marsh K, Hughes G, Ward H. The sexual health of male sex workers in England: analysis of cross-sectional data from genitourinary medicine clinics. Sexually transmitted infections 2014; 90(1): 38-40.
488. McCarty E, Quinn K, Donnelly C, Quah S, Emerson C. Testing for sexually transmissible infections in patients with HIV infection: What are we missing? HIV Medicine 2011; 12(SUPPL. 1): 14-86.
489. McCormack S, Dunn DT, Desai M, et al. Pre-exposure prophylaxis to prevent the acquisition of HIV-1 infection (PROUD): effectiveness results from the pilot phase of a pragmatic open-label randomised trial. Lancet 2016; 387(10013): 53-60.
490. McKenna JG, Fallon RJ, Moyes A, Young H. Anogenital non-gonococcal neisseriae: prevalence and clinical significance. Int J STD AIDS 1993; 4(1): 8-12.
491. McMillan S, Thompson M, Jones R. Sexual health @ the bush. HIV Medicine 2014a; 3): 37.
492. McMillan S, Whitlock G, Day S, et al. Targeted outreach: Does it work? HIV Medicine 2014b; 3): 18.
493. Mechie AM, Pritchard JG. Venereal infection in elderly people. Gerontol Clin (Basel) 1966; 8(4): 207-14.
494. Mendes-Bastos P, Brasileiro A, Matos-Pires E, et al. De novo HIV infection diagnoses in a Department of Dermatology and Venereology in Lisbon, Portugal. Int J STD AIDS 2017; 28(9): 887-92.

495. Mendling W, Krasemann C. Bacteriological findings and therapeutic consequences in adnexitis. Geburtshilfe und Frauenheilkunde 1986; 46(7): 462-6.
496. Mensforth S, Radcliffe K. Is it time to reconsider epidemiological treatment for gonorrhoea? Int J STD AIDS 2018; 29(10): 1043-4.
497. Mesko M, Lipenský S, Jedinák J. [Gonorrhea in women prior to pregnancy interruption]. Zentralbl Gynakol 1972; 94(5): 169-72.
498. Mesko M, Lipensky S, Smotlak A, Jedinak J, Bakos J. [Diagnosis, clinical importance and epidemiology of gonorrhea in pregnancy]. Cesk Gynekol 1972; 37(1): 50-1.
499. Mezinova NN. [TREATMENT OF FEMALE STERILITY, CAUSED BY INFLAMMATORY PROCESSES IN THE UTERINE ADNEXA, WITH CORTISONE AND HYALURONIDASE COMBINED WITH ANTIBIOTICS]. Akush Ginekol (Mosk) 1963; 39: 92-5.
500. Mierzejewski W, Rogoza A, Zabul J. [Evaluation of the bacteriological examination of the semen of men in childless marriages]. Ginekol Pol 1987; 58(12): 811-5.
501. Miettinen A, Heinonen PK, Teisala K, Hakkarainen K, Punnonen R. Serologic evidence for the role of Chlamydia trachomatis, Neisseria gonorrhoeae, and Mycoplasma hominis in the etiology of tubal factor infertility and ectopic pregnancy. Sex Transm Dis 1990; 17(1): 10-4.
502. Miettinen A, Saikku P, Jansson E, Paavonen J. Epidemiologic and clinical characteristics of pelvic inflammatory disease associated with Mycoplasma hominis, Chlamydia trachomatis, and Neisseria gonorrhoeae. Sex Transm Dis 1986; 13(1): 24-8.
503. Mihalik N, Pónyai K, Tóth B, et al. P3.072 STI Co-Infections Without Any Symptoms? - Screening is Necessary. Sexually Transmitted Infections 2013; 89(Suppl 1): A170.3-A.
504. Mikl J, Sudar Z, Smith PF, et al. HIV infection and high risk behavior among patients attending an STD referral clinic in Prague, Czech Republic. Sex Transm Infect 1998; 74(2): 128-30.
505. Milosovic P, Simkovicova M, Hausmannova Z. In vitro sensitivity of strains of Neisseria gonorrhoeae to antibiotics in 1982-1986 in the Regional Hygiene Station Bratislava. Cesko-Slovenska Dermatologie 1988; 63(3): 178-84.
506. Miron N, Socolov D, Mares M, et al. Bacteriological agents which play a role in the development of infertility. Acta Microbiologica et Immunologica Hungarica 2013; 60(1): 41-53.
507. Modde H, Krech U. [Gonococcal diseases from the clinical and bacteriological viewpoint]. Schweiz Med Wochenschr 1969; 99(35): 1242-5.
508. Moi H. Prevalence of bacterial vaginosis and its association with genital infections, inflammation, and contraceptive methods in women attending sexually transmitted disease and primary health clinics. Int J STD AIDS 1990; 1(2): 86-94.
509. Moi H, Reinton N, Moghaddam A. Mycoplasma genitalium is associated with symptomatic and asymptomatic non-gonococcal urethritis in men. Sex Transm Infect 2009; 85(1): 15-8.
510. Møller BR, Ahrons S, Laurin J, Mårdh PA. Pelvic infection after elective abortion associated with Chlamydia trachomatis. Obstet Gynecol 1982; 59(2): 210-3.
511. Moller BR, Allen J, Toft B. Pelvic inflammatory disease after hysterosalpingography associated with Chlamydia trachomatis and Mycoplasma hominis. British Journal of Obstetrics and Gynaecology 1984; 91(12): 1181-7.
512. Moller BR, From E, Christensen RB, Heilmann B, Jensen KE, Thorsen P. The venereological profile in Godthab District Venereal Clinic, Nuuk, Gronland. A 3-month study in 1991. [Danish]. Ugeskrift for laeger 1992; 154(21): 1505-8.
513. Molzhaninov EV, Entin BV, Sliusar ZK. [Results of the anti-inflammatory treatment of women with a history of ascending gonorrhea]. Akush Ginekol (Mosk) 1982; (4): 44-5.
514. Mor Z, Shohat T, Goor Y, Dan M. Risk behaviors and sexually transmitted diseases in gay and heterosexual men attending an STD clinic in Tel Aviv, Israel: A cross-sectional study. Israel Medical Association Journal 2012; 14(3): 147-51.
515. Moreno-Ribera N, Fuertes-de Vega I, Blanco-Arevalo JL, et al. Sexually Transmitted Infections: Experience in a Multidisciplinary Clinic in a Tertiary Hospital (2010-2013). Actas Dermosifiliogr 2016; 107(3): 235-41.
516. Morgat M, Bouiller K, Vuillemenot JB, et al. Number, type and cost of microbiological tests during HIV Pre-Exposure Prophylaxis: The experience of a French hospital. Infect Dis Now 2021; 51(4): 357-61.
517. Morris GC, Stewart CM, Schoeman SA, Wilson JD. A cross-sectional study showing differences in the clinical diagnosis of pelvic inflammatory disease according to the experience of clinicians: implications for training and audit. Sex Transm Infect 2014; 90(6): 445-51.
518. Mulcahy FM, Lacey CJ. Sexually transmitted infections in adolescent girls. Genitourin Med 1987; 63(2): 119-21.
519. Munkvad IM, Klemp P. Co-existence of venereal infection and pediculosis pubis. Acta Derm Venereol 1982; 62(4): 366-7.
520. Muñoz-Pérez MA, Rodriguez-Pichardo A, Camacho Martinez F. Sexually transmitted diseases in 1161 HIV-positive patients: a 38-month prospective study in southern Spain. J Eur Acad Dermatol Venereol 1998; 11(3): 221-6.
521. Nanov Z, Kholevich Z, Nanova B. [Spermatogenesis disorders in men with a history of gonorrhea]. Akush Ginekol (Sofiia) 1987; 26(1): 56-9.
522. Napoli Z, Donati S, Cafissi A, et al. Frequency of most important sexually transmitted diseases and methodological implications. Biochimica Clinica 2013; 1: S142.
523. Nayyar KC, Cummings M, Weber J. Prevalence of genital pathogens among female prostitutes in New York City and in Rotterdam. Sexually Transmitted Diseases 1986; 13(2): 105-7.
524. Netak Y, Vasylyev M, Sluzhynska O, et al. Results of STI/HIV counseling and testing program for commercial sex workers , drug users, men have sex with men and former prisoners during 2012-2013 in Lviv region (Ukraine). Sexually Transmitted Diseases 2014; 1): S123.
525. Nicholls JE, Turner KME, North P, et al. Cross-sectional study to evaluate Trichomonas vaginalis positivity in women tested for Neisseria gonorrhoeae and Chlamydia trachomatis, attending genitourinary medicine and primary care clinics in Bristol, South West England. Sex Transm Infect 2018; 94(2): 93-9.
526. Nichols K, Sim-Ifere O, Pinto-Sander N, Soni S. Testing for and managing Mycoplasma genitalium in women attending a GUM clinic. HIV Medicine 2018; 19(Supplement 2): S38.
527. Nicol E, Shaw J, Ward C. A review of sexually acquired proctitis: An array of symptoms, investigations and treatments. Sexually Transmitted Infections 2016; 92(Supplement 1): A39.
528. Nielsen FH. [Routine culture for gonococci in a family planning clinic]. Ugeskr Laeger 1974; 136(14): 767-8.
529. Nielsen LA, Mikkelsen SJ, Charles AV. [Chlamydia trachomatis and Neisseria gonorrhoeae infections in sexually abused children in Jutland]. Ugeskr Laeger 2002; 164(49): 5806-9.
530. Nielsen R, Søndergaard J, Ullman S. Asymptomatic male and female gonorrhoea. Acta Derm Venereol 1975; 55(6): 499-501.
531. Nielsen S, Haar K, Sailer A, Hamouda O. STI rates and risk factors among female sex workers attending STI testing sites in Germany, January - December 2010. Sexually Transmitted Infections 2011; 1): A125-A6.
532. Nijhuis RHT, Duinsbergen RG, Pol A, Godschalk PCR. Prevalence of Chlamydia trachomatis, Neisseria gonorrhoeae, Mycoplasma genitalium and Trichomonas vaginalis including relevant resistance-associated mutations in a single center in the Netherlands. Eur J Clin Microbiol Infect Dis 2020.
533. Nitschke H, Oliveira F. 10 years of experience: Comprehensive sexual health services for female sex workers (FSW) in Cologne. Sexually Transmitted Infections Conference: STI and AIDS World Congress 2013; 89(SUPPL. 1).
534. Nolskog P, Backhaus E, Nasic S, Enroth H. STI with Mycoplasma genitalium-more common than Chlamydia trachomatis in patients attending youth clinics in Sweden. Eur J Clin Microbiol Infect Dis 2019; 38(1): 81-6.
535. Nouchi A, Caby F, Palich R, Monsel G, Caumes AE. Travel-associated STI amongst HIV and non-HIV infected travellers. J Travel Med 2019; 26(8).

536. Nugent D, Stirrup O, Pett S, et al. Performance of human papillomavirus DNA detection in residual specimens taken for Chlamydia trachomatis and Neisseria gonorrhoeae nucleic acid amplification testing in men who have sex with men. Sex Transm Infect 2021; 97(7): 541-6.
537. O'Brien A, Sherrard-Smith E, Sile B, Watts C, Simms I. Spatial clusters of gonorrhoea in England with particular reference to the outcome of partner notification: 2012 and 2013. PLoS One 2018; 13(4): e0195178.
538. Oakeshott P, Aghaizu A, Hay P, et al. Is Mycoplasma genitalium in women the "New Chlamydia?" A community-based prospective cohort study. Clin Infect Dis 2010; 51(10): 1160-6.
539. Oakeshott P, Kerry-Barnard S, Fleming C, et al. 'Test n Treat' (TnT): a cluster randomized feasibility trial of on-site rapid Chlamydia trachomatis tests and treatment in ethnically diverse, sexually active teenagers attending technical colleges. Clin Microbiol Infect 2019; 25(7): 865-71.
540. Oborin DA, Nikolaeva NV, Godovalov AP, Karpunina TI. [Etiology of purulent-inflammatory processes in the genital tract: suspicions of clinicians and problems of laboratory confirmation.]. Klin Lab Diagn 2020; 65(5): 328-31.
541. Oganesian MG. [An intracutaneous test with gonococcal allergen in married couples with impaired reproductive function]. Vestn Dermatol Venerol 1990; (1): 57-8.
542. Ogaz D, Miltz AR, Desai S, et al. Preparing for prep in England: Prevalence and incidence of HIV and bacterial stis. Topics in Antiviral Medicine 2019; 27(SUPPL 1): 20s-1s.
543. Op de Coul ELM, Peek D, van Weert YWM, et al. Chlamydia trachomatis, Neisseria gonorrhoea, and Trichomonas vaginalis infections among pregnant women and male partners in Dutch midwifery practices: prevalence, risk factors, and perinatal outcomes. Reprod Health 2021; 18(1): 132.
544. Opaneye AA. Sexually transmitted diseases among women in Coventry. J R Soc Health 1997; 117(1): 37-40.
545. Opaneye AA, Willmott C. The role of genito-urinary medicine in adolescent sexuality. Genitourin Med 1991; 67(1): 44-6.
546. Orduña Domingo A, Chu JJ, Eiros Bouza JM, et al. [Age and sex distribution of sexually transmitted diseases in Valladolid. A study of 5076 cases]. Rev Sanid Hig Publica (Madr) 1991; 65(3): 247-58.
547. Orellana MA, Gómez M, Sánchez MT, Fernández-Chacón T. [Diagnosis of urethritis in men. A 3-year review]. Rev Esp Quimioter 2009; 22(2): 83-7.
548. Orellana MA, Gómez-Lus ML. [Which is the best empirical treatment in patients with urethritis?]. Rev Esp Quimioter 2011; 24(3): 136-42.
549. Orellana MA, Gomez-Lus ML. Microorganisms isolated in urethral samples in women. Clinical Microbiology and Infection 2011; 4): S814-S5.
550. Orellana MA, Gomez-Lus ML. Urethritis epidemiology in men in central Madrid, Spain. Clinical Microbiology and Infection 2011; 4): S813-S4.
551. Orsos M, Sziller I, Pulay T, Paulin F, Csomor S, Nasz I. [A microbiology clinic in outpatient gynecologic service]. Orv Hetil 1989; 130(24): 1259-62.
552. Ortayli N, Sahip Y, Amca B, Say L, Sahip N, Aydin D. Curable sexually transmitted infections among the clientele of a family planning clinic in Istanbul, Turkey. Sex Transm Dis 2001; 28(1): 58-61.
553. Osbak KK, Meehan CJ, S GR, et al. Superimposing incident sexually transmitted infections on HIV phylogram to investigate possible misclassification of men who have sex with men as heterosexuals in a cohort in Antwerp, Belgium. Int J STD AIDS 2019; 30(5): 486-95.
554. Oskarsson T, Geirsson RT, Steingrímsson O, Thorarinsson H. Lower genital tract infection with Neisseria gonorrhoeae and Chlamydia trachomatis in women requesting induced abortion and in their sexual partners. Acta Obstet Gynecol Scand 1990; 69(7-8): 635-40.
555. Ostergaard L, Andersen B, Moller JK, Olesen F. Home sampling versus conventional swab sampling for screening of Chlamydia trachomatis in women: a cluster-randomized 1-year follow-up study. Clin Infect Dis 2000; 31(4): 951-7.
556. Ouzounova-Raykova V, Jordanov D, El-Tibi M, Mitov I. Gonococcal infection in symptomatic and asymptomatic persons seeking medical clinics in Sofia--a 3-year study 2008-2010. Apmis 2011; 119(12): 864-7.
557. Ozturk CE, Ozdemir I, Yavuz T, Kaya D, Behcet M. Etiologic agents of cervicovaginitis in Turkish women. Saudi Medical Journal 2006; 27(10): 1503-7.
558. Pace CA, Lioznov D, Cheng DM, et al. Sexually transmitted infections among HIV-infected heavy drinkers in St Petersburg, Russia. Int J STD AIDS 2012; 23(12): 853-8.
559. Pagani C, Rimoldi SG, Chenal LL, et al. Sexually tnsmitted infections in women attending the obstetrics and gynaecologic unit of a university hospital in the north of Milan: Prevalence and risk behaviours. Clinical Microbiology and Infection 2012; 3): 810.
560. Pagani N, De Bartolo A, Pescatori T, Lusco G, Clerici P. Rapid diagnosis of infections caused by Chlamydia trachomatis and Neisseria gonorroeae. Microbiologia Medica 1996; 11(3): 246-7.
561. Palous V, Sturma J, Krecek J, Potuzník V, Hausner O. [Experience with the diagnosis and treatment of gonorrhea in women]. Cesk Dermatol 1971; 46(6): 256-61.
562. Papadogeorgaki H, Caroni C, Frangouli E, Flemetakis A, Katsambas A, Hadjivassiliou M. Prevalence of sexually transmitted infections in female sex workers in Athens, Greece - 2005. Eur J Dermatol 2006; 16(6): 662-5.
563. Papadogeorgakis H, Pittaras TE, Papaparaskevas J, Pitiriga V, Katsambas A, Tsakris A. Chlamydia trachomatis serovar distribution and Neisseria gonorrhoeae coinfection in male patients with urethritis in Greece. J Clin Microbiol 2010; 48(6): 2231-4.
564. Papanagiotou A, Terzi N, Vlachos S, et al. Symptomatic and asymptomatic infections of the lower genital tract in pregnant and non-pregnant women of reproductive age. Clinical Microbiology and Infection 2012; 18: 114-715.
565. Parker RD, Rüütel K. Sexually Transmitted Infections - Prevalence, Knowledge and Behaviours among Professional Defence Forces in Estonia: a Pilot Study. Cent Eur J Public Health 2017; 25(1): 11-4.
566. Parra-Sanchez M, Garcia-Rey S, Pueyo Rodriguez I, Viciana Fernandez P, Torres Sanchez MJ, Palomares Folia JC. Clinical and epidemiological characterisation of lymphogranuloma venereum in southwest Spain, 2013-2015. Sexually Transmitted Infections 2016; 92(8): 629-31.
567. Pasieczny T, Sommerville RG. Outbreak of non-specific urethritis associated with the presence of complement-fixing antibodies to the LB4 strain of TRIC agent. Br J Vener Dis 1966; 42(3): 191-4.
568. Pearson RC, Baumber CD, McGhie D, Thambar IV. The relevance of Chlamydia trachomatis in acute epididymitis in young men. Br J Urol 1988; 62(1): 72-5.
569. Pec J, Jedinak J, Moravcik P. [Trichomoniasis as a concomitant infection in female gonorrhoea (author's transl)]. Cesk Dermatol 1981; 56(2): 119-24.
570. Peeters MF, Bots RS, Merkus JM, Prak FM. [Chlamydia trachomatis, infertility and in vitro fertilization]. Ned Tijdschr Geneeskd 1988; 132(31): 1438-42.
571. Pena MJ, Campos-Herrero MI, Ruiz MC, Rodriguez H, Lafarga B. Microbiological study of vulvovaginitis in premenarcheal girls. [Spanish]. Enfermedades Infecciosas y Microbiologia Clinica 1996; 14(5): 311-3.
572. Penney GC, Thomson M, Norman J, et al. A randomised comparison of strategies for reducing infective complications of induced abortion. Br J Obstet Gynaecol 1998; 105(6): 599-604.
573. Perea EJ, Alvarez-Dardet C, Borobio MV, et al. Three years' experience of sexually transmitted diseases in Seville, Spain. Br J Vener Dis 1981; 57(3): 174-7.
574. Pereyre S, Laurier Nadalié C, Bébéar C. Mycoplasma genitalium and Trichomonas vaginalis in France: a point prevalence study in people screened for sexually transmitted diseases. Clin Microbiol Infect 2017; 23(2): 122.e1-.e7.
575. Pérez Bernal AM, Hernández Aguado I, Sánchez-Pedreño Guillén P, Feliu Pérez MM, Camacho Martínez F. [Mucopurulent cervicitis at a clinic for sexually transmitted diseases]. Med Cutan Ibero Lat Am 1987; 15(6): 517-22.

576. Perez-Hernandez I, Palacios R, Gonzalez-Domenech C, et al. Should screening for Chlamydia trachomatis and Neisseria gonorrhoeae in HIV-men who have sex with men be recommended? Journal of the International AIDS Society 2014; 3): 112-3.
577. Perez-Torralba C, Ruiz-Olivares M, Sanbonmatsu-Gamez S, Exposito-Ruiz M, Navarro-Mari JM, Gutierrez-Fernandez J. [Increased infections by herpes simplex virus type 1 and polymicrobials of the genital tract, in the general population of a Spanish middle city]. Rev Esp Quimioter 2021; 34(4): 320-9.
578. Persson K, Persson K, Hansson H, et al. Prevalence of nine different micro-organisms in the female genital tract. A comparison between women from a venereal disease clinic and from a health control department. Br J Vener Dis 1979; 55(6): 429-33.
579. Peters RP, Nijsten N, Mutsaers J, Jansen CL, Morré SA, van Leeuwen AP. Screening of oropharynx and anorectum increases prevalence of Chlamydia trachomatis and Neisseria gonorrhoeae infection in female STD clinic visitors. Sex Transm Dis 2011; 38(9): 783-7.
580. Peters RP, Verweij SP, Nijsten N, et al. Evaluation of sexual history-based screening of anatomic sites for chlamydia trachomatis and neisseria gonorrhoeae infection in men having sex with men in routine practice. BMC Infect Dis 2011; 11: 203.
581. Petersen CS, Danielsen AG, Renneberg J. Bacterial vaginosis: the leading cause of vaginal discharge in women attending an STD clinic in Copenhagen. Acta Derm Venereol 1999; 79(5): 414-5.
582. Peuchant O, de Diego S, Le Roy C, et al. Comparison of three real-time PCR assays for the detection of Chlamydia trachomatis and Neisseria gonorrhoeae in young pregnant women. Diagnostic Microbiology and Infectious Disease 2015; 83(4): 335-7.
583. Peuchant O, Le Roy C, Desveaux C, et al. Screening for Chlamydia trachomatis, Neisseria gonorrhoeae, and Mycoplasma genitalium should it be integrated into routine pregnancy care in French young pregnant women? Diagn Microbiol Infect Dis 2015; 82(1): 14-9.
584. Peuchant O, Touati A, Laurier-Nadalié C, et al. Prevalence of lymphogranuloma venereum among anorectal Chlamydia trachomatis-positive MSM using pre-exposure prophylaxis for HIV. Sex Transm Infect 2020; 96(8): 615-7.
585. Pfortmueller CA, Koetter JN, Zimmermann H, Exadaktylos AK. Sexual activity-related emergency department admissions: eleven years of experience at a Swiss university hospital. Emerg Med J 2013; 30(10): 846-50.
586. Phillips MD, Davies A, Hewart R, Acladious N, Morgan E. An audit of Neisseria gonorrhoeae detection methods in a district general hospital genitourinary medicine service. Sexually Transmitted Infections Conference: 4th Joint BASHH ASTDA Meeting Brighton United Kingdom Conference Publication: 2012; 88(SUPPL. 1).
587. Pilatz A, Hossain H, Kaiser R, et al. Acute epididymitis revisited: impact of molecular diagnostics on etiology and contemporary guideline recommendations. Eur Urol 2015; 68(3): 428-35.
588. Pittrof R, McLellan J. Test Not Talk screening for asymptomatic men. Int J STD AIDS 2007; 18(4): 274-5.
589. Piyadigamage A, Wilson J. Improvement in the clinical cure rate of outpatient management of pelvic inflammatory disease following a change in therapy. Sex Transm Infect 2005; 81(3): 233-5.
590. Plaas K, Eelmets L, Ratnik K, Janes J, Ruutel K, Naaber P. Sexually transmitted infections among men who have sex with men: Prevalence, locations and challenges for molecular assays. International Journal of STD and AIDS 2015; 1): 102.
591. Plamenova GM, Raykova V, Pehlivanov G. Reliable methods for detection of sexually transmited bacterial fastidious microorganisms. International Journal of STD and AIDS 2015; 1): 84.
592. Platt L, Grenfell P, Bonell C, et al. Risk of sexually transmitted infections and violence among indoor-working female sex workers in London: the effect of migration from Eastern Europe. Sex Transm Infect 2011; 87(5): 377-84.
593. Plecko V, Zele-Starcevic L, Tripkovic V, et al. Unusually low prevalence of Mycoplasma genitalium in urine samples from infertile men and healthy controls: a prevalence study. BMJ Open 2014; 4(8): e005372.
594. Pond M, Wagner J, Laing K, et al. Bacterial populations detected within first void urine samples of symptomatic male patients with urethritis. Sexually Transmitted Infections 2017; 93(Supplement 2): A58-A9.
595. Pónyai K, Marschalkó M, Schöffler M, et al. [Analysis of syphilis and gonorrhoea cases, based on data from the National STD Centre, Department of Dermatology and Venerology, Semmelweis University (2005-2008)]. Orv Hetil 2009; 150(38): 1765-72.
596. Popert AJ, Gill AJ, Laird SM. A Prospective Study of Reiter's Syndrome. An Interim Report on the First 82 Cases. Br J Vener Dis 1964; 40(3): 160-5.
597. Prien-Larsen JC, Kjer JJ. [Prevalence of positive gonococcal and chlamydial findings in women applying for termination of pregnancy. A screening evaluation]. Ugeskr Laeger 1989; 151(26): 1671-2.
598. Prins M, Hooykaas C, Coutinho RA, van Doornum GJ, van den Hoek AJ. Incidence and risk factors for acquisition of sexually transmitted diseases in heterosexuals with multiple partners. Sex Transm Dis 1994; 21(5): 258-67.
599. Pugh E, Pakianathan M, McCormick C, Webb H, Korley K, Patel S. The sexual health behaviour of HIV positive patients in an urban UK cohort. HIV Medicine 2012; 1): 17.
600. Pulvirenti MG, McErlean C, McGrath NF, et al. Evaluation of a multiplex array for the simultaneous detection of ten common STI pathogens. Clinical Chemistry 2015; 61(10 SUPPL. 1): S139-S40.
601. Punnonen R, Terho P, Nikkanen V, Meurman O. Chlamydial serology in infertile women by immunofluorescence. Fertil Steril 1979; 31(6): 656-9.
602. Puolakkainen M, Vesterinen E, Purola E, Saikku P, Paavonen J. Persistence of chlamydial antibodies after pelvic inflammatory disease. J Clin Microbiol 1986; 23(5): 924-8.
603. Purola E, Jahkola M, Osterlund K. Gonorrhoea, trichomoniasis and yeast infection in late pregnancy in an unselected series of gravidae. Ann Chir Gynaecol Fenn 1967; 56(1): 95-8.
604. Querido L, Haspels AA. The incidence of gonorrhea in an abortion population. Contraception 1980; 22(4): 441-4.
605. Qvigstad E, Skaug K, Jerve F, Fylling P, Ulstrup JC. Pelvic inflammatory disease associated with Chlamydia trachomatis infection after therapeutic abortion. A prospective study. Br J Vener Dis 1983; 59(3): 189-92.
606. Rahib D, Delagreverie HM, Bichard I, et al. Expanding testing strategies in Paris: A free postal comprehensive STI test kit. Topics in Antiviral Medicine 2019; 27(SUPPL 1): 21s.
607. Raiga J, Canis M, Le Bouedec G, et al. Laparoscopic management of adnexal abscesses: Consequences for fertility. Fertility and Sterility 1996; 66(5): 712-7.
608. Rao GG, Bacon L, Evans J, Dejahang Y, Michalczyk P, Donaldson N. Prevalence of Neisseria gonorrhoeae infection in young subjects attending community clinics in South London. Sex Transm Infect 2008; 84(2): 117-21.
609. Reimann K, Lind I, Andersen KE. An indirect haemagglutination test for demonstration of gonococcal antibodies using gonococcal pili as antigen. II. Serological investigation of patients attending a dermato-venereological outpatients clinic in Copenhagen. Acta Pathol Microbiol Scand C 1980; 88(3): 155-62.
610. Reimann K, Odum L, Larsen SO, Lind I. [The gonococcal antibody test (GAT) as a diagnostic aid. When can GAT be employed?]. Ugeskr Laeger 1986; 148(37): 2345-8.
611. Reimann K, Odum L, Larsen SO, Lind I. Indirect haemagglutination test using gonococcal pilus antigen: how useful to diagnose gonorrhoea? Genitourin Med 1987; 63(4): 250-5.
612. Reinton N, Moi H, Olsen AO, et al. Anatomic distribution of Neisseria gonorrhoeae, Chlamydia trachomatis and Mycoplasma genitalium infections in men who have sex with men. Sex Health 2013; 10(3): 199-203.
613. Renton A, Filatova E, Ison C, et al. A trial of the validity of genital smears and cultures with gonococcal vaccine provocation in diagnosing genital gonorrhoea in women. Int J STD AIDS 2009; 20(1): 24-9.

614. Resl V, Resl V, Jr. [Statistical evaluation of the activities of the venereological laboratory and departments of the Dermatological clinic of the University Hospital in Pilsen with regard to gonorrhea. Statistical data from the West Bohemiam Region]. Cesk Dermatol 1976; 51(2): 117-3.
615. Reynolds M, Wilson J. Is Trichomonas vaginalis still a marker for other sexually transmitted infections in women? Int J STD AIDS 1996; 7(2): 131-2.
616. Reynolds-Wright J, Verrall F, Hassan-Ibrahim M, Soni S. Implementing a test and treat pathway for mycoplasma genitalium in men with urethritis attending a gum clinic. Sexually Transmitted Infections 2016; 92(Supplement 1): A12.
617. Ribeiro S, de Sousa D, Medina D, Castro R, Lopes Â, Rocha M. Prevalence of gonorrhea and chlamydia in a community clinic for men who have sex with men in Lisbon, Portugal. Int J STD AIDS 2019; 30(10): 951-9.
618. Rich RS, Leventhal A, Sheffer R, Mor Z. Risky sexual behavior and sexually transmitted infections among men who have sex with men and purchase sex attending an Israeli sexually transmitted infection clinic. Int J STD AIDS 2020; 31(3): 236-43.
619. Richardson D, Devlin J, Fitzpatrick C, Pinto-Sander N. Sexually transmitted Shigella flexneri and Shigella sonnei in men who have sex with men. Sex Transm Infect 2021; 97(3): 244.
620. Rimoldi SG, Pagani C, Giacomet V, et al. Sexually transmissible infections among young adolescents in Milan areas: A multicentre study. Clinical Microbiology and Infection 2012; 3): 235.
621. Riordan T, Macaulay ME, James JM, et al. A prospective study of genital infections in a family-planning clinic. 1. Microbiological findings and their association with vaginal symptoms. Epidemiol Infect 1990; 104(1): 47-53.
622. Ripa KT, Svensson L, Treharne JD, Westrom L, Mardh PA. Chlamydia trachomatis infection in patients with laparoscopically verified acute salpingitis. Results of isolation and antibody determinations. Am J Obstet Gynecol 1980; 138(7 Pt 2): 960-4.
623. Roberts J, Richardson D, Evans D, Hutchinson L, Tweed M, Dean G. Uptake of gonorrhoea and chlamydia self-taken nucleic acid amplification tests by men who have sex with men attending an outreach clinic and sex on premises venues. International Journal of STD and AIDS 2013; 1): 34.
624. Robertson DH, George G. Medical and legal problems in the treatment of delinquent girls in Scotland. II. Sexually transmitted disease in girls in custodial institutions. Br J Vener Dis 1970; 46(1): 46-53.
625. Robertson JN, Ward ME, Conway D, Caul EO. Chlamydial and gonococcal antibodies in sera of infertile women with tubal obstruction. J Clin Pathol 1987; 40(4): 377-83.
626. Robinson DK. Letter: Gonorrhoea of the pharynx. Br Med J 1974; 2(5917): 500-1.
627. Rodríguez R, Hernández R, Cabrera L, Fernández A, Alberto J, Prieto P. Análisis del factor cervical de esterilidad. Clínica e Investigación en Ginecología y Obstetricia 2003; 30(2): 53-8.
628. Rodríguez R, Hernández R, Fuster F, Torres A, Prieto P, Alberto J. [Genital infection and infertility]. Enferm Infecc Microbiol Clin 2001; 19(6): 261-6.
629. Rodríguez-Domínguez M, Puerta T, Menéndez B, et al. Clinical and epidemiological characterization of a lymphogranuloma venereum outbreak in Madrid, Spain: co-circulation of two variants. Clin Microbiol Infect 2014; 20(3): 219-25.
630. Rodriguez-Pichardo A, Aznar J, Camacho F, Borobio MV, Perea EJ. Sexually transmitted diseases in homosexual males in Seville, Spain. Genitourin Med 1991; 67(4): 335-8.
631. Rolland C, de La Rochebrochard E, Piron P, Shelly M, Segouin C, Troude P. Who fails to return within 30 days after being tested positive for HIV/STI in a free testing centre? BMC Infect Dis 2020; 20(1): 795.
632. Rolskov AS, Bjorn-Mortensen K, Mulvad G, Poulsen P, Jensen JS, Pedersen ML. Rapid change in the ciprofloxacin resistance pattern among Neisseria gonorrhoeae strains in Nuuk, Greenland: time to reconsider preventive and treatment strategies. Int J Circumpolar Health 2015; 74: 26916.
633. Romosan G, Bjartling C, Skoog L, Valentin L. Ultrasound for diagnosing acute salpingitis: a prospective observational diagnostic study. Hum Reprod 2013; 28(6): 1569-79.
634. Ronn M, Hughes G, White P, Simms I, Ison C, Ward H. Characteristics of LGV repeaters: analysis of LGV surveillance data. Sex Transm Infect 2014; 90(4): 275-8.
635. Rosenvinge MM, Lau R. Screening for asymptomatic chlamydia in women - How often would gonorrhoea be missed? International Journal of STD and AIDS 2009; 20(8): 571-2.
636. Ross JD, McMillan A, Young H. Changing trends of gonococcal infection in homosexual men in Edinburgh. Epidemiol Infect 1991; 107(3): 585-90.
637. Ross JD, Scott GR, Busuttil A. Rape and sexually transmitted diseases: patterns of referral and incidence in a department of genitourinary medicine. J R Soc Med 1991; 84(11): 657-9.
638. Rossignol L, Feuillepain L, Ndeikoundam Ngangro N, et al. Estimate of male urethritis incidences in France between 2007 and 2017 with a specific focus on Neisseria gonorrhoeae, Chlamydia trachomatis, and Trichomonas vaginalis infections. BMC Infect Dis 2019; 19(1): 561.
639. Rozova M, Beilina O, Koniuchova K, et al. Experiences from working with sexually transmitted infections and related issues among teenagers and Youth in Tver, Russia. Sexually Transmitted Infections Conference: STI and AIDS World Congress 2013; 89(SUPPL. 1).
640. Ruijs GJ, Schut IK, Schirm J, Schröder FP. Prevalence, incidence, and risk of acquiring urogenital gonococcal or chlamydial infection in prostitutes working in brothels. Genitourin Med 1988; 64(1): 49-51.
641. Ruiz-Olivares M, Foronda-García-Hidalgo C, Sanbonmatsu-Gámez S, Gutiérrez-Fernández J. [Recent prevalence among females from the general population of agents responsible for sexually transmitted infections and other genital infections]. Aten Primaria 2020; 52(10): 799-801.
642. Rumyantseva T, Golparian D, Nilsson CS, et al. Evaluation of the new AmpliSens multiplex real-time PCR assay for simultaneous detection of Neisseria gonorrhoeae, Chlamydia trachomatis, Mycoplasma genitalium, and Trichomonas vaginalis. Apmis 2015; 123(10): 879-86.
643. Ruozi Berretta L, Schepisi P. [Etiological factors in masculine sterility: results of 934 cases]. Patol Clin Ostet Ginecol 1974; 2(5): 230-9.
644. Rüütel K, Lõhmus L, Jänes J. Internet-based recruitment system for HIV and STI screening for men who have sex with men in Estonia, 2013: analysis of preliminary outcomes. Eurosurveillance 2015; 20(15): 21094.
645. Sacks R, Nwokolo N, McOwan A, Whitlock G. Characteristics of those with newly diagnosed HIV at a central London clinic. HIV Medicine 2017; 18(Supplement 1): 50.
646. Sakem B, Michel R, Nydegger UE, et al. Diagnostic relevance of simultaneous testing for Chlamydia trachomatis and Neisseria gonorrhoeae. Infection 2011; 39(3): 231-7.
647. Salfa MC, Suligoi B. Prevalence of Chlamydia trachomatis, Trichomonas vaginalis and Neisseria gonorrhoeae Based on Data Collected by a Network of Clinical Microbiology Laboratories, in Italy. Adv Exp Med Biol 2016; 901: 47-57.
648. Salva AL, Celorio A, Castro A. The clinico-microbiological relationship in pelvic inflammatory disease. Progresos en Obstetricia y Ginecologia 1986; 29(7): 425-32.
649. Samarasekara K, Fitzpatrick C, Finnerty F, Richardson D. Pilot study of pharyngeal Chlamydia trachomatis in HIV-positive and HIV-negative men who have sex with men. Sex Health 2021; 18(2): 200-1.
650. Sambri V, Dirani G, Farabegoli P. How to facilitate and improve screening of sexually-transmitted infections in women population. Sexually Transmitted Infections 2017; 93(Supplement 2): A64-A5.
651. Samra Z, Dan M, Segev S, et al. Prevalence of sexually transmitted pathogens among women attending a methadone clinic in Israel. Genitourin Med 1991; 67(2): 133-6.

652. Santino I, Cauletti M, Del Piano M, Fiamma S, Santi AL. [Bacterial infection from sexual contact in a sampling of a student population]. Nuovi Ann Ig Microbiol 1985; 36(2): 105-12.
653. Schabereiter-Gurtner C, Hufnagl P, Sonvilla G, et al. Evaluation of a novel internally controlled real-time PCR assay targeting the 16S rRNA gene for confirmation of Neisseria gonorrhoeae infections. Clinical Microbiology and Infection 2008; 14(5): 480-6.
654. Schmidt AJ, Rasi M, Esson C, et al. The Swiss STAR trial - an evaluation of target groups for sexually transmitted infection screening in the sub-sample of men. Swiss Med Wkly 2020; 150: w20392.
655. Schuurs TA, Verweij SP, Weel JFL, Ouburg S, Morre SA. Detection of Chlamydia trachomatis and Neisseria gonorrhoeae in an STI population: Performances of the Presto CT-NG assay, the Lightmix Kit 480 HT CT/NG and the COBAS Amplicor with urine specimens and urethral/cervicovaginal samples. BMJ Open 2013; 3(12) (no pagination).
656. Sednaoui P, Malkin JE, Alonso JM. Neisseria gonorrhoeae RNA/DNA hybridization and culture for screening of gonococcal infections in a low-prevalence population. Eur J Epidemiol 1996; 12(6): 651-4.
657. Selvaag E. No differences in the prevalence of venereological disease in Norwegians compared with non-Norwegians. Sexually Transmitted Infections 1999; 75(1): 68.
658. Semmola L. [Contribution of observations on the cases of male infertility]. Minerva Dermatol 1967; 42(8): 393-7.
659. Sethi G, Holden BM, Gaffney J, Greene L, Ghani AC, Ward H. HIV, sexually transmitted infections, and risk behaviours in male sex workers in London over a 10 year period. Sex Transm Infect 2006; 82(5): 359-63.
660. Sethupathi M, Blackwell A, Davies H. Rectal Chlamydia trachomatis infection in women. Is it overlooked? Int J STD AIDS 2010; 21(2): 93-5.
661. Shafer MA, Pessione F, Scieux C, et al. Chlamydia trachomatis in French women: Association with smoking, cervical ectopy and other clinical factors. Journal de Gynecologie Obstetrique et Biologie de la Reproduction 1993; 22(2): 163-8.
662. Shahidullah M. Pharyngeal gonorrhoea in homosexuals. Br J Vener Dis 1976; 52(3): 168-9.
663. Shakarishvili A, Dubovskaya LK, Zohrabyan LS, et al. Sex work, drug use, HIV infection, and spread of sexually transmitted infections in Moscow, Russian Federation. Lancet 2005; 366(9479): 57-60.
664. Shawe J, White A, Ball A, et al. Improving the sexual health of homeless people: Does providing nurse-led care within hostels improve contraceptive use and uptake of sexual health screening? European Journal of Contraception and Reproductive Health Care 2014; 1): S140.
665. Shiely F, Hayes K, Horgan M. Comparison of risk factors for prevalent sexually transmitted infections based on attendees at two genitourinary medicine clinics in Ireland. Int J STD AIDS 2014; 25(1): 29-39.
666. Shinskii GE, Ignat'eva GF. [Incidence of gonorrhea and characteristics of its course in husbands of women receiving prophylactic anti-gonorrhea treatment in obstetric-gyneocologic institutions]. Vestn Dermatol Venerol 1975; (6): 78-80.
667. Shipitsyna E, Khusnutdinova T, Budilovskaya O, et al. Bacterial vaginosis-associated vaginal microbiota is an age-independent risk factor for Chlamydia trachomatis, Mycoplasma genitalium and Trichomonas vaginalis infections in low-risk women, St. Petersburg, Russia. Eur J Clin Microbiol Infect Dis 2020; 39(7): 1221-30.
668. Shipitsyna E, Khusnutdinova T, Ryzhkova O, et al. Prevalence of sexually transmitted infections in young people in St. Petersburg, Russia, as Determined using self-collected non-invasive specimens. Sexually Transmitted Infections Conference: STI and AIDS World Congress 2013; 89(SUPPL. 1).
669. Shipitsyna E, Zolotoverkhaya E, Hjelmevoll S, et al. Evaluation of six nucleic acid amplification tests used for diagnosis of Neisseria gonorrhoeae in Russia compared with an international strictly validated real-time porA pseudogene polymerase chain reaction. Journal of the European Academy of Dermatology and Venereology 2009; 23(11): 1246-53.
670. Shone J, Winter A, Jones BL, et al. A Scottish multi-centre service evaluation examining the prevalence and diagnosis of Trichomonas vaginalis in symptomatic women attending sexual health clinics. Int J STD AIDS 2016; 27(12): 1066-70.
671. Silva J, Cerqueira F, Teixeira AL, Campainha R, Amorim J, Medeiros R. Prevalence of Neisseria gonorrhoeae and Trichomonas vaginalis in Portuguese women of childbearing age. Journal of Obstetrics and Gynaecology 2021; 41(2): 254-8.
672. Silva L, Queirós C, Borges-Costa J, Barreto R, Filipe P, Melo-Cristino J. Chlamydia trachomatis Infections in a Tertiary Care Portuguese Hospital: An 11-Year Retrospective Study. Actas Dermosifiliogr 2021; 112(6): 528-33.
673. Singha HS, Balsdon MJ. Screening for gonorrhoea, trichomoniasis and candidosis in women presenting for termination of pregnancy. Br J Clin Pract 1979; 33(6): 163-4.
674. Sivaraj V, Ahamed A, Artykov R, Menon-Johansson A. Epididymitis and its aetiologies in a central London sexual health clinic. Int J STD AIDS 2021; 32(1): 96-9.
675. Skaletz-Rorowski A, Potthoff A, Nambiar S, et al. Sexual behaviour, STI knowledge and Chlamydia trachomatis (CT) and Neisseria gonorrhoeae (NG) prevalence in an asymptomatic cohort in Ruhr-area, Germany: PreYoungGo study. J Eur Acad Dermatol Venereol 2021; 35(1): 241-6.
676. Skapinyecz J, Smid I, Juhasz A, Jeney C, Rozgonyi F, Kovacs P. Planning of empirical antibiotic therapy for women with pelvic inflammatory diseases: a geographical area-specific study. Eur J Obstet Gynecol Reprod Biol 2003; 111(2): 183-8.
677. Skidmore S, Copley S, Cordwell D, Donaldson D, Ritchie D, Spraggon M. Positive nucleic acid amplification tests for Neisseria gonorrhoeae in young people tested as part of the National Chlamydia Screening Programme. Int J STD AIDS 2011; 22(7): 398-9.
678. Skinner CJ, Saulsbury NKG, Goh BT. Sexually transmitted infections in Bangladeshis resident in the UK: A case-control study. Sexually Transmitted Infections 2002; 78(2): 120-2.
679. Skinner CJ, Stokes J, Kirlew Y, Kavanagh J, Forster GE. A case-controlled study of the sexual health needs of lesbians. Genitourin Med 1996; 72(4): 277-80.
680. Skjeldestad FE, Dalen A. Chlamydia trachomatis: is it possible to reduce the number of infections after abortions? Scand J Prim Health Care 1988; 6(2): 125-8.
681. Skjeldestad FE, Jerve F. [Chlamydia trachomatis and Neisseria gonorrhoeae among women seeking abortion in Norway. Results from a nationwide study]. Tidsskr Nor Laegeforen 1992; 112(16): 2082-4.
682. Skjeldestad FE, Johansen OJ, Dalen A. A comparative neonatal study of infants born by mothers with Chlamydia trachomatis in cervix uteri. Acta Paediatr Scand 1987; 76(2): 359-60.
683. Skok J, Kilczewski W, Bramska S, Lesinski J. [Methods and epidemiologic usefulness of prophylactic testing for gonorrhea in women]. Przegl Dermatol 1976; (6 Suppl): 473-6.
684. Skovgaard S, Larsen HK, Sand C, et al. Genital and extra-genital screening for gonorrhoea using the BD Probetec ET system with an in-house PCR method targeting the porA pseudogene as confirmatory test. Acta Derm Venereol 2012; 92(1): 45-9.
685. Slater W, Sadler K, Cassell JA, Horner P, Low N. What can be gained from comprehensive disaggregate surveillance? The Avon Surveillance System for Sexually Transmitted Infections. Sex Transm Infect 2007; 83(5): 411-5.
686. Slurink IAL, van Benthem BHB, van Rooijen MS, Achterbergh RCA, van Aar F. Latent classes of sexual risk and corresponding STI and HIV positivity among MSM attending centres for sexual health in the Netherlands. Sex Transm Infect 2020; 96(1): 33-9.
687. Smith J, Day S, Kinsella R, Perera S. Sexual health beyond the binary: Assessing transgender and non-binary individuals' (TNB) needs and their use of online sexual health services (SHS). International Journal of STD & AIDS 2020; 31(12_suppl): 1-114.
688. Smith JR, Murdoch J, Carrington D, et al. Prevalence of Chlamydia trachomatis infection in women having cervical smear tests. Bmj 1991; 302(6768): 82-4.

689. Snell L, Edwards SG, Benn PD. Potential impact of updated UK guidelines for use of post exposure prophylaxis following sexual exposure in a London sexual health service. Sexually Transmitted Infections Conference: 4th Joint BASHH ASTDA Meeting Brighton United Kingdom Conference Publication: 2012; 88(SUPPL. 1).
690. Solomon F, Sompolinsky D, Caspi E, Alkan WJ. Isolation and identification of Mycoplasma from clinical material in Israel. Isr J Med Sci 1970; 6(5): 605-10.
691. Soni S, Alexander S, Verlander N, et al. The prevalence of urethral and rectal Mycoplasma genitalium and its associations in men who have sex with men attending a genitourinary medicine clinic. Sex Transm Infect 2010; 86(1): 21-4.
692. Soni S, Bond K, Fox E, Grieve AP, Sethi G. Black and minority ethnic men who have sex with men: a London genitourinary medicine clinic experience. Int J STD AIDS 2008; 19(9): 617-9.
693. Soni S, White JA. Self-screening for Neisseria gonorrhoeae and Chlamydia trachomatis in the human immunodeficiency virus clinic--high yields and high acceptability. Sex Transm Dis 2011; 38(12): 1107-9.
694. Sonnenberg P, Clifton S, Beddows S, et al. Prevalence, risk factors, and uptake of interventions for sexually transmitted infections in Britain: findings from the National Surveys of Sexual Attitudes and Lifestyles (Natsal). Lancet 2013; 382(9907): 1795-806.
695. Sorbye IK, Jerve F, Staff AC. Reduction in hospitalized women with pelvic inflammatory disease in Oslo over the past decade. Acta Obstet Gynecol Scand 2005; 84(3): 290-6.
696. Sorensen JL, Thranov I, Hoff G, Dirach J, Damsgaard MT. A double-blind randomized study of the effect of erythromycin in preventing pelvic inflammatory disease after first trimester abortion. Br J Obstet Gynaecol 1992; 99(5): 434-8.
697. Sorlózano-Puerto A, Esteban-Sanchís P, Heras-Cañas V, Fernández-Parra J, Navarro-Mari JM, Gutiérrez-Fernández J. Estudio prospectivo de la incidencia de patógenos genitales oportunistas y estrictos que crecen en medios de cultivo artificiales. Revista del Laboratorio Clínico 2018; 11(3): 123-30.
698. Southgate LJ, Treharne JD, Forsey T. Chlamydia trachomatis and Neisseria gonorrhoeae infections in women attending inner city general practices. Br Med J (Clin Res Ed) 1983; 287(6396): 879-81.
699. Spauwen LW, Niekamp AM, Hoebe CJ, Dukers-Muijrers NH. Drug use, sexual risk behaviour and sexually transmitted infections among swingers: a cross-sectional study in The Netherlands. Sex Transm Infect 2015; 91(1): 31-6.
700. Spinillo A, Gorini G, Piazzi G, Baltaro F, Monaco A, Zara F. The impact of oral contraception on chlamydial infection among patients with pelvic inflammatory disease. Contraception 1996; 54(3): 163-8.
701. Spinner CD, Boesecke C, Jordan C, et al. Prevalence of asymptomatic sexually transmitted infections in HIV-positive men who have sex with men in Germany: results of a multicentre cross-sectional study. Infection 2018; 46(3): 341-7.
702. Spornraft-Ragaller P, Dumke R. Prevalence and antibiotic resistance of rectal Mollicutes in HIV-infected men who have sex with men at the University Hospital of Dresden, Germany. Infection 2020; 48(2): 259-65.
703. Sprague DS, Bullough CHW, Rashid S, Roberts SAM. Screening for and treating Chlamydia trachomatis and Neisseria gonorrhoeae before contraceptive use and subsequent pelvic inflammatory infection. British Journal of Family Planning 1990; 16(2): 54-8.
704. Sprenger K, Evison JM, Zwahlen M, et al. Sexually transmitted infections in HIV-infected people in Switzerland: Cross-sectional study. PeerJ 2014; 2014(1) (no pagination).
705. Srugo I, Steinberg J, Madeb R, et al. Agents of non-gonococcal urethritis in males attending an Israeli clinic for sexually transmitted diseases. Isr Med Assoc J 2003; 5(1): 24-7.
706. Stacey CM, Munday PE, Taylor-Robinson D, et al. A longitudinal study of pelvic inflammatory disease. Br J Obstet Gynaecol 1992; 99(12): 994-9.
707. Staerfelt F, Gundersen TJ, Halsos AM, et al. A survey of genital infections in patients attending a clinic for sexually transmitted diseases. Scand J Infect Dis Suppl 1983; 40: 53-7.
708. Stanley B, Todd A. Testing for Neisseria gonorrhoeae by nucleic acid amplification testing of chlamydia samples using Roche Cobas Amplicor in a rural area in the north of England does not find more gonorrhoea in primary care. Sex Transm Infect 2005; 81(6): 518.
709. Starck V, Bygdeman M, Eriksson G, Heinertz NO, Moberg I. [Screening tests for gonorrhea in clinics for contraceptive advice]. Nord Med 1971; 86(48): 1430-2.
710. Stary A, Kopp W, Heller-Vitouch C. Coincidence of hepatitis B-virus markers and other sexually transmitted diseases in different STD-risk groups. Zentralbl Bakteriol 1992; 276(4): 548-55.
711. Stary A, Kopp W, Hofmann H, Heller-Vitouch C, Kunz C. Seroepidemiologic study of hepatitis C virus in sexually transmitted disease risk groups. Sex Transm Dis 1992; 19(5): 252-8.
712. Stary A, Kopp W, Söltz-Szöts J. Medical health care for Viennese prostitutes. Sex Transm Dis 1991; 18(3): 159-65.
713. Stary A, Söltz-Szöts J, Stary J, Gross W. [Chlamydia trachomatis in patients with genital contact infections in Austria--studies of 3,367 patients]. Wien Klin Wochenschr 1986; 98(20): 679-83.
714. Stengrimsson O, Porarinsson H, Sigfusdottir A, Kolbeinsson A. [Comparison of the occurrence of infections caused by C. trachomatis and N. gonorrhea in Iceland]. Nord Med 1984; 99(8-9): 202-4.
715. Stewart CM, Schoeman SA, Booth RA, Smith SD, Wilcox MH, Wilson JD. Assessment of self taken swabs versus clinician taken swab cultures for diagnosing gonorrhoea in women: single centre, diagnostic accuracy study. Bmj 2012; 345: e8107.
716. Stolte IG, de Wit JB, Kolader M, Fennema H, Coutinho RA, Dukers NH. Association between 'safer sex fatigue' and rectal gonorrhea is mediated by unsafe sex with casual partners among HIV-positive homosexual men. Sex Transm Dis 2006; 33(4): 201-8.
717. Stolte IG, Dukers NH, de Wit JB, Fennema JS, Coutinho RA. Increase in sexually transmitted infections among homosexual men in Amsterdam in relation to HAART. Sex Transm Infect 2001; 77(3): 184-6.
718. Storim J, Verheyen J, Wolff E, et al. Antiretroviral therapy suppresses rectal HIV-RNA shedding despite inflammation in MSM with rectal C. trachomatis and N. gonorrhoeae infections-a cross-sectional, single-center study. Sex Transm Infect 2019; 95(2): 95-8.
719. Stray-Pedersen B, Biørnstad J, Dahl M, et al. Induced abortion: microbiological screening and medical complications. Infection 1991; 19(5): 305-8.
720. Streeck H, Janssen K, Crowell TA, et al. Prospective, multicenter study to assess point prevalence, incidence and recurrence of sexually transmitted infections in men who have sex with men in Germany: BRAHMS study. Journal of the International AIDS Society Conference: 10th IAS Conference on HIV Science Mexico City Mexico 2019; 22(Supplement 5).
721. Streeter T, Cousins D, Ghanem M, Riddell L. Sexual health outreach testing in drug and alcohol services to test for previously undiagnosed blood borne virus infections. International Journal of STD and AIDS 2013; 1): 32.
722. Sugunendran H, Birley HDL, Mallinson H, Abbott M, Tong CYW. Comparison of urine, first and second endourethral swabs for PCR based detection of genital Chlamydia trachomatis infection in male patients. Sexually Transmitted Infections 2001; 77(6): 423-6.
723. Sullivan AK, Elliot E, Singh S, et al. High rates of recreational drug use (RDU) and sexually transmitted infections (STIs) in HIV-positive men who have sex with men (MSM) attending clinics across London. HIV Medicine 2016; 17(Supplement 1): 32.
724. Sultan B, Lincoln S, Ghosh I, et al. High prevalence of sexually transmitted infections, homelessness and drug use among street- based sex workers in London: results from a pilot intervention of mobile outreach testing and treatment. HIV Medicine 2021; 22(S2): 24-119.
725. Sultan B, White JA, Fish R, et al. The "3 in 1" Study: Pooling Self-Taken Pharyngeal, Urethral, and Rectal Samples into a Single Sample for Analysis for Detection of Neisseria gonorrhoeae and Chlamydia trachomatis in Men Who Have Sex with Men. J Clin Microbiol 2016; 54(3): 650-6.

726. Sultan BJ, Fish R, Carrick G, et al. P98 Is pooling of self-taken specimens an effective and acceptable method of testing for sexually transmitted infections in MSMs?: Abstract P98 Table 1. Sexually Transmitted Infections 2012; 88(Suppl 1): A42.3-A3.
727. Surel S, Wenzlova M. A French connection between microhematuria and urethritis. Nephrology Dialysis Transplantation 2012; 2): ii106.
728. Svensson L, Mårdh PA, Weström L. Infertility after acute salpingitis with special reference to Chlamydia trachomatis. Fertil Steril 1983; 40(3): 322-9.
729. Svensson L, Weström L, Mårdh PA. Chlamydia trachomatis in women attending a gynaecological outpatient clinic with lower genital tract infection. Br J Vener Dis 1981; 57(4): 259-62.
730. Svensson L, Westrom L, Ripa KT, Mardh PA. Differences in some clinical and laboratory parameters in acute salpingitis related to culture and serologic findings. Am J Obstet Gynecol 1980; 138(7 Pt 2): 1017-21.
731. Syred J, Holdsworth G, Howroyd C, Spelman K, Baraitser P. Choose to test: self-selected testing for sexually transmitted infections within an online service. Sex Transm Infect 2019; 95(3): 171-4.
732. Szymanowski K, Szumala-Kakol A, Pawlaczyk M, Laczkowska-Pawlaczyk M, Jedrzejczak P. [Bacterial flora of genital organs in women treated for infertility]. Ginekol Pol 1997; 68(1): 41-5.
733. Taipale P, Tarjanne H, Ylostalo P. Transvaginal sonography in suspected pelvic inflammatory disease. Ultrasound Obstet Gynecol 1995; 6(6): 430-4.
734. Talbot MD, Kinghorn GR. Epidemiology and control of gonorrhoea in Sheffield. Genitourin Med 1985; 61(4): 230-3.
735. Taylor-Robinson D, Renton A, Jensen JS, et al. Association of Mycoplasma genitalium with acute non-gonococcal urethritis in Russian men: a comparison with gonococcal and chlamydial urethritis. Int J STD AIDS 2009; 20(4): 234-7.
736. Tchiokadze S, Galdava G. CLINICAL AND ANAMNESTIC CHARACTERISTICS OF DEVELOPMENT OF ANTISPERM IMMUNITY IN INFERTILE MEN. Georgian Med News 2015; (246): 18-22.
737. Tchoudomirova K, Stanilova M, Garov V. Clinical manifestations and diagnosis of bacterial vaginosis in a clinic of sexually transmitted diseases. Folia Med (Plovdiv) 1998; 40(1): 34-40.
738. Teisala K. Sexual behavior and risk for pelvic inflammatory disease. Arch Gynecol Obstet 1988; 243(4): 225-30.
739. Tekgul S, Aktepe O, Sahin A, Ergen A, Remzi D. Genital infections in men associated with Chlamydia trachomatis. Int Urol Nephrol 1992; 24(2): 167-70.
740. Theunissen JJ, Minderhoud-Bassie W, Wagenvoort JH, Stolz E, Michel MF, Huikeshoven FJ. Chlamydia trachomatis-specific antibodies in patients with pelvic inflammatory disease: comparison with isolation in tissue culture or detection with polymerase chain reaction. Genitourin Med 1994; 70(5): 304-7.
741. Thewessen EA, van der Meijden WI, Doppenberg HJ, et al. Screening for cervical Chlamydia trachomatis infections in two Dutch populations. Genitourin Med 1990; 66(5): 361-6.
742. Thin RN, Smith DM. Some characteristics of homosexual men. Br J Vener Dis 1976; 52(3): 161-4.
743. Thin RN, Whatley JD, Blackwell AL. STD and contraception in adolescents. Genitourin Med 1989; 65(3): 157-60.
744. Thorsteinsson K, Ladelund S, Storgaard M, et al. Sexually transmitted infections and use of contraceptives in women living with HIV in Denmark - the SHADE cohort. BMC Infect Dis 2016; 16: 81.
745. Tielemans M, van Westreenen M, Klaassen C, Gotz HM. Confirmatory testing of Neisseria gonorrhoeae in a sexual health clinic: implications for epidemiology and treatment policy. Sex Transm Infect 2021.
746. Tipple C, Rayment M, Mandalia S, et al. An evaluation study of the Becton-Dickinson ProbeTec Qx (BDQx) Trichomonas vaginalis trichomoniasis molecular diagnostic test in two large, urban STD services. Sex Transm Infect 2018; 94(5): 334-6.
747. Tishchenko LD, Kirpatovskiĭ ID, Sakhel MI. [Treatment of epididymitis in infertile men with urogenital Chlamydia infection]. Vestn Dermatol Venerol 1988; (2): 76-7.
748. Tittle V, Alston T, Kinsella R, Jones S, Day S, Asboe D. Sexual health London online testing: A review of service users and outcomes. Sexually Transmitted Infections 2019; 95(Supplement 1): A100.
749. Tjagur S, Mändar R, Punab M. Prevalence of Mycoplasma genitalium and other sexually transmitted infections causing urethritis among high-risk heterosexual male patients in Estonia. Infect Dis (Lond) 2018; 50(2): 133-9.
750. Tjiam KH, van Heijst BY, Polak-Vogelzang AA, et al. Sexually communicable micro-organisms in human semen samples to be used for artificial insemination by donor. Genitourin Med 1987; 63(2): 116-8.
751. Tjiam KH, Zeilmaker GH, Alberda AT, et al. Prevalence of antibodies to Chlamydia trachomatis, Neisseria gonorrhoeae, and Mycoplasma hominis in infertile women. Genitourin Med 1985; 61(3): 175-8.
752. Tomkins A, Ahmad S, Cannon L, et al. Prevalence of recreational drug use reported by men who have sex with men attending sexual health clinics in Manchester, UK. Int J STD AIDS 2018; 29(4): 350-6.
753. Tomlinson DR, Hillman RJ, Harris JR, Taylor-Robinson D. Screening for sexually transmitted disease in London-based male prostitutes. Genitourin Med 1991; 67(2): 103-6.
754. Tomusiak A, Heczko PB, Janeczko J, Adamski P, Pilarczyk-Zurek M, Strus M. Bacterial infections of the lower genital tract in fertile and infertile women from the southeastern Poland. Ginekol Pol 2013; 84(5): 352-8.
755. Torgal-Garcia J, Thabaut A, Durosoir JL, Saliou P, Martin-Bouyer G. [Rarity of men as asymptomatic carriers of Neisseria gonorrhoeae]. Ann Dermatol Venereol 1980; 107(8-9): 829-30.
756. Tortorella C, Piazzolla G, Matteo M, et al. Interleukin-6, interleukin-1beta, and tumor necrosis factor alpha in menstrual effluents as biomarkers of chronic endometritis. Fertil Steril 2014; 101(1): 242-7.
757. Toskine I, Verraes-Derancourt S, Letawe C, et al. [Prevalence of asymptomatic sexually transmitted infections among high-risk patients attending a free anonymous HIV-screening center]. Ann Dermatol Venereol 2004; 131(3): 251-4.
758. Trevisan A, Mengoli C, Rossi L, Cattai M, Cavallaro A. [Epidemiology of reproductive tract infections in a symptomatic population of North-East of Italy]. Minerva Ginecol 2008; 60(2): 135-42.
759. Trotman D, Devlin J, Buss Z, et al. The sensitivity and associated features of culture positive rectal gonorrhoea in men who have sex with men. HIV Medicine 2021; 22(S2): 24-119.
760. Turner K, Clarke B, Priestley C, Scofield S, Chapman C. 'The grand slam': Surveying the local landscape of 'chemsex' drug use amongst MSMS in a community outreach sexual health clinic. Sexually Transmitted Infections 2015; 1): A85.
761. Tuveng JM, Skjeldestad FE, Iversen T. Postabortal insertion of IUD. Adv Contracept 1986; 2(4): 387-92.
762. Tuveng JM, Vold I, Jerve F. Hysterosalpingography: Value in estimating tubal function, and risk of infectious complications. Acta Europaea Fertilitatis 1985; 16(2): 125-8.
763. Tyulenev YA, Guschin AE, Kisina VI. Localization features of sexually transmitted infections in subjectively asymptomatic men who have sex with men. Klinicheskaya dermatologiya i venerologiya 2021; 20(1): 55-61.
764. Ujevic B, Habek J, Habek D. Prevalence of infection with neisseria gonorrhoeae or chlamydia trachomatis in acute mucopurulent cervicitis. Arhiv za Higijenu Rada i Toksikologiju 2009; 60(2): 197-203.
765. Ulla M, Campos A, Baranda J, et al. [Sexually transmitted diseases in a high risk subpopulation from the province of Soria]. Enferm Infecc Microbiol Clin 1993; 11(6): 299-303.

766. Unemo M, Salado-Rasmussen K, Hansen M, et al. Clinical and analytical evaluation of the new Aptima Mycoplasma genitalium assay, with data on M. genitalium prevalence and antimicrobial resistance in M. genitalium in Denmark, Norway and Sweden in 2016. Clin Microbiol Infect 2018; 24(5): 533-9.
767. Unemo M, Savicheva A, Budilovskaya O, Sokolovsky E, Larsson M, Domeika M. Laboratory diagnosis of Neisseria gonorrhoeae in St Petersburg, Russia: inventory, performance characteristics and recommended optimisations. Sex Transm Infect 2006; 82(1): 41-4.
768. Uria MJ, Gonzalez A, Saez E, Ballesta A. Prevalence of Chlamydia trachomatis and Neisseria gonorrhoeae infections among women and men in Madrid, Spain. Clinical Microbiology and Infection 2012; 3): 509.
769. Uzlova TV, Teplova SN, Medvedev BI. [The role of different microorganisms in the genesis of tubal-peritoneal infertility and in immunity]. Zh Mikrobiol Epidemiol Immunobiol 2000; (2): 95-7.
770. Vahidnia A, Costa S, Veenings S, Tuin H, van Loon L, Bliekendaal H. Comparative evaluation of Roche Aurora FLOW, Becton and Dickinson Viper system, and Dynex DS2 for detection of Chlamydia trachomatis, Neisseria gonorrhoeae, and Trichomonas vaginalis in various clinical specimens. Diagn Microbiol Infect Dis 2014; 80(3): 191-2.
771. Vall-Mayans M, Villa M, Saravanya M, et al. Sexually transmitted Chlamydia trachomatis, Neisseria gonorrhoeae, and HIV-1 infections in two at-risk populations in Barcelona: female street prostitutes and STI clinic attendees. Int J Infect Dis 2007; 11(2): 115-22.
772. Valle SL. Sexually transmitted diseases and the use of condoms in a cohort of homosexual men followed since 1983 in Finland. Scand J Infect Dis 1988; 20(2): 153-61.
773. van Aar F, Schreuder I, van Weert Y, Spijker R, Götz H, de Coul EO. Current practices of partner notification among MSM with HIV, gonorrhoea and syphilis in the Netherlands: an urgent need for improvement. BMC infectious diseases 2012; 12(1): 1-11.
774. van Aar F, van Benthem BHB, van den Broek IVF, Gotz HM. STIs in sex partners notified for chlamydia exposure: implications for expedited partner therapy. Sex Transm Infect 2018; 94(8): 619-21.
775. Van Benthem BH, Prins M, Larsen C, Delmas MC, Brunet JB, van den Hoek A. Sexually transmitted infections in European HIV-infected women: incidence in relation to time from infection. European Study on the Natural History of HIV Infection in Women. Aids 2000; 14(5): 595-603.
776. van Bergen JE, Spaargaren J, Götz HM, et al. Population prevalence of Chlamydia trachomatis and Neisseria gonorrhoeae in the Netherlands. Should asymptomatic persons be tested during population-based Chlamydia screening also for gonorrhoea or only if chlamydial infection is found? BMC Infect Dis 2006; 6: 42.
777. Van Bergen JEAM. Increased incidence of gonorrhoea and Chlamydia trachomatis infection in a general practice in south-east Amsterdam, the Netherlands, 1996-2000. Nederlands Tijdschrift voor Geneeskunde 2001; 145(35): 1691-3.
778. van Bergen JEAM, Kerssens JJ, Schellevis FG, Sandfort TG, Coenen TJ, Bindels PJ. Prevalence of STI related consultations in general practice: Results from the second Dutch National Survey of General Practice. British Journal of General Practice 2006; 56(523): 104-9.
779. van de Laar MJ, Termorshuizen F, van den Hoek A. Partner referral by patients with gonorrhea and chlamydial infection. Case-finding observations. Sex Transm Dis 1997; 24(6): 334-42.
780. Van Den Hoek JA, Van Haastrecht HJ, Scheeringa-Troost B, Goudsmit J, Coutinho RA. HIV infection and STD in drug addicted prostitutes in Amsterdam: potential for heterosexual HIV transmission. Genitourin Med 1989; 65(3): 146-50.
781. Van Den Hoek JAR, Klompmaker J. Sexually transmitted diseases in victims of sexual assault. Nederlands Tijdschrift voor Geneeskunde 1986; 130(9): 405-7.
782. Van Der Bij AK, Dukers NHTM, Coutinho RA, Fennema HSA. Low HIV-testing rates and awareness of HIV infection among high-risk heterosexual STI clinic attendees in the Netherlands. European Journal of Public Health 2008; 18(4): 376-9.
783. van der Knaap N, Grady BPX, Schim van der Loeff MF, et al. Drug Users in Amsterdam: Are They Still at Risk for HIV? PLoS ONE 2013; 8(3) (no pagination).
784. van der Lugt B, Drogendijk A, Banffer JR. Prevalence of cervical gonorrhoea in women with unwanted pregnancies. Br J Vener Dis 1980; 56(3): 148-50.
785. Van Der Snoek E, De Wit J, Gotz H, et al. Demographics, Sexual Behaviour and STD/HIV Prevalence in Two Groups of Men Who Have Sex with Men, in Rotterdam, The Netherlands. Acta Dermato-Venereologica 2004; 84(2): 145-50.
786. Van Der Snoek EM, Chin ALRAM, De Ridder MAJ, Willems PWJM, Verkooyen RP, Van Der Meijden WI. The prevalence of veneral disease and HIV infection among persons attending the VD Outpatient Clinic of the Dijkzigt University Hospital in Rotterdam; A comparison of the years 1993 and 1998. [Dutch]. Nederlands Tijdschrift voor Geneeskunde 2000; 144(28): 1351-65.
787. van der Snoek EM, Gotz HM, Mulder PG, Verkooyen RP, van der Meijden WI. Prevalence of STD and HIV infections among attenders of the Erasmus MC STD clinic, Rotterdam, The Netherlands, during the years 1996 to 2000. Int J STD AIDS 2003; 14(2): 119-24.
788. van Doornum GJ, Schouls LM, Pijl A, Cairo I, Buimer M, Bruisten S. Comparison between the LCx Probe system and the COBAS AMPLICOR system for detection of Chlamydia trachomatis and Neisseria gonorrhoeae infections in patients attending a clinic for treatment of sexually transmitted diseases in Amsterdam, The Netherlands. J Clin Microbiol 2001; 39(3): 829-35.
789. Van Duynhoven YT, van de Laar MJ, Schop WA, Mouton JW, van der Meijden WI, Sprenger MJ. Different demographic and sexual correlates for chlamydial infection and gonorrhoea in Rotterdam. Int J Epidemiol 1997; 26(6): 1373-85.
790. van Liere G, Dukers-Muijrers N, Kuizenga-Wessel S, Gotz HM, Hoebe C. What Is the Optimal Testing Strategy for Oropharyngeal Neisseria gonorrhoeae in Men Who Have Sex With Men? Comparing Selective Testing Versus Routine Universal Testing From Dutch Sexually Transmitted Infection Clinic Data (2008-2017). Clin Infect Dis 2020; 71(4): 944-51.
791. van Liere GA, Hoebe CJ, Dukers-Muijrers NH. Evaluation of the anatomical site distribution of chlamydia and gonorrhoea in men who have sex with men and in high-risk women by routine testing: cross-sectional study revealing missed opportunities for treatment strategies. Sex Transm Infect 2014; 90(1): 58-60.
792. van Liere GA, Hoebe CJ, Wolffs PF, Dukers-Muijrers NH. High co-occurrence of anorectal chlamydia with urogenital chlamydia in women visiting an STI clinic revealed by routine universal testing in an observational study; a recommendation towards a better anorectal chlamydia control in women. BMC Infect Dis 2014; 14: 274.
793. van Liere GA, van Rooijen MS, Hoebe CJ, Heijman T, de Vries HJ, Dukers-Muijrers NH. Prevalence of and Factors Associated with Rectal-Only Chlamydia and Gonorrhoea in Women and in Men Who Have Sex with Men. PLoS One 2015; 10(10): e0140297.
794. Van Loo I, Mans C, Hoebe C, Van Westering-Kroon E. High prevalence of ureaplasma spp. In women visiting an STI clinic although no azithromycin resistance was discovered. Sexually Transmitted Infections 2019; 95(Supplement 1): A149.
795. Van Praet JT, Steyaert S, Vandecasteele S, et al. Mycoplasma genitalium acquisition and macrolide resistance after initiation of HIV pre-exposure prophylaxis in men who have sex with men. Sex Transm Infect 2020; 96(6): 396-8.
796. van Rooijen MS, Schim van der Loeff MF, van Kempen L, de Vries HJC. Sexually Transmitted Infection Positivity Rate and Treatment Uptake Among Female and Male Sexual Assault Victims Attending The Amsterdam STI Clinic Between 2005 and 2016. Sex Transm Dis 2018; 45(8): 534-41.
797. Van Rooijen MS, Van Der Loeff MFS, Morre SA, Van Dam AP, Speksnijder AGCL, De Vries HJC. Spontaneous pharyngeal Chlamydia trachomatis RNA clearance. A cross-sectional study followed by a cohort study of untreated STI clinic patients in Amsterdam, the Netherlands. Sexually Transmitted Infections 2015; 91(3): 157-64.
798. van Voorst Vader PC, Lier JG, Posma AL, Schroder FP, Schirm J, Kauer FM. [Chlamydia trachomatis infection in women and the use of oral contraceptives]. Ned Tijdschr Geneeskd 1991; 135(8): 323-7.

799. Vanya M, Szili K, Magori K, Krisztina V. Skin diseases and sexually transmitted infection in a Hungarian prison. Reviews in Medical Microbiology 2017; 28(3): 95-6.
800. Varela JA, Otero L, García MJ, et al. Trends in the prevalence of pathogens causing urethritis in Asturias, Spain, 1989-2000. Sex Transm Dis 2003; 30(4): 280-3.
801. Varma R. Women requiring emergency contraception are a high risk group for sexually transmitted infections in future. Sexually Transmitted Infections Conference: 4th Joint BASHH ASTDA Meeting Brighton United Kingdom Conference Publication: 2012; 88(SUPPL. 1).
802. Vasylyev M, Sluzhynska M, Sluzhynska O, Grushynska O, Krystynyak O. Mobile gynecological clinic - Effective tool for HIV\STI screening diagnostics for commercial sex workers in Lviv Region (Ukraine). Sexually Transmitted Infections Conference: STI and AIDS World Congress 2013; 89(SUPPL. 1).
803. Vázquez F, Palacio V, Vázquez JA, Berrón S, González A, Llaneza JJ. Gonorrhea in women prostitutes: clinical data and auxotypes, serovars, plasmid contents of PPNG, and susceptibility profiles. Sex Transm Dis 1991; 18(1): 5-9.
804. Vera M, Rodriguez C, Perez JA, et al. Toxic habits and high-risk sexual behaviour in newly HIV-diagnosed coinfected with other sexually transmitted diseases. International Journal of STD and AIDS 2015; 1): 37.
805. Verbrugge R, Van Beckhoven D, Sasse A. STI-Surveillance within AIDS reference centres in Belgium - High consistent sti incidence among HIV-positive men having sex with men, 2008-2009. Sexually Transmitted Infections 2011; 1): A137.
806. Vergunst C, Van Der Loeff MS, Van Rooijen M, De Vries H, Bruisten S, Van Dam A. Clinical improvement after standard treatment for urethritis: The role of mycoplasma genitalium. Sexually Transmitted Infections 2019; 95(Supplement 1): A272.
807. Verhaegh-Haasnoot A, Dukers-Muijrers NHTM, Hoebe CJPA. High burden of STI and HIV in male sex workers working as internet escorts for men in an observational study: A hidden key population compared with female sex workers and other men who have sex with men. BMC Infectious Diseases 2015; 15(1) (no pagination).
808. Vernazza PL, Rasi M, Ritzler M, et al. The Swiss STAR trial - an evaluation of target groups for sexually transmitted infection screening in the sub-sample of women. Swiss Med Wkly 2020; 150: w20393.
809. Verneuil L, Vidal JS, Ze Bekolo R, et al. Prevalence and risk factors of the whole spectrum of sexually transmitted diseases in male incoming prisoners in France. Eur J Clin Microbiol Infect Dis 2009; 28(4): 409-13.
810. Verougstraete N, Verbeke V, De Cannière AS, Simons C, Padalko E, Coorevits L. To pool or not to pool? Screening of Chlamydia trachomatis and Neisseria gonorrhoeae in female sex workers: pooled versus single-site testing. Sex Transm Infect 2020; 96(6): 417-21.
811. Verscheijden MMA, Woestenberg PJ, Gotz HM, Van Veen MG, Koedijk FDH, Van Benthem BHB. Sexually transmitted infections among female sex workers tested at STI clinics in the Netherlands, 2006-2013. Emerging Themes in Epidemiology 2015; 12(1) (no pagination).
812. Vica ML, Glevitzky I, Glevitzky M, Siserman CV, Matei HV, Teodoru CA. Antibacterial Activity of Propolis Extracts from the Central Region of Romania against Neisseria gonorrhoeae. Antibiotics (Basel) 2021; 10(6).
813. Vicari E, Di Mauro C, Caruso V, Mongioì A. [Antibiotic therapy in infertile subjects with chronic gonococcal infections: measurement of sperm output]. Arch Ital Urol Nefrol Androl 1991; 63(3): 315-21.
814. Vicari E, Mongioi A, Speciale A. Enhancing detection of gonococcus in ejaculates of adult males using sperm dilution. Archives of Andrology 1986; 16(1): 19-23.
815. Vlaspolder F, Mutsaers JA, Blog F, Notowicz A. Value of a DNA probe assay (Gen-Probe) compared with that of culture for diagnosis of gonococcal infection. J Clin Microbiol 1993; 31(1): 107-10.
816. Vriend HJ, Bogaards JA, van der Klis FRM, et al. Patterns of Human Papillomavirus DNA and Antibody Positivity in Young Males and Females, Suggesting a Site-Specific Natural Course of Infection. PLoS ONE 2013; 8(4) (no pagination).
817. Vu F, Cavassini M, D'Acremont V, et al. Epidemiology of sexually transmitted infections among female sex workers in Switzerland: a local, exploratory, cross-sectional study. Swiss Med Wkly 2020; 150: w20357.
818. Wallin J. Sexually transmitted diseases. The present situation in Sweden. Br J Vener Dis 1978; 54(1): 24-7.
819. Walsh A, Rourke FO, Crowley B. Molecular detection and confirmation of Neisseria gonorrhoeae in urogenital and extragenital specimens using the Abbott CT/NG RealTime assay and an in-house assay targeting the porA pseudogene. Eur J Clin Microbiol Infect Dis 2011; 30(4): 561-7.
820. Walsh APH, Omar AB, Marron KD, Walsh DJ, Salma U, Sills ES. Recipient screening in IVF: First data from women undergoing anonymous oocyte donation in Dublin. Reproductive Health 2011; 8(1) (no pagination).
821. Ward C, Hughes G, Mitchell H, Rogstad K. Associations between repeat attendances, sexually transmitted infections and child sexual exploitation in under 16 year olds attending genitourinary medicine clinics. Sexually Transmitted Infections 2015; 1): A4-A5.
822. Ward C, Lee V. Should we offer routine hepatitis C antibody testing in men who have sex with men? Journal of the International AIDS Society 2014; 3): 69-70.
823. Ward C, Thomas D, Anderson T, Evans R, McQuillan O. Manchester chemsex-injecting straight up the M6. Sexually Transmitted Infections 2016; 92(Supplement 1): A78.
824. Ward H, Day S, Mezzone J, et al. Prostitution and risk of HIV: Female prostitutes in London. British Medical Journal 1993; 307(6900): 356-8.
825. Weller IV, Hindley DJ, Adler MW, Meldrum JT. Gonorrhoea in homosexual men and media coverage of the acquired immune deficiency syndrome in London 1982-3. Br Med J (Clin Res Ed) 1984; 289(6451): 1041.
826. Were J, Mohammed H, Furegato M, Hughes G. Who is being diagnosed with bacterial sexually transmitted infections through partner notification? A comparison of persons diagnosed with chlamydia or gonorrhoea through partner notification and those diagnosed otherwise in genitourinary medicine clinics in England, 2013. International Journal of STD and AIDS 2015; 1): 69.
827. Were J, Mohammed H, Mitchell H, et al. Monitoring partner notification outcomes through national STI surveillance in England. HIV Medicine 2014; 3): 82.
828. Wesbonk J, Chmiel C, Rosemann T, Seidenberg A, Senn O. Prevalence and determinants of sexually transmitted infections in women at risk undergoing abortion in a Swiss primary care setting. Praxis (Bern 1994) 2014; 103(15): 875-82.
829. Whitfield C, Rodgers S. Audit of adherence to UK national guideline for management of epididymo-orchitis. Sexually Transmitted Infections Conference: STI and AIDS World Congress 2013; 89(SUPPL. 1).
830. Wijers J, Dukers-Muijrers N, Hoebe C, Wolffs PFG, van Liere G. The characteristics of patients frequently tested and repeatedly infected with Chlamydia trachomatis in Southwest Limburg, the Netherlands. BMC Public Health 2020; 20(1): 1239.
831. Wilkinson AE. The use of vaginal and rectal cultures in the management of gonorrhea in the female. Br J Vener Dis 1949; 25(1): 9-12.
832. Williams H, Makia F, Bennett M, et al. Comorbidity and late presentation: Findings from an over-fifties cohort. HIV Medicine 2011; 1): 12.
833. Williams S, Scholfield C, Nadarzynski T, Symonds Y, Kidsley S. Acceptability, uptake and impact of online home-sampling for stis in Hampshire, UK: A service evaluation. Sexually Transmitted Infections 2017; 93(Supplement 1): A6.
834. Wilson E, Leyrat C, Baraitser P, Free C. Does internet-accessed STI (e-STI) testing increase testing uptake for chlamydia and other STIs among a young population who have never tested? Secondary analyses of data from a randomised controlled trial. Sex Transm Infect 2019; 95(8): 569-74.
835. Wilson J, Hulme C, Stewart C, Schoeman S, Wilcox M. Cost effectiveness of self-taken vaginal swabs using genprobe AC2 assay vs clinician taken urethral and endocervical swabs for detection of gonorrhoea. Sexually Transmitted Infections 2011; 1): A275.

836. Wilson JD, Wallace HE, Loftus-Keeling M, et al. Swab-yourself trial with economic monitoring and testing for infections collectively (SYSTEMATIC): Part 2. A diagnostic accuracy, and cost-effectiveness, study comparing rectal, pharyngeal and urogenital samples analysed individually, versus as a pooled specimen, for the diagnosis of gonorrhoea and chlamydia. Clinical infectious diseases : an official publication of the Infectious Diseases Society of America 2020; 12.
837. Wilson TE, Uuskula A, Feldman J, Holman S, Dehovitz J. A case-control study of beliefs and behaviors associated with sexually transmitted disease occurrence in Estonia. Sex Transm Dis 2001; 28(11): 624-9.
838. Wind CM, de Vries HJ, Schim van der Loeff MF, Unemo M, van Dam AP. Successful Combination of Nucleic Acid Amplification Test Diagnostics and Targeted Deferred Neisseria gonorrhoeae Culture. J Clin Microbiol 2015; 53(6): 1884-90.
839. Wingrove I, McOwan A, Nwokolo N, Whitlock G. Diagnostics within the clinic to test for gonorrhoea and chlamydia reduces the time to treatment: a service evaluation. Sex Transm Infect 2014; 90(6): 474.
840. Woolley PD, Bowman CA, Kinghorn GR. Prostitution in Sheffield: differences between prostitutes. Genitourin Med 1988; 64(6): 391-3.
841. Young H, Harris AB, Robertson DH. Individual susceptibility to neisserial infection? Br J Vener Dis 1979; 55(3): 188-90.
842. Zarakolu P, Alp S, Yağci S. [Frequency of curable sexually transmitted infections among registered female sex-workers in Ankara City]. Mikrobiyol Bul 2010; 44(1): 117-21.
843. Zarakolu P, Çetik S, İnkaya A, Ünal S. [Comparison of immunochromatographic (IC) and real-time polymerase chain reaction (Rt-PCR) tests for screening Neisseria gonorrhoeae, Chlamydia trachomatis, Ureaplasma spp. and Mycoplasma spp. in HIV-infected men]. Mikrobiyol Bul 2019; 53(1): 37-42.
844. Zvulunov A, Medvedovsky E, Biton A, Horowitz S, Vardy D. Association of Ureaplasma urealyticum colonization in male urethra and Condyloma acuminatum. Isr Med Assoc J 2000; 2(8): 580-2.

**Supplementary Table S3. Number of *Neisseria gonorrhoeae* prevalence measures by European subregion and country/territory.**

| European subregion | European country | Number of *Neisseria gonorrhoeae* prevalence measures |
|---|---|---|
| **Eastern Europe** | Bulgaria | 10 |
| | Czech Republic | 29 |
| | Hungary | 7 |
| | Poland | 10 |
| | Romania | 5 |
| | Russian Federation | 45 |
| | Slovakia | 1 |
| | Ukraine | 6 |
| | **Overall** | **113** |
| **Northern Europe** | Denmark | 74 |
| | Estonia | 9 |
| | Finland | 16 |
| | Greenland | 13 |
| | Iceland | 6 |
| | Ireland | 19 |
| | Latvia | 1 |
| | Lithuania | 1 |
| | Norway | 38 |
| | Sweden | 67 |
| | United Kingdom | 441 |
| | **Overall** | **685** |
| **Southern Europe** | Croatia | 9 |
| | Greece | 7 |
| | Italy | 58 |
| | Portugal | 13 |
| | Slovenia | 10 |
| | Spain | 87 |
| | **Overall** | **184** |
| **Western Europe** | Austria | 15 |
| | Belgium | 27 |
| | France | 87 |
| | Germany | 69 |
| | Netherlands | 289 |
| | Switzerland | 34 |
| | **Overall** | **521** |
| **Intersection of Europe and Asia** | Armenia | 7 |
| | Azerbaijan | 2 |
| | Cyprus | 1 |
| | Georgia | 4 |
| | Tajikistan | 1 |
| | **Overall** | **15** |
| **Other** | Israel | 25 |
| | Turkey | 27 |
| | Mixed regions | 3 |
| | **Overall** | **55** |

**Supplementary Table S4. Summary of precision assessment and risk of bias (ROB) assessment for studies reporting *Neisseria gonorrhoeae* prevalence in Europe.**

| Quality assessment | *Neisseria gonorrhoeae* prevalence measures | |
|---|---|---|
| | **Number of studies** | **%** |
| **Precision of prevalence measures[a]** | | |
| Low precision | 341 | 21.7 |
| High precision | 1,232 | 78.3 |
| **Risk of bias quality domain[b]** | | |
| **Sampling method** | | |
| Low risk of bias | 50 | 3.2 |
| High risk of bias | 1,523 | 96.8 |
| **Response rate** | | |
| Low risk of bias | 93 | 5.9 |
| High risk of bias | 92 | 5.9 |
| Unclear risk of bias | 1,388 | 88.2 |
| **Summary of the risk of bias assessment** | | |
| **Low risk of bias** | | |
| In at least one quality domain | 137 | 8.7 |
| In both quality domains | 6 | 0.4 |
| **High risk of bias** | | |
| In at least one quality domain | 1,540 | 97.9 |
| In both quality domains | 75 | 4.8 |
| **Unclear risk of bias** | | |
| In at least one quality domain | 1,388 | 88.2 |
| In both quality domains | 0 | 0.0 |
| **Prevalence studies where risk of bias assessment was possible** | 1,573 | 100.0 |

[a] Precision was assessed based on the overall sample size (not each stratum subsample size) of the study as reported in the record/publication.
[b] Risk of bias was assessed based on the overall sample size (not each stratum subsample size) of the study as reported in the record/publication.

**Supplementary Figure S1. Forest plots presenting outcomes of the pooled urogenital *Neisseria gonorrhoeae* prevalence among the different populations in Europe.**

**A. General populations**

| Author, Year | Gonorrhea positive | Sample size | Events per 100 observations | W(Random) | Prev(%) | 95% CI |
|---|---|---|---|---|---|---|
| Claeys, 2001 | 9 | 320 | | 0.4% | 2.8 | [ 1.3; 5.3] |
| Avonts, 1985 | 1 | 161 | | 0.4% | 0.6 | [ 0.0; 3.4] |
| Avonts, 1989 | 1 | 248 | | 0.4% | 0.4 | [ 0.0; 2.2] |
| Buekens, 1984 | 2 | 96 | | 0.3% | 2.1 | [ 0.3; 7.3] |
| Buekens, 1984 | 1 | 207 | | 0.4% | 0.5 | [ 0.0; 2.7] |
| Plecko, 2014 | 0 | 49 | | 0.3% | 0.0 | [ 0.0; 7.3] |
| Kacena, 2001 | 0 | 134 | | 0.4% | 0.0 | [ 0.0; 2.7] |
| Kacena, 2001 | 0 | 217 | | 0.4% | 0.0 | [ 0.0; 1.7] |
| Andersen, 1974 | 18 | 847 | | 0.4% | 2.1 | [ 1.3; 3.3] |
| Andersen, 1974 | 5 | 856 | | 0.4% | 0.6 | [ 0.2; 1.4] |
| Bro, 1989 | 1 | 229 | | 0.4% | 0.4 | [ 0.0; 2.4] |
| Gregersen, 1972 | 17 | 759 | | 0.4% | 2.2 | [ 1.3; 3.6] |
| Henriques, 1994 | 2 | 297 | | 0.4% | 0.7 | [ 0.1; 2.4] |
| Jensen, 1979 | 1 | 123 | | 0.3% | 0.8 | [ 0.0; 4.4] |
| Knudsen, 1989 | 1 | 224 | | 0.4% | 0.4 | [ 0.0; 2.5] |
| Nielsen, 1974 | 6 | 190 | | 0.4% | 3.2 | [ 1.2; 6.7] |
| Nielsen, 1974 | 7 | 347 | | 0.4% | 2.0 | [ 0.8; 4.1] |
| Nielsen, 1974 | 9 | 384 | | 0.4% | 2.3 | [ 1.1; 4.4] |
| Nielsen, 1974 | 13 | 353 | | 0.4% | 3.7 | [ 2.0; 6.2] |
| Nielsen, 1974 | 5 | 308 | | 0.4% | 1.6 | [ 0.5; 3.7] |
| Nielsen, 1974 | 5 | 211 | | 0.4% | 2.4 | [ 0.8; 5.4] |
| Nielsen, 1974 | 6 | 700 | | 0.4% | 0.9 | [ 0.3; 1.9] |
| Nielsen, 1974 | 1 | 276 | | 0.4% | 0.4 | [ 0.0; 2.0] |
| Nielsen, 1974 | 0 | 183 | | 0.4% | 0.0 | [ 0.0; 2.0] |
| Nielsen, 1974 | 0 | 82 | | 0.3% | 0.0 | [ 0.0; 4.4] |
| Nielsen, 1974 | 0 | 44 | | 0.2% | 0.0 | [ 0.0; 8.0] |
| Ostergaard, 2000 | 0 | 487 | | 0.4% | 0.0 | [ 0.0; 0.8] |
| Ostergaard, 2000 | 0 | 443 | | 0.4% | 0.0 | [ 0.0; 0.8] |
| Prien-Larsen, 1989 | 2 | 355 | | 0.4% | 0.6 | [ 0.1; 2.0] |
| Skovgaard, 2012 | 49 | 36727 | | 0.5% | 0.1 | [ 0.1; 0.2] |
| Skovgaard, 2012 | 63 | 9055 | | 0.5% | 0.7 | [ 0.5; 0.9] |
| Sorensen, 1992 | 3 | 431 | | 0.4% | 0.7 | [ 0.1; 2.0] |
| Wilson, 2001 | 3 | 112 | | 0.3% | 2.7 | [ 0.6; 7.6] |
| Bayette, 2013 | 9 | 63 | | 0.3% | 14.3 | [ 6.7; 25.4] |
| Bayette, 2013 | 2 | 238 | | 0.4% | 0.8 | [ 0.1; 3.0] |
| Bercot, 2015 | 10 | 93 | | 0.3% | 10.8 | [ 5.3; 18.9] |
| Bourgeois-Nicolaos, 2015 | 4 | 82 | | 0.3% | 4.9 | [ 1.3; 12.0] |
| Bourgeois-Nicolaos, 2015 | 13 | 285 | | 0.4% | 4.6 | [ 2.5; 7.7] |
| Bourgeois-Nicolaos, 2015 | 1 | 222 | | 0.4% | 0.5 | [ 0.0; 2.5] |
| Dao, 2020 | 0 | 209 | | 0.4% | 0.0 | [ 0.0; 1.7] |
| Dautigny, 2013 | 21 | 683 | | 0.4% | 3.1 | [ 1.9; 4.7] |
| Dautigny, 2013 | 12 | 2432 | | 0.4% | 0.5 | [ 0.3; 0.9] |
| Diquelou, 1988 | 0 | 30 | | 0.2% | 0.0 | [ 0.0; 11.6] |
| Ebel, 2015 | 15 | 2201 | | 0.4% | 0.7 | [ 0.4; 1.1] |
| Ebel, 2015 | 35 | 785 | | 0.4% | 4.5 | [ 3.1; 6.1] |
| Jaureguy, 2013 | 19 | 634 | | 0.4% | 3.0 | [ 1.8; 4.6] |
| Benjamin, 1978 | 4 | 646 | | 0.4% | 0.6 | [ 0.2; 1.6] |
| Pereyre, 2017 | 2 | 174 | | 0.4% | 1.1 | [ 0.1; 4.1] |
| Pereyre, 2017 | 3 | 64 | | 0.3% | 4.7 | [ 1.0; 13.1] |
| Pereyre, 2017 | 10 | 333 | | 0.4% | 3.0 | [ 1.4; 5.5] |
| Pereyre, 2017 | 5 | 147 | | 0.4% | 3.4 | [ 1.1; 7.8] |
| Pereyre, 2017 | 2 | 72 | | 0.3% | 2.8 | [ 0.3; 9.7] |
| Pereyre, 2017 | 4 | 104 | | 0.3% | 3.8 | [ 1.1; 9.6] |
| Pereyre, 2017 | 10 | 180 | | 0.4% | 5.6 | [ 2.7; 10.0] |
| Pereyre, 2017 | 5 | 215 | | 0.4% | 2.3 | [ 0.8; 5.3] |
| Pereyre, 2017 | 0 | 112 | | 0.3% | 0.0 | [ 0.0; 3.2] |
| Pereyre, 2017 | 5 | 145 | | 0.4% | 3.4 | [ 1.1; 7.9] |
| Pereyre, 2017 | 15 | 264 | | 0.4% | 5.7 | [ 3.2; 9.2] |
| Pereyre, 2017 | 2 | 307 | | 0.4% | 0.7 | [ 0.1; 2.3] |
| Pereyre, 2017 | 0 | 83 | | 0.3% | 0.0 | [ 0.0; 4.3] |
| Pereyre, 2017 | 1 | 166 | | 0.4% | 0.6 | [ 0.0; 3.3] |
| Pereyre, 2017 | 3 | 150 | | 0.4% | 2.0 | [ 0.4; 5.7] |
| Pereyre, 2017 | 2 | 78 | | 0.3% | 2.6 | [ 0.3; 9.0] |
| Peuchant, 2015a | 5 | 265 | | 0.4% | 1.9 | [ 0.6; 4.3] |
| Peuchant, 2015a | 2 | 112 | | 0.3% | 1.8 | [ 0.2; 6.3] |
| Peuchant, 2015b | 0 | 1004 | | 0.4% | 0.0 | [ 0.0; 0.4] |
| Peuchant, 2015b | 0 | 1004 | | 0.4% | 0.0 | [ 0.0; 0.4] |
| Shafer, 1993 | 14 | 148 | | 0.4% | 9.5 | [ 5.3; 15.4] |
| Torgal-Garcia, 1980 | 0 | 259 | | 0.4% | 0.0 | [ 0.0; 1.4] |
| Dewilde, 1986 | 2 | 263 | | 0.4% | 0.8 | [ 0.1; 2.7] |
| Barthell, 2009 | 1 | 399 | | 0.4% | 0.3 | [ 0.0; 1.4] |
| Bremer, 2013 | 5 | 809 | | 0.4% | 0.6 | [ 0.2; 1.4] |
| Geuenich, 1993 | 7 | 4347 | | 0.5% | 0.2 | [ 0.1; 0.3] |
| Graspeuntner, 2018 | 0 | 89 | | 0.3% | 0.0 | [ 0.0; 4.1] |
| Mesko, 1972 | 7 | 795 | | 0.4% | 0.9 | [ 0.4; 1.8] |
| Skaletz-Rorowski, 2021 | 1 | 250 | | 0.4% | 0.4 | [ 0.0; 2.2] |
| Papanagiotou, 2012 | 0 | 5610 | | 0.5% | 0.0 | [ 0.0; 0.1] |
| Papanagiotou, 2012 | 0 | 2793 | | 0.4% | 0.0 | [ 0.0; 0.1] |
| Gesink, 2012 | 5 | 297 | | 0.4% | 1.7 | [ 0.5; 3.9] |
| Homoe, 2018 | 95 | 690 | | 0.4% | 13.8 | [11.3; 16.6] |
| Homoe, 2018 | 267 | 2421 | | 0.4% | 11.0 | [ 9.8; 12.3] |
| Homoe, 2018 | 227 | 1430 | | 0.4% | 15.9 | [14.0; 17.9] |
| Homoe, 2018 | 255 | 2993 | | 0.4% | 8.5 | [ 7.5; 9.6] |
| Homoe, 2018 | 154 | 1231 | | 0.4% | 12.5 | [10.7; 14.5] |
| Homoe, 2018 | 179 | 2500 | | 0.4% | 7.2 | [ 6.2; 8.2] |
| Homoe, 2018 | 80 | 747 | | 0.4% | 10.7 | [ 8.6; 13.2] |
| Homoe, 2018 | 79 | 1629 | | 0.4% | 4.8 | [ 3.9; 6.0] |
| Homoe, 2018 | 47 | 499 | | 0.4% | 9.4 | [ 7.0; 12.3] |
| Homoe, 2018 | 45 | 946 | | 0.4% | 4.8 | [ 3.5; 6.3] |
| Homoe, 2018 | 21 | 355 | | 0.4% | 5.9 | [ 3.7; 8.9] |
| Homoe, 2018 | 18 | 555 | | 0.4% | 3.2 | [ 1.9; 5.1] |
| Homoe, 2018 | 26 | 326 | | 0.4% | 8.0 | [ 5.3; 11.5] |
| Homoe, 2018 | 19 | 533 | | 0.4% | 3.6 | [ 2.2; 5.5] |
| Homoe, 2018 | 14 | 240 | | 0.4% | 5.8 | [ 3.2; 9.6] |
| Homoe, 2018 | 6 | 349 | | 0.4% | 1.7 | [ 0.6; 3.7] |
| Homoe, 2018 | 11 | 151 | | 0.4% | 7.3 | [ 3.7; 12.7] |
| Homoe, 2018 | 6 | 159 | | 0.4% | 3.8 | [ 1.4; 8.0] |
| Homoe, 2018 | 9 | 122 | | 0.3% | 7.4 | [ 3.4; 13.5] |
| Homoe, 2018 | 3 | 53 | | 0.3% | 5.7 | [ 1.2; 15.7] |

| | | | | | |
|---|---|---|---|---|---|
| Dan, 2003 | 0 | 100 | 0.3% | 0.0 | [ 0.0; 3.6] |
| Camporiondo, 2016 | 0 | 309 | 0.4% | 0.0 | [ 0.0; 1.2] |
| Cevenini, 1982 | 0 | 30 | 0.2% | 0.0 | [ 0.0; 11.6] |
| Cevenini, 1982 | 0 | 106 | 0.3% | 0.0 | [ 0.0; 3.4] |
| Cevenini, 1982 | 0 | 70 | 0.3% | 0.0 | [ 0.0; 5.1] |
| Cevenini, 1982 | 0 | 50 | 0.3% | 0.0 | [ 0.0; 7.1] |
| Del Prete, 2017 | 2 | 1272 | 0.4% | 0.2 | [ 0.0; 0.6] |
| Del Prete, 2017 | 11 | 303 | 0.4% | 3.6 | [ 1.8; 6.4] |
| Frati, 2017 | 1 | 537 | 0.4% | 0.2 | [ 0.0; 1.0] |
| Marcone, 2012 | 0 | 1110 | 0.4% | 0.0 | [ 0.0; 0.3] |
| Matteelli, 2016 | 0 | 1606 | 0.4% | 0.0 | [ 0.0; 0.2] |
| Matteelli, 2016 | 0 | 1112 | 0.4% | 0.0 | [ 0.0; 0.3] |
| Pagani, 2012 | 3 | 233 | 0.4% | 1.3 | [ 0.3; 3.7] |
| Rimoldi, 2012 | 1 | 57 | 0.3% | 1.8 | [ 0.0; 9.4] |
| Sambri, 2017 | 0 | 78 | 0.3% | 0.0 | [ 0.0; 4.6] |
| Barthell, 2009 | 11 | 4624 | 0.5% | 0.2 | [ 0.1; 0.4] |
| Bassiri, 1997 | 9 | 3340 | 0.4% | 0.3 | [ 0.1; 0.5] |
| Geelen, 2013 | 4 | 675 | 0.4% | 0.6 | [ 0.2; 1.5] |
| Gotz, 2006 | 1 | 73 | 0.3% | 1.4 | [ 0.0; 7.4] |
| Gotz, 2006 | 1 | 99 | 0.3% | 1.0 | [ 0.0; 5.5] |
| Heijne, 2019 | 0 | 550 | 0.4% | 0.0 | [ 0.0; 0.7] |
| Jenniskens, 2017 | 27 | 5628 | 0.5% | 0.5 | [ 0.3; 0.7] |
| Op de Coul, 2021 | 2 | 548 | 0.4% | 0.4 | [ 0.0; 1.3] |
| Op de Coul, 2021 | 1 | 425 | 0.4% | 0.2 | [ 0.0; 1.3] |
| Querido, 1980 | 3 | 1021 | 0.4% | 0.3 | [ 0.1; 0.9] |
| Thewessen, 1990 | 2 | 557 | 0.4% | 0.4 | [ 0.0; 1.3] |
| Tjiam, 1987 | 0 | 237 | 0.4% | 0.0 | [ 0.0; 1.5] |
| Van Bergen, 2006a | 0 | 605 | 0.4% | 0.0 | [ 0.0; 0.6] |
| Van Der Lugt, 1980 | 4 | 340 | 0.4% | 1.2 | [ 0.3; 3.0] |
| Van Der Lugt, 1980 | 7 | 740 | 0.4% | 0.9 | [ 0.4; 1.9] |
| Van Der Lugt, 1980 | 2 | 608 | 0.4% | 0.3 | [ 0.0; 1.2] |
| Anestad, 1984 | 0 | 43 | 0.2% | 0.0 | [ 0.0; 8.2] |
| Anestad, 1984 | 0 | 68 | 0.3% | 0.0 | [ 0.0; 5.3] |
| Falk, 1993 | 1238 | 25938 | 0.5% | 4.8 | [ 4.5; 5.0] |
| Falk, 1993 | 1048 | 25901 | 0.5% | 4.0 | [ 3.8; 4.3] |
| Falk, 1993 | 988 | 23383 | 0.5% | 4.2 | [ 4.0; 4.5] |
| Falk, 1993 | 952 | 23415 | 0.5% | 4.1 | [ 3.8; 4.3] |
| Falk, 1993 | 727 | 22946 | 0.5% | 3.2 | [ 2.9; 3.4] |
| Falk, 1993 | 353 | 23104 | 0.5% | 1.5 | [ 1.4; 1.7] |
| Falk, 1993 | 204 | 23281 | 0.5% | 0.9 | [ 0.8; 1.0] |
| Falk, 1993 | 135 | 24445 | 0.5% | 0.6 | [ 0.5; 0.7] |
| Falk, 1993 | 68 | 26144 | 0.5% | 0.3 | [ 0.2; 0.3] |
| Falk, 1993 | 320 | 12565 | 0.5% | 2.5 | [ 2.3; 2.8] |
| Falk, 1993 | 454 | 15861 | 0.5% | 2.9 | [ 2.6; 3.1] |
| Falk, 1993 | 298 | 17677 | 0.5% | 1.7 | [ 1.5; 1.9] |
| Falk, 1993 | 228 | 17072 | 0.5% | 1.3 | [ 1.2; 1.5] |
| Falk, 1993 | 95 | 17039 | 0.5% | 0.6 | [ 0.5; 0.7] |
| Falk, 1993 | 54 | 19004 | 0.5% | 0.3 | [ 0.2; 0.4] |
| Falk, 1993 | 29 | 20125 | 0.5% | 0.1 | [ 0.1; 0.2] |
| Qvigstad, 1983 | 5 | 557 | 0.4% | 0.9 | [ 0.3; 2.1] |
| Skjeldestad, 1987 | 2 | 599 | 0.4% | 0.3 | [ 0.0; 1.2] |
| Skjeldestad, 1992 | 8 | 1702 | 0.4% | 0.5 | [ 0.2; 0.9] |
| Stray-Pedersen, 1991 | 10 | 1193 | 0.4% | 0.8 | [ 0.4; 1.5] |
| Tuveng, 1986 | 0 | 11 | 0.1% | 0.0 | [ 0.0; 28.5] |
| Tuveng, 1986 | 0 | 34 | 0.2% | 0.0 | [ 0.0; 10.3] |
| Tomusiak, 2013 | 0 | 60 | 0.3% | 0.0 | [ 0.0; 6.0] |
| Borges-Costa, 2012 | 10 | 204 | 0.4% | 4.9 | [ 2.4; 8.8] |
| Silva, 2021 | 3 | 224 | 0.4% | 1.3 | [ 0.3; 3.9] |
| Silva, 2021 | 2 | 130 | 0.4% | 1.5 | [ 0.2; 5.4] |
| Silva, 2021 | 1 | 182 | 0.4% | 0.5 | [ 0.0; 3.0] |
| Silva, 2021 | 1 | 51 | 0.3% | 2.0 | [ 0.0; 10.4] |
| Silva, 2021 | 1 | 37 | 0.2% | 2.7 | [ 0.1; 14.2] |
| Silva, 2021 | 1 | 56 | 0.3% | 1.8 | [ 0.0; 9.6] |
| Miron, 2013 | 1 | 45 | 0.2% | 2.2 | [ 0.1; 11.8] |
| Detels, 2011 | 4 | 2212 | 0.4% | 0.2 | [ 0.0; 0.5] |
| Frolova, 2013 | 3 | 403 | 0.4% | 0.7 | [ 0.2; 2.2] |
| Shipitsyna, 2013 | 6 | 1053 | 0.4% | 0.6 | [ 0.2; 1.2] |
| Shipitsyna, 2013 | 0 | 154 | 0.4% | 0.0 | [ 0.0; 2.4] |
| Shipitsyna, 2020 | 4 | 2598 | 0.4% | 0.2 | [ 0.0; 0.4] |
| klavs, 2019 | 0 | 430 | 0.4% | 0.0 | [ 0.0; 0.9] |
| klavs, 2019 | 0 | 593 | 0.4% | 0.0 | [ 0.0; 0.6] |
| Criado, 2019 | 2 | 71 | 0.3% | 2.8 | [ 0.3; 9.8] |
| Franceschi, 2007 | 0 | 157 | 0.4% | 0.0 | [ 0.0; 2.3] |
| Franceschi, 2007 | 0 | 327 | 0.4% | 0.0 | [ 0.0; 1.1] |
| Rodriguez, 2001 | 2 | 111 | 0.3% | 1.8 | [ 0.2; 6.4] |
| Arvidson, 1995 | 15 | 120 | 0.3% | 12.5 | [ 7.2; 19.8] |
| Arvidson, 1995 | 27 | 876 | 0.4% | 3.1 | [ 2.0; 4.5] |
| Arvidson, 1995 | 24 | 595 | 0.4% | 4.0 | [ 2.6; 5.9] |
| Bryman, 1988 | 0 | 130 | 0.4% | 0.0 | [ 0.0; 2.8] |
| Bryman, 1988 | 0 | 115 | 0.3% | 0.0 | [ 0.0; 3.2] |
| Frolund, 2011 | 0 | 30 | 0.2% | 0.0 | [ 0.0; 11.6] |
| Giertz, 1987 | 2 | 560 | 0.4% | 0.4 | [ 0.0; 1.3] |
| Hellberg, 1999 | 25 | 192 | 0.4% | 13.0 | [ 8.6; 18.6] |
| Hellberg, 1999 | 17 | 792 | 0.4% | 2.1 | [ 1.3; 3.4] |
| Nolskog, 2019 | 1 | 1001 | 0.4% | 0.1 | [ 0.0; 0.6] |
| Svensson, 1983 | 1 | 20 | 0.2% | 5.0 | [ 0.1; 24.9] |
| Svensson, 1983 | 0 | 10 | 0.1% | 0.0 | [ 0.0; 30.8] |
| Svensson, 1983 | 1 | 19 | 0.2% | 5.3 | [ 0.1; 26.0] |
| Wesbonk, 2014 | 2 | 192 | 0.4% | 1.0 | [ 0.1; 3.7] |
| Jamalova, 1999 | 1 | 400 | 0.4% | 0.2 | [ 0.0; 1.4] |
| Acik, 2004 | 1 | 60 | 0.3% | 1.7 | [ 0.0; 8.9] |
| Dolapci, 2005 | 2 | 58 | 0.3% | 3.4 | [ 0.4; 11.9] |
| Eksi, 2011 | 0 | 97 | 0.3% | 0.0 | [ 0.0; 3.7] |
| Eksi, 2011 | 0 | 97 | 0.3% | 0.0 | [ 0.0; 3.7] |
| Genc, 1993 | 0 | 190 | 0.4% | 0.0 | [ 0.0; 1.9] |
| Hassan, 2016 | 0 | 255 | 0.4% | 0.0 | [ 0.0; 1.4] |
| Hodoglugil, 1999 | 18 | 410 | 0.4% | 4.4 | [ 2.6; 6.8] |
| Hodoglugil, 2000 | 13 | 211 | 0.4% | 6.2 | [ 3.3; 10.3] |
| Hodoglugil, 2000 | 4 | 155 | 0.4% | 2.6 | [ 0.7; 6.5] |
| Kanbur, 2018 | 4 | 386 | 0.4% | 1.0 | [ 0.3; 2.6] |
| Ortayli, 2001 | 0 | 569 | 0.4% | 0.0 | [ 0.0; 0.6] |
| Tekgul, 1992 | 0 | 26 | 0.2% | 0.0 | [ 0.0; 13.2] |
| Adler, 1981 | 0 | 528 | 0.4% | 0.0 | [ 0.0; 0.7] |
| Adler, 1981 | 3 | 903 | 0.4% | 0.3 | [ 0.1; 1.0] |
| Adler, 1981 | 2 | 480 | 0.4% | 0.4 | [ 0.1; 1.5] |
| Adler, 1981 | 0 | 300 | 0.4% | 0.0 | [ 0.0; 1.2] |
| Bartholomew, 2021 | 0 | 267 | 0.4% | 0.0 | [ 0.0; 1.4] |
| Bartholomew, 2021 | 3 | 236 | 0.4% | 1.3 | [ 0.3; 3.7] |
| Blackwell, 1993 | 1 | 400 | 0.4% | 0.2 | [ 0.0; 1.4] |
| Dhairyawan, 2012 | 0 | 430 | 0.4% | 0.0 | [ 0.0; 0.9] |
| Duthie, 1987 | 0 | 167 | 0.4% | 0.0 | [ 0.0; 2.2] |
| Fowler, 2013 | 59 | 18044 | 0.5% | 0.3 | [ 0.2; 0.4] |
| Fowler, 2013 | 174 | 41873 | 0.5% | 0.4 | [ 0.4; 0.5] |
| Gillespie, 2000 | 2 | 1630 | 0.4% | 0.1 | [ 0.0; 0.4] |
| Gillespie, 2000 | 2 | 1742 | 0.4% | 0.1 | [ 0.0; 0.4] |
| Gillespie, 2000 | 0 | 1622 | 0.4% | 0.0 | [ 0.0; 0.2] |
| Harindra, 2002 | 2 | 805 | 0.4% | 0.2 | [ 0.0; 0.9] |
| Harindra, 2002 | 3 | 255 | 0.4% | 1.2 | [ 0.2; 3.4] |
| Hay, 2016 | 30 | 1908 | 0.4% | 1.6 | [ 1.1; 2.2] |
| Hilton, 1974 | 0 | 63 | 0.3% | 0.0 | [ 0.0; 5.7] |
| Kerry-Barnard, 2020 | 3 | 108 | 0.3% | 2.8 | [ 0.6; 7.9] |
| Kerry-Barnard, 2020 | 0 | 94 | 0.3% | 0.0 | [ 0.0; 3.8] |

| | | | | | |
|---|---|---|---|---|---|
| Mahto, 2011 | 0 | 382 | 0.4% | 0.0 | [ 0.0; 1.0] |
| Nicholls, 2018 | 12 | 3573 | 0.4% | 0.3 | [ 0.2; 0.6] |
| Oakeshott, 2010 | 8 | 2369 | 0.4% | 0.3 | [ 0.1; 0.7] |
| Oakeshott, 2019 | 3 | 259 | 0.4% | 1.2 | [ 0.2; 3.3] |
| Oakeshott, 2019 | 0 | 250 | 0.4% | 0.0 | [ 0.0; 1.5] |
| Pearson, 1988 | 0 | 22 | 0.2% | 0.0 | [ 0.0; 15.4] |
| Penney, 1998 | 1 | 392 | 0.4% | 0.3 | [ 0.0; 1.4] |
| Penney, 1998 | 2 | 1207 | 0.4% | 0.2 | [ 0.0; 0.6] |
| Riordan, 1990 | 2 | 162 | 0.4% | 1.2 | [ 0.1; 4.4] |
| Robertson, 1970 | 9 | 158 | 0.4% | 5.7 | [ 2.6; 10.5] |
| Robertson, 1970 | 1 | 34 | 0.2% | 2.9 | [ 0.1; 15.3] |
| Robertson, 1970 | 1 | 63 | 0.3% | 1.6 | [ 0.0; 8.5] |
| Robertson, 1970 | 3 | 158 | 0.4% | 1.9 | [ 0.4; 5.4] |
| Robertson, 1970 | 1 | 34 | 0.2% | 2.9 | [ 0.1; 15.3] |
| Robertson, 1970 | 1 | 63 | 0.3% | 1.6 | [ 0.0; 8.5] |
| Singha, 1979 | 2 | 100 | 0.3% | 2.0 | [ 0.2; 7.0] |
| Singha, 1979 | 0 | 119 | 0.3% | 0.0 | [ 0.0; 3.1] |
| Smith, 1991 | 2 | 197 | 0.4% | 1.0 | [ 0.1; 3.6] |
| Sonnenberg, 2013 | 0 | 171 | 0.4% | 0.0 | [ 0.0; 2.1] |
| Sonnenberg, 2013 | 0 | 224 | 0.4% | 0.0 | [ 0.0; 1.6] |
| Sonnenberg, 2013 | 1 | 597 | 0.4% | 0.2 | [ 0.0; 0.9] |
| Sonnenberg, 2013 | 0 | 1146 | 0.4% | 0.0 | [ 0.0; 0.3] |
| Sonnenberg, 2013 | 0 | 527 | 0.4% | 0.0 | [ 0.0; 0.7] |
| Sonnenberg, 2013 | 0 | 150 | 0.4% | 0.0 | [ 0.0; 2.4] |
| Sonnenberg, 2013 | 0 | 193 | 0.4% | 0.0 | [ 0.0; 1.9] |
| Sonnenberg, 2013 | 1 | 497 | 0.4% | 0.2 | [ 0.0; 1.1] |
| Sonnenberg, 2013 | 0 | 693 | 0.4% | 0.0 | [ 0.0; 0.5] |
| Sonnenberg, 2013 | 0 | 352 | 0.4% | 0.0 | [ 0.0; 1.0] |
| Southgate, 1983 | 4 | 248 | 0.4% | 1.6 | [ 0.4; 4.1] |
| Sprague, 1990 | 0 | 286 | 0.4% | 0.0 | [ 0.0; 1.3] |
| Sprague, 1990 | 0 | 236 | 0.4% | 0.0 | [ 0.0; 1.6] |
| Tittle, 2019 | 726 | 64907 | 0.5% | 1.1 | [ 1.0; 1.2] |
| Surel, 2012 | 1 | 280 | 0.4% | 0.4 | [ 0.0; 2.0] |
| Surel, 2012 | 0 | 202 | 0.4% | 0.0 | [ 0.0; 1.8] |
| Schabereiter-Gurtner, 2008 | 11 | 1313 | 0.4% | 0.8 | [ 0.4; 1.5] |
| Kourbatova, 2008 | 0 | 1000 | 0.4% | 0.0 | [ 0.0; 0.4] |
| Kourbatova, 2008 | 3 | 750 | 0.4% | 0.4 | [ 0.1; 1.2] |
| Kourbatova, 2008 | 0 | 256 | 0.4% | 0.0 | [ 0.0; 1.4] |
| Low, 2003 | 2 | 109 | 0.3% | 1.8 | [ 0.2; 6.5] |
| Low, 2003 | 0 | 23 | 0.2% | 0.0 | [ 0.0; 14.8] |
| Halvorsen, 1992 | 0 | 517 | 0.4% | 0.0 | [ 0.0; 0.7] |
| **Common effect model** | **10643** | **644913** | **--** | **0.9** | **[ 0.9; 0.9]** |
| **Random effects model** | | | **100.0%** | **1.0** | **[ 0.7; 1.2]** |
| **Prediction interval** | | | | | **[ 0.0; 6.9]** |

Heterogeneity: $I^2 = 98\%$, $\chi^2_{261} = 12242.65$ ($p = 0$)

0 20 40 60 80 100
Gonorrhea prevalence (95% CI)

## B. Intermediate-risk populations

| Author, Year | Gonorrhea positive | Sample size | W(Random) | Prev(%) | 95% CI |
|---|---|---|---|---|---|
| Amirkhanian, 2013 | 15 | 405 | 4.4% | 3.7 | [ 2.1; 6.0] |
| Kabakchieva, 2006 | 13 | 286 | 4.3% | 4.5 | [ 2.4; 7.6] |
| David Parker, 2015 | 5 | 584 | 4.4% | 0.9 | [ 0.3; 2.0] |
| Parker, 2017 | 0 | 186 | 4.3% | 0.0 | [ 0.0; 2.0] |
| Diquelou, 1988 | 0 | 20 | 3.2% | 0.0 | [ 0.0; 16.8] |
| Duron, 2018 | 1 | 925 | 4.4% | 0.1 | [ 0.0; 0.6] |
| Verneuil, 2009 | 0 | 400 | 4.4% | 0.0 | [ 0.0; 0.9] |
| Gyarmathy, 2009 | 0 | 186 | 4.3% | 0.0 | [ 0.0; 2.0] |
| Herman, 1985 | 0 | 80 | 4.0% | 0.0 | [ 0.0; 4.5] |
| Samra, 1991 | 0 | 51 | 3.8% | 0.0 | [ 0.0; 7.0] |
| Solomon, 1970 | 10 | 65 | 4.0% | 15.4 | [ 7.6; 26.5] |
| Van Der Knaap, 2013 | 0 | 181 | 4.3% | 0.0 | [ 0.0; 2.0] |
| Shakarishvili, 2005 | 88 | 401 | 4.4% | 21.9 | [18.0; 26.3] |
| Shakarishvili, 2005 | 71 | 240 | 4.3% | 29.6 | [23.9; 35.8] |
| Shakarishvili, 2005 | 63 | 372 | 4.4% | 16.9 | [13.3; 21.1] |
| Kacena, 2001 | 0 | 128 | 4.2% | 0.0 | [ 0.0; 2.8] |
| Folch, 2011 | 5 | 748 | 4.4% | 0.7 | [ 0.2; 1.6] |
| Andersson, 2021a | 0 | 117 | 4.2% | 0.0 | [ 0.0; 3.1] |
| Andersson, 2021a | 0 | 118 | 4.2% | 0.0 | [ 0.0; 3.1] |
| Kamwendo, 1993 | 8 | 200 | 4.3% | 4.0 | [ 1.7; 7.7] |
| Arumainayagam, 1991 | 1 | 100 | 4.1% | 1.0 | [ 0.0; 5.4] |
| Mahto, 2011 | 1 | 269 | 4.3% | 0.4 | [ 0.0; 2.1] |
| Sultan, 2021 | 7 | 23 | 3.3% | 30.4 | [13.2; 52.9] |
| Mahto, 2008 | 1 | 474 | 4.4% | 0.2 | [ 0.0; 1.2] |
| **Common effect model** | **289** | **6559** | **--** | **1.8** | **[ 1.5; 2.2]** |
| **Random effects model** | | | **100.0%** | **2.4** | **[ 0.5; 5.2]** |
| **Prediction interval** | | | | | **[ 0.0; 25.7]** |

Heterogeneity: $I^2 = 97\%$, $\chi^2_{23} = 680.52$ ($p < 0.01$)

Events per 100 observations

0 20 40 60 80 100
Gonorrhea prevalence (95% CI)

## C. Infertility clinic attendees

| Author, Year | Gonorrhea positive | Sample size | W(Random) | Prev(%) | 95% CI |
|---|---|---|---|---|---|
| Oganesian, 1990 | 43 | 200 | 2.2% | 21.5 | [16.0; 27.8] |
| Oganesian, 1990 | 45 | 200 | 2.2% | 22.5 | [16.9; 28.9] |
| Dietrich, 2010 | 0 | 40 | 1.8% | 0.0 | [ 0.0; 8.8] |
| Dietrich, 2010 | 0 | 162 | 2.2% | 0.0 | [ 0.0; 2.3] |
| Dolenec, 1980 | 1 | 61 | 1.9% | 1.6 | [ 0.0; 8.8] |
| Plecko, 2014 | 1 | 145 | 2.1% | 0.7 | [ 0.0; 3.8] |
| Klanica, 1978 | 21 | 63 | 1.9% | 33.3 | [22.0; 46.3] |
| Klanica, 1978 | 15 | 63 | 1.9% | 23.8 | [14.0; 36.2] |
| Kjaergaard, 1997 | 0 | 201 | 2.2% | 0.0 | [ 0.0; 1.8] |
| Moller, 1984 | 0 | 116 | 2.1% | 0.0 | [ 0.0; 3.1] |
| Alexandre, 1971 | 38 | 534 | 2.3% | 7.1 | [ 5.1; 9.6] |
| Diquelou, 1988 | 0 | 80 | 2.0% | 0.0 | [ 0.0; 4.5] |
| Guillet-Rosso, 1987 | 0 | 519 | 2.3% | 0.0 | [ 0.0; 0.7] |
| Henry-Suchet, 1980 | 0 | 17 | 1.3% | 0.0 | [ 0.0; 19.5] |
| Henry-Suchet, 1980 | 0 | 29 | 1.6% | 0.0 | [ 0.0; 11.9] |
| Henry-Suchet, 1980 | 0 | 36 | 1.7% | 0.0 | [ 0.0; 9.7] |
| Henry-Suchet, 1983 | 0 | 16 | 1.3% | 0.0 | [ 0.0; 20.6] |
| Eggert-Kruse, 1987 | 0 | 233 | 2.2% | 0.0 | [ 0.0; 1.6] |
| Eggert-Kruse, 1987 | 0 | 230 | 2.2% | 0.0 | [ 0.0; 1.6] |
| Eggert-Kruse, 1995 | 0 | 163 | 2.2% | 0.0 | [ 0.0; 2.2] |
| Eggert-Kruse, 2015 | 0 | 128 | 2.1% | 0.0 | [ 0.0; 2.8] |
| Graspeuntner, 2018 | 0 | 26 | 1.6% | 0.0 | [ 0.0; 13.2] |
| Hofmann, 1985 | 0 | 121 | 2.1% | 0.0 | [ 0.0; 3.0] |
| Hofmann, 1985 | 0 | 121 | 2.1% | 0.0 | [ 0.0; 3.0] |
| Walsh, 2011a | 0 | 225 | 2.2% | 0.0 | [ 0.0; 1.6] |
| Cevenini, 1982 | 0 | 30 | 1.6% | 0.0 | [ 0.0; 11.6] |
| Lucisano, 1992 | 0 | 42 | 1.8% | 0.0 | [ 0.0; 8.4] |
| Lucisano, 1992 | 0 | 41 | 1.8% | 0.0 | [ 0.0; 8.6] |
| Ruozi Berretta, 1974 | 17 | 137 | 2.1% | 12.4 | [ 7.4; 19.1] |
| Semmola, 1967 | 2 | 55 | 1.9% | 3.6 | [ 0.4; 12.5] |
| Semmola, 1967 | 1 | 63 | 1.9% | 1.6 | [ 0.0; 8.5] |
| Semmola, 1967 | 0 | 152 | 2.1% | 0.0 | [ 0.0; 2.4] |
| Tortorella, 2014 | 0 | 64 | 1.9% | 0.0 | [ 0.0; 5.6] |
| Vicari, 1986 | 9 | 90 | 2.0% | 10.0 | [ 4.7; 18.1] |
| Vicari, 1991 | 111 | 785 | 2.3% | 14.1 | [11.8; 16.8] |
| Tuveng, 1985 | 0 | 22 | 1.5% | 0.0 | [ 0.0; 15.4] |
| Tuveng, 1985 | 0 | 58 | 1.9% | 0.0 | [ 0.0; 6.2] |
| Mierzejewski, 1987 | 1 | 61 | 1.9% | 1.6 | [ 0.0; 8.8] |
| Szymanowski, 1997 | 0 | 72 | 2.0% | 0.0 | [ 0.0; 5.0] |
| Tomusiak, 2013 | 0 | 101 | 2.1% | 0.0 | [ 0.0; 3.6] |
| Mares, 2009 | 1 | 125 | 2.1% | 0.8 | [ 0.0; 4.4] |
| Miron, 2013 | 1 | 176 | 2.2% | 0.6 | [ 0.0; 3.1] |
| Uzlova, 2000 | 1 | 363 | 2.2% | 0.3 | [ 0.0; 1.5] |
| Rodriguez, 2001 | 1 | 376 | 2.3% | 0.3 | [ 0.0; 1.5] |
| Rodriguez, 2003 | 1 | 281 | 2.2% | 0.4 | [ 0.0; 2.0] |
| Akgul, 2018 | 0 | 25 | 1.5% | 0.0 | [ 0.0; 13.7] |
| Akgul, 2018 | 0 | 25 | 1.5% | 0.0 | [ 0.0; 13.7] |
| Eksi, 2011 | 0 | 98 | 2.1% | 0.0 | [ 0.0; 3.7] |
| Eksi, 2011 | 1 | 98 | 2.1% | 1.0 | [ 0.0; 5.6] |
| Eldivan, 2016 | 0 | 60 | 1.9% | 0.0 | [ 0.0; 6.0] |
| Hinton, 1979 | 0 | 21 | 1.4% | 0.0 | [ 0.0; 16.1] |
| **Common effect model** | **311** | **7150** | -- | **1.6** | **[ 1.3; 2.0]** |
| **Random effects model** | | | **100.0%** | **1.1** | **[ 0.3; 2.4]** |
| **Prediction interval** | | | | | **[ 0.0; 15.7]** |

Heterogeneity: $I^2 = 93\%$, $\chi^2_{50} = 704.64$ ($p < 0.01$)

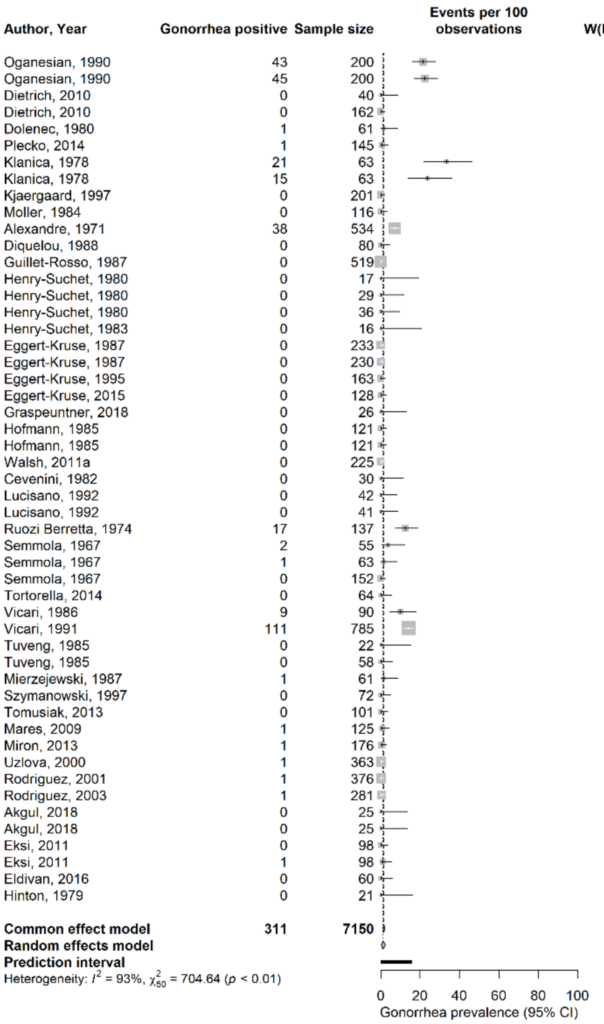


## D. Other populations

| Author, Year | Gonorrhea positive | Sample size | W(Random) | Prev(%) | 95% CI |
|---|---|---|---|---|---|
| Edouard, 2017b | 64 | 2163 | 4.2% | 3.0 | [ 2.3; 3.8] |
| Edouard, 2017b | 0 | 58 | 3.5% | 0.0 | [ 0.0; 6.2] |
| Jaureguy, 2016 | 19 | 274 | 4.0% | 6.9 | [ 4.2; 10.6] |
| Loubinoux, 2012 | 5 | 840 | 4.1% | 0.6 | [ 0.2; 1.4] |
| Loubinoux, 2012 | 6 | 433 | 4.1% | 1.4 | [ 0.5; 3.0] |
| Loubinoux, 2012 | 5 | 407 | 4.1% | 1.2 | [ 0.4; 2.8] |
| Morgat, 2021 | 3 | 189 | 4.0% | 1.6 | [ 0.3; 4.6] |
| Rolskov, 2015 | 190 | 1748 | 4.2% | 10.9 | [ 9.4; 12.4] |
| Rolskov, 2015 | 232 | 3688 | 4.2% | 6.3 | [ 5.5; 7.1] |
| Rolskov, 2015 | 396 | 3346 | 4.2% | 11.8 | [10.8; 13.0] |
| Rolskov, 2015 | 424 | 5685 | 4.2% | 7.5 | [ 6.8; 8.2] |
| Matinato, 2012 | 32 | 461 | 4.1% | 6.9 | [ 4.8; 9.7] |
| Napoli, 2013 | 22 | 300 | 4.1% | 7.3 | [ 4.7; 10.9] |
| Napoli, 2013 | 4 | 300 | 4.1% | 1.3 | [ 0.4; 3.4] |
| Van Rooijen, 2018 | 15 | 1063 | 4.2% | 1.4 | [ 0.8; 2.3] |
| Van Rooijen, 2018 | 1 | 143 | 3.9% | 0.7 | [ 0.0; 3.8] |
| Dahl, 1974 | 12 | 59 | 3.6% | 20.3 | [11.0; 32.8] |
| Dahl, 1974 | 8 | 59 | 3.6% | 13.6 | [ 6.0; 25.0] |
| Dahl, 1974 | 26 | 59 | 3.6% | 44.1 | [31.2; 57.6] |
| Hagemann, 2014 | 0 | 300 | 4.1% | 0.0 | [ 0.0; 1.2] |
| Domeika, 2011 | 12 | 446 | 4.1% | 2.7 | [ 1.4; 4.7] |
| Orellana, 2011a | 3 | 377 | 4.1% | 0.8 | [ 0.2; 2.3] |
| Cherkaoui, 2019 | 6 | 463 | 4.1% | 1.3 | [ 0.5; 2.8] |
| Popert, 1964 | 20 | 82 | 3.7% | 24.4 | [15.6; 35.1] |
| Pulvirenti, 2015 | 8 | 772 | 4.1% | 1.0 | [ 0.4; 2.0] |
| **Common effect model** | **1513** | **23715** | -- | **5.4** | **[ 5.2; 5.7]** |
| **Random effects model** | | | **100.0%** | **4.7** | **[ 2.4; 7.7]** |
| **Prediction interval** | | | | | **[ 0.0; 26.8]** |

Heterogeneity: $I^2 = 97\%$, $\chi^2_{24} = 822.34$ ($p < 0.01$)

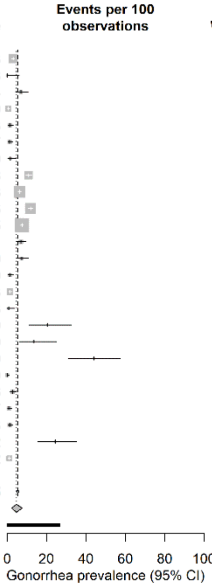

## E. Female sex workers

| Author, Year | Gonorrhea positive | Sample size | W(Random) | Prev(%) | 95% CI |
|---|---|---|---|---|---|
| Coorevits, 2018 | 7 | 299 | 3.7% | 2.3 | [ 0.9; 4.8] |
| Verougstraete, 2020 | 10 | 489 | 3.9% | 2.0 | [ 1.0; 3.7] |
| Kacena, 2001 | 1 | 35 | 2.3% | 2.9 | [ 0.1; 14.9] |
| Geuenich, 1993 | 4 | 386 | 3.8% | 1.0 | [ 0.3; 2.6] |
| Hofmann, 1985 | 32 | 548 | 3.9% | 5.8 | [ 4.0; 8.1] |
| Hofmann, 1985 | 89 | 548 | 3.9% | 16.2 | [13.3; 19.6] |
| Hofmann, 1985 | 62 | 1680 | 4.0% | 3.7 | [ 2.8; 4.7] |
| Hofmann, 1985 | 104 | 1680 | 4.0% | 6.2 | [ 5.1; 7.5] |
| Jansen, 2013 | 14 | 131 | 3.4% | 10.7 | [ 6.0; 17.3] |
| Jansen, 2013 | 4 | 66 | 2.9% | 6.1 | [ 1.7; 14.8] |
| Jansen, 2013 | 2 | 3 | 0.5% | 66.7 | [ 9.4; 99.2] |
| Klingenberg, 2016 | 4 | 112 | 3.3% | 3.6 | [ 1.0; 8.9] |
| Papadogeorgaki, 2006 | 19 | 299 | 3.7% | 6.4 | [ 3.9; 9.7] |
| Cwikel, 2006 | 0 | 41 | 2.5% | 0.0 | [ 0.0; 8.6] |
| Linhart, 2008 | 15 | 300 | 3.7% | 5.0 | [ 2.8; 8.1] |
| Kurova, 1998 | 11 | 107 | 3.3% | 10.3 | [ 5.2; 17.7] |
| Druckler, 2020 | 14 | 1353 | 4.0% | 1.0 | [ 0.6; 1.7] |
| Ruijs, 1988 | 2 | 20 | 1.8% | 10.0 | [ 1.2; 31.7] |
| Almeida, 2020 | 1 | 74 | 3.0% | 1.4 | [ 0.0; 7.3] |
| Bernier, 2020 | 6 | 385 | 3.8% | 1.6 | [ 0.6; 3.4] |
| Folch, 2009 | 2 | 400 | 3.8% | 0.5 | [ 0.1; 1.8] |
| Vall-Mayans, 2007 | 11 | 301 | 3.7% | 3.7 | [ 1.8; 6.4] |
| Vu, 2020 | 0 | 96 | 3.2% | 0.0 | [ 0.0; 3.8] |
| Zarakolu, 2010 | 0 | 146 | 3.4% | 0.0 | [ 0.0; 2.5] |
| Kimmitt, 2010 | 0 | 61 | 2.8% | 0.0 | [ 0.0; 5.9] |
| Kimmitt, 2010 | 6 | 53 | 2.7% | 11.3 | [ 4.3; 23.0] |
| King, 2020 | 4 | 50 | 2.7% | 8.0 | [ 2.2; 19.2] |
| Macauley, 2009 | 1 | 40 | 2.5% | 2.5 | [ 0.1; 13.2] |
| Platt, 2011 | 0 | 93 | 3.2% | 0.0 | [ 0.0; 3.9] |
| Platt, 2011 | 5 | 139 | 3.4% | 3.6 | [ 1.2; 8.2] |
| Ulla, 1993 | 12 | 86 | 3.1% | 14.0 | [ 7.4; 23.1] |
| **Common effect model** | **442** | **10021** | **--** | **2.9** | **[ 2.6; 3.3]** |
| **Random effects model** | | | **100.0%** | **3.2** | **[ 1.8; 4.8]** |
| **Prediction interval** | | | | | **[ 0.0; 14.8]** |

Heterogeneity: $I^2 = 91\%$, $\chi^2_{30} = 318.47$ ($p < 0.01$)

Events per 100 observations; x-axis: Gonorrhea prevalence (95% CI), 0–100

## F. Male sex workers and men who have sex with men

| Author, Year | Gonorrhea positive | Sample size | W(Random) | Prev(%) | 95% CI |
|---|---|---|---|---|---|
| De Baetselier, 2019 | 9 | 464 | 9.2% | 1.9 | [0.9; 3.7] |
| De Baetselier, 2020 | 0 | 117 | 3.5% | 0.0 | [0.0; 3.1] |
| De Baetselier, 2021b | 2 | 179 | 5.0% | 1.1 | [0.1; 4.0] |
| Leuridan, 2005 | 2 | 115 | 3.5% | 1.7 | [0.2; 6.1] |
| Bozicevic, 2009 | 1 | 359 | 8.0% | 0.3 | [0.0; 1.5] |
| Plaas, 2015 | 3 | 233 | 6.0% | 1.3 | [0.3; 3.7] |
| Rüütel, 2015 | 1 | 65 | 2.1% | 1.5 | [0.0; 8.3] |
| Fata, 2017 | 2 | 211 | 5.6% | 0.9 | [0.1; 3.4] |
| Rahib, 2019 | 7 | 1226 | 13.9% | 0.6 | [0.2; 1.2] |
| Dudareva-Vizule, 2014 | 13 | 685 | 11.2% | 1.9 | [1.0; 3.2] |
| Druckler, 2020 | 4 | 99 | 3.1% | 4.0 | [1.1; 10.0] |
| Druckler, 2020 | 0 | 20 | 0.7% | 0.0 | [0.0; 16.8] |
| Ribeiro, 2019 | 28 | 1832 | 15.5% | 1.5 | [1.0; 2.2] |
| Tyulenev, 2021 | 13 | 763 | 11.7% | 1.7 | [0.9; 2.9] |
| Vall-Mayans, 2007 | 0 | 28 | 1.0% | 0.0 | [0.0; 12.3] |
| **Common effect model** | **85** | **6396** | **--** | **0.9** | **[0.6; 1.2]** |
| **Random effects model** | | | **100.0%** | **0.9** | **[0.5; 1.4]** |
| **Prediction interval** | | | | | **[0.1; 2.3]** |

Heterogeneity: $I^2 = 38\%$, $\chi^2_{14} = 22.47$ ($p = 0.07$)

Events per 100 observations; x-axis: Gonorrhea prevalence (95% CI), 0–100

## G. HIV-positive individuals and individuals in HIV-discordant couples

| Author, Year | Gonorrhea positive | Sample size | W(Random) | Prev(%) | 95% CI |
|---|---|---|---|---|---|
| Thorsteinsson, 2016 | 0 | 339 | 6.4% | 0.0 | [0.0; 1.1] |
| Farfour, 2021 | 0 | 490 | 6.9% | 0.0 | [0.0; 0.8] |
| Farfour, 2021 | 1 | 291 | 6.2% | 0.3 | [0.0; 1.9] |
| Spinner, 2018 | 4 | 294 | 6.2% | 1.4 | [0.4; 3.4] |
| Keaveney, 2014 | 0 | 50 | 2.8% | 0.0 | [0.0; 7.1] |
| Keaveney, 2014 | 0 | 71 | 3.4% | 0.0 | [0.0; 5.1] |
| Banani, 2013 | 0 | 85 | 3.8% | 0.0 | [0.0; 4.2] |
| De Francesco, 2018 | 25 | 598 | 7.1% | 4.2 | [2.7; 6.1] |
| Heiligenberg, 2012 | 1 | 395 | 6.6% | 0.3 | [0.0; 1.4] |
| Heiligenberg, 2012 | 1 | 229 | 5.8% | 0.4 | [0.0; 2.4] |
| Pace, 2012 | 14 | 682 | 7.2% | 2.1 | [1.1; 3.4] |
| Sprenger, 2014 | 0 | 112 | 4.4% | 0.0 | [0.0; 3.2] |
| Sprenger, 2014 | 0 | 65 | 3.3% | 0.0 | [0.0; 5.5] |
| Sprenger, 2014 | 0 | 47 | 2.7% | 0.0 | [0.0; 7.5] |
| Zarakolu, 2019 | 1 | 80 | 3.7% | 1.2 | [0.0; 6.8] |
| McCarty, 2011 | 9 | 294 | 6.2% | 3.1 | [1.4; 5.7] |
| Soni, 2011 | 0 | 201 | 5.5% | 0.0 | [0.0; 1.8] |
| Soni, 2011 | 0 | 141 | 4.8% | 0.0 | [0.0; 2.6] |
| Soni, 2011 | 8 | 605 | 7.1% | 1.3 | [0.6; 2.6] |
| **Common effect model** | **64** | **5069** | **--** | **0.7** | **[0.5; 1.0]** |
| **Random effects model** | | | **100.0%** | **0.5** | **[0.1; 1.0]** |
| **Prediction interval** | | | | | **[0.0; 3.6]** |

Heterogeneity: $I^2 = 76\%$, $\chi^2_{18} = 74.32$ ($p < 0.01$)

Events per 100 observations; x-axis: Gonorrhea prevalence (95% CI), 0–100

## H. Sexually transmitted infection clinic attendees

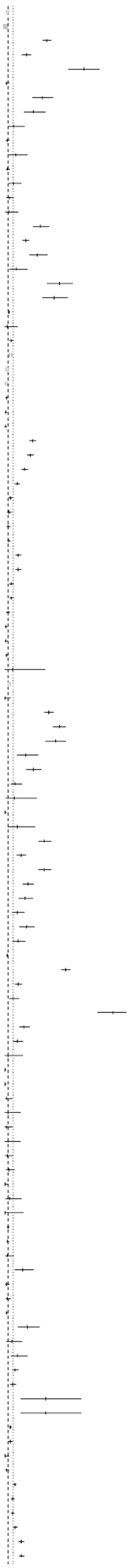


| Author, Year | Gonorrhea positive | Sample size | W(Random) | Prev(%) | 95% CI |
|---|---|---|---|---|---|
| Koch, 1997 | 153 | 10217 | 0.3% | 1.5 | [ 1.3; 1.8] |
| Koch, 1997 | 95 | 31763 | 0.3% | 0.3 | [ 0.2; 0.4] |
| Stary, 1986 | 309 | 1248 | 0.3% | 24.8 | [22.4; 27.3] |
| Stary, 1991 | 90 | 713 | 0.3% | 12.6 | [10.3; 15.3] |
| Stary, 1992a | 56 | 120 | 0.3% | 46.7 | [37.5; 56.0] |
| Stary, 1992a | 4 | 378 | 0.3% | 1.1 | [ 0.3; 2.7] |
| Stary, 1992b | 44 | 200 | 0.3% | 22.0 | [16.5; 28.4] |
| Claeys, 2001 | 26 | 153 | 0.3% | 17.0 | [11.4; 23.9] |
| Ouzounova-Raykova, 2011 | 5 | 96 | 0.3% | 5.2 | [ 1.7; 11.7] |
| Ouzounova-Raykova, 2011 | 7 | 521 | 0.3% | 1.3 | [ 0.5; 2.7] |
| Ouzounova-Raykova, 2011 | 6 | 96 | 0.3% | 6.2 | [ 2.3; 13.1] |
| Ouzounova-Raykova, 2011 | 8 | 521 | 0.3% | 1.5 | [ 0.7; 3.0] |
| Tchoudomirova, 1998 | 8 | 156 | 0.3% | 5.1 | [ 2.2; 9.9] |
| Guralp, 2019 | 7 | 273 | 0.3% | 2.6 | [ 1.0; 5.2] |
| Kacena, 2001 | 2 | 91 | 0.3% | 2.2 | [ 0.3; 7.7] |
| Mikl, 1998 | 64 | 304 | 0.3% | 21.1 | [16.6; 26.1] |
| Mikl, 1998 | 134 | 1090 | 0.3% | 12.3 | [10.4; 14.4] |
| Christophersen, 1988 | 45 | 234 | 0.3% | 19.2 | [14.4; 24.9] |
| Christophersen, 1988 | 7 | 105 | 0.3% | 6.7 | [ 2.7; 13.3] |
| Jensen, 1979 | 49 | 153 | 0.3% | 32.0 | [24.7; 40.0] |
| Jensen, 1979 | 44 | 151 | 0.3% | 29.1 | [22.0; 37.1] |
| Skovgaard, 2012 | 94 | 4180 | 0.3% | 2.2 | [ 1.8; 2.7] |
| Krolov, 2014 | 1 | 70 | 0.2% | 1.4 | [ 0.0; 7.7] |
| Tjagur, 2018 | 31 | 825 | 0.3% | 3.8 | [ 2.6; 5.3] |
| Hiltunen-Back, 1998 | 490 | 14778 | 0.3% | 3.3 | [ 3.0; 3.6] |
| Hiltunen-Back, 1998 | 162 | 12887 | 0.3% | 1.3 | [ 1.1; 1.5] |
| Hiltunen-Back, 1998 | 92 | 10600 | 0.3% | 0.9 | [ 0.7; 1.1] |
| Bebear, 2011 | 7 | 658 | 0.3% | 1.1 | [ 0.4; 2.2] |
| Clarivet, 2014 | 3 | 743 | 0.3% | 0.4 | [ 0.1; 1.2] |
| Clarivet, 2014 | 2 | 638 | 0.3% | 0.3 | [ 0.0; 1.1] |
| Le Faou, 1991 | 218 | 1343 | 0.3% | 16.2 | [14.3; 18.3] |
| Le Faou, 1991 | 208 | 1393 | 0.3% | 14.9 | [13.1; 16.9] |
| Le Faou, 1991 | 161 | 1387 | 0.3% | 11.6 | [10.0; 13.4] |
| Le Faou, 1991 | 95 | 1315 | 0.3% | 7.2 | [ 5.9; 8.8] |
| Le Faou, 1991 | 37 | 1122 | 0.3% | 3.3 | [ 2.3; 4.5] |
| Le Faou, 1991 | 29 | 1171 | 0.3% | 2.5 | [ 1.7; 3.5] |
| Le Faou, 1991 | 17 | 857 | 0.3% | 2.0 | [ 1.2; 3.2] |
| Le Faou, 1991 | 14 | 642 | 0.3% | 2.2 | [ 1.2; 3.6] |
| Le Faou, 1991 | 97 | 1230 | 0.3% | 7.9 | [ 6.4; 9.5] |
| Le Faou, 1991 | 97 | 1238 | 0.3% | 7.8 | [ 6.4; 9.5] |
| Le Faou, 1991 | 39 | 1010 | 0.3% | 3.9 | [ 2.8; 5.2] |
| Le Faou, 1991 | 43 | 1112 | 0.3% | 3.9 | [ 2.8; 5.2] |
| Le Faou, 1991 | 18 | 1057 | 0.3% | 1.7 | [ 1.0; 2.7] |
| Le Faou, 1991 | 4 | 749 | 0.3% | 0.5 | [ 0.1; 1.4] |
| Le Faou, 1991 | 3 | 582 | 0.3% | 0.5 | [ 0.1; 1.5] |
| Le Faou, 1991 | 5 | 520 | 0.3% | 1.0 | [ 0.3; 2.2] |
| Le Roy, 2012 | 1 | 21 | 0.2% | 4.8 | [ 0.1; 23.8] |
| Le Roy, 2013 | 277 | 11010 | 0.3% | 2.5 | [ 2.2; 2.8] |
| Toskine, 2004 | 0 | 101 | 0.3% | 0.0 | [ 0.0; 3.6] |
| Bozdech, 1982 | 284 | 1096 | 0.3% | 25.9 | [23.3; 28.6] |
| Bozdech, 1982 | 206 | 643 | 0.3% | 32.0 | [28.4; 35.8] |
| Bozdech, 1982 | 70 | 235 | 0.3% | 29.8 | [24.0; 36.1] |
| Bozdech, 1982 | 15 | 122 | 0.3% | 12.3 | [ 7.0; 19.5] |
| Breustedt, 1989 | 54 | 321 | 0.3% | 16.8 | [12.9; 21.4] |
| Breustedt, 1989 | 14 | 230 | 0.3% | 6.1 | [ 3.4; 10.0] |
| Elsner, 1985 | 2 | 36 | 0.2% | 5.6 | [ 0.7; 18.7] |
| Elsner, 1987 | 0 | 420 | 0.3% | 0.0 | [ 0.0; 0.9] |
| Graspeuntner, 2018 | 4 | 54 | 0.2% | 7.4 | [ 2.1; 17.9] |
| Hofmann, 1985 | 123 | 526 | 0.3% | 23.4 | [19.8; 27.2] |
| Hofmann, 1985 | 44 | 464 | 0.3% | 9.5 | [ 7.0; 12.5] |
| Hofmann, 1985 | 123 | 526 | 0.3% | 23.4 | [19.8; 27.2] |
| Hofmann, 1985 | 63 | 464 | 0.3% | 13.6 | [10.6; 17.0] |
| Hofmann, 1985 | 31 | 261 | 0.3% | 11.9 | [ 8.2; 16.4] |
| Hofmann, 1985 | 16 | 220 | 0.3% | 7.3 | [ 4.2; 11.5] |
| Hofmann, 1985 | 33 | 261 | 0.3% | 12.6 | [ 8.9; 17.3] |
| Hofmann, 1985 | 17 | 220 | 0.3% | 7.7 | [ 4.6; 12.1] |
| Jansen, 2020 | 32 | 2303 | 0.3% | 1.4 | [ 1.0; 2.0] |
| Cordtz, 1988 | 483 | 1346 | 0.3% | 35.9 | [33.3; 38.5] |
| Cordtz, 1988 | 62 | 788 | 0.3% | 7.9 | [ 6.1; 10.0] |
| Cordtz, 1988 | 11 | 229 | 0.3% | 4.8 | [ 2.4; 8.4] |
| Mardh, 1980 | 84 | 132 | 0.3% | 63.6 | [54.8; 71.8] |
| Moller, 1992 | 52 | 465 | 0.3% | 11.2 | [ 8.5; 14.4] |
| Moller, 1992 | 25 | 338 | 0.3% | 7.4 | [ 4.8; 10.7] |
| Mihalik, 2013 | 1 | 51 | 0.2% | 2.0 | [ 0.0; 10.4] |
| Hilmarsdottir, 2021 | 0 | 491 | 0.3% | 0.0 | [ 0.0; 0.7] |
| Hilmarsdottir, 2021 | 0 | 487 | 0.3% | 0.0 | [ 0.0; 0.8] |
| Walsh, 2011b | 2 | 164 | 0.3% | 1.2 | [ 0.1; 4.3] |
| Walsh, 2011b | 1 | 58 | 0.2% | 1.7 | [ 0.0; 9.2] |
| Walsh, 2011b | 2 | 164 | 0.3% | 1.2 | [ 0.1; 4.3] |
| Walsh, 2011b | 1 | 58 | 0.2% | 1.7 | [ 0.0; 9.2] |
| Gottesman, 2017 | 2 | 141 | 0.3% | 1.4 | [ 0.2; 5.0] |
| Joffe, 2006 | 5 | 200 | 0.3% | 2.5 | [ 0.8; 5.7] |
| Joffe, 2006 | 0 | 176 | 0.3% | 0.0 | [ 0.0; 2.1] |
| Madjar, 1996 | 2 | 71 | 0.2% | 2.8 | [ 0.3; 9.8] |
| Madjar, 1996 | 0 | 32 | 0.2% | 0.0 | [ 0.0; 10.9] |
| Mor, 2012 | 78 | 4213 | 0.3% | 1.9 | [ 1.5; 2.3] |
| Rich, 2020 | 37 | 2259 | 0.3% | 1.6 | [ 1.2; 2.3] |
| Rich, 2020 | 2 | 130 | 0.3% | 1.5 | [ 0.2; 5.4] |
| Castriciano, 2011 | 14 | 134 | 0.3% | 10.4 | [ 5.8; 16.9] |
| Chiodo, 1991 | 4 | 385 | 0.3% | 1.0 | [ 0.3; 2.6] |
| Chiodo, 1991 | 6 | 385 | 0.3% | 1.6 | [ 0.6; 3.4] |
| D'Antuono, 2001 | 5 | 558 | 0.3% | 0.9 | [ 0.3; 2.1] |
| Ferrero, 2011 | 16 | 122 | 0.3% | 13.1 | [ 7.7; 20.4] |
| Foschi, 2017 | 4 | 100 | 0.3% | 4.0 | [ 1.1; 9.9] |
| Foschi, 2020 | 10 | 134 | 0.3% | 7.5 | [ 3.6; 13.3] |
| Marangoni, 2011 | 41 | 700 | 0.3% | 5.9 | [ 4.2; 7.9] |
| Marangoni, 2011 | 33 | 700 | 0.3% | 4.7 | [ 3.3; 6.6] |
| Pagani, 1996 | 6 | 25 | 0.2% | 24.0 | [ 9.4; 45.1] |
| Pagani, 1996 | 6 | 25 | 0.2% | 24.0 | [ 9.4; 45.1] |
| Achterbergh, 2020 | 103 | 3196 | 0.3% | 3.2 | [ 2.6; 3.9] |
| Achterbergh, 2020 | 39 | 1255 | 0.3% | 3.1 | [ 2.2; 4.2] |
| De Vrieze, 2014 | 0 | 139 | 0.3% | 0.0 | [ 0.0; 2.6] |
| De Vrieze, 2014 | 3 | 334 | 0.3% | 0.9 | [ 0.2; 2.6] |
| De Wit, 1993 | 174 | 3000 | 0.3% | 5.8 | [ 5.0; 6.7] |
| De Wit, 1993 | 95 | 2065 | 0.3% | 4.6 | [ 3.7; 5.6] |
| De Wit, 1993 | 73 | 1622 | 0.3% | 4.5 | [ 3.5; 5.6] |
| De Wit, 1993 | 101 | 1656 | 0.3% | 6.1 | [ 5.0; 7.4] |
| De Wit, 1993 | 157 | 1619 | 0.3% | 9.7 | [ 8.3; 11.2] |
| De Wit, 1993 | 163 | 1663 | 0.3% | 9.8 | [ 8.4; 11.3] |

| | | | | | |
|---|---|---|---|---|---|
| Den Heijer, 2017 | 68 | 10268 | 0.3% | 0.7 | [ 0.5; 0.8] |
| Den Heijer, 2017 | 123 | 5177 | 0.3% | 2.4 | [ 2.0; 2.8] |
| Druckler, 2018 | 145 | 4925 | 0.3% | 2.9 | [ 2.5; 3.5] |
| Druckler, 2021 | 0 | 218 | 0.3% | 0.0 | [ 0.0; 1.7] |
| Druckler, 2021 | 0 | 49 | 0.2% | 0.0 | [ 0.0; 7.3] |
| Dukers-Muijrers, 2010 | 718 | 6706 | 0.3% | 10.7 | [10.0; 11.5] |
| Dukers-Muijrers, 2010 | 177 | 861 | 0.3% | 20.6 | [17.9; 23.4] |
| Dukers-Muijrers, 2010 | 20 | 358 | 0.3% | 5.6 | [ 3.4; 8.5] |
| Dukers-Muijrers, 2010 | 153 | 1040 | 0.3% | 14.7 | [12.6; 17.0] |
| Fennema, 1997 | 162 | 680 | 0.3% | 23.8 | [20.7; 27.2] |
| Gotz, 2011 | 8 | 26 | 0.2% | 30.8 | [14.3; 51.8] |
| Heijman, 2007 | 630 | 14391 | 0.3% | 4.4 | [ 4.0; 4.7] |
| Heijman, 2007 | 52 | 8056 | 0.3% | 0.6 | [ 0.5; 0.8] |
| Heijman, 2009 | 69 | 340 | 0.3% | 20.3 | [16.1; 25.0] |
| Heijman, 2009 | 23 | 315 | 0.3% | 7.3 | [ 4.7; 10.8] |
| Heiligenberg, 2011b | 11 | 961 | 0.3% | 1.1 | [ 0.6; 2.0] |
| Heiligenberg, 2011b | 32 | 667 | 0.3% | 4.8 | [ 3.3; 6.7] |
| Heiligenberg, 2011b | 9 | 1125 | 0.3% | 0.8 | [ 0.4; 1.5] |
| Hoebe, 2006 | 5 | 413 | 0.3% | 1.2 | [ 0.4; 2.8] |
| Hoornenborg, 2019 | 3 | 354 | 0.3% | 0.8 | [ 0.2; 2.5] |
| Koedijk, 2012 | 2065 | 206525 | 0.3% | 1.0 | [ 1.0; 1.0] |
| Koedijk, 2012 | 2349 | 69077 | 0.3% | 3.4 | [ 3.3; 3.5] |
| Lelijveld, 1981 | 13 | 28 | 0.2% | 46.4 | [27.5; 66.1] |
| Lelijveld, 1981 | 36 | 101 | 0.3% | 35.6 | [26.4; 45.8] |
| Lelijveld, 1981 | 19 | 61 | 0.2% | 31.1 | [19.9; 44.3] |
| Lelijveld, 1981 | 7 | 29 | 0.2% | 24.1 | [10.3; 43.5] |
| Lelijveld, 1981 | 10 | 29 | 0.2% | 34.5 | [17.9; 54.3] |
| Matser, 2018 | 126 | 2983 | 0.3% | 4.2 | [ 3.5; 5.0] |
| Peters, 2011 | 41 | 1451 | 0.3% | 2.8 | [ 2.0; 3.8] |
| Peters, 2011 | 48 | 4299 | 0.3% | 1.1 | [ 0.8; 1.5] |
| Prins, 1994 | 12 | 155 | 0.3% | 7.7 | [ 4.1; 13.1] |
| Prins, 1994 | 5 | 234 | 0.3% | 2.1 | [ 0.7; 4.9] |
| Slurink, 2020 | 53 | 2961 | 0.3% | 1.8 | [ 1.3; 2.3] |
| Slurink, 2020 | 103 | 4178 | 0.3% | 2.5 | [ 2.0; 3.0] |
| Slurink, 2020 | 66 | 2484 | 0.3% | 2.7 | [ 2.1; 3.4] |
| Slurink, 2020 | 39 | 825 | 0.3% | 4.7 | [ 3.4; 6.4] |
| Slurink, 2020 | 49 | 1795 | 0.3% | 2.7 | [ 2.0; 3.6] |
| Slurink, 2020 | 57 | 1237 | 0.3% | 4.6 | [ 3.5; 5.9] |
| Spauwen, 2015 | 3 | 289 | 0.3% | 1.0 | [ 0.2; 3.0] |
| Thewessen, 1990 | 12 | 482 | 0.3% | 2.5 | [ 1.3; 4.3] |
| Tielemans, 2021 | 94 | 3216 | 0.3% | 2.9 | [ 2.4; 3.6] |
| Tielemans, 2021 | 126 | 4175 | 0.3% | 3.0 | [ 2.5; 3.6] |
| Tielemans, 2021 | 105 | 2334 | 0.3% | 4.5 | [ 3.7; 5.4] |
| Tielemans, 2021 | 92 | 3089 | 0.3% | 3.0 | [ 2.4; 3.6] |
| Tielemans, 2021 | 52 | 1343 | 0.3% | 3.9 | [ 2.9; 5.0] |
| Tielemans, 2021 | 80 | 1828 | 0.3% | 4.4 | [ 3.5; 5.4] |
| Vahidnia, 2014 | 0 | 45 | 0.2% | 0.0 | [ 0.0; 7.9] |
| Vahidnia, 2014 | 1 | 255 | 0.3% | 0.4 | [ 0.0; 2.2] |
| Vahidnia, 2014 | 1 | 147 | 0.3% | 0.7 | [ 0.0; 3.7] |
| Vahidnia, 2014 | 0 | 83 | 0.2% | 0.0 | [ 0.0; 4.3] |
| Vahidnia, 2014 | 0 | 17 | 0.2% | 0.0 | [ 0.0; 19.5] |
| Vahidnia, 2014 | 3 | 100 | 0.3% | 3.0 | [ 0.6; 8.5] |
| Vahidnia, 2014 | 0 | 105 | 0.3% | 0.0 | [ 0.0; 3.5] |
| Vahidnia, 2014 | 2 | 84 | 0.2% | 2.4 | [ 0.3; 8.3] |
| Van den Hoek, 1989 | 53 | 281 | 0.3% | 18.9 | [14.5; 23.9] |
| Van der Snoek, 2004 | 3 | 286 | 0.3% | 1.0 | [ 0.2; 3.0] |
| Van der Snoek, 2004 | 17 | 318 | 0.3% | 5.3 | [ 3.1; 8.4] |
| Van Doornum, 2001 | 6 | 503 | 0.3% | 1.2 | [ 0.4; 2.6] |
| Van Doornum, 2001 | 21 | 498 | 0.3% | 4.2 | [ 2.6; 6.4] |
| Van Doornum, 2001 | 3 | 503 | 0.3% | 0.6 | [ 0.1; 1.7] |
| Van Doornum, 2001 | 18 | 498 | 0.3% | 3.6 | [ 2.2; 5.7] |
| Van Liere, 2014a | 37 | 2436 | 0.3% | 1.5 | [ 1.1; 2.1] |
| Van Liere, 2014a | 17 | 1321 | 0.3% | 1.3 | [ 0.8; 2.1] |
| Van Liere, 2014b | 0 | 654 | 0.3% | 0.0 | [ 0.0; 0.6] |
| Van Liere, 2015 | 88 | 10972 | 0.3% | 0.8 | [ 0.6; 1.0] |
| Van Liere, 2015 | 176 | 9534 | 0.3% | 1.8 | [ 1.6; 2.1] |
| Van Liere, 2020 | 8438 | 221087 | 0.3% | 3.8 | [ 3.7; 3.9] |
| Van Liere, 2020 | 955 | 26290 | 0.3% | 3.6 | [ 3.4; 3.9] |
| Van Rooijen, 2015 | 357 | 12926 | 0.3% | 2.8 | [ 2.5; 3.1] |
| Van Rooijen, 2015 | 103 | 6690 | 0.3% | 1.5 | [ 1.3; 1.9] |
| Van Rooijen, 2018 | 1680 | 157763 | 0.3% | 1.1 | [ 1.0; 1.1] |
| Van Rooijen, 2018 | 5369 | 193137 | 0.3% | 2.8 | [ 2.7; 2.9] |
| Van Voorst Vader, 1991 | 41 | 217 | 0.3% | 18.9 | [13.9; 24.7] |
| Verhaegh-Haasnoot, 2015 | 23 | 801 | 0.3% | 2.9 | [ 1.8; 4.3] |
| Verscheijden, 2015 | 57 | 3762 | 0.3% | 1.5 | [ 1.1; 2.0] |
| Verscheijden, 2015 | 55 | 4173 | 0.3% | 1.3 | [ 1.0; 1.7] |
| Verscheijden, 2015 | 84 | 4933 | 0.3% | 1.7 | [ 1.4; 2.1] |
| Verscheijden, 2015 | 94 | 5286 | 0.3% | 1.8 | [ 1.4; 2.2] |
| Verscheijden, 2015 | 104 | 5805 | 0.3% | 1.8 | [ 1.5; 2.2] |
| Verscheijden, 2015 | 103 | 5749 | 0.3% | 1.8 | [ 1.5; 2.2] |
| Vlaspolder, 1993 | 25 | 491 | 0.3% | 5.1 | [ 3.3; 7.4] |
| Vlaspolder, 1993 | 89 | 623 | 0.3% | 14.3 | [11.6; 17.3] |
| Vlaspolder, 1993 | 22 | 491 | 0.3% | 4.5 | [ 2.8; 6.7] |
| Vlaspolder, 1993 | 84 | 623 | 0.3% | 13.5 | [10.9; 16.4] |
| Vriend, 2013 | 32 | 2226 | 0.3% | 1.4 | [ 1.0; 2.0] |
| Vriend, 2013 | 17 | 914 | 0.3% | 1.9 | [ 1.1; 3.0] |
| Vriend, 2013 | 7 | 171 | 0.3% | 4.1 | [ 1.7; 8.3] |
| Wind, 2015 | 37 | 1085 | 0.3% | 3.4 | [ 2.4; 4.7] |
| Wind, 2015 | 15 | 805 | 0.3% | 1.9 | [ 1.0; 3.1] |
| Wind, 2015 | 33 | 1085 | 0.3% | 3.0 | [ 2.1; 4.2] |
| Wind, 2015 | 13 | 805 | 0.3% | 1.6 | [ 0.9; 2.7] |
| Falk, 1993 | 1142 | 21824 | 0.3% | 5.2 | [ 4.9; 5.5] |
| Falk, 1993 | 1798 | 28893 | 0.3% | 6.2 | [ 5.9; 6.5] |
| Falk, 1993 | 1524 | 30942 | 0.3% | 4.9 | [ 4.7; 5.2] |
| Falk, 1993 | 1644 | 32097 | 0.3% | 5.1 | [ 4.9; 5.4] |
| Falk, 1993 | 1510 | 32857 | 0.3% | 4.6 | [ 4.4; 4.8] |
| Falk, 1993 | 1284 | 31706 | 0.3% | 4.0 | [ 3.8; 4.3] |
| Falk, 1993 | 1444 | 28561 | 0.3% | 5.1 | [ 4.8; 5.3] |
| Falk, 1993 | 1135 | 26095 | 0.3% | 4.3 | [ 4.1; 4.6] |
| Hjelmevoll, 2012 | 50 | 257 | 0.3% | 19.5 | [14.8; 24.8] |
| Moi, 2009 | 136 | 8468 | 0.3% | 1.6 | [ 1.3; 1.9] |
| Reinton, 2013 | 30 | 2000 | 0.3% | 1.5 | [ 1.0; 2.1] |
| Staerfelt, 1983 | 77 | 531 | 0.3% | 14.5 | [11.6; 17.8] |
| Staerfelt, 1983 | 40 | 216 | 0.3% | 18.5 | [13.6; 24.4] |
| Unemo, 2018 | 8 | 1826 | 0.3% | 0.4 | [ 0.2; 0.9] |
| Unemo, 2018 | 1 | 721 | 0.3% | 0.1 | [ 0.0; 0.8] |
| Grad, 2020 | 17 | 207 | 0.3% | 8.2 | [ 4.9; 12.8] |
| Grad, 2020 | 1 | 42 | 0.2% | 2.4 | [ 0.1; 12.6] |
| Vica, 2021 | 44 | 622 | 0.3% | 7.1 | [ 5.2; 9.4] |
| Renton, 2009 | 24 | 105 | 0.3% | 22.9 | [15.2; 32.1] |
| Renton, 2009 | 16 | 105 | 0.3% | 15.2 | [ 9.0; 23.6] |
| Renton, 2009 | 13 | 105 | 0.3% | 12.4 | [ 6.8; 20.2] |
| Renton, 2009 | 24 | 99 | 0.3% | 24.2 | [16.2; 33.9] |
| Renton, 2009 | 15 | 99 | 0.3% | 15.2 | [ 8.7; 23.8] |
| Renton, 2009 | 6 | 99 | 0.3% | 6.1 | [ 2.3; 12.7] |
| Taylor-Robinson, 2009 | 41 | 172 | 0.3% | 23.8 | [17.7; 30.9] |

| | | | | | |
|---|---|---|---|---|---|
| Unemo, 2006 | 91 | 19880 | 0.3% | 0.5 | [ 0.4; 0.6] |
| Unemo, 2006 | 205 | 200106 | 0.3% | 0.1 | [ 0.1; 0.1] |
| Unemo, 2006 | 73 | 5519 | 0.3% | 1.3 | [ 1.0; 1.7] |
| Unemo, 2006 | 260 | 30281 | 0.3% | 0.9 | [ 0.8; 1.0] |
| Jeverica, 2013 | 0 | 67 | 0.2% | 0.0 | [ 0.0; 5.4] |
| Aguirrebengoa, 2021 | 0 | 110 | 0.3% | 0.0 | [ 0.0; 3.3] |
| Ayerdi Aguirreb, 2020 | 11 | 149 | 0.3% | 7.4 | [ 3.7; 12.8] |
| Ayerdi Aguirreb, 2020 | 19 | 85 | 0.2% | 22.4 | [14.0; 32.7] |
| Ayerdi Aguirreb, 2020 | 14 | 140 | 0.3% | 10.0 | [ 5.6; 16.2] |
| Corbeto, 2011 | 1 | 423 | 0.3% | 0.2 | [ 0.0; 1.3] |
| De Salazar, 2019 | 2 | 107 | 0.3% | 1.9 | [ 0.2; 6.6] |
| De Salazar, 2019 | 11 | 214 | 0.3% | 5.1 | [ 2.6; 9.0] |
| Gonzalez, 1983 | 145 | 1380 | 0.3% | 10.5 | [ 8.9; 12.2] |
| Gonzalez, 1983 | 46 | 1380 | 0.3% | 3.3 | [ 2.5; 4.4] |
| Lopez-Corbeto, 2020 | 0 | 50 | 0.2% | 0.0 | [ 0.0; 7.1] |
| Lopez-Corbeto, 2020 | 6 | 982 | 0.3% | 0.6 | [ 0.2; 1.3] |
| Perea, 1981 | 61 | 534 | 0.3% | 11.4 | [ 8.9; 14.4] |
| Perea, 1981 | 31 | 345 | 0.3% | 9.0 | [ 6.2; 12.5] |
| Perez Bernal, 1987 | 72 | 1011 | 0.3% | 7.1 | [ 5.6; 8.9] |
| Vall-Mayans, 2007 | 9 | 201 | 0.3% | 4.5 | [ 2.1; 8.3] |
| Vall-Mayans, 2007 | 6 | 191 | 0.3% | 3.1 | [ 1.2; 6.7] |
| Vall-Mayans, 2007 | 9 | 132 | 0.3% | 6.8 | [ 3.2; 12.5] |
| Vazquez, 1991 | 89 | 757 | 0.3% | 11.8 | [ 9.5; 14.3] |
| Andersson, 2021b | 1 | 101 | 0.3% | 1.0 | [ 0.0; 5.4] |
| Andersson, 2021b | 1 | 193 | 0.3% | 0.5 | [ 0.0; 2.9] |
| Andersson, 2021b | 4 | 109 | 0.3% | 3.7 | [ 1.0; 9.1] |
| Andersson, 2021b | 0 | 356 | 0.3% | 0.0 | [ 0.0; 1.0] |
| Bjornelius, 2000 | 4 | 105 | 0.3% | 3.8 | [ 1.0; 9.5] |
| Falk, 2004 | 2 | 88 | 0.3% | 2.3 | [ 0.3; 8.0] |
| Hellgren, 1982 | 1686 | 8865 | 0.3% | 19.0 | [18.2; 19.9] |
| Hellgren, 1982 | 2629 | 8865 | 0.3% | 29.7 | [28.7; 30.6] |
| Lycke, 1980 | 99 | 211 | 0.3% | 46.9 | [40.0; 53.9] |
| Rumyantseva, 2015 | 2 | 554 | 0.3% | 0.4 | [ 0.0; 1.3] |
| Rumyantseva, 2015 | 0 | 209 | 0.3% | 0.0 | [ 0.0; 1.7] |
| Unemo, 2018 | 5 | 914 | 0.3% | 0.5 | [ 0.2; 1.3] |
| Unemo, 2018 | 5 | 535 | 0.3% | 0.9 | [ 0.3; 2.2] |
| Bovet, 1982 | 162 | 498 | 0.3% | 32.5 | [28.4; 36.8] |
| Dona, 2018 | 46 | 177 | 0.3% | 26.0 | [19.7; 33.1] |
| Dona, 2018 | 4 | 11 | 0.1% | 36.4 | [10.9; 69.2] |
| Sakem, 2011 | 11 | 1346 | 0.3% | 0.8 | [ 0.4; 1.5] |
| Sakem, 2011 | 26 | 436 | 0.3% | 6.0 | [ 3.9; 8.6] |
| Boiko, 2019 | 1 | 296 | 0.3% | 0.3 | [ 0.0; 1.9] |
| Boiko, 2019 | 1 | 296 | 0.3% | 0.3 | [ 0.0; 1.9] |
| Boiko, 2019 | 1 | 27 | 0.2% | 3.7 | [ 0.1; 19.0] |
| Boiko, 2019 | 6 | 159 | 0.3% | 3.8 | [ 1.4; 8.0] |
| Boiko, 2019 | 4 | 159 | 0.3% | 2.5 | [ 0.7; 6.3] |
| Boiko, 2019 | 3 | 16 | 0.2% | 18.8 | [ 4.0; 45.6] |
| Annan, 2009 | 142 | 3076 | 0.3% | 4.6 | [ 3.9; 5.4] |
| Arumainayagam, 1991 | 0 | 50 | 0.2% | 0.0 | [ 0.0; 7.1] |
| Arya, 1981 | 149 | 474 | 0.3% | 31.4 | [27.3; 35.8] |
| Bailey, 2004 | 2 | 708 | 0.3% | 0.3 | [ 0.0; 1.0] |
| Benn, 2007 | 16 | 165 | 0.3% | 9.7 | [ 5.6; 15.3] |
| Benn, 2007 | 22 | 303 | 0.3% | 7.3 | [ 4.6; 10.8] |
| Benn, 2007 | 5 | 131 | 0.3% | 3.8 | [ 1.3; 8.7] |
| Bhaduri, 2007 | 3 | 181 | 0.3% | 1.7 | [ 0.3; 4.8] |
| Bhaduri, 2007 | 3 | 181 | 0.3% | 1.7 | [ 0.3; 4.8] |
| Bhaduri, 2007 | 4 | 174 | 0.3% | 2.3 | [ 0.6; 5.8] |
| Bhaduri, 2007 | 0 | 174 | 0.3% | 0.0 | [ 0.0; 2.1] |
| Broad, 2021 | 4 | 307 | 0.3% | 1.3 | [ 0.4; 3.3] |
| Broad, 2021 | 10 | 173 | 0.3% | 5.8 | [ 2.8; 10.4] |
| Broad, 2021 | 11 | 79 | 0.2% | 13.9 | [ 7.2; 23.5] |
| Carne, 2013 | 3 | 140 | 0.3% | 2.1 | [ 0.4; 6.1] |
| Carne, 2013 | 3 | 458 | 0.3% | 0.7 | [ 0.1; 1.9] |
| Carne, 2013 | 1 | 200 | 0.3% | 0.5 | [ 0.0; 2.8] |
| Carne, 2013 | 0 | 151 | 0.3% | 0.0 | [ 0.0; 2.4] |
| Carne, 2013 | 2 | 231 | 0.3% | 0.9 | [ 0.1; 3.1] |
| Carne, 2013 | 2 | 431 | 0.3% | 0.5 | [ 0.1; 1.7] |
| Carne, 2013 | 0 | 126 | 0.3% | 0.0 | [ 0.0; 2.9] |
| Carne, 2013 | 0 | 75 | 0.2% | 0.0 | [ 0.0; 4.8] |
| Chalker, 2009 | 6 | 286 | 0.3% | 2.1 | [ 0.8; 4.5] |
| Chalker, 2009 | 0 | 25 | 0.2% | 0.0 | [ 0.0; 13.7] |
| Champion, 1999 | 1 | 19 | 0.2% | 5.3 | [ 0.1; 26.0] |
| Dave, 2012 | 1 | 197 | 0.3% | 0.5 | [ 0.0; 2.8] |
| Downing, 2010 | 18 | 3847 | 0.3% | 0.5 | [ 0.3; 0.7] |
| Downing, 2010 | 78 | 12181 | 0.3% | 0.6 | [ 0.5; 0.8] |
| Evans, 1998 | 7 | 748 | 0.3% | 0.9 | [ 0.4; 1.9] |
| Evans, 1998 | 24 | 156 | 0.3% | 15.4 | [10.1; 22.0] |
| Fraser, 2014 | 3 | 34 | 0.2% | 8.8 | [ 1.9; 23.7] |
| Garner, 2015 | 16 | 339 | 0.3% | 4.7 | [ 2.7; 7.6] |
| Garner, 2015 | 13 | 135 | 0.3% | 9.6 | [ 5.2; 15.9] |
| Garner, 2015 | 5 | 540 | 0.3% | 0.9 | [ 0.3; 2.1] |
| Garner, 2015 | 6 | 188 | 0.3% | 3.2 | [ 1.2; 6.8] |
| Garner, 2015 | 5 | 616 | 0.3% | 0.8 | [ 0.3; 1.9] |
| Garner, 2015 | 5 | 304 | 0.3% | 1.6 | [ 0.5; 3.8] |
| Gellan, 1986 | 940 | 18903 | 0.3% | 5.0 | [ 4.7; 5.3] |
| Gellan, 1986 | 768 | 17800 | 0.3% | 4.3 | [ 4.0; 4.6] |
| Gellan, 1986 | 723 | 18760 | 0.3% | 3.9 | [ 3.6; 4.1] |
| Gellan, 1986 | 483 | 17595 | 0.3% | 2.7 | [ 2.5; 3.0] |
| Harding-Esch, 2019 | 3 | 395 | 0.3% | 0.8 | [ 0.2; 2.2] |
| Harding-Esch, 2019 | 12 | 392 | 0.3% | 3.1 | [ 1.6; 5.3] |
| Higgins, 2016 | 37 | 390 | 0.3% | 9.5 | [ 6.8; 12.8] |
| Higgins, 2016 | 34 | 390 | 0.3% | 8.7 | [ 6.1; 12.0] |
| Higgins, 2016 | 35 | 390 | 0.3% | 9.0 | [ 6.3; 12.3] |
| Hilton, 1974 | 57 | 279 | 0.3% | 20.4 | [15.9; 25.6] |
| Hopkins, 2012 | 76 | 15253 | 0.3% | 0.5 | [ 0.4; 0.6] |
| Hunter, 1981 | 62 | 480 | 0.3% | 12.9 | [10.0; 16.3] |
| Hussey, 2003 | 2 | 12 | 0.1% | 16.7 | [ 2.1; 48.4] |
| Killick, 2012 | 15 | 4160 | 0.3% | 0.4 | [ 0.2; 0.6] |
| Killick, 2012 | 18 | 4160 | 0.3% | 0.4 | [ 0.3; 0.7] |
| King, 2020 | 3 | 50 | 0.2% | 6.0 | [ 1.3; 16.5] |
| Kinghorn, 1981 | 224 | 1080 | 0.3% | 20.7 | [18.4; 23.3] |
| Leung, 2006 | 2 | 378 | 0.3% | 0.5 | [ 0.1; 1.9] |
| Lewis, 2004 | 2 | 92 | 0.3% | 2.2 | [ 0.3; 7.6] |
| Mahto, 2011 | 1 | 358 | 0.3% | 0.3 | [ 0.0; 1.5] |
| Maini, 1992 | 262 | 5571 | 0.3% | 4.7 | [ 4.2; 5.3] |
| Maini, 1992 | 228 | 8992 | 0.3% | 2.5 | [ 2.2; 2.9] |
| Maini, 1992 | 806 | 15976 | 0.3% | 5.0 | [ 4.7; 5.4] |
| Manavi, 2004 | 26 | 411 | 0.3% | 6.3 | [ 4.2; 9.1] |
| Manavi, 2005 | 7 | 60 | 0.2% | 11.7 | [ 4.8; 22.6] |
| Morris, 2014 | 95 | 3804 | 0.3% | 2.5 | [ 2.0; 3.0] |
| Mulcahy, 1987 | 19 | 109 | 0.3% | 17.4 | [10.8; 25.9] |
| Mulcahy, 1987 | 16 | 115 | 0.3% | 13.9 | [ 8.2; 21.6] |
| Mulcahy, 1987 | 7 | 83 | 0.2% | 8.4 | [ 3.5; 16.6] |
| Nicholls, 2018 | 5 | 1584 | 0.3% | 0.3 | [ 0.1; 0.7] |
| Pittrof, 2007 | 1 | 295 | 0.3% | 0.3 | [ 0.0; 1.9] |
| Pond, 2017 | 0 | 124 | 0.3% | 0.0 | [ 0.0; 2.9] |

| | | | | |
|---|---|---|---|---|
| Rao, 2008 | 49 | 817 | 0.3% | 6.0 [ 4.5; 7.9] |
| Rao, 2008 | 40 | 695 | 0.3% | 5.8 [ 4.1; 7.8] |
| Rao, 2008 | 221 | 4438 | 0.3% | 5.0 [ 4.4; 5.7] |
| Rao, 2008 | 70 | 3499 | 0.3% | 2.0 [ 1.6; 2.5] |
| Robinson, 1974 | 38 | 450 | 0.3% | 8.4 [ 6.0; 11.4] |
| Ross, 1991a | 26 | 606 | 0.3% | 4.3 [ 2.8; 6.2] |
| Ross, 1991a | 31 | 504 | 0.3% | 6.2 [ 4.2; 8.6] |
| Ross, 1991a | 16 | 369 | 0.3% | 4.3 [ 2.5; 6.9] |
| Ross, 1991a | 24 | 374 | 0.3% | 6.4 [ 4.2; 9.4] |
| Ross, 1991a | 11 | 347 | 0.3% | 3.2 [ 1.6; 5.6] |
| Ross, 1991a | 26 | 378 | 0.3% | 6.9 [ 4.5; 9.9] |
| Sethupathi, 2010 | 8 | 24 | 0.2% | 33.3 [15.6; 55.3] |
| Shahidullah, 1976 | 13 | 106 | 0.3% | 12.3 [ 6.7; 20.1] |
| Shahidullah, 1976 | 10 | 106 | 0.3% | 9.4 [ 4.6; 16.7] |
| Skinner, 1996 | 1 | 199 | 0.3% | 0.5 [ 0.0; 2.8] |
| Skinner, 1996 | 4 | 204 | 0.3% | 2.0 [ 0.5; 4.9] |
| Soni, 2008 | 14 | 177 | 0.3% | 7.9 [ 4.4; 12.9] |
| Soni, 2008 | 11 | 180 | 0.3% | 6.1 [ 3.1; 10.7] |
| Stewart, 2012 | 78 | 3859 | 0.3% | 2.0 [ 1.6; 2.5] |
| Stewart, 2012 | 92 | 3859 | 0.3% | 2.4 [ 1.9; 2.9] |
| Sugunendran, 2001 | 11 | 80 | 0.2% | 13.8 [ 7.1; 23.3] |
| Sultan, 2016 | 140 | 1064 | 0.3% | 13.2 [11.2; 15.3] |
| Thin, 1989 | 10 | 50 | 0.2% | 20.0 [10.0; 33.7] |
| Thin, 1989 | 14 | 71 | 0.2% | 19.7 [11.2; 30.9] |
| Thin, 1989 | 7 | 44 | 0.2% | 15.9 [ 6.6; 30.1] |
| Thin, 1989 | 7 | 51 | 0.2% | 13.7 [ 5.7; 26.3] |
| Tipple, 2018 | 3 | 901 | 0.3% | 0.3 [ 0.1; 1.0] |
| Tomlinson, 1991 | 2 | 11 | 0.1% | 18.2 [ 2.3; 51.8] |
| Tomlinson, 1991 | 1 | 19 | 0.2% | 5.3 [ 0.1; 26.0] |
| Wilson, 2011 | 100 | 3973 | 0.3% | 2.5 [ 2.1; 3.1] |
| Wilson, 2020 | 75 | 1793 | 0.3% | 4.2 [ 3.3; 5.2] |
| Ghanem, 2004 | 198 | 6600 | 0.3% | 3.0 [ 2.6; 3.4] |
| Ghanem, 2004 | 28 | 2545 | 0.3% | 1.1 [ 0.7; 1.6] |
| **Common effect model** | **57601** | **1962406** | **--** | **2.0 [ 2.0; 2.1]** |
| **Random effects model** | | | **100.0%** | **4.9 [ 4.2; 5.6]** |
| **Prediction interval** | | | | **[ 0.0; 24.4]** |

Heterogeneity: $I^2 = 99\%$, $\chi^2_{373} = 53523.49$ ($p = 0$)

0 20 40 60 80 100
Gonorrhea prevalence (95% CI)

# I. Symptomatic women

| | | | Events per 100 | | |
|---|---|---|---|---|---|
| Kamwendo, 1996 | 0 | 69 | | 1.0% | 0.0 [ 0.0; 5.2] |
| Kamwendo, 1996 | 0 | 39 | | 0.9% | 0.0 [ 0.0; 9.0] |
| Kamwendo, 1996 | 12 | 56 | | 0.9% | 21.4 [11.6; 34.4] |
| Kamwendo, 1996 | 13 | 88 | | 1.0% | 14.8 [ 8.1; 23.9] |
| Kamwendo, 1996 | 4 | 65 | | 0.9% | 6.2 [ 1.7; 15.0] |
| Kamwendo, 1996 | 1 | 44 | | 0.9% | 2.3 [ 0.1; 12.0] |
| Kamwendo, 1996 | 0 | 26 | | 0.8% | 0.0 [ 0.0; 13.2] |
| Kamwendo, 1996 | 8 | 57 | | 0.9% | 14.0 [ 6.3; 25.8] |
| Kamwendo, 1996 | 3 | 57 | | 0.9% | 5.3 [ 1.1; 14.6] |
| Kamwendo, 1996 | 6 | 80 | | 1.0% | 7.5 [ 2.8; 15.6] |
| Kamwendo, 1996 | 0 | 65 | | 0.9% | 0.0 [ 0.0; 5.5] |
| Kamwendo, 1996 | 0 | 54 | | 0.9% | 0.0 [ 0.0; 6.6] |
| Larsson, 1992 | 0 | 79 | | 1.0% | 0.0 [ 0.0; 4.6] |
| Romosan, 2013 | 0 | 36 | | 0.9% | 0.0 [ 0.0; 9.7] |
| Svensson, 1983 | 18 | 90 | | 1.0% | 20.0 [12.3; 29.8] |
| Svensson, 1983 | 14 | 101 | | 1.0% | 13.9 [ 7.8; 22.2] |
| Svensson, 1983 | 7 | 63 | | 0.9% | 11.1 [ 4.6; 21.6] |
| Svensson, 1983 | 7 | 45 | | 0.9% | 15.6 [ 6.5; 29.5] |
| Asicioglu, 2013 | 50 | 560 | | 1.1% | 8.9 [ 6.7; 11.6] |
| Asicioglu, 2013 | 54 | 543 | | 1.0% | 9.9 [ 7.6; 12.8] |
| Eksi, 2011 | 4 | 165 | | 1.0% | 2.4 [ 0.7; 6.1] |
| Eksi, 2011 | 2 | 165 | | 1.0% | 1.2 [ 0.1; 4.3] |
| Ozturk, 2006 | 1 | 828 | | 1.1% | 0.1 [ 0.0; 0.7] |
| Beckett, 2018 | 1 | 71 | | 1.0% | 1.4 [ 0.0; 7.6] |
| Beckett, 2018 | 0 | 71 | | 1.0% | 0.0 [ 0.0; 5.1] |
| Bevan, 1995 | 15 | 104 | | 1.0% | 14.4 [ 8.3; 22.7] |
| Cousins, 2015 | 3 | 273 | | 1.0% | 1.1 [ 0.2; 3.2] |
| Feeney, 1976 | 7 | 48 | | 0.9% | 14.6 [ 6.1; 27.8] |
| Harding-Esch, 2017 | 0 | 35 | | 0.9% | 0.0 [ 0.0; 10.0] |
| Jungmann, 2004 | 2 | 139 | | 1.0% | 1.4 [ 0.2; 5.1] |
| Morris, 2014 | 14 | 169 | | 1.0% | 8.3 [ 4.6; 13.5] |
| Nicholls, 2018 | 10 | 530 | | 1.0% | 1.9 [ 0.9; 3.4] |
| Nicholls, 2018 | 7 | 3499 | | 1.1% | 0.2 [ 0.1; 0.4] |
| Piyadigamage, 2005 | 18 | 147 | | 1.0% | 12.2 [ 7.4; 18.7] |
| Riordan, 1990 | 10 | 297 | | 1.0% | 3.4 [ 1.6; 6.1] |
| Shone, 2016 | 1 | 395 | | 1.0% | 0.3 [ 0.0; 1.4] |
| Stacey, 1992 | 7 | 23 | | 0.8% | 30.4 [13.2; 52.9] |
| Maleckiene, 2012 | 14 | 52 | | 0.9% | 26.9 [15.6; 41.0] |
| **Common effect model** | **1364** | **28485** | | **--** | **2.4 [ 2.2; 2.6]** |
| **Random effects model** | | | | **100.0%** | **5.6 [ 4.0; 7.4]** |
| **Prediction interval** | | | | | **[ 0.0; 31.9]** |

Heterogeneity: $I^2 = 96\%$, $\chi^2_{102} = 2566.37$ ($p = 0$)

0 20 40 60 80 100
Gonorrhea prevalence (95% CI)

| | | | | |
|---|---|---|---|---|
| Shipitsyna, 2009 | 6 | 319 | 1.0% | 1.9 [ 0.7; 4.0] |
| Bolumburu, 2020 | 0 | 139 | 1.0% | 0.0 [ 0.0; 2.6] |
| Pena, 1996 | 1 | 262 | 1.0% | 0.4 [ 0.0; 2.1] |
| Salva, 1986 | 9 | 17 | 0.7% | 52.9 [27.8; 77.0] |
| Salva, 1986 | 3 | 16 | 0.7% | 18.8 [ 4.0; 45.6] |
| Brihmer, 1987 | 21 | 187 | 1.0% | 11.2 [ 7.1; 16.7] |
| Brihmer, 1987 | 3 | 172 | 1.0% | 1.7 [ 0.4; 5.0] |
| Eggert, 2006 | 0 | 23 | 0.8% | 0.0 [ 0.0; 14.8] |
| Jacobson, 1969 | 223 | 623 | 1.1% | 35.8 [32.0; 39.7] |
| Kamwendo, 1996 | 50 | 182 | 1.0% | 27.5 [21.1; 34.6] |
| Kamwendo, 1996 | 32 | 192 | 1.0% | 16.7 [11.7; 22.7] |
| Kamwendo, 1996 | 18 | 129 | 1.0% | 14.0 [ 8.5; 21.2] |
| Kamwendo, 1996 | 5 | 94 | 1.0% | 5.3 [ 1.7; 12.0] |
| Kamwendo, 1996 | 0 | 28 | 0.8% | 0.0 [ 0.0; 12.3] |
| Kamwendo, 1996 | 46 | 198 | 1.0% | 23.2 [17.5; 29.7] |
| Kamwendo, 1996 | 36 | 255 | 1.0% | 14.1 [10.1; 19.0] |
| Kamwendo, 1996 | 11 | 143 | 1.0% | 7.7 [ 3.9; 13.3] |
| Kamwendo, 1996 | 7 | 118 | 1.0% | 5.9 [ 2.4; 11.8] |
| Kamwendo, 1996 | 0 | 69 | 1.0% | 0.0 [ 0.0; 5.2] |
| Kamwendo, 1996 | 33 | 132 | 1.0% | 25.0 [17.9; 33.3] |
| Kamwendo, 1996 | 18 | 147 | 1.0% | 12.2 [ 7.4; 18.7] |
| Kamwendo, 1996 | 13 | 97 | 1.0% | 13.4 [ 7.3; 21.8] |

## J. Symptomatic men

| Author, Year | Gonorrhea positive | Sample size | W(Random) | Prev(%) | 95% CI |
|---|---|---|---|---|---|
| Hovhannisyan, 2007 | 129 | 407 | 1.7% | 31.7 | [27.2; 36.5] |
| Libois, 2015 | 45 | 194 | 1.7% | 23.2 | [17.5; 29.8] |
| Ansart, 2009 | 18 | 37 | 1.5% | 48.6 | [31.9; 65.6] |
| Hermabessiere, 1977 | 1 | 66 | 1.6% | 1.5 | [ 0.0; 8.2] |
| Janier, 1990 | 1 | 62 | 1.6% | 1.6 | [ 0.0; 8.7] |
| Janier, 1995 | 25 | 219 | 1.7% | 11.4 | [ 7.5; 16.4] |
| Joly-Guillou, 1996 | 40 | 189 | 1.7% | 21.2 | [15.6; 27.7] |
| Lefevre, 1991 | 24 | 225 | 1.7% | 10.7 | [ 7.0; 15.5] |
| Akhvlediani, 2015 | 11 | 100 | 1.6% | 11.0 | [ 5.6; 18.8] |
| Akhvlediani, 2015 | 25 | 100 | 1.6% | 25.0 | [16.9; 34.7] |
| Durglishvili, 2013 | 16 | 54 | 1.6% | 29.6 | [18.0; 43.6] |
| Elsner, 1987 | 33 | 169 | 1.7% | 19.5 | [13.8; 26.3] |
| Marconi, 2009 | 0 | 38 | 1.5% | 0.0 | [ 0.0; 9.3] |
| Marconi, 2009 | 0 | 12 | 1.2% | 0.0 | [ 0.0; 26.5] |
| Marconi, 2009 | 8 | 21 | 1.4% | 38.1 | [18.1; 61.6] |
| Pilatz, 2015 | 2 | 89 | 1.6% | 2.2 | [ 0.3; 7.9] |
| Pilatz, 2015 | 4 | 48 | 1.6% | 8.3 | [ 2.3; 20.0] |
| Papadogeorgakis, 2010 | 30 | 100 | 1.6% | 30.0 | [21.2; 40.0] |
| Gottesman, 2017 | 58 | 118 | 1.7% | 49.2 | [39.8; 58.5] |
| Srugo, 2003 | 3 | 238 | 1.7% | 1.3 | [ 0.3; 3.6] |
| Zvulunov, 2000 | 1 | 169 | 1.7% | 0.6 | [ 0.0; 3.3] |
| Donati, 2009 | 62 | 157 | 1.7% | 39.5 | [31.8; 47.6] |
| Trevisan, 2008 | 3 | 26 | 1.4% | 11.5 | [ 2.4; 30.2] |
| Van Der Snoek, 2000 | 122 | 1849 | 1.7% | 6.6 | [ 5.5; 7.8] |
| Van Der Snoek, 2000 | 70 | 1810 | 1.7% | 3.9 | [ 3.0; 4.9] |
| Vergunst, 2019 | 87 | 379 | 1.7% | 23.0 | [18.8; 27.5] |
| Anestad, 1984 | 4 | 71 | 1.6% | 5.6 | [ 1.6; 13.8] |
| Guschin, 2015 | 29 | 271 | 1.7% | 10.7 | [ 7.3; 15.0] |
| Shipitsyna, 2009 | 4 | 127 | 1.7% | 3.1 | [ 0.9; 7.9] |
| Foronda-Garcia-Hidalgo, 2019 | 64 | 3066 | 1.7% | 2.1 | [ 1.6; 2.7] |
| Moreno-Ribera, 2016 | 45 | 119 | 1.7% | 37.8 | [29.1; 47.2] |
| Orellana, 2009 | 25 | 402 | 1.7% | 6.2 | [ 4.1; 9.0] |
| Orellana, 2009 | 34 | 442 | 1.7% | 7.7 | [ 5.4; 10.6] |
| Orellana, 2009 | 20 | 404 | 1.7% | 5.0 | [ 3.0; 7.5] |
| Orellana, 2011b | 30 | 270 | 1.7% | 11.1 | [ 7.6; 15.5] |
| Orellana, 2011c | 149 | 2021 | 1.7% | 7.4 | [ 6.3; 8.6] |
| Uria, 2012 | 392 | 5607 | 1.7% | 7.0 | [ 6.3; 7.7] |
| Varela, 2003 | 70 | 985 | 1.7% | 7.1 | [ 5.6; 8.9] |
| Varela, 2003 | 27 | 1116 | 1.7% | 2.4 | [ 1.6; 3.5] |
| Frolund, 2011 | 0 | 28 | 1.4% | 0.0 | [ 0.0; 12.3] |
| Agacfidan, 2001 | 18 | 192 | 1.7% | 9.4 | [ 5.7; 14.4] |
| Dolapci, 2005 | 6 | 63 | 1.6% | 9.5 | [ 3.6; 19.6] |
| Tekgul, 1992 | 6 | 78 | 1.6% | 7.7 | [ 2.9; 16.0] |
| Arya, 1986 | 29 | 56 | 1.6% | 51.8 | [38.0; 65.3] |
| Hanna, 1980 | 67 | 169 | 1.7% | 39.6 | [32.2; 47.4] |
| Hanna, 1980 | 74 | 169 | 1.7% | 43.8 | [36.2; 51.6] |
| Harding-Esch, 2017 | 1 | 35 | 1.5% | 2.9 | [ 0.1; 14.9] |
| Hill-Tout, 2018 | 2 | 46 | 1.5% | 4.3 | [ 0.5; 14.8] |
| Hirst, 2016 | 2 | 254 | 1.7% | 0.8 | [ 0.1; 2.8] |
| Khatib, 2015 | 14 | 83 | 1.6% | 16.9 | [ 9.5; 26.7] |
| Leung, 2006 | 27 | 302 | 1.7% | 8.9 | [ 6.0; 12.7] |
| Lomas, 1993 | 23 | 106 | 1.7% | 21.7 | [14.3; 30.8] |
| Pasieczny, 1966 | 114 | 565 | 1.7% | 20.2 | [16.9; 23.7] |
| Pearson, 1988 | 1 | 29 | 1.5% | 3.4 | [ 0.1; 17.8] |
| Pearson, 1988 | 31 | 90 | 1.6% | 34.4 | [24.7; 45.2] |
| Pond, 2017 | 7 | 79 | 1.6% | 8.9 | [ 3.6; 17.4] |
| Pond, 2017 | 1 | 83 | 1.6% | 1.2 | [ 0.0; 6.5] |
| Reynolds-Wright, 2016 | 1 | 38 | 1.5% | 2.6 | [ 0.1; 13.8] |
| Soni, 2010 | 10 | 438 | 1.7% | 2.3 | [ 1.1; 4.2] |
| Whitfield, 2013 | 3 | 84 | 1.6% | 3.6 | [ 0.7; 10.1] |
| Kostyakova, 2011 | 74 | 120 | 1.7% | 61.7 | [52.4; 70.4] |
| **Common effect model** | **2222** | **24884** | **--** | **7.1** | **[ 6.8; 7.5]** |
| **Random effects model** | | | **100.0%** | **12.1** | **[ 8.8; 15.8]** |
| **Prediction interval** | | | | | **[ 0.0; 48.6]** |

Heterogeneity: $I^2 = 97\%$, $\chi^2_{60} = 1906.61$ ($p = 0$)

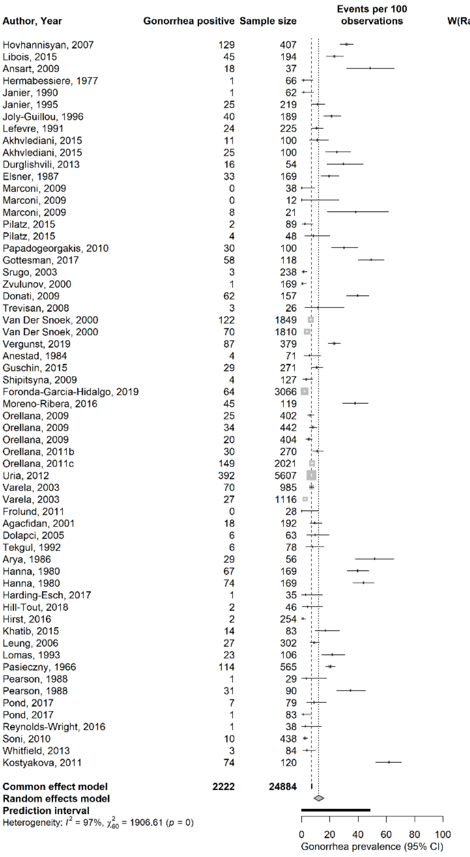


## K. Sexual contact of persons infected with *Neisseria gonorrhoeae*/*Chlamydia trachomatis*

| Author, Year | Gonorrhea positive | Sample size | W(Random) | Prev(%) | 95% CI |
|---|---|---|---|---|---|
| Magnusson, 1986 | 16 | 93 | 9.9% | 17.2 | [10.2; 26.4] |
| Oskarsson, 1990 | 23 | 207 | 10.1% | 11.1 | [ 7.2; 16.2] |
| Karp-Damdin, 1975 | 42 | 296 | 10.2% | 14.2 | [10.4; 18.7] |
| Lycke, 1980 | 80 | 156 | 10.0% | 51.3 | [43.2; 59.4] |
| Ahmed-Jushuf, 1988 | 10 | 37 | 9.2% | 27.0 | [13.8; 44.1] |
| Burton, 2021 | 50 | 107 | 9.9% | 46.7 | [37.0; 56.6] |
| Burton, 2021 | 22 | 143 | 10.0% | 15.4 | [ 9.9; 22.4] |
| Burton, 2021 | 9 | 183 | 10.1% | 4.9 | [ 2.3; 9.1] |
| Lavelle, 2006 | 8 | 473 | 10.2% | 1.7 | [ 0.7; 3.3] |
| Lavelle, 2006 | 47 | 4680 | 10.3% | 1.0 | [ 0.7; 1.3] |
| **Common effect model** | **307** | **6375** | **--** | **2.5** | **[ 2.1; 3.0]** |
| **Random effects model** | | | **100.0%** | **15.7** | **[ 6.5; 27.8]** |
| **Prediction interval** | | | | | **[ 0.0; 68.7]** |

Heterogeneity: $I^2 = 99\%$, $\chi^2_9 = 648.43$ ($p < 0.01$)

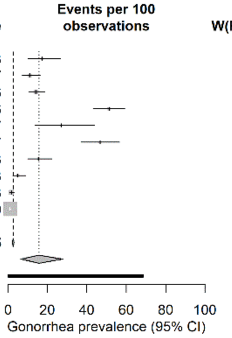

## L. Patients with confirmed/suspected sexually transmitted infections and related infections

| Author, Year | Gonorrhea positive | Sample size | W(Random) | Prev(%) | 95% CI |
|---|---|---|---|---|---|
| Granitzka, 1979 | 27 | 193 | 2.8% | 14.0 | [ 9.4; 19.7] |
| Granitzka, 1979 | 43 | 366 | 2.8% | 11.7 | [ 8.6; 15.5] |
| Granitzka, 1979 | 64 | 536 | 2.9% | 11.9 | [ 9.3; 15.0] |
| Granitzka, 1979 | 63 | 729 | 2.9% | 8.6 | [ 6.7; 10.9] |
| Granitzka, 1979 | 90 | 890 | 2.9% | 10.1 | [ 8.2; 12.3] |
| Granitzka, 1979 | 71 | 754 | 2.9% | 9.4 | [ 7.4; 11.7] |
| Granitzka, 1979 | 61 | 726 | 2.9% | 8.4 | [ 6.5; 10.7] |
| Granitzka, 1979 | 67 | 780 | 2.9% | 8.6 | [ 6.7; 10.8] |
| Granitzka, 1979 | 45 | 795 | 2.9% | 5.7 | [ 4.2; 7.5] |
| Granitzka, 1979 | 21 | 790 | 2.9% | 2.7 | [ 1.7; 4.0] |
| Stengrimsson, 1984 | 224 | 788 | 2.9% | 28.4 | [25.3; 31.7] |
| Stengrimsson, 1984 | 104 | 363 | 2.8% | 28.7 | [24.1; 33.6] |
| Nijhuis, 2020 | 5 | 91 | 2.6% | 5.5 | [ 1.8; 12.4] |
| Nijhuis, 2020 | 12 | 1607 | 2.9% | 0.7 | [ 0.4; 1.3] |
| Van Bergen, 2006a | 4 | 166 | 2.7% | 2.4 | [ 0.7; 6.1] |
| Van Rooijen, 2015 | 5 | 148 | 2.7% | 3.4 | [ 1.1; 7.7] |
| Van Rooijen, 2015 | 13 | 159 | 2.7% | 8.2 | [ 4.4; 13.6] |
| Hovding, 1977 | 296 | 906 | 2.9% | 32.7 | [29.6; 35.8] |
| Tishchenko, 1988 | 0 | 240 | 2.8% | 0.0 | [ 0.0; 1.5] |
| Adelantado Lacasa, 2019 | 5 | 173 | 2.7% | 2.9 | [ 0.9; 6.6] |
| Orduna Domingo, 1991 | 113 | 1625 | 2.9% | 7.0 | [ 5.8; 8.3] |
| Orduna Domingo, 1991 | 460 | 3451 | 2.9% | 13.3 | [12.2; 14.5] |
| Perez-Torralba, 2021 | 91 | 4983 | 2.9% | 1.8 | [ 1.5; 2.2] |
| Perez-Torralba, 2021 | 23 | 4983 | 2.9% | 0.5 | [ 0.3; 0.7] |
| Perez-Torralba, 2021 | 75 | 865 | 2.9% | 8.7 | [ 6.9; 10.7] |
| Perez-Torralba, 2021 | 9 | 865 | 2.9% | 1.0 | [ 0.5; 2.0] |
| Ruiz-Olivares, 2020 | 15 | 589 | 2.9% | 2.5 | [ 1.4; 4.2] |
| Ruiz-Olivares, 2020 | 22 | 624 | 2.9% | 3.5 | [ 2.2; 5.3] |
| Harindra, 2002 | 13 | 282 | 2.8% | 4.6 | [ 2.5; 7.8] |
| Harindra, 2002 | 13 | 207 | 2.8% | 6.3 | [ 3.4; 10.5] |
| Hussey, 2003 | 1 | 15 | 1.6% | 6.7 | [ 0.2; 31.9] |
| Manavi, 2004 | 2 | 32 | 2.1% | 6.2 | [ 0.8; 20.8] |
| Reynolds, 1996 | 51 | 251 | 2.8% | 20.3 | [15.5; 25.8] |
| Reynolds, 1996 | 8 | 83 | 2.5% | 9.6 | [ 4.3; 18.1] |
| Rosenvinge, 2009 | 38 | 559 | 2.9% | 6.8 | [ 4.9; 9.2] |
| Oborin, 2020 | 23 | 102 | 2.6% | 22.5 | [14.9; 31.9] |
| **Common effect model** | **2177** | **30716** | **--** | **5.1** | **[ 4.9; 5.4]** |
| **Random effects model** | | | **100.0%** | **7.5** | **[ 5.2; 10.2]** |
| **Prediction interval** | | | | | **[ 0.0; 28.4]** |

Heterogeneity: $I^2 = 99\%$, $\chi^2_{35} = 2555.15$ ($p = 0$)

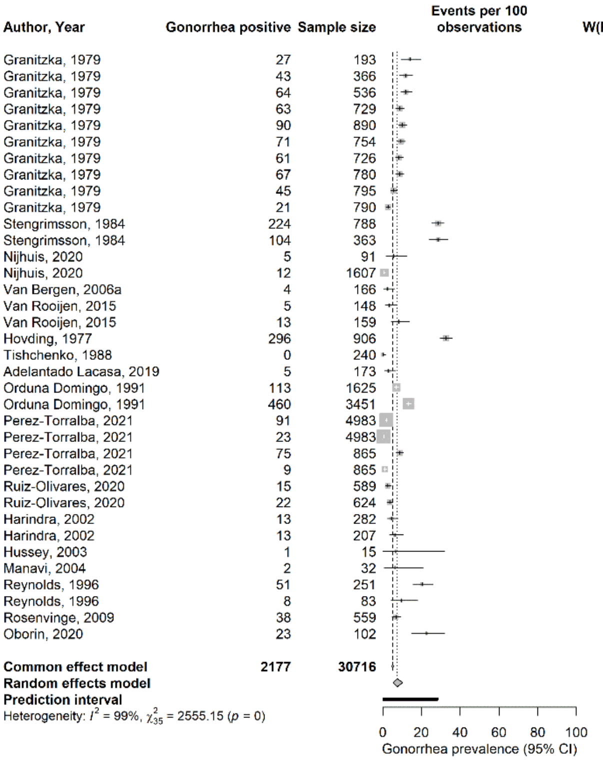

**Supplementary Table S5. Univariable and multivariable meta-regression analyses for *Neisseria gonorrhoeae* prevalence in urogenital specimens in Europe using the year of publication instead of the year of data collection as the time variable.**

| Urogenital specimens | | | Outcome measures | Sample size | Univariable analysis | | | | Multivariable analyses | | | |
|---|---|---|---|---|---|---|---|---|---|---|---|---|
| | | | | | | | | | Model 3[a] | | Model 4[b] | |
| | | | Total n | Total N | *RR* (95% CI) | p-value | LT test p-value | Adjusted $R^2$ | *ARR* (95% CI) | p-value | *ARR* (95% CI) | p-value |
| Population characteristics | Population type | General populations | 262 | 64,4913 | 1.00 | - | <0.001 | 18.84 | 1.00 | - | 1.00 | - |
| | | Intermediate-risk populations | 24 | 6,559 | 2.54 (1.28-5.04) | 0.008 | | | 3.05 (1.61-5.76) | 0.001 | 2.49 (1.33-4.66) | 0.004 |
| | | FSWs | 31 | 10,021 | 3.28 (1.96-5.47) | <0.001 | | | 4.10 (2.56-6.58) | <0.001 | 3.80 (2.39-6.04) | <0.001 |
| | | MSWs, MSM, and transgender people | 16 | 6,436 | 0.95 (0.45-2.01) | 0.887 | | | 1.29 (0.63-2.61) | 0.483 | 1.35 (0.67-2.71) | 0.401 |
| | | Infertility clinic attendees | 51 | 7,150 | 2.58 (1.38-4.80) | 0.003 | | | 1.84 (1.02-3.32) | 0.042 | 1.49 (0.83-2.68) | 0.178 |
| | | Symptomatic women | 103 | 28,485 | 4.11 (2.97-5.69) | <0.001 | | | 3.25 (2.38-4.45) | <0.001 | 3.33 (2.45-4.53) | <0.001 |
| | | Symptomatic men | 61 | 24,884 | 7.19 (4.99-10.30) | <0.001 | | | 5.50 (3.72-8.12) | <0.001 | 5.27 (3.60-7.73) | <0.001 |
| | | STI clinic attendees | 374 | 1,962,406 | 2.91 (2.33-3.65) | <0.001 | | | 3.13 (2.50-3.91) | <0.001 | 3.03 (2.43-3.77) | <0.001 |
| | | HIV positive individuals and individuals in HIV discordant couples | 19 | 5,069 | 0.85 (0.35-2.04) | 0.714 | | | 1.03 (0.45-2.33) | 0.949 | 0.94 (0.42-2.12) | 0.888 |
| | | Sexual contacts of persons infected with NG/CT | 10 | 6,375 | 7.83 (3.67-16.60) | <0.001 | | | 8.20 (4.14-16.20) | <0.001 | 7.15 (3.64-14.00) | <0.001 |
| | | Patients with confirmed or suspected STIs and related infections | 36 | 30,716 | 4.53 (2.93-7.01) | <0.001 | | | 4.70 (3.14-7.03) | <0.001 | 4.68 (3.15-6.95) | <0.001 |
| | | Other populations[c] | 25 | 23,715 | 2.83 (1.67-4.81) | <0.001 | | | 4.10 (2.53-6.65) | <0.001 | 3.67 (2.29-5.90) | <0.001 |
| | Age group | <20 years | 50 | 11,635 | 1.00 | - | 0.007 | 1.53 | 1.00 | - | 1.00 | - |
| | | 20-29 years | 34 | 16,002 | 0.74 (0.39-1.41) | 0.359 | | | 0.85 (0.50-1.44) | 0.539 | 0.90 (0.53-1.52) | 0.696 |
| | | 30-39 years | 20 | 5,386 | 0.99 (0.43-2.26) | 0.979 | | | 1.27 (0.65-2.47) | 0.486 | 1.41 (0.73-2.73) | 0.305 |
| | | ≥40 years | 18 | 4,012 | 0.82 (0.37-1.85) | 0.639 | | | 1.67 (0.84-3.30) | 0.142 | 2.00 (1.02-3.92) | 0.045 |
| | | Mixed ages | 890 | 2,719,694 | 0.53 (0.35-0.80) | 0.003 | | | 0.36 (0.25-0.52) | <0.001 | 0.40 (0.28-0.58) | <0.001 |
| | Sex | Women | 558 | 1,154,985 | 1.00 | - | <0.001 | 2.40 | 1.00 | - | 1.00 | - |
| | | Men | 371 | 933,280 | 1.62 (1.34-1.98) | <0.001 | | | 1.41 (1.15-1.72) | 0.001 | 1.46 (1.20-1.78) | <0.001 |
| | | Mixed sexes | 83 | 668,464 | 1.21 (0.88-1.65) | 0.239 | | | 1.33 (1.01-1.75) | 0.042 | 1.26 (0.96-1.65) | 0.095 |
| | European Subregions | Eastern Europe | 77 | 281,396 | 1.00 | - | 0.922 | 0.00 | - | - | - | - |
| | | Southern Europe | 121 | 72,030 | 1.08 (0.70-1.67) | 0.724 | | | - | - | - | - |
| | | Western Europe | 330 | 1,242,348 | 0.96 (0.66-1.38) | 0.816 | | | - | - | - | - |
| | | Northern Europe | 424 | 1,136,092 | 1.04 (0.73-1.50) | 0.815 | | | - | - | - | - |
| | | Israel, Turkey, and mixed regions | 60 | 24,863 | 1.06 (0.62-1.81) | 0.838 | | | - | - | - | - |
| | Country's Income | LMIC | 7 | 1,353 | 1.00 | - | 0.021 | 1.01 | 1.00 | - | 1.00 | - |
| | | UMIC | 76 | 279,505 | 2.16 (0.68-6.89) | 0.193 | | | 2.30 (0.84-6.30) | 0.104 | 1.53 (0.57-4.09) | 0.396 |
| | | HIC | 927 | 2,467,907 | 1.93 (0.63-5.88) | 0.246 | | | 1.53 (0.58-4.01) | 0.388 | 1.09 (0.42-2.82) | 0.862 |
| | | Mixed income | 2 | 7,964 | 0.14 (0.02-1.17) | 0.069 | | | 0.43 (0.07-2.55) | 0.351 | 0.28 (0.05-1.62) | 0.156 |
| Study methodology characteristics | Assay type | NAAT/PCR | 427 | 1,448,120 | 1.00 | - | <0.001 | 6.31 | 1.00 | - | 1.00 | - |
| | | Culture | 453 | 985,482 | 1.91 (1.57-2.32) | <0.001 | | | 1.02 (0.80-1.32) | 0.849 | 0.87 (0.69-1.10) | 0.232 |
| | | Gram Staining | 32 | 247,107 | 2.52 (1.53-4.14) | <0.001 | | | 1.36 (0.87-2.14) | 0.178 | 1.01 (0.64-1.59) | 0.954 |
| | | Other/unclear | 100 | 76,020 | 1.79 (1.30-2.46) | <0.001 | | | 0.98 (0.73-1.31) | 0.873 | 0.88 (0.66-1.17) | 0.381 |
| | Sample size[d] | <200 | 189 | 14,814 | 1.00 | - | <0.001 | 7.82 | 1.00 | - | 1.00 | - |
| | | ≥200 | 823 | 2,741,915 | 0.38 (0.29-0.49) | <0.001 | | | 0.44 (0.35-0.56) | <0.001 | 0.44 (0.35-0.55) | <0.001 |

| | | | | | | | | | | | | |
|---|---|---|---|---|---|---|---|---|---|---|---|---|
| | **Sampling method** | Probability based | 39 | 15,610 | 1.00 | - | 0.106 | 0.00 | - | - | - | - |
| | | Non-probability based | 973 | 2,741,119 | 1.73 (0.89-3.34) | 0.106 | | | - | - | - | - |
| | **Response rate** | ≥80% | 62 | 44,933 | 1.00 | - | <0.001 | 1.44 | 1.00 | - | 1.00 | - |
| | | <80% | 68 | 30,509 | 1.05 (0.58-1.89) | 0.874 | | | 0.82 (0.50-1.36) | 0.450 | 0.76 (0.47-1.25) | 0.286 |
| | | Unclear | 882 | 2,681,287 | 1.99 (1.33-2.98) | 0.001 | | | 1.34 (0.95-1.88) | 0.092 | 1.30 (0.93-1.82) | 0.129 |
| **Temporal trend** | **Year of publication category** | <2005 | 455 | 949,433 | 1.00 | - | <0.001 | 6.57 | 1.00 | - | - | - |
| | | 2005-2014 | 258 | 874,728 | 0.49 (0.39-0.61) | <0.001 | | | 0.46 (0.36-0.59) | <0.001 | - | - |
| | | ≥2015 | 299 | 932,568 | 0.54 (0.43-0.67) | <0.001 | | | 0.59 (0.45-0.78) | <0.001 | - | - |
| | **Year of publication** | | 1,012 | 2,756,729 | 0.97 (0.97-0.98) | <0.001 | <0.001 | 9.83 | - | - | 0.97 (0.96-0.98) | <0.001 |

[a]Adjusted $R^2$ in the final multivariable model 3 = 37.24%.
[b]Adjusted $R^2$ in the final multivariable model 4 = 39.19%.
[c]Other populations include populations with an undetermined risk of acquiring *Neisseria gonorrhoeae* infection such as patients with cervical cancer, victims of sexual assault, specimens from virology/bacteriology laboratory and requesting home-based *N. gonorrhoeae* or *Chlamydia trachomatis* testing.
[d]Sample size denotes the sample size of each study population at the baseline found in the original publication.
Abbreviations: *ARR*: Adjusted risk ratio; CI: Confidence interval; CT: *Chlamydia trachomatis*; FSWs: Female sex workers; HIC: High-income country; PCR: Polymerase chain reaction; MSM: Men who have sex with men; MSWs: Male sex workers; NAAT: Nucleic acid amplification test; NG: *Neisseria gonorrhoeae*; LMIC: Low-middle income country; LT test: Likelihood ratio test; *RR*: Risk ratio; STI: Sexually transmitted infection; UMIC: Upper-middle income country.

**Supplementary Table S6. Univariable and multivariable meta-regression analyses for *Neisseria gonorrhoeae* prevalence in anorectal specimens in Europe using the year of publication instead of the year of data collection as the time variable.**

| Anorectal specimens | | | Outcome measures | Sample size | Univariable analysis | | | | Multivariable analyses | | | |
|---|---|---|---|---|---|---|---|---|---|---|---|---|
| | | | | | | | | | Model 3[a] | | Model 4[b] | |
| | | | Total n | Total N | *RR* (95% CI) | p-value | LT test p-value | Adjusted $R^2$ | *ARR* (95% CI) | p-value | *ARR* (95% CI) | p-value |
| Population characteristics | Population type | MSWs, MSM, and transgender[c] | 15 | 9,893 | 1.00 | - | <0.001 | 12.87 | 1.00 | - | 1.00 | - |
| | | General populations | 3 | 1,258 | 0.41 (0.11-1.55) | 0.187 | | | 1.33 (0.39-4.54) | 0.644 | 1.23 (0.35-4.27) | 0.743 |
| | | Intermediate-risk populations | 2 | 141 | 4.01 (0.46-34.70) | 0.207 | | | 9.84 (1.42-68.10) | 0.021 | 9.08 (1.28-64.60) | 0.028 |
| | | FSWs | 5 | 2,440 | 0.50 (0.16-1.61) | 0.244 | | | 1.11 (0.37-3.32) | 0.850 | 1.01 (0.33-3.05) | 0.988 |
| | | Symptomatic women | 5 | 3,813 | 0.27 (0.09-0.81) | 0.020 | | | 1.47 (0.44-4.88) | 0.526 | 1.16 (0.35-3.84) | 0.813 |
| | | Symptomatic men | 13 | 8,398 | 1.30 (0.59-2.85) | 0.512 | | | 1.11 (0.55-2.23) | 0.775 | 1.10 (0.54-2.24) | 0.786 |
| | | STI clinic attendees | 137 | 739,542 | 0.86 (0.49-1.52) | 0.597 | | | 1.30 (0.78-2.18) | 0.315 | 1.21 (0.72-2.04) | 0.469 |
| | | HIV positive individuals and individuals in HIV discordant couples | 21 | 4,672 | 1.05 (0.51-2.19) | 0.887 | | | 0.92 (0.48-1.77) | 0.809 | 0.88 (0.46-1.70) | 0.708 |
| | | Sexual contacts of persons infected with NG/CT | 5 | 467 | 2.97 (0.98-9.00) | 0.054 | | | 3.41 (1.25-9.33) | 0.017 | 3.56 (1.29-9.85) | 0.015 |
| | | Patients with confirmed or suspected STIs and related infections | 16 | 2,605 | 3.13 (1.44-6.78) | 0.004 | | | 3.29 (1.65-6.57) | 0.001 | 3.18 (1.58-6.42) | 0.001 |
| | | Other populations[d] | 7 | 1,348 | 1.17 (0.42-3.25) | 0.762 | | | 2.21 (0.87-5.64) | 0.095 | 2.25 (0.87-5.79) | 0.093 |
| | Age group | ≤30 years | 8 | 1,165 | 1.00 | - | 0.559 | 0.00 | - | - | - | - |
| | | >30 years | 6 | 498 | 1.41 (0.34-5.85) | 0.631 | | | - | - | - | - |
| | | Mixed ages | 215 | 772,914 | 0.80 (0.34-1.89) | 0.615 | | | - | - | - | - |
| | Sex | Women | 52 | 176,849 | 1.00 | - | <0.001 | 11.86 | 1.00 | - | 1.00 | - |
| | | Men | 167 | 579,396 | 2.62 (1.83-3.75) | <0.001 | | | 2.99 (2.05-4.35) | <0.001 | 2.74 (1.88-3.99) | <0.001 |
| | | Mixed sexes | 10 | 18,332 | 2.05 (0.99-4.24) | 0.052 | | | 2.05 (1.07-3.93) | 0.031 | 2.10 (1.09-4.06) | 0.027 |
| | European Subregions | Eastern Europe | 6 | 2,132 | 1.00 | - | 0.004 | 5.83 | 1.00 | - | 1.00 | - |
| | | Southern Europe | 24 | 11,557 | 3.39 (1.19-9.64) | 0.023 | | | 0.71 (0.24-2.10) | 0.538 | 0.61 (0.21-1.84) | 0.383 |
| | | Western Europe | 120 | 633,385 | 1.58 (0.61-4.14) | 0.347 | | | 0.77 (0.29-2.06) | 0.607 | 0.66 (0.24-1.78) | 0.409 |
| | | Northern Europe | 78 | 127,487 | 2.25 (0.85-5.97) | 0.101 | | | 0.96 (0.35-2.63) | 0.932 | 0.82 (0.30-2.29) | 0.708 |
| | | Israel, Turkey, and mixed regions | 1 | 16 | 12.30 (1.17-129.80) | 0.037 | | | 2.08 (0.24-17.90) | 0.504 | 1.86 (0.21-16.60) | 0.575 |
| | Country's Income | UMIC | 4 | 1,342 | 1.00 | - | 0.784 | 0.00 | - | - | - | - |
| | | HIC | 225 | 773,235 | 1.18 (0.37-3.76) | 0.784 | | | - | - | - | - |
| Study methodology characteristics | Assay type | NAAT/PCR | 121 | 593,512 | 1.00 | - | 0.288 | 0.41 | - | - | - | - |
| | | Culture | 65 | 129,889 | 0.74 (0.52-1.06) | 0.098 | | | - | - | - | - |
| | | Gram Staining | 4 | 9,857 | 0.80 (0.25-2.53) | 0.707 | | | - | - | - | - |
| | | Other/unclear | 39 | 41,319 | 1.10 (0.73-1.65) | 0.661 | | | - | - | - | - |
| | Sample size[e] | <200 | 38 | 2,715 | 1.00 | - | <0.001 | 11.42 | 1.00 | - | 1.00 | - |
| | | ≥200 | 191 | 771,862 | 0.38 (0.26-0.56) | <0.001 | | | 0.48 (0.33-0.69) | <0.001 | 0.46 (0.31-0.67) | <0.001 |
| | Sampling method | Probability based | 4 | 1,154 | 1.00 | - | 0.927 | 0.00 | - | - | - | - |
| | | Non-probability based | 225 | 773,423 | 0.95 (0.32-2.85) | 0.927 | | | - | - | - | - |
| | | ≥80% | 7 | 22,936 | 1.00 | - | 0.509 | 0.00 | - | - | - | - |

| | | | | | | | | | | | | |
|---|---|---|---|---|---|---|---|---|---|---|---|---|
| | **Response rate** | <80% | 16 | 5,859 | 0.95 (0.32-2.78) | 0.925 | | | - | - | - | - |
| | | Unclear | 206 | 745,782 | 1.32 (0.54-3.21) | 0.536 | | | - | - | - | - |
| **Temporal trend** | **Year of publication category** | <2005 | 50 | 130,383 | 1.00 | - | <0.001 | 7.60 | 1.00 | - | - | - |
| | | 2005-2014 | 61 | 133,879 | 1.45 (0.95-2.21) | 0.088 | | | 1.54 (1.06-2.23) | 0.024 | - | - |
| | | ≥2015 | 118 | 310,315 | 2.11 (1.47-3.03) | <0.001 | | | 2.28 (1.61-3.24) | <0.001 | - | - |
| | **Year of publication** | | 229 | 774,577 | 1.02 (1.01-1.03) | 0.001 | 0.001 | 5.40 | - | - | 1.02 (1.01-1.04) | <0.001 |

[a]Adjusted $R^2$ in the final multivariable model 3 = 37.63%.
[b]Adjusted $R^2$ in the final multivariable model 4 = 35.04%.
[c]MSM, MSW, and transgender people group was used as a reference because of epidemiological relevance and because the general populations group had small number of measures.
[d]Other populations include populations with an undetermined risk of acquiring *Neisseria gonorrhoeae* infection such as patients with cervical cancer, victims of sexual assault, specimens from virology/bacteriology laboratory and requesting home-based *N. gonorrhoeae* or *Chlamydia trachomatis* testing.
[e]Sample size denotes the sample size of each study population at the baseline found in the original publication.
Abbreviations: *ARR*: Adjusted risk ratio; CI: Confidence interval; CT: *Chlamydia trachomatis*; FSWs: Female sex workers; HIC: High-income country; PCR: Polymerase chain reaction; MSM: Men who have sex with men; MSWs: Male sex workers; NAAT: Nucleic acid amplification test; NG: *Neisseria gonorrhoeae*; LT test: Likelihood ratio test; *RR*: Risk ratio; STI: Sexually transmitted infection; UMIC: Upper-middle income country.

**Supplementary Table S7. Univariable and multivariable meta-regression analyses for *Neisseria gonorrhoeae* prevalence in oropharyngeal specimens in Europe using the year of publication instead of the year of data collection as the time variable.**

| Oropharyngeal specimens | | | Outcome measures | Sample size | Univariable analysis | | | | Multivariable analyses | | | |
|---|---|---|---|---|---|---|---|---|---|---|---|---|
| | | | | | | | | | Model 3[a] | | Model 4[b] | |
| | | | Total n | Total N | *RR* (95% CI) | p-value | LT test p-value | Adjusted $R^2$ | *ARR* (95% CI) | p-value | *ARR* (95% CI) | p-value |
| Population characteristics | Population type[c] | MSWs, MSM, and transgender[d] | 19 | 11,395 | 1.00 | - | 0.266 | 1.56 | 1.00 | - | 1.00 | - |
| | | General populations | 3 | 475 | 1.08 (0.14-8.34) | 0.936 | | | 1.62 (0.25-10.60) | 0.614 | 1.56 (0.24-10.20) | 0.642 |
| | | FSWs | 7 | 2,949 | 0.84 (0.31-2.28) | 0.738 | | | 1.64 (0.64-4.22) | 0.298 | 1.58 (0.62-4.05) | 0.340 |
| | | STI clinic attendees | 107 | 394,371 | 1.08 (0.63-1.82) | 0.772 | | | 1.58 (0.98-2.55) | 0.060 | 1.55 (0.96-2.49) | 0.071 |
| | | HIV positive individuals and individuals in HIV discordant couples | 14 | 3,510 | 0.87 (0.39-1.95) | 0.752 | | | 0.85 (0.42-1.76) | 0.668 | 0.78 (0.39-1.58) | 0.493 |
| | | Sexual contacts of persons infected with NG/CT | 3 | 433 | 4.37 (1.26-15.10) | 0.020 | | | 5.10 (1.73-14.90) | 0.003 | 5.05 (1.72-14.80) | 0.003 |
| | | Patients with confirmed or suspected STIs and related infections | 6 | 469 | 1.73 (0.57-5.28) | 0.329 | | | 2.83 (1.05-7.65) | 0.041 | 2.82 (1.04-7.60) | 0.041 |
| | | Other populations[e] | 8 | 1,460 | 1.76 (0.70-4.45) | 0.224 | | | 2.58 (1.10-6.06) | 0.029 | 2.62 (1.12-6.12) | 0.026 |
| | Age group | ≤30 years | 6 | 664 | 1.00 | - | 0.253 | 0.45 | - | - | - | - |
| | | >30 years | 4 | 462 | 0.83 (0.16-4.37) | 0.832 | | | - | - | - | - |
| | | Mixed ages | 157 | 413,936 | 0.50 (0.20-1.22) | 0.129 | | | - | - | - | - |
| | Sex | Women | 43 | 151,890 | 1.00 | - | 0.020 | 5.66 | 1.00 | - | 1.00 | - |
| | | Men | 116 | 241,147 | 1.70 (1.15-2.50) | 0.007 | | | 2.71 (1.82-4.03) | <0.001 | 2.63 (1.76-3.92) | <0.001 |
| | | Mixed sexes | 8 | 22,025 | 1.12 (0.51-2.45) | 0.767 | | | 1.50 (0.71-3.19) | 0.288 | 1.52 (0.72-3.22) | 0.275 |
| | European Subregions | Eastern Europe | 3 | 1,198 | 1.00 | - | 0.979 | 0.00 | - | - | - | - |
| | | Southern Europe | 19 | 8,217 | 1.36 (0.35-5.30) | 0.648 | | | - | - | - | - |
| | | Western Europe | 85 | 379,721 | 1.31 (0.36-4.72) | 0.668 | | | - | - | - | - |
| | | Northern Europe | 57 | 23,580 | 1.43 (0.39-5.21) | 0.579 | | | - | - | - | - |
| | | Israel, Turkey, and mixed regions | 3 | 2,346 | 1.25 (0.22-7.05) | 0.793 | | | - | - | - | - |
| | Country's Income | UMIC | 3 | 1,198 | 1.00 | - | 0.629 | 0.00 | - | - | - | - |
| | | HIC | 164 | 413,864 | 1.35 (0.38-4.76) | 0.629 | | | - | - | - | - |
| Study methodology characteristics | Assay type | NAAT/PCR | 102 | 359,846 | 1.00 | - | 0.029 | 4.14 | 1.00 | - | 1.00 | - |
| | | Culture | 40 | 29,580 | 0.56 (0.37-0.85) | 0.008 | | | 0.60 (0.35-1.05) | 0.073 | 0.58 (0.34-1.01) | 0.054 |
| | | Gram Staining | 3 | 2,544 | 0.44 (0.13-1.46) | 0.181 | | | 0.42 (0.14-1.23) | 0.113 | 0.44 (0.15-1.29) | 0.132 |
| | | Other/unclear | 22 | 23,092 | 1.05 (0.65-1.71) | 0.822 | | | 0.69 (0.43-1.10) | 0.116 | 0.71 (0.45-1.13) | 0.145 |
| | Sample size[f] | <200 | 18 | 1,557 | 1.00 | - | 0.011 | 5.10 | 1.00 | - | 1.00 | - |
| | | ≥200 | 149 | 413,505 | 0.48 (0.27-0.84) | 0.011 | | | 0.42 (0.25-0.71) | 0.001 | 0.41 (0.24-0.69) | 0.001 |
| | Sampling method | Probability based | 4 | 1,118 | 1.00 | - | 0.505 | 0.00 | - | - | - | - |
| | | Non-probability based | 163 | 413,944 | 1.43 (0.49-4.20) | 0.505 | | | - | - | - | - |
| | Response rate | ≥80% | 9 | 3,826 | 1.00 | - | 0.156 | 1.10 | - | - | - | - |
| | | <80% | 11 | 3,909 | 0.36 (0.12-1.01) | 0.054 | | | - | - | - | - |
| | | Unclear | 147 | 407,327 | 0.59 (0.27-1.29) | 0.192 | | | - | - | - | - |

| | | | | | | | | | | | | |
|---|---|---|---|---|---|---|---|---|---|---|---|---|
| **Temporal trend** | **Year of publication category** | <2005 | 27 | 9,573 | 1.00 | - | <0.001 | 6.20 | 1.00 | - | - | - |
| | | 2005-2014 | 48 | 150,948 | 1.57 (0.92-2.67) | 0.094 | | | 1.34 (0.75-2.41) | 0.323 | - | - |
| | | ≥2015 | 92 | 254,541 | 2.25 (1.39-3.62) | <0.001 | | | 1.76 (0.92-3.36) | 0.087 | - | - |
| | **Year of publication** | | 167 | 415,062 | 1.03 (1.01-1.04) | 0.001 | 0.001 | 8.00 | - | - | 1.02 (1.00-1.04) | 0.107 |

[a]Adjusted $R^2$ in the final multivariable model 3 = 30.02%.
[b]Adjusted $R^2$ in the final multivariable model 4 = 30.23%.
[c]Population classification was included in the multivariable analyses for epidemiological relevance.
[d]MSM, MSW, and transgender people group was used as a reference because of epidemiological relevance and because the general populations group had small number of measures.
[e]Other populations include populations with an undetermined risk of acquiring *Neisseria gonorrhoeae* infection such as patients with cervical cancer, victims of sexual assault, specimens from virology/bacteriology laboratory and requesting home-based *N. gonorrhoeae* or *Chlamydia trachomatis* testing.
[f]Sample size denotes the sample size of each study population at the baseline found in the original publication.
Abbreviations: *ARR*: Adjusted risk ratio; CI: Confidence interval; CT: *Chlamydia trachomatis*; FSWs: Female sex workers; HIC: High-income country; PCR: Polymerase chain reaction; MSM: Men who have sex with men; MSWs: Male sex workers; NAAT: Nucleic acid amplification test; NG: *Neisseria gonorrhoeae*; LT test: Likelihood ratio test; *RR*: Risk ratio; STI: Sexually transmitted infection; UMIC: Upper-middle income country.

**Supplementary Figure S2. Forest plots displaying the results of cumulative meta-analyses, utilizing the year of publication as the ordering variable, to confirm the trend in *Neisseria gonorrhoeae* prevalence identified by the meta-regression analyses.**

**A. Urogenital[a]**

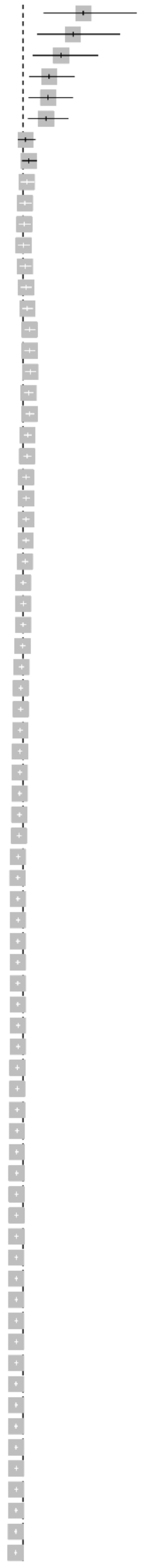

| Study | Proportion | 95%-CI | P-value | Tau2 | Tau | I2 |
|---|---|---|---|---|---|---|
| Adding Robertson, 1970 (k=1) | 0.06 | [0.03; 0.10] | . | . | . | . |
| Adding Robertson, 1970 (k=2) | 0.05 | [0.02; 0.09] | . | 0 | 0 | 0% |
| Adding Robertson, 1970 (k=3) | 0.04 | [0.02; 0.07] | . | 0.0003 | 0.0174 | 0% |
| Adding Robertson, 1970 (k=4) | 0.03 | [0.01; 0.05] | . | 0.0011 | 0.0332 | 15% |
| Adding Robertson, 1970 (k=5) | 0.03 | [0.01; 0.05] | . | 0.0007 | 0.0273 | 0% |
| Adding Robertson, 1970 (k=6) | 0.03 | [0.01; 0.04] | . | 0.0005 | 0.0223 | 0% |
| Adding Mesko, 1972 (k=7) | 0.01 | [0.01; 0.02] | . | 0.0022 | 0.0473 | 57% |
| Adding Gregersen, 1972 (k=8) | 0.01 | [0.01; 0.02] | . | 0.0016 | 0.0395 | 53% |
| Adding Nielsen, 1974 (k=9) | 0.01 | [0.01; 0.02] | . | 0.0020 | 0.0442 | 58% |
| Adding Nielsen, 1974 (k=10) | 0.01 | [0.01; 0.02] | . | 0.0026 | 0.0514 | 65% |
| Adding Nielsen, 1974 (k=11) | 0.01 | [0.01; 0.01] | . | 0.0026 | 0.0506 | 63% |
| Adding Nielsen, 1974 (k=12) | 0.01 | [0.01; 0.01] | . | 0.0024 | 0.0487 | 60% |
| Adding Nielsen, 1974 (k=13) | 0.01 | [0.01; 0.02] | . | 0.0024 | 0.0486 | 60% |
| Adding Nielsen, 1974 (k=14) | 0.01 | [0.01; 0.02] | . | 0.0020 | 0.0447 | 58% |
| Adding Nielsen, 1974 (k=15) | 0.01 | [0.01; 0.02] | . | 0.0017 | 0.0416 | 56% |
| Adding Nielsen, 1974 (k=16) | 0.01 | [0.01; 0.02] | . | 0.0019 | 0.0433 | 60% |
| Adding Nielsen, 1974 (k=17) | 0.01 | [0.01; 0.02] | . | 0.0016 | 0.0402 | 57% |
| Adding Nielsen, 1974 (k=18) | 0.01 | [0.01; 0.02] | . | 0.0015 | 0.0382 | 55% |
| Adding Nielsen, 1974 (k=19) | 0.01 | [0.01; 0.02] | . | 0.0015 | 0.0381 | 57% |
| Adding Andersen, 1974 (k=20) | 0.01 | [0.01; 0.02] | . | 0.0013 | 0.0354 | 56% |
| Adding Andersen, 1974 (k=21) | 0.01 | [0.01; 0.02] | . | 0.0014 | 0.0376 | 61% |
| Adding Hilton, 1974 (k=22) | 0.01 | [0.01; 0.02] | . | 0.0014 | 0.0374 | 60% |
| Adding Benjamin, 1978 (k=23) | 0.01 | [0.01; 0.01] | . | 0.0014 | 0.0378 | 62% |
| Adding Singha, 1979 (k=24) | 0.01 | [0.01; 0.01] | . | 0.0014 | 0.0370 | 60% |
| Adding Singha, 1979 (k=25) | 0.01 | [0.01; 0.01] | . | 0.0014 | 0.0379 | 61% |
| Adding Jensen, 1979 (k=26) | 0.01 | [0.01; 0.01] | . | 0.0014 | 0.0369 | 59% |
| Adding Torgal-Garcia, 1980 (k=27) | 0.01 | [0.01; 0.01] | . | 0.0016 | 0.0406 | 63% |
| Adding Querido, 1980 (k=28) | 0.01 | [0.01; 0.01] | . | 0.0018 | 0.0424 | 67% |
| Adding Van Der Lugt, 1980 (k=29) | 0.01 | [0.01; 0.01] | . | 0.0017 | 0.0410 | 66% |
| Adding Van Der Lugt, 1980 (k=30) | 0.01 | [0.01; 0.01] | . | 0.0016 | 0.0395 | 65% |
| Adding Van Der Lugt, 1980 (k=31) | 0.01 | [0.01; 0.01] | . | 0.0016 | 0.0400 | 66% |
| Adding Adler, 1981 (k=32) | 0.01 | [0.01; 0.01] | . | 0.0019 | 0.0437 | 70% |
| Adding Adler, 1981 (k=33) | 0.01 | [0.01; 0.01] | . | 0.0019 | 0.0437 | 71% |
| Adding Adler, 1981 (k=34) | 0.01 | [0.01; 0.01] | . | 0.0019 | 0.0430 | 71% |
| Adding Adler, 1981 (k=35) | 0.01 | [0.01; 0.01] | . | 0.0020 | 0.0442 | 71% |
| Adding Cevenini, 1982 (k=36) | 0.01 | [0.00; 0.01] | . | 0.0019 | 0.0439 | 70% |
| Adding Cevenini, 1982 (k=37) | 0.01 | [0.00; 0.01] | . | 0.0019 | 0.0436 | 70% |
| Adding Cevenini, 1982 (k=38) | 0.01 | [0.00; 0.01] | . | 0.0019 | 0.0432 | 69% |
| Adding Cevenini, 1982 (k=39) | 0.01 | [0.00; 0.01] | . | 0.0018 | 0.0429 | 68% |
| Adding Svensson, 1983 (k=40) | 0.01 | [0.00; 0.01] | . | 0.0019 | 0.0431 | 68% |
| Adding Svensson, 1983 (k=41) | 0.00 | [0.00; 0.01] | . | 0.0019 | 0.0430 | 67% |
| Adding Svensson, 1983 (k=42) | 0.00 | [0.00; 0.01] | . | 0.0019 | 0.0433 | 67% |
| Adding Southgate, 1983 (k=43) | 0.00 | [0.00; 0.01] | . | 0.0018 | 0.0427 | 67% |
| Adding Qvigstad, 1983 (k=44) | 0.00 | [0.00; 0.01] | . | 0.0017 | 0.0416 | 66% |
| Adding Anestad, 1984 (k=45) | 0.00 | [0.00; 0.01] | . | 0.0017 | 0.0413 | 65% |
| Adding Anestad, 1984 (k=46) | 0.00 | [0.00; 0.01] | . | 0.0017 | 0.0409 | 64% |
| Adding Buekens, 1984 (k=47) | 0.00 | [0.00; 0.01] | . | 0.0017 | 0.0409 | 64% |
| Adding Buekens, 1984 (k=48) | 0.00 | [0.00; 0.01] | . | 0.0016 | 0.0402 | 63% |
| Adding Avonts, 1985 (k=49) | 0.00 | [0.00; 0.01] | . | 0.0016 | 0.0395 | 63% |
| Adding Dewilde, 1986 (k=50) | 0.00 | [0.00; 0.01] | . | 0.0015 | 0.0388 | 62% |
| Adding Tuveng, 1986 (k=51) | 0.00 | [0.00; 0.01] | . | 0.0015 | 0.0387 | 61% |
| Adding Tuveng, 1986 (k=52) | 0.00 | [0.00; 0.01] | . | 0.0015 | 0.0385 | 60% |
| Adding Tjiam, 1987 (k=53) | 0.00 | [0.00; 0.01] | . | 0.0015 | 0.0391 | 61% |
| Adding Skjeldestad, 1987 (k=54) | 0.00 | [0.00; 0.01] | . | 0.0015 | 0.0388 | 61% |
| Adding Giertz, 1987 (k=55) | 0.00 | [0.00; 0.01] | . | 0.0015 | 0.0384 | 61% |
| Adding Duthie, 1987 (k=56) | 0.00 | [0.00; 0.01] | . | 0.0015 | 0.0384 | 61% |
| Adding Pearson, 1988 (k=57) | 0.00 | [0.00; 0.01] | . | 0.0015 | 0.0383 | 60% |
| Adding Bryman, 1988 (k=58) | 0.00 | [0.00; 0.01] | . | 0.0015 | 0.0382 | 60% |
| Adding Bryman, 1988 (k=59) | 0.00 | [0.00; 0.00] | . | 0.0014 | 0.0380 | 59% |
| Adding Diquelou, 1988 (k=60) | 0.00 | [0.00; 0.00] | . | 0.0014 | 0.0378 | 58% |
| Adding Avonts, 1989 (k=61) | 0.00 | [0.00; 0.00] | . | 0.0014 | 0.0373 | 58% |
| Adding Bro, 1989 (k=62) | 0.00 | [0.00; 0.00] | . | 0.0014 | 0.0368 | 57% |
| Adding Knudsen, 1989 (k=63) | 0.00 | [0.00; 0.00] | . | 0.0013 | 0.0362 | 57% |
| Adding Prien-Larsen, 1989 (k=64) | 0.00 | [0.00; 0.00] | . | 0.0013 | 0.0356 | 56% |
| Adding Sprague, 1990 (k=65) | 0.00 | [0.00; 0.00] | . | 0.0013 | 0.0359 | 56% |
| Adding Sprague, 1990 (k=66) | 0.00 | [0.00; 0.00] | . | 0.0013 | 0.0364 | 57% |
| Adding Riordan, 1990 (k=67) | 0.00 | [0.00; 0.00] | . | 0.0013 | 0.0361 | 57% |
| Adding Thewessen, 1990 (k=68) | 0.00 | [0.00; 0.00] | . | 0.0013 | 0.0357 | 56% |
| Adding Stray-Pedersen, 1991 (k=69) | 0.00 | [0.00; 0.00] | . | 0.0012 | 0.0349 | 56% |
| Adding Smith, 1991 (k=70) | 0.00 | [0.00; 0.00] | . | 0.0012 | 0.0345 | 55% |
| Adding Sorensen, 1992 (k=71) | 0.00 | [0.00; 0.00] | . | 0.0011 | 0.0339 | 54% |
| Adding Tekgul, 1992 (k=72) | 0.00 | [0.00; 0.00] | . | 0.0011 | 0.0338 | 54% |
| Adding Halvorsen, 1992 (k=73) | 0.00 | [0.00; 0.00] | . | 0.0012 | 0.0350 | 56% |
| Adding Skjeldestad, 1992 (k=74) | 0.00 | [0.00; 0.00] | . | 0.0012 | 0.0345 | 56% |

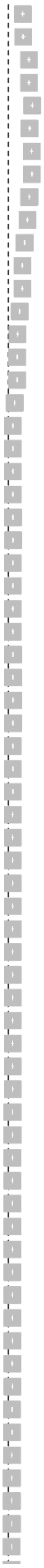

| | | | | | | |
|---|---|---|---|---|---|---|
| Adding Falk, 1993 (k=75) | 0.02 | [0.02; 0.02] | . | 0.0017 | 0.0417 | 93% |
| Adding Falk, 1993 (k=76) | 0.02 | [0.02; 0.02] | . | 0.0018 | 0.0425 | 93% |
| Adding Falk, 1993 (k=77) | 0.02 | [0.02; 0.03] | . | 0.0021 | 0.0453 | 93% |
| Adding Falk, 1993 (k=78) | 0.02 | [0.02; 0.02] | . | 0.0021 | 0.0460 | 93% |
| Adding Falk, 1993 (k=79) | 0.03 | [0.03; 0.03] | . | 0.0023 | 0.0482 | 94% |
| Adding Falk, 1993 (k=80) | 0.02 | [0.02; 0.03] | . | 0.0023 | 0.0478 | 94% |
| Adding Falk, 1993 (k=81) | 0.03 | [0.03; 0.03] | . | 0.0024 | 0.0494 | 95% |
| Adding Falk, 1993 (k=82) | 0.03 | [0.03; 0.03] | . | 0.0025 | 0.0499 | 94% |
| Adding Falk, 1993 (k=83) | 0.02 | [0.02; 0.02] | . | 0.0024 | 0.0495 | 95% |
| Adding Falk, 1993 (k=84) | 0.02 | [0.02; 0.02] | . | 0.0024 | 0.0490 | 96% |
| Adding Falk, 1993 (k=85) | 0.02 | [0.02; 0.02] | . | 0.0024 | 0.0485 | 96% |
| Adding Falk, 1993 (k=86) | 0.02 | [0.02; 0.02] | . | 0.0023 | 0.0483 | 97% |
| Adding Shafer, 1993 (k=87) | 0.02 | [0.02; 0.02] | . | 0.0026 | 0.0511 | 97% |
| Adding Falk, 1993 (k=88) | 0.02 | [0.02; 0.02] | . | 0.0026 | 0.0508 | 97% |
| Adding Falk, 1993 (k=89) | 0.02 | [0.02; 0.02] | . | 0.0026 | 0.0509 | 98% |
| Adding Geuenich, 1993 (k=90) | 0.02 | [0.01; 0.02] | . | 0.0026 | 0.0512 | 98% |
| Adding Blackwell, 1993 (k=91) | 0.02 | [0.01; 0.02] | . | 0.0026 | 0.0511 | 98% |
| Adding Falk, 1993 (k=92) | 0.01 | [0.01; 0.01] | . | 0.0026 | 0.0511 | 98% |
| Adding Falk, 1993 (k=93) | 0.01 | [0.01; 0.01] | . | 0.0026 | 0.0514 | 98% |
| Adding Genc, 1993 (k=94) | 0.01 | [0.01; 0.01] | . | 0.0027 | 0.0515 | 98% |
| Adding Henriques, 1994 (k=95) | 0.01 | [0.01; 0.01] | . | 0.0026 | 0.0511 | 98% |
| Adding Arvidson, 1995 (k=96) | 0.01 | [0.01; 0.01] | . | 0.0030 | 0.0547 | 98% |
| Adding Arvidson, 1995 (k=97) | 0.01 | [0.01; 0.01] | . | 0.0030 | 0.0550 | 98% |
| Adding Arvidson, 1995 (k=98) | 0.01 | [0.01; 0.01] | . | 0.0031 | 0.0558 | 98% |
| Adding Bassiri, 1997 (k=99) | 0.01 | [0.01; 0.01] | . | 0.0031 | 0.0557 | 98% |
| Adding Penney, 1998 (k=100) | 0.01 | [0.01; 0.01] | . | 0.0031 | 0.0555 | 98% |
| Adding Penney, 1998 (k=101) | 0.01 | [0.01; 0.01] | . | 0.0031 | 0.0556 | 98% |
| Adding Hellberg, 1999 (k=102) | 0.01 | [0.01; 0.01] | . | 0.0037 | 0.0605 | 98% |
| Adding Hellberg, 1999 (k=103) | 0.01 | [0.01; 0.01] | . | 0.0036 | 0.0603 | 98% |
| Adding Jamalova, 1999 (k=104) | 0.01 | [0.01; 0.01] | . | 0.0036 | 0.0601 | 98% |
| Adding Hodoglugil, 1999 (k=105) | 0.01 | [0.01; 0.01] | . | 0.0037 | 0.0608 | 98% |
| Adding Gillespie, 2000 (k=106) | 0.01 | [0.01; 0.01] | . | 0.0037 | 0.0609 | 98% |
| Adding Gillespie, 2000 (k=107) | 0.01 | [0.01; 0.01] | . | 0.0037 | 0.0610 | 98% |
| Adding Hodoglugil, 2000 (k=108) | 0.01 | [0.01; 0.01] | . | 0.0039 | 0.0622 | 98% |
| Adding Hodoglugil, 2000 (k=109) | 0.01 | [0.01; 0.01] | . | 0.0039 | 0.0621 | 98% |
| Adding Gillespie, 2000 (k=110) | 0.01 | [0.01; 0.01] | . | 0.0039 | 0.0627 | 98% |
| Adding Ostergaard, 2000 (k=111) | 0.01 | [0.01; 0.01] | . | 0.0040 | 0.0629 | 98% |
| Adding Ostergaard, 2000 (k=112) | 0.01 | [0.01; 0.01] | . | 0.0040 | 0.0631 | 98% |
| Adding Kacena, 2001 (k=113) | 0.01 | [0.01; 0.01] | . | 0.0040 | 0.0630 | 98% |
| Adding Kacena, 2001 (k=114) | 0.01 | [0.01; 0.01] | . | 0.0040 | 0.0630 | 98% |
| Adding Wilson, 2001 (k=115) | 0.01 | [0.01; 0.01] | . | 0.0040 | 0.0629 | 98% |
| Adding Rodriguez, 2001 (k=116) | 0.01 | [0.01; 0.01] | . | 0.0039 | 0.0627 | 98% |
| Adding Ortayli, 2001 (k=117) | 0.01 | [0.01; 0.01] | . | 0.0040 | 0.0630 | 98% |
| Adding Claeys, 2001 (k=118) | 0.01 | [0.01; 0.01] | . | 0.0040 | 0.0630 | 98% |
| Adding Harindra, 2002 (k=119) | 0.01 | [0.01; 0.01] | . | 0.0039 | 0.0628 | 98% |
| Adding Harindra, 2002 (k=120) | 0.01 | [0.01; 0.01] | . | 0.0039 | 0.0625 | 98% |
| Adding Dan, 2003 (k=121) | 0.01 | [0.01; 0.01] | . | 0.0039 | 0.0623 | 98% |
| Adding Low, 2003 (k=122) | 0.01 | [0.01; 0.01] | . | 0.0039 | 0.0622 | 98% |
| Adding Low, 2003 (k=123) | 0.01 | [0.01; 0.01] | . | 0.0038 | 0.0620 | 98% |
| Adding Acik, 2004 (k=124) | 0.01 | [0.01; 0.01] | . | 0.0038 | 0.0619 | 98% |
| Adding Dolapci, 2005 (k=125) | 0.01 | [0.01; 0.01] | . | 0.0038 | 0.0620 | 98% |
| Adding Van Bergen, 2006a (k=126) | 0.01 | [0.01; 0.01] | . | 0.0039 | 0.0622 | 98% |
| Adding Gotz, 2006 (k=127) | 0.01 | [0.01; 0.01] | . | 0.0038 | 0.0620 | 98% |
| Adding Gotz, 2006 (k=128) | 0.01 | [0.01; 0.01] | . | 0.0038 | 0.0618 | 98% |
| Adding Franceschi, 2007 (k=129) | 0.01 | [0.01; 0.01] | . | 0.0038 | 0.0617 | 98% |
| Adding Franceschi, 2007 (k=130) | 0.01 | [0.01; 0.01] | . | 0.0038 | 0.0618 | 98% |
| Adding Kourbatova, 2008 (k=131) | 0.01 | [0.01; 0.01] | . | 0.0039 | 0.0621 | 98% |
| Adding Kourbatova, 2008 (k=132) | 0.01 | [0.01; 0.01] | . | 0.0038 | 0.0619 | 98% |
| Adding Kourbatova, 2008 (k=133) | 0.01 | [0.01; 0.01] | . | 0.0038 | 0.0619 | 98% |
| Adding Schabereiter-Gurtner, 2008 (k=134) | 0.01 | [0.01; 0.01] | . | 0.0038 | 0.0615 | 98% |
| Adding Barthell, 2009 (k=135) | 0.01 | [0.01; 0.01] | . | 0.0038 | 0.0613 | 98% |
| Adding Barthell, 2009 (k=136) | 0.01 | [0.01; 0.01] | . | 0.0037 | 0.0612 | 98% |
| Adding Oakeshott, 2010 (k=137) | 0.01 | [0.01; 0.01] | . | 0.0037 | 0.0610 | 98% |
| Adding Detels, 2011 (k=138) | 0.01 | [0.01; 0.01] | . | 0.0037 | 0.0609 | 98% |
| Adding Frolund, 2011 (k=139) | 0.01 | [0.01; 0.01] | . | 0.0037 | 0.0608 | 98% |
| Adding Mahto, 2011 (k=140) | 0.01 | [0.01; 0.01] | . | 0.0037 | 0.0609 | 98% |
| Adding Eksi, 2011 (k=141) | 0.01 | [0.01; 0.01] | . | 0.0037 | 0.0607 | 98% |
| Adding Eksi, 2011 (k=142) | 0.01 | [0.01; 0.01] | . | 0.0037 | 0.0606 | 98% |
| [illegible] | [illegible] | [illegible] | . | [illegible] | [illegible] | [illegible] |

| | | | | | | |
|---|---|---|---|---|---|---|
| Adding Marcone, 2012 (k=143) | 0.01 | [0.01; 0.01] | . | 0.0037 | 0.0608 | 98% |
| Adding Borges-Costa, 2012 (k=144) | 0.01 | [0.01; 0.01] | . | 0.0038 | 0.0615 | 98% |
| Adding Skovgaard, 2012 (k=145) | 0.01 | [0.01; 0.01] | . | 0.0038 | 0.0615 | 98% |
| Adding Skovgaard, 2012 (k=146) | 0.01 | [0.01; 0.01] | . | 0.0037 | 0.0611 | 98% |
| Adding Gesink, 2012 (k=147) | 0.01 | [0.01; 0.01] | . | 0.0037 | 0.0610 | 98% |
| Adding Surel, 2012 (k=148) | 0.01 | [0.01; 0.01] | . | 0.0037 | 0.0607 | 98% |
| Adding Surel, 2012 (k=149) | 0.01 | [0.01; 0.01] | . | 0.0037 | 0.0607 | 98% |
| Adding Pagani, 2012 (k=150) | 0.01 | [0.01; 0.01] | . | 0.0037 | 0.0605 | 98% |
| Adding Papanagiotou, 2012 (k=151) | 0.01 | [0.01; 0.01] | . | 0.0037 | 0.0608 | 98% |
| Adding Papanagiotou, 2012 (k=152) | 0.01 | [0.01; 0.01] | . | 0.0037 | 0.0611 | 98% |
| Adding Dhairyawan, 2012 (k=153) | 0.01 | [0.01; 0.01] | . | 0.0037 | 0.0612 | 98% |
| Adding Rimoldi, 2012 (k=154) | 0.01 | [0.01; 0.01] | . | 0.0037 | 0.0611 | 98% |
| Adding Dautigny, 2013 (k=155) | 0.01 | [0.01; 0.01] | . | 0.0038 | 0.0613 | 98% |
| Adding Dautigny, 2013 (k=156) | 0.01 | [0.01; 0.01] | . | 0.0037 | 0.0610 | 98% |
| Adding Tomusiak, 2013 (k=157) | 0.01 | [0.01; 0.01] | . | 0.0037 | 0.0609 | 98% |
| Adding Frolova, 2013 (k=158) | 0.01 | [0.01; 0.01] | . | 0.0037 | 0.0606 | 98% |
| Adding Fowler, 2013 (k=159) | 0.01 | [0.01; 0.01] | . | 0.0037 | 0.0605 | 98% |
| Adding Fowler, 2013 (k=160) | 0.01 | [0.01; 0.01] | . | 0.0036 | 0.0602 | 98% |
| Adding Bayette, 2013 (k=161) | 0.01 | [0.01; 0.01] | . | 0.0038 | 0.0619 | 98% |
| Adding Bayette, 2013 (k=162) | 0.01 | [0.01; 0.01] | . | 0.0038 | 0.0616 | 98% |
| Adding Geelen, 2013 (k=163) | 0.01 | [0.01; 0.01] | . | 0.0038 | 0.0614 | 98% |
| Adding Miron, 2013 (k=164) | 0.01 | [0.01; 0.01] | . | 0.0038 | 0.0613 | 98% |
| Adding Shipitsyna, 2013 (k=165) | 0.01 | [0.01; 0.01] | . | 0.0037 | 0.0611 | 98% |
| Adding Shipitsyna, 2013 (k=166) | 0.01 | [0.01; 0.01] | . | 0.0037 | 0.0610 | 98% |
| Adding Sonnenberg, 2013 (k=167) | 0.01 | [0.01; 0.01] | . | 0.0037 | 0.0609 | 98% |
| Adding Sonnenberg, 2013 (k=168) | 0.01 | [0.01; 0.01] | . | 0.0037 | 0.0608 | 98% |
| Adding Sonnenberg, 2013 (k=169) | 0.01 | [0.01; 0.01] | . | 0.0037 | 0.0607 | 98% |
| Adding Sonnenberg, 2013 (k=170) | 0.01 | [0.01; 0.01] | . | 0.0037 | 0.0609 | 98% |
| Adding Sonnenberg, 2013 (k=171) | 0.01 | [0.01; 0.01] | . | 0.0037 | 0.0609 | 98% |
| Adding Sonnenberg, 2013 (k=172) | 0.01 | [0.01; 0.01] | . | 0.0037 | 0.0608 | 98% |
| Adding Sonnenberg, 2013 (k=173) | 0.01 | [0.01; 0.01] | . | 0.0037 | 0.0607 | 98% |
| Adding Sonnenberg, 2013 (k=174) | 0.01 | [0.01; 0.01] | . | 0.0037 | 0.0606 | 98% |
| Adding Sonnenberg, 2013 (k=175) | 0.01 | [0.01; 0.01] | . | 0.0037 | 0.0606 | 98% |
| Adding Sonnenberg, 2013 (k=176) | 0.01 | [0.01; 0.01] | . | 0.0037 | 0.0606 | 98% |
| Adding Jaureguy, 2013 (k=177) | 0.01 | [0.01; 0.01] | . | 0.0037 | 0.0608 | 98% |
| Adding Bremer, 2013 (k=178) | 0.01 | [0.01; 0.01] | . | 0.0037 | 0.0606 | 98% |
| Adding Wesbonk, 2014 (k=179) | 0.01 | [0.01; 0.01] | . | 0.0036 | 0.0604 | 98% |
| Adding Plecko, 2014 (k=180) | 0.01 | [0.01; 0.01] | . | 0.0036 | 0.0603 | 98% |
| Adding Peuchant, 2015b (k=181) | 0.01 | [0.01; 0.01] | . | 0.0036 | 0.0604 | 98% |
| Adding Peuchant, 2015b (k=182) | 0.01 | [0.01; 0.01] | . | 0.0037 | 0.0605 | 98% |
| Adding Bercot, 2015 (k=183) | 0.01 | [0.01; 0.01] | . | 0.0038 | 0.0619 | 98% |
| Adding Bourgeois-Nicolaos, 2015 (k=184) | 0.01 | [0.01; 0.01] | . | 0.0039 | 0.0623 | 98% |
| Adding Bourgeois-Nicolaos, 2015 (k=185) | 0.01 | [0.01; 0.01] | . | 0.0039 | 0.0628 | 98% |
| Adding Bourgeois-Nicolaos, 2015 (k=186) | 0.01 | [0.01; 0.01] | . | 0.0039 | 0.0626 | 98% |
| Adding Peuchant, 2015a (k=187) | 0.01 | [0.01; 0.01] | . | 0.0039 | 0.0625 | 98% |
| Adding Peuchant, 2015a (k=188) | 0.01 | [0.01; 0.01] | . | 0.0039 | 0.0624 | 98% |
| Adding Ebel, 2015 (k=189) | 0.01 | [0.01; 0.01] | . | 0.0039 | 0.0621 | 98% |
| Adding Ebel, 2015 (k=190) | 0.01 | [0.01; 0.01] | . | 0.0039 | 0.0628 | 98% |
| Adding Hay, 2016 (k=191) | 0.01 | [0.01; 0.01] | . | 0.0039 | 0.0626 | 98% |
| Adding Matteelli, 2016 (k=192) | 0.01 | [0.01; 0.01] | . | 0.0039 | 0.0627 | 98% |
| Adding Matteelli, 2016 (k=193) | 0.01 | [0.01; 0.01] | . | 0.0039 | 0.0628 | 98% |
| Adding Camporiondo, 2016 (k=194) | 0.01 | [0.01; 0.01] | . | 0.0039 | 0.0628 | 98% |
| Adding Hassan, 2016 (k=195) | 0.01 | [0.01; 0.01] | . | 0.0039 | 0.0628 | 98% |
| Adding Frati, 2017 (k=196) | 0.01 | [0.01; 0.01] | . | 0.0039 | 0.0626 | 98% |
| Adding Sambri, 2017 (k=197) | 0.01 | [0.01; 0.01] | . | 0.0039 | 0.0625 | 98% |
| Adding Jenniskens, 2017 (k=198) | 0.01 | [0.01; 0.01] | . | 0.0039 | 0.0623 | 98% |
| Adding Pereyre, 2017 (k=199) | 0.01 | [0.01; 0.01] | . | 0.0039 | 0.0621 | 98% |
| Adding Pereyre, 2017 (k=200) | 0.01 | [0.01; 0.01] | . | 0.0039 | 0.0623 | 98% |
| Adding Pereyre, 2017 (k=201) | 0.01 | [0.01; 0.01] | . | 0.0039 | 0.0624 | 98% |
| Adding Pereyre, 2017 (k=202) | 0.01 | [0.01; 0.01] | . | 0.0039 | 0.0626 | 98% |
| Adding Pereyre, 2017 (k=203) | 0.01 | [0.01; 0.01] | . | 0.0039 | 0.0626 | 98% |
| Adding Pereyre, 2017 (k=204) | 0.01 | [0.01; 0.01] | . | 0.0039 | 0.0628 | 98% |
| Adding Pereyre, 2017 (k=205) | 0.01 | [0.01; 0.01] | . | 0.0040 | 0.0634 | 98% |
| Adding Pereyre, 2017 (k=206) | 0.01 | [0.01; 0.01] | . | 0.0040 | 0.0634 | 98% |
| Adding Pereyre, 2017 (k=207) | 0.01 | [0.01; 0.01] | . | 0.0040 | 0.0633 | 98% |
| Adding Pereyre, 2017 (k=208) | 0.01 | [0.01; 0.01] | . | 0.0040 | 0.0634 | 98% |
| Adding Pereyre, 2017 (k=209) | 0.01 | [0.01; 0.01] | . | 0.0041 | 0.0641 | 98% |
| Adding Pereyre, 2017 (k=210) | 0.01 | [0.01; 0.01] | . | 0.0041 | 0.0639 | 98% |
| Adding Pereyre, 2017 (k=211) | 0.01 | [0.01; 0.01] | . | 0.0041 | 0.0638 | 98% |
| Adding Pereyre, 2017 (k=212) | 0.01 | [0.01; 0.01] | . | 0.0040 | 0.0636 | 98% |
| Adding Pereyre, 2017 (k=213) | 0.01 | [0.01; 0.01] | . | 0.0040 | 0.0635 | 98% |
| Adding Pereyre, 2017 (k=214) | 0.01 | [0.01; 0.01] | . | 0.0040 | 0.0635 | 98% |
| Adding Del Prete, 2017 (k=215) | 0.01 | [0.01; 0.01] | . | 0.0040 | 0.0634 | 98% |
| Adding Del Prete, 2017 (k=216) | 0.01 | [0.01; 0.01] | . | 0.0041 | 0.0637 | 98% |

| | | | | | | |
|---|---|---|---|---|---|---|
| [illegible] | | | | | | |
| Adding Kanbur, 2018 (k=217) | 0.01 | [0.01; 0.01] | . | 0.0040 | 0.0635 | 98% |
| Adding Graspeuntner, 2018 (k=218) | 0.01 | [0.01; 0.01] | . | 0.0040 | 0.0634 | 98% |
| Adding Nicholls, 2018 (k=219) | 0.01 | [0.01; 0.01] | . | 0.0040 | 0.0632 | 98% |
| Adding Homoe, 2018 (k=220) | 0.01 | [0.01; 0.01] | . | 0.0045 | 0.0668 | 98% |
| Adding Homoe, 2018 (k=221) | 0.01 | [0.01; 0.01] | . | 0.0048 | 0.0693 | 98% |
| Adding Homoe, 2018 (k=222) | 0.01 | [0.01; 0.01] | . | 0.0054 | 0.0732 | 98% |
| Adding Homoe, 2018 (k=223) | 0.01 | [0.01; 0.01] | . | 0.0056 | 0.0745 | 98% |
| Adding Homoe, 2018 (k=224) | 0.01 | [0.01; 0.01] | . | 0.0059 | 0.0768 | 98% |
| Adding Homoe, 2018 (k=225) | 0.01 | [0.01; 0.01] | . | 0.0060 | 0.0776 | 98% |
| Adding Homoe, 2018 (k=226) | 0.01 | [0.01; 0.01] | . | 0.0063 | 0.0791 | 98% |
| Adding Homoe, 2018 (k=227) | 0.01 | [0.01; 0.01] | . | 0.0063 | 0.0794 | 98% |
| Adding Homoe, 2018 (k=228) | 0.01 | [0.01; 0.01] | . | 0.0065 | 0.0804 | 98% |
| Adding Homoe, 2018 (k=229) | 0.01 | [0.01; 0.01] | . | 0.0065 | 0.0806 | 98% |
| Adding Homoe, 2018 (k=230) | 0.01 | [0.01; 0.01] | . | 0.0066 | 0.0810 | 98% |
| Adding Homoe, 2018 (k=231) | 0.01 | [0.01; 0.01] | . | 0.0065 | 0.0809 | 98% |
| Adding Homoe, 2018 (k=232) | 0.01 | [0.01; 0.01] | . | 0.0067 | 0.0816 | 98% |
| Adding Homoe, 2018 (k=233) | 0.01 | [0.01; 0.01] | . | 0.0067 | 0.0816 | 98% |
| Adding Homoe, 2018 (k=234) | 0.01 | [0.01; 0.01] | . | 0.0067 | 0.0818 | 98% |
| Adding Homoe, 2018 (k=235) | 0.01 | [0.01; 0.01] | . | 0.0067 | 0.0817 | 98% |
| Adding Homoe, 2018 (k=236) | 0.01 | [0.01; 0.01] | . | 0.0067 | 0.0821 | 98% |
| Adding Homoe, 2018 (k=237) | 0.01 | [0.01; 0.01] | . | 0.0067 | 0.0821 | 98% |
| Adding Homoe, 2018 (k=238) | 0.01 | [0.01; 0.01] | . | 0.0068 | 0.0824 | 98% |
| Adding Homoe, 2018 (k=239) | 0.01 | [0.01; 0.01] | . | 0.0068 | 0.0825 | 98% |
| Adding Nolskog, 2019 (k=240) | 0.01 | [0.01; 0.01] | . | 0.0068 | 0.0825 | 98% |
| Adding Criado, 2019 (k=241) | 0.01 | [0.01; 0.01] | . | 0.0068 | 0.0824 | 98% |
| Adding Oakeshott, 2019 (k=242) | 0.01 | [0.01; 0.01] | . | 0.0068 | 0.0822 | 98% |
| Adding Oakeshott, 2019 (k=243) | 0.01 | [0.01; 0.01] | . | 0.0068 | 0.0822 | 98% |
| Adding Heijne, 2019 (k=244) | 0.01 | [0.01; 0.01] | . | 0.0068 | 0.0823 | 98% |
| Adding klavs, 2019 (k=245) | 0.01 | [0.01; 0.01] | . | 0.0068 | 0.0823 | 98% |
| Adding klavs, 2019 (k=246) | 0.01 | [0.01; 0.01] | . | 0.0068 | 0.0823 | 98% |
| Adding Tittle, 2019 (k=247) | 0.01 | [0.01; 0.01] | . | 0.0067 | 0.0821 | 98% |
| Adding Shipitsyna, 2020 (k=248) | 0.01 | [0.01; 0.01] | . | 0.0067 | 0.0820 | 98% |
| Adding Kerry-Barnard, 2020 (k=249) | 0.01 | [0.01; 0.01] | . | 0.0067 | 0.0820 | 98% |
| Adding Kerry-Barnard, 2020 (k=250) | 0.01 | [0.01; 0.01] | . | 0.0067 | 0.0819 | 98% |
| Adding Dao, 2020 (k=251) | 0.01 | [0.01; 0.01] | . | 0.0067 | 0.0818 | 98% |
| Adding Silva, 2021 (k=252) | 0.01 | [0.01; 0.01] | . | 0.0067 | 0.0816 | 98% |
| Adding Silva, 2021 (k=253) | 0.01 | [0.01; 0.01] | . | 0.0066 | 0.0815 | 98% |
| Adding Silva, 2021 (k=254) | 0.01 | [0.01; 0.01] | . | 0.0066 | 0.0813 | 98% |
| Adding Silva, 2021 (k=255) | 0.01 | [0.01; 0.01] | . | 0.0066 | 0.0812 | 98% |
| Adding Silva, 2021 (k=256) | 0.01 | [0.01; 0.01] | . | 0.0066 | 0.0812 | 98% |
| Adding Silva, 2021 (k=257) | 0.01 | [0.01; 0.01] | . | 0.0066 | 0.0811 | 98% |
| Adding Op de Coul, 2021 (k=258) | 0.01 | [0.01; 0.01] | . | 0.0066 | 0.0810 | 98% |
| Adding Op de Coul, 2021 (k=259) | 0.01 | [0.01; 0.01] | . | 0.0065 | 0.0809 | 98% |
| Adding Bartholomew, 2021 (k=260) | 0.01 | [0.01; 0.01] | . | 0.0065 | 0.0808 | 98% |
| Adding Bartholomew, 2021 (k=261) | 0.01 | [0.01; 0.01] | . | 0.0065 | 0.0807 | 98% |
| Adding Skaletz-Rorowski, 2021 (k=262) | 0.01 | [0.01; 0.01] | . | 0.0065 | 0.0805 | 98% |
| **Common effect model** | **0.01** | **[0.01; 0.01]** | **.** | **0.0065** | **0.0805** | **98%** |

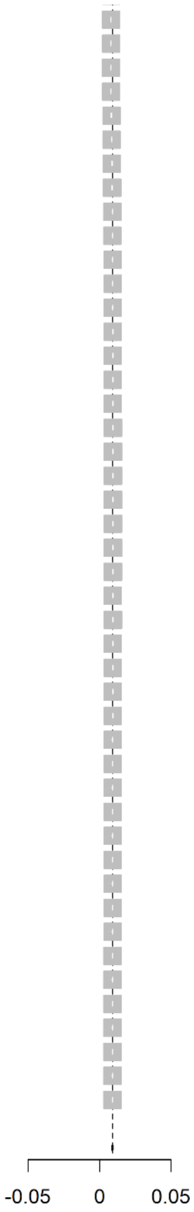


[a] For the cumulative meta-analysis of urogenital measures, only measures from general populations were included due to technical limitations. Attempting to include the very large urogenital dataset for all population types encountered code-related challenges in conducting this analysis.

## B. Anorectal

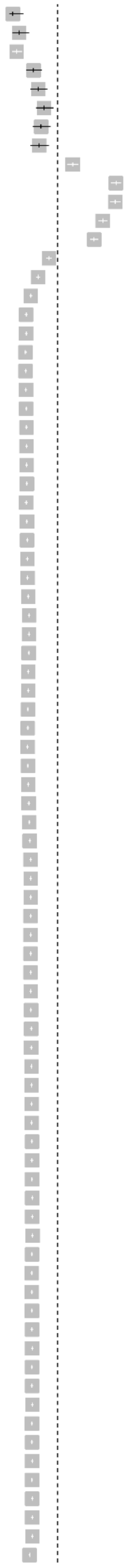

| Study | Proportion | 95%-CI | P-value | Tau2 | Tau | I2 |
|---|---|---|---|---|---|---|
| Adding Palous, 1971 (k=1) | 0.00 | [0.00; 0.01] | . | . | . | . |
| Adding Palous, 1971 (k=2) | 0.01 | [0.00; 0.02] | . | 0.0017 | 0.0407 | 72% |
| Adding Gregersen, 1972 (k=3) | 0.01 | [0.00; 0.01] | . | 0.0007 | 0.0269 | 58% |
| Adding Robinson, 1974 (k=4) | 0.02 | [0.01; 0.03] | . | 0.0190 | 0.1378 | 97% |
| Adding Shahidullah, 1976 (k=5) | 0.02 | [0.02; 0.03] | . | 0.0295 | 0.1717 | 97% |
| Adding Shahidullah, 1976 (k=6) | 0.03 | [0.02; 0.04] | . | 0.0361 | 0.1900 | 97% |
| Adding Shahidullah, 1976 (k=7) | 0.03 | [0.02; 0.03] | . | 0.0377 | 0.1943 | 97% |
| Adding Shahidullah, 1976 (k=8) | 0.02 | [0.02; 0.03] | . | 0.0358 | 0.1892 | 97% |
| Adding Hellgren, 1982 (k=9) | 0.05 | [0.05; 0.06] | . | 0.0306 | 0.1750 | 97% |
| Adding Hellgren, 1982 (k=10) | 0.09 | [0.08; 0.09] | . | 0.0280 | 0.1674 | 99% |
| Adding Bovet, 1982 (k=11) | 0.09 | [0.08; 0.09] | . | 0.0251 | 0.1584 | 99% |
| Adding Gonzalez, 1983 (k=12) | 0.08 | [0.07; 0.08] | . | 0.0296 | 0.1720 | 99% |
| Adding Gonzalez, 1983 (k=13) | 0.07 | [0.07; 0.07] | . | 0.0304 | 0.1744 | 99% |
| Adding Gellan, 1986 (k=14) | 0.03 | [0.03; 0.03] | . | 0.0296 | 0.1720 | 100% |
| Adding Gellan, 1986 (k=15) | 0.02 | [0.02; 0.02] | . | 0.0283 | 0.1683 | 100% |
| Adding Gellan, 1986 (k=16) | 0.02 | [0.02; 0.02] | . | 0.0275 | 0.1658 | 100% |
| Adding Gellan, 1986 (k=17) | 0.01 | [0.01; 0.01] | . | 0.0269 | 0.1640 | 100% |
| Adding Christophersen, 1988 (k=18) | 0.01 | [0.01; 0.01] | . | 0.0257 | 0.1604 | 100% |
| Adding Christophersen, 1988 (k=19) | 0.01 | [0.01; 0.01] | . | 0.0248 | 0.1576 | 100% |
| Adding Stary, 1991 (k=20) | 0.01 | [0.01; 0.01] | . | 0.0239 | 0.1547 | 100% |
| Adding Ross, 1991a (k=21) | 0.01 | [0.01; 0.01] | . | 0.0225 | 0.1500 | 100% |
| Adding Ross, 1991a (k=22) | 0.01 | [0.01; 0.01] | . | 0.0212 | 0.1456 | 99% |
| Adding Ross, 1991a (k=23) | 0.01 | [0.01; 0.01] | . | 0.0202 | 0.1421 | 99% |
| Adding Ross, 1991a (k=24) | 0.01 | [0.01; 0.01] | . | 0.0192 | 0.1387 | 99% |
| Adding Ross, 1991a (k=25) | 0.01 | [0.01; 0.01] | . | 0.0187 | 0.1367 | 99% |
| Adding Ross, 1991a (k=26) | 0.01 | [0.01; 0.01] | . | 0.0180 | 0.1343 | 99% |
| Adding Tomlinson, 1991 (k=27) | 0.01 | [0.01; 0.01] | . | 0.0179 | 0.1340 | 99% |
| Adding De Wit, 1993 (k=28) | 0.01 | [0.01; 0.01] | . | 0.0171 | 0.1308 | 99% |
| Adding De Wit, 1993 (k=29) | 0.01 | [0.01; 0.02] | . | 0.0164 | 0.1280 | 99% |
| Adding De Wit, 1993 (k=30) | 0.01 | [0.01; 0.02] | . | 0.0157 | 0.1254 | 99% |
| Adding De Wit, 1993 (k=31) | 0.01 | [0.01; 0.02] | . | 0.0151 | 0.1228 | 99% |
| Adding De Wit, 1993 (k=32) | 0.02 | [0.01; 0.02] | . | 0.0145 | 0.1205 | 99% |
| Adding De Wit, 1993 (k=33) | 0.02 | [0.02; 0.02] | . | 0.0143 | 0.1198 | 99% |
| Adding Vlaspolder, 1993 (k=34) | 0.02 | [0.01; 0.02] | . | 0.0140 | 0.1182 | 99% |
| Adding Vlaspolder, 1993 (k=35) | 0.02 | [0.01; 0.02] | . | 0.0136 | 0.1167 | 99% |
| Adding Lister, 1993 (k=36) | 0.02 | [0.01; 0.02] | . | 0.0134 | 0.1157 | 99% |
| Adding Van Der Snoek, 2000 (k=37) | 0.02 | [0.01; 0.02] | . | 0.0131 | 0.1145 | 99% |
| Adding Van Der Snoek, 2000 (k=38) | 0.01 | [0.01; 0.02] | . | 0.0133 | 0.1155 | 99% |
| Adding Van Der Snoek, 2000 (k=39) | 0.01 | [0.01; 0.02] | . | 0.0133 | 0.1154 | 99% |
| Adding Van Der Snoek, 2000 (k=40) | 0.01 | [0.01; 0.02] | . | 0.0132 | 0.1149 | 99% |
| Adding Stolte, 2001 (k=41) | 0.01 | [0.01; 0.02] | . | 0.0128 | 0.1131 | 99% |
| Adding Stolte, 2001 (k=42) | 0.02 | [0.01; 0.02] | . | 0.0124 | 0.1113 | 99% |
| Adding Stolte, 2001 (k=43) | 0.02 | [0.01; 0.02] | . | 0.0120 | 0.1098 | 99% |
| Adding Stolte, 2001 (k=44) | 0.02 | [0.02; 0.02] | . | 0.0117 | 0.1084 | 99% |
| Adding Stolte, 2001 (k=45) | 0.02 | [0.02; 0.02] | . | 0.0114 | 0.1068 | 99% |
| Adding Stolte, 2001 (k=46) | 0.02 | [0.02; 0.02] | . | 0.0112 | 0.1060 | 99% |
| Adding Van der Snoek, 2004 (k=47) | 0.02 | [0.02; 0.02] | . | 0.0110 | 0.1047 | 99% |
| Adding Van der Snoek, 2004 (k=48) | 0.02 | [0.02; 0.02] | . | 0.0107 | 0.1033 | 99% |
| Adding Manavi, 2004 (k=49) | 0.02 | [0.02; 0.02] | . | 0.0105 | 0.1025 | 99% |
| Adding Manavi, 2004 (k=50) | 0.02 | [0.02; 0.02] | . | 0.0102 | 0.1012 | 99% |
| Adding Manavi, 2005 (k=51) | 0.02 | [0.02; 0.02] | . | 0.0106 | 0.1029 | 99% |
| Adding Papadogeorgaki, 2006 (k=52) | 0.02 | [0.02; 0.02] | . | 0.0109 | 0.1043 | 99% |
| Adding Stolte, 2006 (k=53) | 0.02 | [0.02; 0.02] | . | 0.0117 | 0.1081 | 99% |
| Adding Stolte, 2006 (k=54) | 0.02 | [0.02; 0.02] | . | 0.0115 | 0.1070 | 99% |
| Adding Stolte, 2006 (k=55) | 0.02 | [0.02; 0.02] | . | 0.0113 | 0.1064 | 99% |
| Adding Unemo, 2006 (k=56) | 0.02 | [0.02; 0.02] | . | 0.0112 | 0.1060 | 99% |
| Adding Heijman, 2007 (k=57) | 0.02 | [0.02; 0.02] | . | 0.0110 | 0.1049 | 99% |
| Adding Benn, 2007 (k=58) | 0.02 | [0.02; 0.02] | . | 0.0112 | 0.1060 | 99% |
| Adding Benn, 2007 (k=59) | 0.02 | [0.02; 0.02] | . | 0.0111 | 0.1055 | 99% |
| Adding Benn, 2007 (k=60) | 0.02 | [0.02; 0.02] | . | 0.0109 | 0.1046 | 99% |
| Adding Ivens, 2007 (k=61) | 0.02 | [0.02; 0.02] | . | 0.0107 | 0.1035 | 99% |
| Adding Soni, 2008 (k=62) | 0.02 | [0.02; 0.02] | . | 0.0105 | 0.1026 | 99% |
| Adding Soni, 2008 (k=63) | 0.02 | [0.02; 0.02] | . | 0.0106 | 0.1028 | 99% |
| Adding Bozicevic, 2009 (k=64) | 0.02 | [0.02; 0.02] | . | 0.0109 | 0.1045 | 99% |
| Adding Shipitsyna, 2009 (k=65) | 0.02 | [0.02; 0.02] | . | 0.0111 | 0.1055 | 99% |
| Adding Annan, 2009 (k=66) | 0.02 | [0.02; 0.02] | . | 0.0109 | 0.1045 | 99% |
| Adding Kamarashev, 2010 (k=67) | 0.02 | [0.02; 0.02] | . | 0.0110 | 0.1050 | 99% |
| Adding Sethupathi, 2010 (k=68) | 0.02 | [0.02; 0.02] | . | 0.0117 | 0.1083 | 99% |
| Adding Soni, 2010 (k=69) | 0.02 | [0.02; 0.02] | . | 0.0115 | 0.1073 | 99% |
| Adding Walsh, 2011b (k=70) | 0.02 | [0.02; 0.02] | . | 0.0113 | 0.1065 | 99% |
| Adding Walsh, 2011b (k=71) | 0.02 | [0.02; 0.02] | . | 0.0111 | 0.1056 | 99% |
| Adding Heiligenberg, 2011b (k=72) | 0.02 | [0.02; 0.02] | . | 0.0109 | 0.1046 | 99% |
| Adding Heiligenberg, 2011b (k=73) | 0.02 | [0.02; 0.02] | . | 0.0111 | 0.1055 | 99% |
| Adding Peters, 2011 (k=74) | 0.02 | [0.02; 0.02] | . | 0.0110 | 0.1049 | 99% |
| Adding Peters, 2011 (k=75) | 0.02 | [0.02; 0.02] | . | 0.0108 | 0.1041 | 99% |
| Adding Heras, 2011 (k=76) | 0.02 | [0.02; 0.02] | . | 0.0109 | 0.1044 | 99% |
| Adding McCarty, 2011 (k=77) | 0.02 | [0.02; 0.02] | . | 0.0107 | 0.1035 | 99% |
| Adding Soni, 2011 (k=78) | 0.02 | [0.02; 0.02] | . | 0.0105 | 0.1026 | 99% |
| Adding Skovgaard, 2012 (k=79) | 0.02 | [0.02; 0.02] | . | 0.0106 | 0.1028 | 99% |
| Adding Skovgaard, 2012 (k=80) | 0.02 | [0.02; 0.02] | . | 0.0104 | 0.1019 | 99% |
| Adding Heiligenberg, 2012 (k=81) | 0.02 | [0.02; 0.02] | . | 0.0102 | 0.1011 | 98% |
| Adding Heiligenberg, 2012 (k=82) | 0.02 | [0.02; 0.02] | . | 0.0101 | 0.1003 | 98% |
| Adding Koedijk, 2012 (k=83) | 0.02 | [0.02; 0.02] | . | 0.0100 | 0.1001 | 99% |

| | | | | | | |
|---|---|---|---|---|---|---|
| [illegible] | [illegible] | [illegible] | [illegible] | [illegible] | [illegible] | [illegible] |
| Adding Koedijk, 2012 (k=84) | 0.02 | [0.02; 0.02] | . | 0.0099 | 0.0994 | 99% |
| Adding Geelen, 2013 (k=85) | 0.02 | [0.02; 0.02] | . | 0.0098 | 0.0988 | 99% |
| Adding Vriend, 2013 (k=86) | 0.02 | [0.02; 0.02] | . | 0.0096 | 0.0982 | 99% |
| Adding Vriend, 2013 (k=87) | 0.02 | [0.02; 0.02] | . | 0.0096 | 0.0981 | 99% |
| Adding Reinton, 2013 (k=88) | 0.02 | [0.02; 0.02] | . | 0.0095 | 0.0974 | 99% |
| Adding Jeverica, 2013 (k=89) | 0.02 | [0.02; 0.02] | . | 0.0095 | 0.0974 | 99% |
| Adding Roberts, 2013 (k=90) | 0.02 | [0.02; 0.02] | . | 0.0094 | 0.0967 | 99% |
| Adding Dudareva-Vizule, 2014 (k=91) | 0.02 | [0.02; 0.02] | . | 0.0092 | 0.0960 | 99% |
| Adding Keaveney, 2014 (k=92) | 0.02 | [0.02; 0.02] | . | 0.0092 | 0.0959 | 99% |
| Adding Keaveney, 2014 (k=93) | 0.02 | [0.02; 0.02] | . | 0.0091 | 0.0955 | 99% |
| Adding De Vrieze, 2014 (k=94) | 0.02 | [0.02; 0.02] | . | 0.0090 | 0.0951 | 99% |
| Adding De Vrieze, 2014 (k=95) | 0.02 | [0.02; 0.02] | . | 0.0089 | 0.0944 | 99% |
| Adding Vahidnia, 2014 (k=96) | 0.02 | [0.02; 0.02] | . | 0.0089 | 0.0942 | 99% |
| Adding Vahidnia, 2014 (k=97) | 0.02 | [0.02; 0.02] | . | 0.0088 | 0.0940 | 99% |
| Adding Vahidnia, 2014 (k=98) | 0.02 | [0.02; 0.02] | . | 0.0088 | 0.0938 | 99% |
| Adding Van Liere, 2014a (k=99) | 0.02 | [0.02; 0.02] | . | 0.0087 | 0.0931 | 99% |
| Adding Van Liere, 2014a (k=100) | 0.02 | [0.02; 0.02] | . | 0.0087 | 0.0932 | 99% |
| Adding Van Liere, 2014b (k=101) | 0.02 | [0.02; 0.02] | . | 0.0090 | 0.0947 | 99% |
| Adding Hagemann, 2014 (k=102) | 0.02 | [0.02; 0.02] | . | 0.0090 | 0.0947 | 99% |
| Adding Perez-Hernandez, 2014 (k=103) | 0.02 | [0.02; 0.02] | . | 0.0089 | 0.0941 | 99% |
| Adding Rodríguez-Domínguez, 2014 (k=104) | 0.02 | [0.02; 0.02] | . | 0.0092 | 0.0957 | 99% |
| Adding Sprenger, 2014 (k=105) | 0.02 | [0.02; 0.02] | . | 0.0091 | 0.0952 | 99% |
| Adding Sprenger, 2014 (k=106) | 0.02 | [0.02; 0.02] | . | 0.0091 | 0.0954 | 99% |
| Adding Sprenger, 2014 (k=107) | 0.02 | [0.02; 0.02] | . | 0.0091 | 0.0953 | 99% |
| Adding Cresswell, 2014 (k=108) | 0.02 | [0.02; 0.02] | . | 0.0090 | 0.0951 | 99% |
| Adding Dolling, 2014 (k=109) | 0.02 | [0.02; 0.02] | . | 0.0089 | 0.0945 | 99% |
| Adding Fraser, 2014 (k=110) | 0.02 | [0.02; 0.02] | . | 0.0089 | 0.0942 | 98% |
| Adding Ward, 2014 (k=111) | 0.02 | [0.02; 0.02] | . | 0.0090 | 0.0947 | 98% |
| Adding Plaas, 2015 (k=112) | 0.02 | [0.02; 0.02] | . | 0.0089 | 0.0942 | 98% |
| Adding Marcus, 2015 (k=113) | 0.02 | [0.02; 0.02] | . | 0.0088 | 0.0936 | 98% |
| Adding Spauwen, 2015 (k=114) | 0.02 | [0.02; 0.02] | . | 0.0088 | 0.0939 | 98% |
| Adding Van Liere, 2015 (k=115) | 0.02 | [0.02; 0.02] | . | 0.0088 | 0.0939 | 98% |
| Adding Van Liere, 2015 (k=116) | 0.02 | [0.02; 0.02] | . | 0.0087 | 0.0934 | 98% |
| Adding Van Rooijen, 2015 (k=117) | 0.02 | [0.02; 0.02] | . | 0.0086 | 0.0928 | 98% |
| Adding Van Rooijen, 2015 (k=118) | 0.02 | [0.02; 0.02] | . | 0.0088 | 0.0936 | 98% |
| Adding Van Rooijen, 2015 (k=119) | 0.02 | [0.02; 0.02] | . | 0.0088 | 0.0938 | 98% |
| Adding Van Rooijen, 2015 (k=120) | 0.02 | [0.02; 0.02] | . | 0.0087 | 0.0933 | 98% |
| Adding Verscheijden, 2015 (k=121) | 0.02 | [0.02; 0.02] | . | 0.0086 | 0.0929 | 98% |
| Adding Verscheijden, 2015 (k=122) | 0.02 | [0.02; 0.02] | . | 0.0086 | 0.0927 | 98% |
| Adding Verscheijden, 2015 (k=123) | 0.02 | [0.02; 0.02] | . | 0.0086 | 0.0926 | 98% |
| Adding Verscheijden, 2015 (k=124) | 0.02 | [0.02; 0.02] | . | 0.0085 | 0.0924 | 98% |
| Adding Verscheijden, 2015 (k=125) | 0.02 | [0.02; 0.02] | . | 0.0085 | 0.0922 | 98% |
| Adding Verscheijden, 2015 (k=126) | 0.02 | [0.02; 0.02] | . | 0.0085 | 0.0922 | 98% |
| Adding Wind, 2015 (k=127) | 0.02 | [0.02; 0.02] | . | 0.0086 | 0.0926 | 98% |
| Adding Wind, 2015 (k=128) | 0.02 | [0.02; 0.02] | . | 0.0085 | 0.0924 | 98% |
| Adding Garner, 2015 (k=129) | 0.02 | [0.02; 0.02] | . | 0.0086 | 0.0926 | 98% |
| Adding Garner, 2015 (k=130) | 0.02 | [0.02; 0.02] | . | 0.0085 | 0.0921 | 98% |
| Adding Garner, 2015 (k=131) | 0.02 | [0.02; 0.02] | . | 0.0084 | 0.0919 | 98% |
| Adding Garner, 2015 (k=132) | 0.02 | [0.02; 0.02] | . | 0.0084 | 0.0918 | 98% |
| Adding Thorsteinsson, 2016 (k=133) | 0.02 | [0.02; 0.02] | . | 0.0086 | 0.0926 | 98% |
| Adding Jaureguy, 2016 (k=134) | 0.02 | [0.02; 0.02] | . | 0.0086 | 0.0928 | 98% |
| Adding Fuchs, 2016 (k=135) | 0.02 | [0.02; 0.02] | . | 0.0087 | 0.0930 | 98% |
| Adding Cabello Ubeda, 2016 (k=136) | 0.02 | [0.02; 0.02] | . | 0.0087 | 0.0932 | 98% |
| Adding Moreno-Ribera, 2016 (k=137) | 0.02 | [0.02; 0.02] | . | 0.0087 | 0.0931 | 98% |
| Adding Gardner, 2016 (k=138) | 0.02 | [0.02; 0.02] | . | 0.0087 | 0.0935 | 98% |
| Adding Gardner, 2016 (k=139) | 0.02 | [0.02; 0.02] | . | 0.0090 | 0.0951 | 98% |
| Adding Gardner, 2016 (k=140) | 0.02 | [0.02; 0.02] | . | 0.0097 | 0.0983 | 98% |
| Adding Sultan, 2016 (k=141) | 0.02 | [0.02; 0.02] | . | 0.0100 | 0.1000 | 98% |
| Adding Apers, 2017 (k=142) | 0.02 | [0.02; 0.02] | . | 0.0109 | 0.1042 | 98% |
| Adding Edouard, 2017a (k=143) | 0.02 | [0.02; 0.02] | . | 0.0108 | 0.1038 | 98% |
| Adding Edouard, 2017b (k=144) | 0.02 | [0.02; 0.02] | . | 0.0108 | 0.1039 | 98% |
| Adding Fata, 2017 (k=145) | 0.02 | [0.02; 0.02] | . | 0.0107 | 0.1035 | 98% |
| Adding Achterbergh, 2017 (k=146) | 0.02 | [0.02; 0.02] | . | 0.0106 | 0.1030 | 98% |
| Adding Den Heijer, 2017 (k=147) | 0.02 | [0.02; 0.02] | . | 0.0106 | 0.1028 | 98% |
| Adding Den Heijer, 2017 (k=148) | 0.02 | [0.02; 0.02] | . | 0.0105 | 0.1023 | 98% |
| Adding Cox, 2017 (k=149) | 0.02 | [0.02; 0.02] | . | 0.0104 | 0.1019 | 98% |
| Adding Davies, 2017 (k=150) | 0.02 | [0.02; 0.02] | . | 0.0107 | 0.1032 | 98% |
| Adding Lawrence, 2017 (k=151) | 0.02 | [0.02; 0.02] | . | 0.0108 | 0.1041 | 98% |
| Adding Marchant, 2017 (k=152) | 0.02 | [0.02; 0.02] | . | 0.0110 | 0.1050 | 98% |
| Adding Sacks, 2017 (k=153) | 0.02 | [0.02; 0.02] | . | 0.0118 | 0.1088 | 98% |
| Adding Lourtet Hascoet, 2018 (k=154) | 0.02 | [0.02; 0.02] | . | 0.0126 | 0.1122 | 98% |
| Adding Lourtet Hascoet, 2018 (k=155) | 0.02 | [0.02; 0.02] | . | 0.0131 | 0.1145 | 98% |
| Adding Spinner, 2018 (k=156) | 0.02 | [0.02; 0.02] | . | 0.0130 | 0.1141 | 98% |
| Adding Levy, 2018 (k=157) | 0.02 | [0.02; 0.02] | . | 0.0133 | 0.1151 | 98% |
| Adding Druckler, 2018 (k=158) | 0.02 | [0.02; 0.02] | . | 0.0132 | 0.1148 | 98% |
| Adding Matser, 2018 (k=159) | 0.02 | [0.02; 0.02] | . | 0.0131 | 0.1143 | 98% |

| | | | | | | |
|---|---|---|---|---|---|---|
| Adding Van Rooijen, 2018 (k=160) | 0.02 | [0.02; 0.02] | . | 0.0131 | 0.1143 | 98% |
| Adding Van Rooijen, 2018 (k=161) | 0.02 | [0.02; 0.02] | . | 0.0130 | 0.1140 | 98% |
| Adding Van Rooijen, 2018 (k=162) | 0.02 | [0.02; 0.02] | . | 0.0130 | 0.1139 | 98% |
| Adding Van Rooijen, 2018 (k=163) | 0.02 | [0.02; 0.03] | . | 0.0129 | 0.1135 | 99% |
| Adding Dona, 2018 (k=164) | 0.02 | [0.02; 0.03] | . | 0.0140 | 0.1185 | 99% |
| Adding Jeffery, 2018 (k=165) | 0.02 | [0.02; 0.03] | . | 0.0141 | 0.1188 | 99% |
| Adding Rahib, 2019 (k=166) | 0.02 | [0.02; 0.03] | . | 0.0140 | 0.1183 | 99% |
| Adding Storim, 2019 (k=167) | 0.02 | [0.02; 0.03] | . | 0.0140 | 0.1181 | 99% |
| Adding Ceccarani, 2019 (k=168) | 0.02 | [0.02; 0.03] | . | 0.0145 | 0.1206 | 99% |
| Adding Hoornenborg, 2019 (k=169) | 0.02 | [0.02; 0.03] | . | 0.0145 | 0.1205 | 99% |
| Adding Ribeiro, 2019 (k=170) | 0.02 | [0.02; 0.03] | . | 0.0144 | 0.1200 | 99% |
| Adding De Salazar, 2019 (k=171) | 0.02 | [0.02; 0.03] | . | 0.0143 | 0.1197 | 99% |
| Adding Fernandez-Huerta, 2019 (k=172) | 0.02 | [0.02; 0.03] | . | 0.0144 | 0.1200 | 99% |
| Adding Foronda-Garcia-Hidalgo, 2019 (k=173) | 0.02 | [0.02; 0.03] | . | 0.0145 | 0.1204 | 99% |
| Adding Cherkaoui, 2019 (k=174) | 0.02 | [0.02; 0.02] | . | 0.0146 | 0.1209 | 99% |
| Adding De Baetselier, 2020 (k=175) | 0.02 | [0.02; 0.02] | . | 0.0145 | 0.1205 | 99% |
| Adding Verougstraete, 2020 (k=176) | 0.02 | [0.02; 0.02] | . | 0.0145 | 0.1203 | 99% |
| Adding Peuchant, 2020 (k=177) | 0.02 | [0.02; 0.02] | . | 0.0146 | 0.1208 | 99% |
| Adding Peuchant, 2020 (k=178) | 0.02 | [0.02; 0.02] | . | 0.0147 | 0.1214 | 99% |
| Adding Jansen, 2020 (k=179) | 0.02 | [0.02; 0.03] | . | 0.0146 | 0.1209 | 99% |
| Adding Spornraft-Ragaller, 2020 (k=180) | 0.02 | [0.02; 0.03] | . | 0.0145 | 0.1206 | 99% |
| Adding Foschi, 2020 (k=181) | 0.02 | [0.02; 0.03] | . | 0.0144 | 0.1202 | 99% |
| Adding Achterbergh, 2020 (k=182) | 0.02 | [0.02; 0.03] | . | 0.0144 | 0.1199 | 99% |
| Adding Achterbergh, 2020 (k=183) | 0.03 | [0.02; 0.03] | . | 0.0143 | 0.1196 | 99% |
| Adding Druckler, 2020 (k=184) | 0.03 | [0.02; 0.03] | . | 0.0143 | 0.1195 | 99% |
| Adding Druckler, 2020 (k=185) | 0.03 | [0.02; 0.03] | . | 0.0143 | 0.1198 | 99% |
| Adding Druckler, 2020 (k=186) | 0.02 | [0.02; 0.03] | . | 0.0143 | 0.1197 | 99% |
| Adding Nijhuis, 2020 (k=187) | 0.02 | [0.02; 0.03] | . | 0.0143 | 0.1196 | 99% |
| Adding Nijhuis, 2020 (k=188) | 0.02 | [0.02; 0.03] | . | 0.0143 | 0.1195 | 99% |
| Adding Slurink, 2020 (k=189) | 0.02 | [0.02; 0.03] | . | 0.0142 | 0.1190 | 99% |
| Adding Slurink, 2020 (k=190) | 0.03 | [0.02; 0.03] | . | 0.0141 | 0.1187 | 99% |
| Adding Slurink, 2020 (k=191) | 0.03 | [0.02; 0.03] | . | 0.0140 | 0.1182 | 99% |
| Adding Slurink, 2020 (k=192) | 0.03 | [0.02; 0.03] | . | 0.0140 | 0.1183 | 99% |
| Adding Slurink, 2020 (k=193) | 0.03 | [0.03; 0.03] | . | 0.0139 | 0.1180 | 99% |
| Adding Slurink, 2020 (k=194) | 0.03 | [0.03; 0.03] | . | 0.0141 | 0.1187 | 99% |
| Adding Van Liere, 2020 (k=195) | 0.04 | [0.04; 0.04] | . | 0.0140 | 0.1184 | 99% |
| Adding Van Liere, 2020 (k=196) | 0.04 | [0.04; 0.04] | . | 0.0140 | 0.1183 | 99% |
| Adding Bernier, 2020 (k=197) | 0.04 | [0.04; 0.04] | . | 0.0140 | 0.1181 | 99% |
| Adding Ayerdi Aguirreb, 2020 (k=198) | 0.04 | [0.04; 0.04] | . | 0.0140 | 0.1184 | 99% |
| Adding Ayerdi Aguirreb, 2020 (k=199) | 0.04 | [0.04; 0.04] | . | 0.0140 | 0.1182 | 99% |
| Adding Cameron, 2020 (k=200) | 0.04 | [0.04; 0.04] | . | 0.0142 | 0.1190 | 99% |
| Adding King, 2020 (k=201) | 0.04 | [0.04; 0.04] | . | 0.0142 | 0.1190 | 99% |
| Adding King, 2020 (k=202) | 0.04 | [0.04; 0.04] | . | 0.0141 | 0.1188 | 99% |
| Adding Wilson, 2020 (k=203) | 0.04 | [0.04; 0.04] | . | 0.0140 | 0.1184 | 99% |
| Adding De Baetselier, 2021b (k=204) | 0.04 | [0.04; 0.04] | . | 0.0139 | 0.1181 | 99% |
| Adding Farfour, 2021 (k=205) | 0.04 | [0.04; 0.04] | . | 0.0139 | 0.1179 | 99% |
| Adding Farfour, 2021 (k=206) | 0.04 | [0.04; 0.04] | . | 0.0138 | 0.1175 | 99% |
| Adding Morgat, 2021 (k=207) | 0.04 | [0.04; 0.04] | . | 0.0138 | 0.1173 | 99% |
| Adding Skaletz-Rorowski, 2021 (k=208) | 0.04 | [0.04; 0.04] | . | 0.0137 | 0.1170 | 99% |
| Adding Marangoni, 2021 (k=209) | 0.04 | [0.04; 0.04] | . | 0.0138 | 0.1173 | 99% |
| Adding Marangoni, 2021 (k=210) | 0.04 | [0.04; 0.04] | . | 0.0141 | 0.1186 | 99% |
| Adding Marangoni, 2021 (k=211) | 0.04 | [0.04; 0.04] | . | 0.0142 | 0.1192 | 99% |
| Adding Marangoni, 2021 (k=212) | 0.04 | [0.04; 0.04] | . | 0.0148 | 0.1216 | 99% |
| Adding Marangoni, 2021 (k=213) | 0.04 | [0.04; 0.04] | . | 0.0149 | 0.1222 | 99% |
| Adding Druckler, 2021 (k=214) | 0.04 | [0.04; 0.04] | . | 0.0148 | 0.1219 | 99% |
| Adding Tielemans, 2021 (k=215) | 0.04 | [0.04; 0.04] | . | 0.0148 | 0.1216 | 99% |
| Adding Tielemans, 2021 (k=216) | 0.04 | [0.04; 0.04] | . | 0.0147 | 0.1213 | 99% |
| Adding Tielemans, 2021 (k=217) | 0.04 | [0.04; 0.04] | . | 0.0147 | 0.1210 | 99% |
| Adding Tielemans, 2021 (k=218) | 0.04 | [0.04; 0.04] | . | 0.0146 | 0.1208 | 99% |
| Adding Tyulenev, 2021 (k=219) | 0.04 | [0.04; 0.04] | . | 0.0145 | 0.1205 | 99% |
| Adding Aguirrebengoa, 2021 (k=220) | 0.04 | [0.04; 0.04] | . | 0.0148 | 0.1217 | 99% |
| Adding Andersson, 2021a (k=221) | 0.04 | [0.04; 0.04] | . | 0.0149 | 0.1220 | 99% |
| Adding Broad, 2021 (k=222) | 0.04 | [0.04; 0.04] | . | 0.0153 | 0.1236 | 99% |
| Adding Burton, 2021 (k=223) | 0.04 | [0.04; 0.04] | . | 0.0156 | 0.1247 | 99% |
| Adding Burton, 2021 (k=224) | 0.04 | [0.04; 0.04] | . | 0.0156 | 0.1247 | 99% |
| Adding Burton, 2021 (k=225) | 0.04 | [0.04; 0.04] | . | 0.0159 | 0.1263 | 99% |
| Adding Hatton, 2021 (k=226) | 0.04 | [0.04; 0.04] | . | 0.0164 | 0.1282 | 99% |
| Adding Hatton, 2021 (k=227) | 0.04 | [0.04; 0.04] | . | 0.0164 | 0.1280 | 99% |
| Adding Sultan, 2021 (k=228) | 0.04 | [0.04; 0.04] | . | 0.0165 | 0.1284 | 99% |
| Adding Trotman, 2021 (k=229) | 0.04 | [0.04; 0.04] | . | 0.0164 | 0.1281 | 99% |
| **Common effect model** | **0.04** | **[0.04; 0.04]** | **.** | **0.0164** | **0.1281** | **99%** |

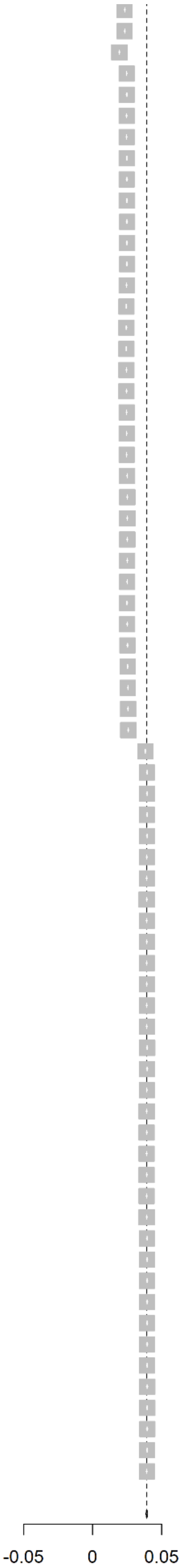

## C. Oropharyngeal

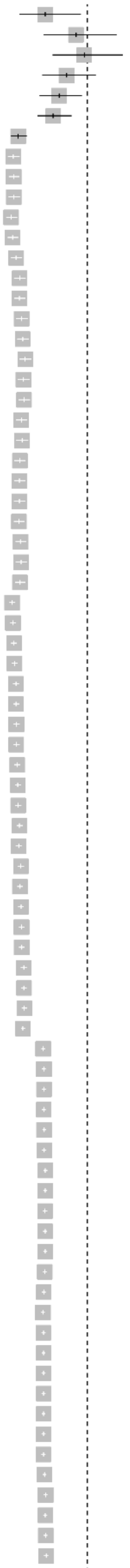

| Study | Proportion | 95%-CI | P-value | Tau2 | Tau | I2 |
|---|---|---|---|---|---|---|
| Adding Robinson, 1974 (k=1) | 0.02 | [0.01; 0.03] | . | . | . | . |
| Adding Shahidullah, 1976 (k=2) | 0.03 | [0.02; 0.05] | . | 0.0272 | 0.1649 | 95% |
| Adding Shahidullah, 1976 (k=3) | 0.03 | [0.02; 0.05] | . | 0.0131 | 0.1146 | 91% |
| Adding Reimann, 1980 (k=4) | 0.03 | [0.02; 0.04] | . | 0.0124 | 0.1112 | 88% |
| Adding Reimann, 1980 (k=5) | 0.02 | [0.02; 0.03] | . | 0.0097 | 0.0987 | 85% |
| Adding Bovet, 1982 (k=6) | 0.02 | [0.01; 0.03] | . | 0.0079 | 0.0889 | 82% |
| Adding Gonzalez, 1983 (k=7) | 0.01 | [0.00; 0.01] | . | 0.0112 | 0.1059 | 93% |
| Adding Gonzalez, 1983 (k=8) | 0.00 | [0.00; 0.01] | . | 0.0108 | 0.1042 | 93% |
| Adding Christophersen, 1988 (k=9) | 0.00 | [0.00; 0.01] | . | 0.0094 | 0.0968 | 92% |
| Adding Christophersen, 1988 (k=10) | 0.00 | [0.00; 0.01] | . | 0.0083 | 0.0909 | 91% |
| Adding Christophersen, 1988 (k=11) | 0.00 | [0.00; 0.01] | . | 0.0080 | 0.0896 | 90% |
| Adding Stary, 1991 (k=12) | 0.00 | [0.00; 0.01] | . | 0.0071 | 0.0844 | 89% |
| Adding Ross, 1991a (k=13) | 0.01 | [0.00; 0.01] | . | 0.0064 | 0.0800 | 90% |
| Adding Ross, 1991a (k=14) | 0.01 | [0.01; 0.01] | . | 0.0060 | 0.0772 | 90% |
| Adding Ross, 1991a (k=15) | 0.01 | [0.01; 0.01] | . | 0.0056 | 0.0749 | 90% |
| Adding Ross, 1991a (k=16) | 0.01 | [0.01; 0.01] | . | 0.0053 | 0.0727 | 90% |
| Adding Ross, 1991a (k=17) | 0.01 | [0.01; 0.01] | . | 0.0048 | 0.0696 | 89% |
| Adding Ross, 1991a (k=18) | 0.01 | [0.01; 0.01] | . | 0.0048 | 0.0690 | 89% |
| Adding Tomlinson, 1991 (k=19) | 0.01 | [0.01; 0.01] | . | 0.0046 | 0.0679 | 89% |
| Adding Vlaspolder, 1993 (k=20) | 0.01 | [0.01; 0.01] | . | 0.0044 | 0.0667 | 88% |
| Adding Vlaspolder, 1993 (k=21) | 0.01 | [0.01; 0.01] | . | 0.0047 | 0.0686 | 88% |
| Adding Vlaspolder, 1993 (k=22) | 0.01 | [0.01; 0.01] | . | 0.0045 | 0.0673 | 88% |
| Adding Vlaspolder, 1993 (k=23) | 0.01 | [0.01; 0.01] | . | 0.0046 | 0.0677 | 87% |
| Adding Van der Snoek, 2004 (k=24) | 0.01 | [0.01; 0.01] | . | 0.0046 | 0.0675 | 87% |
| Adding Van der Snoek, 2004 (k=25) | 0.01 | [0.01; 0.01] | . | 0.0044 | 0.0663 | 86% |
| Adding Manavi, 2004 (k=26) | 0.01 | [0.00; 0.01] | . | 0.0043 | 0.0659 | 86% |
| Adding Manavi, 2004 (k=27) | 0.01 | [0.01; 0.01] | . | 0.0041 | 0.0641 | 86% |
| Adding Manavi, 2005 (k=28) | 0.01 | [0.01; 0.01] | . | 0.0047 | 0.0685 | 86% |
| Adding Papadogeorgaki, 2006 (k=29) | 0.01 | [0.01; 0.01] | . | 0.0051 | 0.0711 | 86% |
| Adding Heijman, 2007 (k=30) | 0.00 | [0.00; 0.01] | . | 0.0049 | 0.0702 | 87% |
| Adding Benn, 2007 (k=31) | 0.00 | [0.00; 0.01] | . | 0.0059 | 0.0769 | 88% |
| Adding Benn, 2007 (k=32) | 0.00 | [0.00; 0.01] | . | 0.0061 | 0.0779 | 89% |
| Adding Benn, 2007 (k=33) | 0.01 | [0.00; 0.01] | . | 0.0064 | 0.0799 | 89% |
| Adding Linhart, 2008 (k=34) | 0.01 | [0.00; 0.01] | . | 0.0070 | 0.0835 | 91% |
| Adding Soni, 2008 (k=35) | 0.01 | [0.00; 0.01] | . | 0.0068 | 0.0826 | 90% |
| Adding Soni, 2008 (k=36) | 0.01 | [0.00; 0.01] | . | 0.0066 | 0.0812 | 90% |
| Adding Shipitsyna, 2009 (k=37) | 0.01 | [0.00; 0.01] | . | 0.0067 | 0.0821 | 90% |
| Adding Annan, 2009 (k=38) | 0.01 | [0.01; 0.01] | . | 0.0065 | 0.0808 | 90% |
| Adding Walsh, 2011b (k=39) | 0.01 | [0.01; 0.01] | . | 0.0068 | 0.0825 | 90% |
| Adding Walsh, 2011b (k=40) | 0.01 | [0.01; 0.01] | . | 0.0066 | 0.0812 | 90% |
| Adding Heiligenberg, 2011b (k=41) | 0.01 | [0.01; 0.01] | . | 0.0064 | 0.0801 | 91% |
| Adding Heiligenberg, 2011b (k=42) | 0.01 | [0.01; 0.01] | . | 0.0066 | 0.0810 | 91% |
| Adding Peters, 2011 (k=43) | 0.01 | [0.01; 0.01] | . | 0.0064 | 0.0800 | 91% |
| Adding Peters, 2011 (k=44) | 0.01 | [0.01; 0.01] | . | 0.0064 | 0.0797 | 91% |
| Adding Soni, 2011 (k=45) | 0.01 | [0.01; 0.01] | . | 0.0062 | 0.0787 | 91% |
| Adding Bozicevic, 2012 (k=46) | 0.01 | [0.01; 0.01] | . | 0.0060 | 0.0776 | 91% |
| Adding Skovgaard, 2012 (k=47) | 0.01 | [0.01; 0.01] | . | 0.0059 | 0.0765 | 91% |
| Adding Skovgaard, 2012 (k=48) | 0.01 | [0.01; 0.01] | . | 0.0058 | 0.0763 | 92% |
| Adding Heiligenberg, 2012 (k=49) | 0.01 | [0.01; 0.01] | . | 0.0057 | 0.0753 | 92% |
| Adding Heiligenberg, 2012 (k=50) | 0.01 | [0.01; 0.01] | . | 0.0055 | 0.0744 | 92% |
| Adding Koedijk, 2012 (k=51) | 0.01 | [0.01; 0.01] | . | 0.0054 | 0.0738 | 92% |
| Adding Koedijk, 2012 (k=52) | 0.02 | [0.02; 0.02] | . | 0.0053 | 0.0730 | 97% |
| Adding Reinton, 2013 (k=53) | 0.02 | [0.02; 0.02] | . | 0.0052 | 0.0722 | 97% |
| Adding Jeverica, 2013 (k=54) | 0.02 | [0.02; 0.02] | . | 0.0051 | 0.0713 | 97% |
| Adding Jeverica, 2013 (k=55) | 0.02 | [0.02; 0.02] | . | 0.0050 | 0.0707 | 97% |
| Adding Jimenez, 2013 (k=56) | 0.02 | [0.02; 0.02] | . | 0.0054 | 0.0731 | 97% |
| Adding Roberts, 2013 (k=57) | 0.02 | [0.02; 0.02] | . | 0.0053 | 0.0730 | 97% |
| Adding Dudareva-Vizule, 2014 (k=58) | 0.02 | [0.02; 0.02] | . | 0.0053 | 0.0730 | 97% |
| Adding Keaveney, 2014 (k=59) | 0.02 | [0.02; 0.02] | . | 0.0054 | 0.0734 | 97% |
| Adding Keaveney, 2014 (k=60) | 0.02 | [0.02; 0.02] | . | 0.0054 | 0.0735 | 97% |
| Adding De Vrieze, 2014 (k=61) | 0.02 | [0.02; 0.02] | . | 0.0053 | 0.0728 | 97% |
| Adding De Vrieze, 2014 (k=62) | 0.02 | [0.02; 0.02] | . | 0.0053 | 0.0729 | 97% |
| Adding Vahidnia, 2014 (k=63) | 0.02 | [0.02; 0.02] | . | 0.0053 | 0.0727 | 97% |
| Adding Vahidnia, 2014 (k=64) | 0.02 | [0.02; 0.02] | . | 0.0053 | 0.0725 | 97% |
| Adding Vahidnia, 2014 (k=65) | 0.02 | [0.02; 0.02] | . | 0.0052 | 0.0723 | 96% |
| Adding Van Liere, 2014a (k=66) | 0.02 | [0.02; 0.02] | . | 0.0051 | 0.0715 | 96% |
| Adding Van Liere, 2014a (k=67) | 0.02 | [0.02; 0.02] | . | 0.0050 | 0.0707 | 96% |
| Adding Hagemann, 2014 (k=68) | 0.02 | [0.02; 0.02] | . | 0.0050 | 0.0707 | 96% |
| Adding Perez-Hernandez, 2014 (k=69) | 0.02 | [0.02; 0.02] | . | 0.0049 | 0.0701 | 96% |
| Adding Sprenger, 2014 (k=70) | 0.02 | [0.02; 0.02] | . | 0.0049 | 0.0697 | 96% |
| Adding Sprenger, 2014 (k=71) | 0.02 | [0.02; 0.02] | . | 0.0048 | 0.0693 | 96% |
| Adding Sprenger, 2014 (k=72) | 0.02 | [0.02; 0.02] | . | 0.0048 | 0.0692 | 96% |
| Adding Dakshina, 2014 (k=73) | 0.02 | [0.02; 0.02] | . | 0.0053 | 0.0731 | 96% |
| Adding Dakshina, 2014 (k=74) | 0.02 | [0.02; 0.02] | . | 0.0058 | 0.0764 | 97% |
| Adding Fraser, 2014 (k=75) | 0.02 | [0.02; 0.02] | . | 0.0058 | 0.0761 | 97% |
| Adding Plaas, 2015 (k=76) | 0.02 | [0.02; 0.02] | . | 0.0063 | 0.0795 | 97% |
| Adding Marcus, 2015 (k=77) | 0.02 | [0.02; 0.02] | . | 0.0063 | 0.0791 | 97% |

| | | | | | | |
|---|---|---|---|---|---|---|
| Adding Spauwen, 2015 (k=78) | 0.02 | [0.02; 0.02] | . | 0.0062 | 0.0786 | 97% |
| Adding Van Liere, 2015 (k=79) | 0.02 | [0.02; 0.02] | . | 0.0222 | 0.1491 | 98% |
| Adding Van Rooijen, 2015 (k=80) | 0.02 | [0.02; 0.02] | . | 0.0220 | 0.1482 | 98% |
| Adding Van Rooijen, 2015 (k=81) | 0.02 | [0.02; 0.02] | . | 0.0219 | 0.1479 | 98% |
| Adding Van Rooijen, 2015 (k=82) | 0.02 | [0.02; 0.02] | . | 0.0217 | 0.1472 | 98% |
| Adding Van Rooijen, 2015 (k=83) | 0.02 | [0.02; 0.02] | . | 0.0215 | 0.1465 | 98% |
| Adding Verscheijden, 2015 (k=84) | 0.02 | [0.02; 0.02] | . | 0.0213 | 0.1458 | 98% |
| Adding Verscheijden, 2015 (k=85) | 0.02 | [0.02; 0.02] | . | 0.0210 | 0.1451 | 98% |
| Adding Verscheijden, 2015 (k=86) | 0.02 | [0.02; 0.02] | . | 0.0208 | 0.1443 | 98% |
| Adding Verscheijden, 2015 (k=87) | 0.02 | [0.02; 0.02] | . | 0.0206 | 0.1434 | 98% |
| Adding Verscheijden, 2015 (k=88) | 0.02 | [0.02; 0.02] | . | 0.0203 | 0.1426 | 98% |
| Adding Verscheijden, 2015 (k=89) | 0.02 | [0.02; 0.02] | . | 0.0201 | 0.1418 | 98% |
| Adding Lenart, 2015 (k=90) | 0.02 | [0.02; 0.02] | . | 0.0200 | 0.1413 | 98% |
| Adding Lenart, 2015 (k=91) | 0.02 | [0.02; 0.02] | . | 0.0198 | 0.1406 | 98% |
| Adding Lenart, 2015 (k=92) | 0.02 | [0.02; 0.02] | . | 0.0196 | 0.1398 | 98% |
| Adding Garner, 2015 (k=93) | 0.02 | [0.02; 0.02] | . | 0.0193 | 0.1391 | 98% |
| Adding Garner, 2015 (k=94) | 0.02 | [0.02; 0.02] | . | 0.0192 | 0.1387 | 98% |
| Adding Garner, 2015 (k=95) | 0.02 | [0.02; 0.02] | . | 0.0192 | 0.1385 | 98% |
| Adding Garner, 2015 (k=96) | 0.02 | [0.02; 0.02] | . | 0.0191 | 0.1381 | 98% |
| Adding Garner, 2015 (k=97) | 0.02 | [0.02; 0.02] | . | 0.0190 | 0.1378 | 98% |
| Adding Garner, 2015 (k=98) | 0.02 | [0.02; 0.02] | . | 0.0189 | 0.1374 | 98% |
| Adding Thorsteinsson, 2016 (k=99) | 0.02 | [0.02; 0.02] | . | 0.0189 | 0.1377 | 98% |
| Adding Jaureguy, 2016 (k=100) | 0.02 | [0.02; 0.02] | . | 0.0188 | 0.1370 | 98% |
| Adding Sultan, 2016 (k=101) | 0.02 | [0.02; 0.02] | . | 0.0190 | 0.1379 | 98% |
| Adding Edouard, 2017b (k=102) | 0.02 | [0.02; 0.02] | . | 0.0189 | 0.1373 | 98% |
| Adding Fata, 2017 (k=103) | 0.02 | [0.02; 0.02] | . | 0.0187 | 0.1366 | 98% |
| Adding Den Heijer, 2017 (k=104) | 0.02 | [0.02; 0.02] | . | 0.0185 | 0.1359 | 98% |
| Adding Den Heijer, 2017 (k=105) | 0.02 | [0.02; 0.02] | . | 0.0183 | 0.1353 | 98% |
| Adding Spinner, 2018 (k=106) | 0.02 | [0.02; 0.02] | . | 0.0181 | 0.1346 | 98% |
| Adding Druckler, 2018 (k=107) | 0.02 | [0.02; 0.02] | . | 0.0180 | 0.1341 | 98% |
| Adding Matser, 2018 (k=108) | 0.02 | [0.02; 0.02] | . | 0.0178 | 0.1335 | 98% |
| Adding Van Rooijen, 2018 (k=109) | 0.02 | [0.02; 0.02] | . | 0.0178 | 0.1332 | 98% |
| Adding Van Rooijen, 2018 (k=110) | 0.02 | [0.02; 0.02] | . | 0.0176 | 0.1327 | 98% |
| Adding Van Rooijen, 2018 (k=111) | 0.02 | [0.02; 0.02] | . | 0.0175 | 0.1323 | 98% |
| Adding Van Rooijen, 2018 (k=112) | 0.02 | [0.02; 0.03] | . | 0.0173 | 0.1317 | 99% |
| Adding Dona, 2018 (k=113) | 0.02 | [0.02; 0.03] | . | 0.0190 | 0.1379 | 99% |
| Adding Jeffery, 2018 (k=114) | 0.02 | [0.02; 0.03] | . | 0.0190 | 0.1378 | 99% |
| Adding Rahib, 2019 (k=115) | 0.02 | [0.02; 0.03] | . | 0.0189 | 0.1374 | 99% |
| Adding Hoornenborg, 2019 (k=116) | 0.02 | [0.02; 0.03] | . | 0.0187 | 0.1368 | 99% |
| Adding Ribeiro, 2019 (k=117) | 0.02 | [0.02; 0.03] | . | 0.0185 | 0.1362 | 99% |
| Adding De Salazar, 2019 (k=118) | 0.02 | [0.02; 0.03] | . | 0.0184 | 0.1356 | 99% |
| Adding Cherkaoui, 2019 (k=119) | 0.02 | [0.02; 0.03] | . | 0.0193 | 0.1390 | 99% |
| Adding De Baetselier, 2020 (k=120) | 0.02 | [0.02; 0.03] | . | 0.0192 | 0.1386 | 99% |
| Adding Verougstraete, 2020 (k=121) | 0.02 | [0.02; 0.03] | . | 0.0190 | 0.1380 | 99% |
| Adding Jansen, 2020 (k=122) | 0.02 | [0.02; 0.03] | . | 0.0189 | 0.1373 | 99% |
| Adding Rich, 2020 (k=123) | 0.02 | [0.02; 0.03] | . | 0.0187 | 0.1368 | 99% |
| Adding Rich, 2020 (k=124) | 0.02 | [0.02; 0.03] | . | 0.0186 | 0.1362 | 99% |
| Adding Foschi, 2020 (k=125) | 0.02 | [0.02; 0.03] | . | 0.0184 | 0.1358 | 99% |
| Adding Marangoni, 2020 (k=126) | 0.02 | [0.02; 0.03] | . | 0.0198 | 0.1407 | 99% |
| Adding Achterbergh, 2020 (k=127) | 0.03 | [0.02; 0.03] | . | 0.0196 | 0.1402 | 99% |
| Adding Achterbergh, 2020 (k=128) | 0.03 | [0.02; 0.03] | . | 0.0195 | 0.1395 | 99% |
| Adding Druckler, 2020 (k=129) | 0.03 | [0.02; 0.03] | . | 0.0193 | 0.1391 | 99% |
| Adding Druckler, 2020 (k=130) | 0.03 | [0.02; 0.03] | . | 0.0193 | 0.1388 | 99% |
| Adding Druckler, 2020 (k=131) | 0.03 | [0.02; 0.03] | . | 0.0192 | 0.1384 | 99% |
| Adding Druckler, 2020 (k=132) | 0.03 | [0.02; 0.03] | . | 0.0190 | 0.1379 | 99% |
| Adding Nijhuis, 2020 (k=133) | 0.03 | [0.02; 0.03] | . | 0.0189 | 0.1375 | 99% |
| Adding Nijhuis, 2020 (k=134) | 0.02 | [0.02; 0.03] | . | 0.0188 | 0.1373 | 99% |
| Adding Slurink, 2020 (k=135) | 0.03 | [0.02; 0.03] | . | 0.0187 | 0.1367 | 98% |
| Adding Slurink, 2020 (k=136) | 0.03 | [0.02; 0.03] | . | 0.0185 | 0.1361 | 98% |
| Adding Slurink, 2020 (k=137) | 0.03 | [0.02; 0.03] | . | 0.0184 | 0.1355 | 98% |
| Adding Slurink, 2020 (k=138) | 0.03 | [0.02; 0.03] | . | 0.0182 | 0.1350 | 98% |
| Adding Slurink, 2020 (k=139) | 0.03 | [0.03; 0.03] | . | 0.0181 | 0.1345 | 98% |
| Adding Slurink, 2020 (k=140) | 0.03 | [0.03; 0.03] | . | 0.0181 | 0.1344 | 98% |

| | | | | | | |
|---|---|---|---|---|---|---|
| Adding Van Liere, 2020 (k=141) | 0.03 | [0.03; 0.03] | . | 0.0212 | 0.1455 | 100% |
| Adding Almeida, 2020 (k=142) | 0.03 | [0.03; 0.03] | . | 0.0211 | 0.1451 | 100% |
| Adding Bernier, 2020 (k=143) | 0.03 | [0.03; 0.03] | . | 0.0210 | 0.1450 | 100% |
| Adding Ayerdi Aguirreb, 2020 (k=144) | 0.03 | [0.03; 0.03] | . | 0.0213 | 0.1461 | 100% |
| Adding Ayerdi Aguirreb, 2020 (k=145) | 0.03 | [0.03; 0.03] | . | 0.0212 | 0.1455 | 100% |
| Adding Kerry-Barnard, 2020 (k=146) | 0.03 | [0.03; 0.03] | . | 0.0212 | 0.1456 | 100% |
| Adding Kerry-Barnard, 2020 (k=147) | 0.03 | [0.03; 0.03] | . | 0.0212 | 0.1455 | 100% |
| Adding King, 2020 (k=148) | 0.03 | [0.03; 0.03] | . | 0.0212 | 0.1457 | 100% |
| Adding King, 2020 (k=149) | 0.03 | [0.03; 0.03] | . | 0.0213 | 0.1461 | 100% |
| Adding Wilson, 2020 (k=150) | 0.03 | [0.03; 0.03] | . | 0.0212 | 0.1455 | 100% |
| Adding De Baetselier, 2021b (k=151) | 0.03 | [0.03; 0.03] | . | 0.0210 | 0.1451 | 100% |
| Adding Farfour, 2021 (k=152) | 0.03 | [0.03; 0.03] | . | 0.0209 | 0.1446 | 100% |
| Adding Farfour, 2021 (k=153) | 0.03 | [0.03; 0.03] | . | 0.0208 | 0.1441 | 100% |
| Adding Morgat, 2021 (k=154) | 0.03 | [0.03; 0.03] | . | 0.0206 | 0.1436 | 100% |
| Adding Skaletz-Rorowski, 2021 (k=155) | 0.03 | [0.03; 0.03] | . | 0.0205 | 0.1431 | 100% |
| Adding Druckler, 2021 (k=156) | 0.03 | [0.03; 0.03] | . | 0.0203 | 0.1426 | 100% |
| Adding Tielemans, 2021 (k=157) | 0.03 | [0.03; 0.03] | . | 0.0202 | 0.1422 | 100% |
| Adding Tielemans, 2021 (k=158) | 0.03 | [0.03; 0.04] | . | 0.0201 | 0.1418 | 100% |
| Adding Tielemans, 2021 (k=159) | 0.03 | [0.03; 0.04] | . | 0.0202 | 0.1421 | 100% |
| Adding Tielemans, 2021 (k=160) | 0.04 | [0.03; 0.04] | . | 0.0202 | 0.1423 | 100% |
| Adding Tyulenev, 2021 (k=161) | 0.04 | [0.03; 0.04] | . | 0.0201 | 0.1418 | 100% |
| Adding Aguirrebengoa, 2021 (k=162) | 0.04 | [0.03; 0.04] | . | 0.0202 | 0.1422 | 100% |
| Adding Broad, 2021 (k=163) | 0.04 | [0.03; 0.04] | . | 0.0204 | 0.1428 | 100% |
| Adding Burton, 2021 (k=164) | 0.04 | [0.03; 0.04] | . | 0.0213 | 0.1461 | 100% |
| Adding Burton, 2021 (k=165) | 0.04 | [0.03; 0.04] | . | 0.0212 | 0.1456 | 99% |
| Adding Burton, 2021 (k=166) | 0.04 | [0.03; 0.04] | . | 0.0215 | 0.1466 | 99% |
| Adding Sultan, 2021 (k=167) | 0.04 | [0.03; 0.04] | . | 0.0216 | 0.1468 | 99% |
| **Common effect model** | **0.04** | **[0.03; 0.04]** | **.** | **0.0216** | **0.1468** | **99%** |

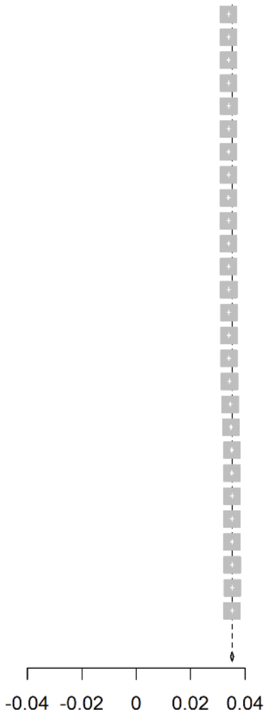

**References**

1. Page MJ, McKenzie JE, Bossuyt PM, Boutron I, Hoffmann TC, Mulrow CD, et al. The PRISMA 2020 statement: an updated guideline for reporting systematic reviews. Bmj. 2021;372.

2. Hoy D, Brooks P, Woolf A, Blyth F, March L, Bain C, et al. Assessing risk of bias in prevalence studies: modification of an existing tool and evidence of interrater agreement. Journal of Clinical Epidemiology. 2012;65(9):934-9.

3. World Bank. World bank country and lending groups 2020. https://datahelpdesk.worldbank.org/knowledgebase/articles/906519-world-bank-country-and-lending-groups (accessed January 21, 2023).
